# Knowledge-Guided Bioinformatics Model for Identifying Autism Spectrum Disorder Diagnostic MicroRNA Biomarkers

**DOI:** 10.1038/srep39663

**Published:** 2016-12-21

**Authors:** Li Shen, Yuxin Lin, Zhandong Sun, Xuye Yuan, Luonan Chen, Bairong Shen

**Affiliations:** 1 Center for Systems Biology, Soochow University, Suzhou, 215006, China; 2 Institute of Biological Sciences and Biotechnology, Donghua University, Shanghai, 201620, China; 3 Key laboratory of Systems Biology, Shanghai Institute of Biological Sciences, Chinese Academy of Sciences, Shanghai, 200031, China

## Abstract

Autism spectrum disorder (ASD) is a severe neurodevelopmental disease with a high incidence and effective biomarkers are urgently needed for its diagnosis. A few previous studies have reported the detection of miRNA biomarkers for autism diagnosis, especially those based on bioinformatics approaches. In this study, we developed a knowledge-guided bioinformatics model for identifying autism miRNA biomarkers. We downloaded gene expression microarray data from the GEO Database and extracted genes with expression levels that differed in ASD and the controls. We then constructed an autism-specific miRNA–mRNA network and inferred candidate autism biomarker miRNAs based on their regulatory modes and functions. We defined a novel parameter called the autism gene percentage as autism-specific knowledge to further facilitate the identification of autism-specific biomarker miRNAs. Finally, 11 miRNAs were screened as putative autism biomarkers, where eight miRNAs (72.7%) were significantly dysregulated in ASD samples according to previous reports. Functional enrichment results indicated that the targets of the identified miRNAs were enriched in autism-associated pathways, such as Wnt signaling (in KEGG and IPA), cell cycle (in KEGG), and glioblastoma multiforme signaling (in IPA), thereby supporting the predictive power of our model.

Autism spectrum disorder (ASD) is a severe neurodevelopmental disorder, which has attracted increasing public attention in recent years. ASD has been defined as a common developmental disorder that occurs in children according to the *Diagnostic and Statistical Manual IV (DSM-IV)*. In general, it is characterized by defective communication and social interaction, abnormal narrow interests, and repetitive behavior^1^. A recent study showed that the incidence rate of ASD was 1.47% in the USA during 2010^2^. Due to the heterogeneous nature of this disorder, a large number of studies have aimed to understand its mechanisms at different levels. In recent years, an increasing number of studies have found hundreds of genes that may contribute to autism and relevant phenotypes^3^. However, only a few of these studies considered autism-associated microRNAs (miRNAs), especially miRNA biomarkers.

miRNAs are small non-coding regulatory RNAs that measure 20–25 nucleotides in length. Abnormal changes in miRNA expression are known to be associated with the occurrence and development of human diseases^4^. Mundalil *et al*. used serum samples without drug treatment to conduct miRNA expression profiling and found several significant differentially expressed miRNAs that could be potential biomarkers^5^. Marrale *et al*. and Vaishnavi *et al*. reported that copy number variants or single nucleotide polymorphisms in miRNAs were correlated with ASD^6^,^7^. Nguyen *et al*. reported four miRNAs that are deregulated in ASD^8^. Hicks *et al*. screened 14 salivary miRNAs as accurate indicators of ASD^9^. Wu *et al*. investigated the miRNA expression profiles in post-mortem brains from ASD patients and confirmed the function of miRNAs in ASD pathophysiology^10^.

We investigated ASD-associated miRNAs in our previous study and found that almost no consensus miRNA sets could be extracted. Thus, similar to prostate cancer^11^, ASD seems to be a complex disease where the dysregulation of miRNAs is generally heterogeneous due to different genetic and non-genetic risk factors. Many mechanisms can lead to ASD and diverse miRNA expression profiles are used as factors to identify associations with ASD. In order to discover diagnostic biomarkers, an association with the disease is the only necessary but not sufficient condition for a biomarker.

Most current methods for miRNA biomarker discovery are based on experimental screening. High-throughput expression profiling is often used to identify differentially expressed genes in disease samples and controls, before low-throughput experimental validation is employed to further refine the candidate sets. However, discovering miRNA biomarkers in the laboratory is expensive and time-consuming due to the complexity and diversity of disease progression. Given the accumulated data and knowledge regarding ASD, it is now possible as well as necessary to develop a computational and bioinformatics model to identify biomarkers that may support the precise diagnosis of ASD.

Computational or bioinformatics models for biomarker discovery are often based on machine learning and statistical inference. For example, Cogill and Wang trained a support vector machine model using developmental brain gene expression data to prioritize candidate autism genes^12^, but the models built using a training-and-testing procedure were often overfitted and their generalizability was poor because the data used for training and testing were not always representative of the heterogeneity of this complex disease. However, models built according to knowledge-guided and mechanism-based analyses are more useful for biomarker discovery in complex diseases. To discover miRNA biomarkers, we previously proposed a model called Pipeline of Outlier MicroRNA Analysis (POMA) based on an integrative analysis of the substructure of a miRNA–mRNA regulatory network and disease-specific expression profiles, and this model was applied successfully to miRNA biomarker discovery in different cancers^13^,^14^,^15^,^16^. In the present study, we extended and improved the POMA model to discover miRNA biomarkers for autism.

## Materials and Methods

### Knowledge-guided model (improved POMA model)

The POMA model enriches for weak sites in a miRNA-mRNA regulatory network and considers the biological functions of miRNA targets. Two feature parameters, i.e., the novel out degree (NOD) and transcription factor gene percentage (TFP), were defined to quantify the regulatory power of miRNAs in an autism-specific miRNA–mRNA network, where the former represents the numbers of genes that are independently regulated by an individual miRNA and the latter is the percentage of transcription factor (TF) genes targeted by given miRNAs. Statistical evidence indicates that miRNAs with high NOD and TFP values are likely to be biomarkers, and applications to miRNA biomarker discovery in prostate cancer^15^,^16^, clear cell renal cell carcinoma^13^, sepsis^17^, and pediatric acute myeloid leukemia^14^ support the predictive power.

**Figure 1:**
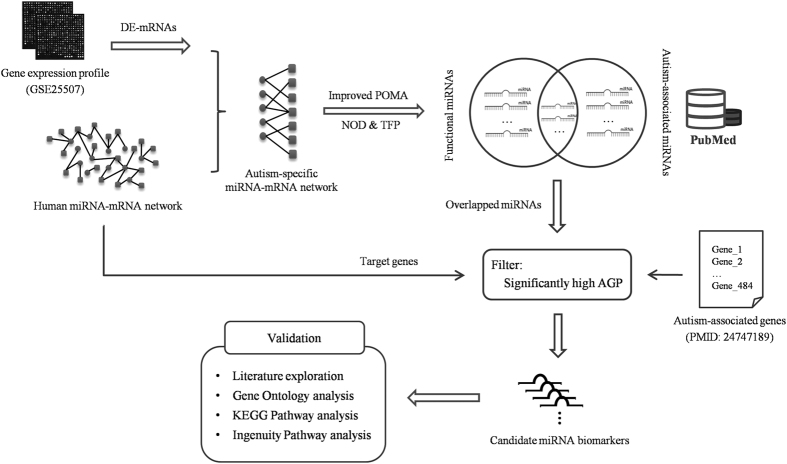
Schematic of the pipeline employed for identifying miRNAs as candidate autism biomarkers.

To employ autism-specific knowledge to improve our ability to discover autism biomarkers, we defined a novel parameter called the autism gene percentage (AGP), which is numerically equivalent to the percentage of autism-associated genes in the miRNA targets. We assumed that miRNAs with high AGP values are more closely related to the initiation and progression of autism, and thus they are more suitable for use as biomarkers. The novel AGP index made the model more specific to autism miRNA biomarker discovery, thereby improving the precision of the predictions, and we refer to it as the improved POMA model. A schematic illustrating the pipeline of our method is shown in Fig. 1.

### Dataset collection

Gene expression profile GSE25507^18^ was generated by an Affymetrix Human Genome U133 Plus 2.0 Array was selected from the Gene Expression Omnibus (GEO)^19^. The dataset contained 82 autism samples and 64 controls, where the gene samples were extracted from peripheral blood lymphocytes, and the normalized data were downloaded directly from the GEO database.

### Differentially expressed gene extraction

The Limma R package was utilized to identify differentially expressed genes (DE-mRNAs) based on the selected dataset^20^. The expression levels of genes with multiple probes equaled their average probe intensity. The Student’s *t*-test was used to calculate the significant differences (*p*-value) and fold changes were determined according to the average expression level of each gene in the autism and control groups, respectively. A *p*-value <0.05 and |fold-change|>2 were used as the cut-off criteria.

### Autism-specific miRNA–mRNA network construction

A human miRNA–mRNA regulatory network was built based on miRNA–mRNA pairs mined from experimentally validated (i.e., miRTarBase^21^, miRecords^22^, miR2Disease^23^, and TarBase^24^) and computationally predicted databases (i.e., starBase^25^, HOCTAR^26^, and ExprTargetDB^27^). This network was used as a reference for autism-specific miRNA–mRNA network construction. In the next step, DE-mRNAs were mapped onto the network and the unaltered mRNAs were trimmed, where the remainder comprised the autism-specific miRNA–mRNA network.

### Functional miRNA detection

We employed our in-house POMA model to detect key miRNAs with functions in autism progression (see the Introduction). First, we extracted all of the miRNAs with significantly high NOD values (*p*-value <0.05, Wilcoxon signed-rank test) in the autism-specific miRNA–mRNA network. These miRNAs had higher independent regulatory abilities so they were assumed to be sufficiently powerful to affect the system’s stability. Next, we selected the miRNAs with significantly high TFP values (*p*-value <0.05, Wilcoxon signed-rank test) from those screened in the first step. These miRNAs regulated more TF genes in addition to their independent regulatory power, and thus they were considered as autism functional outliers.

### Autism-associated miRNA mining

We manually searched for citations in PubMed in order to determine whether the identified functional miRNAs had reported associations with autism. In this case, “association” meant: 1) miRNAs with differential expression levels in autism samples compared with the normal controls^28^,^29^; OR 2) putative autism functional miRNAs inferred by computational methods^30^.

### Candidate miRNA biomarker identification

The targets of the miRNAs were extracted from the human miRNA–mRNA regulatory network. To further investigate the specificity of these genes, we defined a novel parameter called AGP, which represents the percentage of autism-associated genes targeted by a single miRNA. Finally, miRNAs with significantly high AGP values (*p*-value <0.05, Wilcoxon signed-rank test) were identified as candidate biomarkers.

### Functional enrichment analyses

Gene Ontology (GO) and pathway analysis were performed to validate the relationships between the targets of identified miRNA biomarkers and autism. The online Database for Annotation, Visualization and Integrated Discovery (DAVID, version 6.7)^31^ was used for GO annotation and the Kyoto Encyclopedia of Genes and Genomes (KEGG)^32^ pathway analysis. In addition, the commercial Ingenuity Pathway Analysis (IPA) program was used for pathway enrichment analyses. The Bonferroni method was used to adjust raw *p*-values and terms with adjusted *p*-values (adj.p-value) <0.05 were considered statistically significant and subjected to further analysis. Pathways highly connected with cancers were removed and the top 10 remaining significantly enriched pathways were selected and their correlations with autism were further confirmed by PubMed literature searches.

## Results

### Biomarker miRNAs for autism diagnosis

We selected 2,768 genes with different expression levels in ASD and the controls. The autism-specific miRNA–mRNA network contained 6,719 miRNA-mRNA pairs among 472 miRNAs and 1,138 genes. We identified 66 autism functional miRNAs based on our POMA model, 30 of which had reported associations with autism according to citations in PubMed (see Table S1).

**Figure 2:**
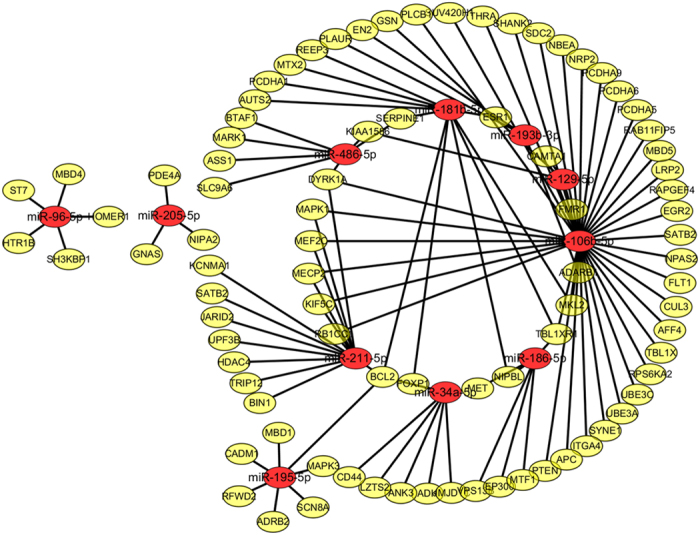
Autism-associated genes targeted by the identified miRNA biomarkers. Ellipses in red and yellow represent miRNAs and genes, respectively. In the network, the genes in the inner circle are regulated by at least two of the miRNAs.

We employed the specific AGP parameter to measure the power of miRNAs that regulate autism-associated genes. In total, 484 autism-associated genes were extracted from the study by Vaishnavi *et al*.^7^. We also screened candidate miRNA biomarkers from the 30 miRNAs based on our improved POMA model, i.e., considering the AGP distribution in the miRNA targets, we finally obtained 11 miRNAs (i.e., miR-193b-3p, miR-186–5p, miR-486-5p, miR-129-5p, miR-106b-5p, miR-181b-5p, miR-34a-5p, miR-96-5p, miR-211-5p, miR-205-5p, and miR-195-5p, as listed in Table 1) with significantly high AGP values as biomarkers. As shown in Fig. 2, key genes (e.g., PTEN, MeCP2, and SHANK3^33^,^34^,^35^) with important roles during autism development were regulated directly by these miRNAs.

### Literature validation of identified miRNA biomarkers

All 11 miRNAs were experimentally supported or they had inferred connections with autism according to our research pipeline. In particular, miR-181b-5p, miR-106b-5p, and miR-195-5p were validated by Mundalil Vasu *et al*. as candidate miRNA biomarkers for autism^5^. In their experiment, total RNA was extracted from the serum samples of autism patients and control groups, where they examined the expression levels of miRNAs and finally selected 14 differentially expressed miRNAs as autism candidates. The experimental result indicated that miR-181b-5p was downregulated in autism serum samples whereas the other two miRNAs were significantly upregulated.

Wu *et al*. performed genome-wide miRNA expression profiling and screened key miRNAs with differential expression in ASD brain samples using a linear mixed-effects model^10^. We found that seven of the miRNAs in our results, i.e., miR-193b-3p, miR-486-5p, miR-129-5p, miR-181b-5p, miR-34a-5p, miR-96-5p, and miR-195-5p, were also dysregulated in the study by Wu *et al*. (*p*-value ≤ 0.05; see Table S2 and Figure S1), which demonstrated the diagnostic value of our identified miRNA biomarkers.

In summary, eight of our miRNAs (72.7%, 8/11), i.e., miR-106b-5p, miR-193b-3p, miR-486-5p, miR-129-5p, miR-181b-5p, miR-34a-5p, miR-96-5p, and miR-195-5p, have been reported as significantly dysregulated in ASD samples according to previous studies.

### GO analysis

**Figure 3:**
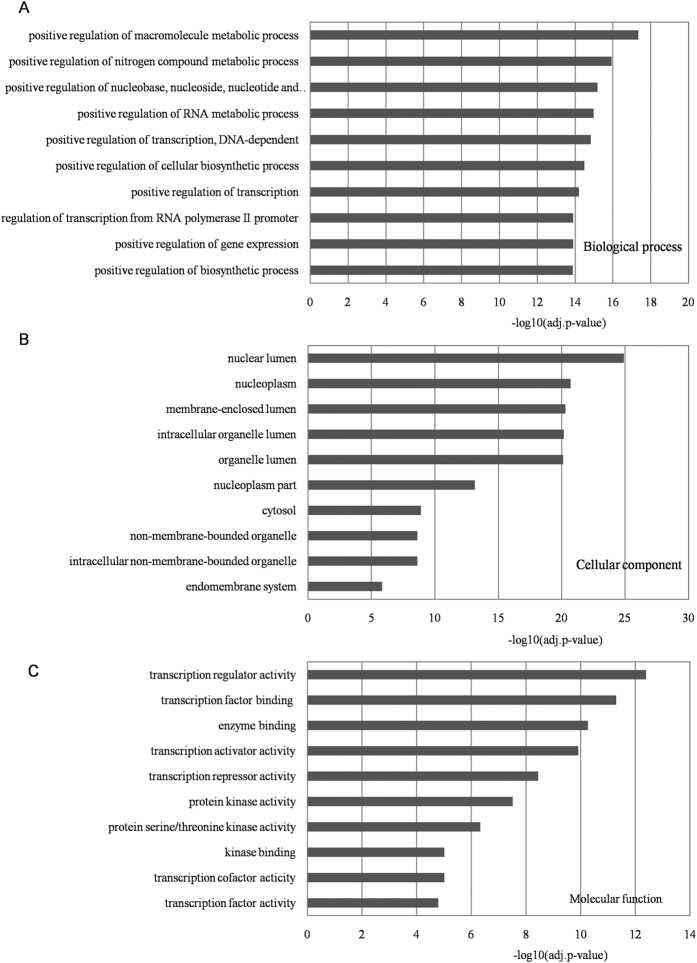
Gene ontology (GO) annotations for the targets of identified miRNA biomarkers. The targets of the identified miRNA biomarkers were extracted from the human miRNA–mRNA network and the online tool DAVID was used to perform GO annotation at three levels: biological process, cellular component, and molecular function, as shown in subfigures (**A–C**), respectively. Each statistical significance value (adj.p-value) was negative log-10 base transformed and the top 10 significantly enriched items at each level are listed.

The online tool DAVID was used for GO analysis at three levels: biological process (BP), cellular component, and molecular function (MF). The results are summarized in Table S3. The top 10 significantly enriched items were selected at each level, as shown in Fig. 3. Most of the genes regulated by the identified biomarker miRNAs mapped well onto autism-associated processes or biological factors. For example, at the BP level, recent studies have demonstrated that the regulation of retinoic acid-related orphan receptor γ (RORγ) is connected with the cholesterol biosynthetic pathway^36^. RORs have also been demonstrated to play key roles in several neural disorders, including autism^37^. At the MF level, if we consider TF activity as an example, evidence indicates that several TFs, such as th1 and th2, are dysregulated in autism groups^38^,^39^. All the results supported the functional importance of the identified autism miRNA biomarkers.

### KEGG pathway analysis

**Figure 4:**
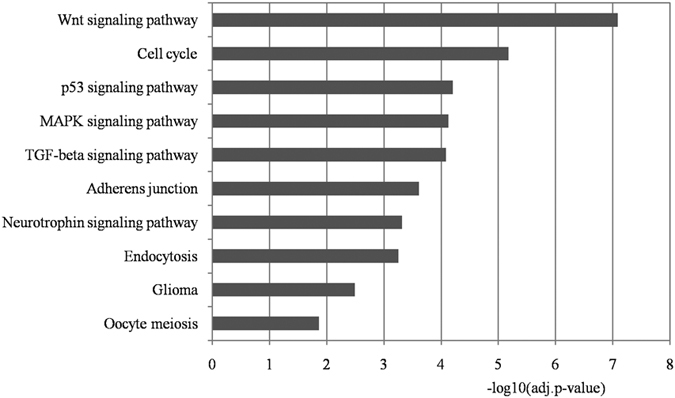
KEGG pathway enrichment analysis for the targets of the identified miRNA biomarkers. Each statistical significance value (adj.p-value) was negative log-10 base transformed. Pathways highly connected with cancers were removed and the top 10 remaining significantly enriched pathways are shown.

To further investigate the functional mechanisms of these 11 miRNAs, we performed pathway enrichment analysis based on their targets. The enriched pathways are listed in Table S4. We removed the pathways with strong connections to cancer. Next, we selected the top 10 pathways with highly significant levels, as shown in Fig. 4. Seven pathways had experimentally validated relationships with autism, including the Wnt signaling pathway, cell cycle, p53 signaling pathway, MAPK signaling pathway, glioma signaling pathway, endocytosis, and neurotrophin signaling pathway^40^,^41^,^42^,^43^. In addition, two pathways (i.e., adherens junction and TGF-beta signaling pathway) were confirmed by computational methods^44^.

For example, the En2 gene, which is one of the targets of miR-193b-3p, was enriched in the Wnt signaling pathway^45^. Evidence indicates that its location, 7q36, is a specific chromosomal region that is strongly associated with autism^46^,^47^. There is also a pvuII polymorphism in En2, which differs clearly between autistic and non-autistic children^48^. An investigation of En2 null mutant mice detected autism-like symptoms, such as repetitive behavior and a communication barrier^49^. The results all support a potential relationship between the Wnt signaling pathway and autism pathogenesis.

The MAPK signaling pathway transmits signals from cell surface receptors to DNA. Using KEGG, it was demonstrated that the dysregulated network built by Ghahramani Seno *et al*. was associated with nervous system development. It is interesting that the network was nucleated by several kinases from the MAPK signaling pathway. These kinases, such as MAPK and AKT, are systematically dysregulated in the development of autism. Thus, their roles in cell signal transmission may contribute to the pathogenesis of autism^40^.

In the p53 signaling pathway, evidence indicates that in the brains of autistic subjects, the Bcl2 gene expression level is significantly decreased whereas the expression of p53 is increased^50^. The p53 signaling pathway is also involved with the cell cycle. In addition, a recent study found that dysregulated gene networks in children affected early brain development, thereby influencing the development of autism^51^.

The other three pathways validated by biological experiments are endocytosis, the neurotrophin signaling pathway, and the glioma signaling pathway. During endocytosis, the SCAMP5 gene is essential when the neuronal activity is high, where it might control the recycling of synaptic vesicles, and thus it could maintain sufficient endocytosis during intense neuronal activity. Thus, the knockdown of SCAMP5 would have negative effects on neuronal functions and lead to synaptic dysfunction^43^. Moreover, brain-derived neurotrophic factor (BDNF) in the neurotrophin signaling pathway plays vital roles in neurodevelopmental and neurodegenerative diseases^52^. Several recent studies have indicated that reduced cerebrospinal fluid levels of BDNF are associated with Rett syndrome^53^. It has been demonstrated that the loss of MeCP2 might contribute to decreases in neurotrophic factors, including BDNF, which have strong relationships with autism. A study of C6 glioma cells found that the risperidone targets on glial cells *in vivo* regulate the secretion of S100B from C6 glioma cells, which may be involved in autism^54^.

### IPA

**Figure 5:**
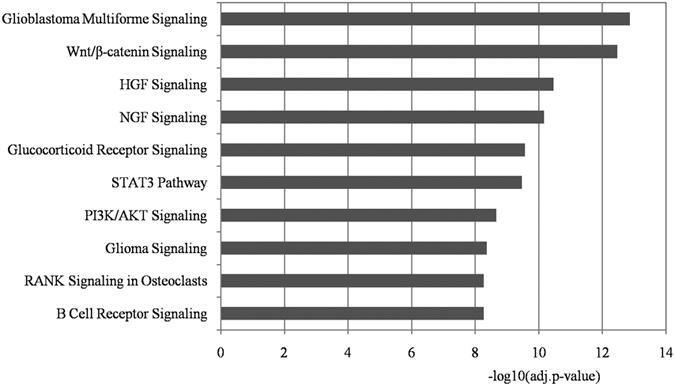
IPA pathway enrichment analysis of the targets of the identified miRNA biomarkers. Each statistical significance value (adj.p-value) was negative log-10 base transformed. Pathways strongly connected with cancers and mouse embryonic stem cell pluripotency were removed. The top 10 remaining significantly enriched pathways are shown.

IPA is a commercial program for understanding biological systems, including pathway analysis. The significantly enriched IPA pathways are listed in Table S5. Pathways related to cancers and mouse embryonic stem cell pluripotency were removed. The top 10 remaining significantly enriched pathways are summarized in Fig. 5.

We also evaluated the relevance of these pathways to autism by searching for publications in PubMed. In total, 8/10 enriched pathways have been reported to have associations with autism, including the nerve growth factor (NGF) signaling pathway, glioblastoma multiforme signaling pathway, hepatocyte growth factor (HGF) signaling pathway, glucocorticoid receptor signaling pathway, STAT3 pathway, glioma signaling pathway, Wnt/β-catenin signaling pathway, and PI3K/AKT signaling pathway. Interestingly, the Wnt/β-catenin and glioma signaling pathway also appeared in the IPA outcome lists, thereby strengthening their relationships with autism. Previous studies have indicated that several differential alternative splicing genes (DAS), such as FXR1, C19orf2, GSN, and SRPK1, are associated with autism. These DAS genes are functional in many signaling pathways, including the NGF signaling pathway^55^.

Omura also identified a relationship between the glioblastoma multiforme signaling pathway and autism^56^. Glioblastoma multiforme is one of the most serious brain tumors and patients with this tumor have often been exposed to a high concentration of asbestos. Asbestos also has relatively strong effects on many other diseases, including autism. This is the only report that has connected autism and glioblastoma multiforme, and thus further experimental validation is needed.

The key gene MET in the HGF signaling pathway, which is regulated by our identified marker miR-186-5p, plays important roles in the pathophysiology of neurodevelopmental disorders, including autism^57^. Campbell *et al*. also reported a strong link between MET in the HGF signaling pathway and autism^58^.

The glucocorticoid receptor signaling pathway is an important pathway *in vivo*. Studies have shown that there is a link between this pathway and relaxin family peptide (RXFP) members^59^. One of the relaxin family peptide members is RXFP-3 and it is a potential drug target for autism treatment^60^.

The remaining three pathways that have been reported as abnormal in autism are the STAT3 pathway, glioma signaling pathway, and PI3K/AKT signaling pathway. Evidence suggests that increased interleukin-6 (IL-6) levels have an important role in influencing brain development. IL-6 induces JAK/STAT3 phosphorylation *in vivo*, which could lead to social and behavior deficits. Inhibiting this process may reduce the risk of autism^61^. Bailey *et al*. found that the PI3K/AKT signaling process driven by sAPP-α was aberrant in autistic patients^62^.

## Discussion

It has been widely reported that miRNAs can be effective biomarkers for neural disorders, such as Alzheimer’s disease and Parkinson’s disease^63^,^64^. However, few studies have focused on detecting miRNA biomarkers for autism and only a small number of approaches have been proposed based on computational methods. In this study, we improved our miRNA biomarker discovery model by adding a novel autism-specific index called AGP, which we then employed to screen key miRNAs for autism diagnosis. We assumed that miRNAs should have at least two vital characteristics as biomarkers: first, they tend to be dysfunctional in disease samples compared with normal controls, and second, but more importantly, they are sufficiently powerful to indicate the state of the system in health and disease. We inferred 66 functional autism-associated miRNAs using gene expression data based on miRNA–mRNA network analysis and we then selected 30 outliers by manually searching for citations in PubMed. The selected miRNAs satisfied the characteristics of miRNA biomarkers well because they all had reported associations with autism progression, as well as having strong independent regulatory power (high NOD values), and they could regulate more TF genes (high TFP values) according to our predictive model. As mentioned in our previous study^15^, disorders in unique regulatory sites are more likely to cause system-level changes, while dysfunctions of TFs may also contribute to disease pathogenesis because they are important regulators that are always located at the center of multiple biological activities^14^.

To strengthen the bioinformatics model and make it more specific to autism miRNA biomarker discovery, we defined the parameter AGP to guide biomarker discovery. Based on the hypothesis that miRNAs with regulatory effects on autism-associated genes would have stronger relationships with autism, we screened a total of 11 miRNAs with significantly high AGP values from the selected miRNA dataset, which were identified as candidate biomarkers for autism diagnosis. Three of these miRNAs have been reported as potential biomarkers for autism^5^, and the regulated genes, including MeCP2, MET, and EN2, were differentially expressed in samples of autism patients validated by biological experiments^65^,^66^,^67^. Moreover, seven miRNAs were also shown to be significantly dysregulated in brain samples from autism patients, according to a study by Wu *et al*.^10^. We used DAVID and IPA as functional enrichment tools for GO and pathway analysis. The results showed that the genes targeted by the identified miRNA biomarkers were enriched in pathways associated strongly with neural development, such as the Wnt signaling pathway, p53 signaling pathway, and PI3K/AKT signaling pathway^42^,^50^,^62^. For example, previous studies have implicated Wnt signaling in vertebrate neural patterning^68^, and the functional gene EN2, which is located in a chromosomal region that is strongly associated with autism development, is a direct target of this pathway^45^. Several DAS genes, such as FXR1, C19orf2, and GSN, play essential roles in the NGF signaling pathway, and their functional dysregulation may contribute to the initiation of autism in children^55^. In addition to the biological functions of the genes in this pathway, the occurrence of autism is often due to abnormal changes in pathways related to other neural diseases. For example, glioblastoma multiforme signaling is potentially related to autism because exposure to a high concentration of asbestos (which causes glioblastoma multiforme) may promote the initiation of autism^56^. Overall, the pathway enrichment analyses showed that the targets of the identified miRNA biomarkers are strongly related to autism-associated processes, thereby supporting the predictive power of our proposed model. In this study, we proposed a systematic bioinformatics method for identifying autism miRNA biomarkers, which can provide insights into the pathogenesis of autism at the post-transcriptional level. Further clinical validation will be needed in the future in order to translate our findings from theoretical insights into clinical practice.

## Conclusion

In this study, we identified 11 miRNAs that could be used as candidate biomarkers for autism diagnosis based on a knowledge-guided bioinformatics model. Literature searches and functional enrichment analyses confirmed our predictions.

## Additional Information

**How to cite this article**: Shen, L. *et al*. Knowledge-Guided Bioinformatics Model for Identifying Autism Spectrum Disorder Diagnostic MicroRNA Biomarkers. *Sci. Rep.* **6**, 39663; doi: 10.1038/srep39663 (2016).

**Publisher's note:** Springer Nature remains neutral with regard to jurisdictional claims in published maps and institutional affiliations.

## Supplementary Material

Supplementary Information

## Figures and Tables

**Table 1:** Candidate miRNA biomarkers identified by our proposed model.

No.	miRNA ID	NOD (p-value)	TFP (p-value)	AGP (p-value)
1	miR-193b-3p	1 (6.01E-30)	0.308 (8.30E-34)	0.0714 (1.86E-09)
2	miR-186-5p	1 (6.01E-30)	0.3 (4.85E-33)	0.0566 (6.15E-08)
3	miR-486-5p	1 (6.01E-30)	0.2 (2.13E-12)	0.056 (1.66E-07)
4	miR-129-5p	1 (6.01E-30)	0.286 (2.63E-31)	0.0556 (3.15E-07)
5	miR-106b-5p	2 (8.82E-79)	0.157 (3.63E-03)	0.0508 (3.79E-06)
6	miR-181b-5p	1 (6.01E-30)	0.167 (5.07E-05)	0.05 (9.19E-06)
7	miR-34a-5p	3 (1.97E-108)	0.28 (7.02E-31)	0.0444 (2.78E-03)
8	miR-96-5p	1 (6.01E-30)	0.267 (4.88E-29)	0.0442 (3.47E-03)
9	miR-211-5p	1 (6.01E-30)	0.2 (2.13E-12)	0.0436 (6.99E-03)
10	miR-205-5p	3 (1.97E-108)	0.25 (6.55E-26)	0.0429 (1.22E-02)
11	miR-195-5p	1 (6.01E-30)	0.226 (5.59E-20)	0.0417 (4.42E-02)

Note: The miRNAs are shown in descending order according to their AGP values.
