# Supplementary material for: Knowledge-Guided Bioinformatics Model for Identifying Autism Spectrum Disorder Diagnostic MicroRNA Biomarkers
Source: Sci Rep. 2016 Dec 21;6:39663. doi: 10.1038/srep39663 (PMC5175196; doi:10.1038/srep39663)
Supplement: Supplementary Information [file srep39663-s1.pdf]

Knowledge-Guided Bioinformatics Model for Identifying Autism Spectrum Disorder Diagnostic MicroRNA Biomarkers

Li Shen[1, 2, †], Yuxin Lin[1, †], Zhandong Sun[1], Xuye Yuan[1], Luonan Chen[3], Bairong Shen[1, *]

[1] Center for Systems Biology, Soochow University, Suzhou, 215006, China

[2] Institute of Biological Sciences and Biotechnology, Donghua University, Shanghai, 201620, China

[3] Key laboratory of Systems Biology, Shanghai Institute of Biological Sciences, Chinese Academy of Sciences, Shanghai, 200031, China

[*] To whom correspondence should be addressed: bairong.shen@suda.edu.cn.

[†] These authors contributed equally to this work.

Supplementary Information

Table S1

Functional miRNAs associated with autism identified by the POMA model.

No.	miRNA ID	NOD	TFP	Reported as autism-associated miRNAs (TRUE/FALSE)	Evidence (PMID)
1	miR-326	2	0.1562	TRUE	20374639
2	miR-193b-3p	1	0.3077	TRUE	18563458
3	miR-342-3p	1	0.2222	TRUE	20374639, 22981949
4	miR-27a-3p	2	0.2917	TRUE	18563458, 27105825
5	miR-29b-3p	1	0.25	TRUE	22981949
6	miR-218-5p	1	0.1765	TRUE	27105825
7	miR-34a-5p	3	0.28	TRUE	23451085
8	let-7f-5p	1	0.2353	TRUE	26061495
9	miR-186-5p	1	0.3	TRUE	20374639
10	miR-432-5p	1	0.2	TRUE	18563458
11	miR-200b-3p	1	0.1818	TRUE	24667286, 23451085
12	miR-195-5p	1	0.2258	TRUE	20374639, 25126405, 22539927, 23451085, 20374639
13	miR-9-5p	2	0.25	TRUE	22981949
14	miR-486-5p	1	0.2	TRUE	20868653
15	miR-211-5p	1	0.2	TRUE	20374639, 18805830

16	miR-129-5p	1	0.2857	TRUE	24423034, 18563458
17	miR-144-3p	2	0.2941	TRUE	26273428
18	miR-142-3p	1	0.2188	TRUE	26273428
19	miR-184	1	0.3333	TRUE	22981949
20	miR-106b-5p	2	0.157	TRUE	25126405, 18563458, 20374639
21	miR-196a-5p	2	0.4286	TRUE	20868653
22	miR-191-5p	1	0.1667	TRUE	27105825
23	miR-574-5p	1	0.2	TRUE	26061495
24	miR-29a-3p	1	0.1935	TRUE	22981949
25	miR-96-5p	1	0.2667	TRUE	24423034
26	miR-124-3p	6	0.1579	TRUE	23451085
27	miR-155-5p	10	0.2553	TRUE	27014996
28	miR-146a-5p	2	0.2308	TRUE	27014996, 26753090, 19360674
29	miR-205-5p	3	0.25	TRUE	20374639
30	miR-181b-5p	1	0.1667	TRUE	25126405, 22539927
31	miR-579	2	0.1739	FALSE	NA
32	miR-29c-3p	1	0.2	FALSE	NA
33	miR-204-5p	1	0.1905	FALSE	NA
34	miR-503-5p	1	0.1667	FALSE	NA
35	miR-448	3	0.1728	FALSE	NA
36	miR-421	2	0.1667	FALSE	NA
37	miR-661	2	0.2727	FALSE	NA
38	miR-766-3p	3	0.1538	FALSE	NA
39	miR-182-5p	1	0.1818	FALSE	NA
40	miR-637	5	0.1818	FALSE	NA

41	miR-648	2	0.2	FALSE	NA
42	miR-653	1	0.2667	FALSE	NA
43	miR-614	1	0.1667	FALSE	NA
44	miR-627	2	0.2857	FALSE	NA
45	miR-504	2	0.2222	FALSE	NA
46	miR-582-3p	2	0.1538	FALSE	NA
47	miR-885-5p	1	0.2	FALSE	NA
48	miR-203a	2	0.1765	FALSE	NA
49	miR-934	1	0.2857	FALSE	NA
50	miR-488-3p	1	0.4	FALSE	NA
51	miR-378a-3p	1	0.1786	FALSE	NA
52	miR-150-5p	1	0.1667	FALSE	NA
53	miR-200c-3p	3	0.1724	FALSE	NA
54	miR-222-3p	1	0.2105	FALSE	NA
55	miR-767-5p	1	0.2	FALSE	NA
56	miR-922	4	0.2	FALSE	NA
57	miR-590-5p	1	0.1875	FALSE	NA
58	miR-33a-5p	1	0.2353	FALSE	NA
59	miR-335-5p	5	0.1833	FALSE	NA
60	miR-556-3p	1	0.3333	FALSE	NA
61	miR-589-3p	1	0.2	FALSE	NA
62	miR-324-3p	1	0.1538	FALSE	NA
63	miR-22-3p	5	0.225	FALSE	NA
64	miR-191-3p	1	1	FALSE	NA
65	let-7b-5p	1	0.2	FALSE	NA
66	miR-203a-3p	2	0.1765	FALSE	NA

Note: Here, "NA" is the abbreviation of "Not Available"

Table S2

Identified biomarker miRNAs with different expression levels in the study by Wu et al[1].

No.	miRNA ID	Sample group	Number of samples (ASD/Control)	Reported p-value	Method
1	miR-96-5p	cortex	95 (47/48)	0.043	LME model
2	miR-486-5p	cortex	95 (47/48)	0.05	LME model
3	miR-34a-5p	cortex age ⩽ 30 yrs	47 (22/25)	0.041	LME model
4	miR-193b-3p	temporal cortex	48 (25/23)	0.034	LME model
5	miR-181b-5p	cerebellum	47 (21/26)	0.0035	Linear model
6	miR-195-5p	cerebellum	47 (21/26)	6.05E-05	Linear model
7	miR-129-5p	cerebellum	47 (21/26)	0.0043	Linear model

Note: LME is the abbreviation of linear mixed-effects model[1].

Reference:

1. Wu, Y. E., Parikshak, N. N., Belgard, T. G. & Geschwind, D. H. Genome-wide, integrative analysis implicates microRNA dysregulation in autism spectrum disorder. Nature neuroscience 19, 1463-1476, doi:10.1038/nn.4373 (2016).

Table S3:

Significant GO terms (adjusted p-value<0.05) enriched for the targets of the identified miRNA biomarkers.

Table S3-1

Significant Biological Process terms (adjusted p-value<0.05) enriched by targets of identified miRNA biomarkers.

Term	Count	%	p-value	Adjusted p-value	Genes

GO:0010604~positive regulation of macromolecule metabolic process	188	10.30	1.08E-21	4.44E-18	MEF2C, CDX2, THRA, WFS1, MORF4L2, HOXD13, CASK, FOXO1, TBP, FOXO3, HOXD10, CBFB, CITED2, IL11, EPC1, WNT1, ZFP91, APP, GATA6, WIBG, FOXF2, RARB, ITCH, CUL1, MYST4, ADAM9, SATB2, STRN3, MTA2, ARID1A, CD40, IRS1, DDIT3, TNKS2, HHEX, PSMA1, MAPK1, EP300, HIF1A, HNF4A, MTF1, PIAS3, VEGFA, PDGFRA, MAPK9, TGFB1I1, LRRK2, CAV1, HOXA13, SOX2, BMPR2, SOX4, NFKBIA, SRF, PSMB5, LIF, ECE1, TAF9, DYRK2, UBE2D1, TCF4, RUNX1, ASF1A, TCF3, PLAGL2, TAF2, KLF6, DVL3, BMP2, MLL, IKZF2, EPAS1, KLF12, MAP2K1, SMAD7, TGFBR1, SMAD5, FOXA1, NR4A2, CDC23, NR4A1, SMAD2, NR4A3, DDX5, FURIN, HDAC4, PSMC6, P2RX7, RNF6, NOTCH1, RNF4, HDAC1, ETS1, DYRK1B, SMARCC1, ADRA1B, SMURF1, BMP7, UBE2E1, BMP6, E2F1, PPARA, E2F3, RSF1, MITF, PRKAG2, PPARG, SPI1, GJA1, ZEB1, PAWR, GLI3, CCNE1, ZNF148, PSMD5, MKL2, NR2F2, FAM129A, USP16, MKL1, FOSL1, MYC, SERTAD2, TBL1XR1, EGR2, FOXJ2, SOX11, ESR1, TP53, LEF1, RB1, CDK4, ARHGEF11, UBE2N, ADRB2, EYA1, CCND1, NCOA3, CCND3, EREG, BPTF, PSME1, CCND2, NCOA4, NCOA6, UBC, MGEA5, ZFPM2, EIF5A2, MAPRE3, CLOCK, TBX19, MED1, ING5, FKBP1A, TCF7L1, IGF1R, NPAS2, SQSTM1, RB1CC1, BCL11B, BCL2, NFAT5, POU2F1, POU3F2, THBS1, ING1, NFATC3, APC, CEBPB, VHL, PSRC1, CREB5, AFF1, STAT3, ATXN7L3, TP73, ATXN1, PKNOX1, ILF2, PSMD11, IRF1, JAK2, TBL1X, RBM14, NFIB, F2R, BMPR1A

GO:0051173~positive regulation of nitrogen compound metabolic process	151	8.27	2.83E-20	1.17E-16	MEF2C, CDX2, THRA, MORF4L2, CASK, HOXD13, FOXO1, TBP, FOXO3, HOXD10, CBFB, CITED2, IL11, EPC1, WNT1, APP, GATA6, FOXF2, RARB, MYST4, SATB2, STRN3, MTA2, ARID1A, CD40, DDIT3, TNKS2, PTHLH, HHEX, MAPK1, EP300, HIF1A, MTF1, HNF4A, VEGFA, PDGFRA, TGFB1I1, HOXA13, SOX2, NFKBIA, SOX4, ABCA1, SRF, LIF, TAF9, TCF4, RUNX1, ASF1A, TCF3, PLAGL2, TAF2, KLF6, DVL3, BMP2, MLL, IKZF2, EPAS1, KLF12, MAP2K1, TGFBR1, SMAD5, FOXA1, NR4A2, NR4A1, SMAD2, NR4A3, DDX5, HDAC4, P2RX4, RNF6, NOTCH1, RNF4, HDAC1, ETS1, DYRK1B, SMARCC1, BMP7, BMP6, HSP90AB1, E2F1, PPARA, E2F3, RSF1, MITF, PPARG, SPI1, ZEB1, GLI3, CCNE1, ZNF148, GUCY1A3, MKL2, NR2F2, USP16, MKL1, FOSL1, MYC, SERTAD2, TBL1XR1, EGR2, FOXJ2, SOX11, TP53, LEF1, RB1, ARHGEF11, UBE2N, EYA1, ADRB2, NCOA3, BPTF, EREG, NCOA4, NCOA6, UBC, MGEA5, PEBP1, ZFPM2, MAPRE3, CLOCK, TBX19, MED1, ING5, HPRT1, TCF7L1, IGF1R, NPAS2, SQSTM1, BCL11B, NFAT5, POU2F1, POU3F2, ING1, NFATC3, CEBPB, VHL, PSRC1, CREB5, AFF1, STAT3, ATXN7L3, TP73, ATXN1, PKNOX1, ILF2, IRF1, JAK2, TBL1X, RBM14, NFIB, F2R

GO:0045935~positive regulation of nucleobase, nucleoside, nucleotide and nucleic acid metabolic process	146	8.00	1.58E-19	6.53E-16	MEF2C, CDX2, THRA, MORF4L2, CASK, HOXD13, FOXO1, TBP, FOXO3, HOXD10, CBFB, CITED2, IL11, EPC1, WNT1, APP, GATA6, FOXF2, RARB, MYST4, SATB2, STRN3, MTA2, ARID1A, CD40, DDIT3, TNKS2, PTHLH, HHEX, MAPK1, EP300, HIF1A, MTF1, HNF4A, VEGFA, PDGFRA, TGFB1I1, HOXA13, SOX2, NFKBIA, SOX4, ABCA1, SRF, LIF, TAF9, TCF4, RUNX1, ASF1A, TCF3, PLAGL2, TAF2, KLF6, DVL3, BMP2, MLL, IKZF2, EPAS1, KLF12, MAP2K1, TGFBR1, SMAD5, FOXA1, NR4A2, NR4A1, SMAD2, NR4A3, DDX5, HDAC4, RNF6, NOTCH1, RNF4, HDAC1, ETS1, DYRK1B, SMARCC1, BMP7, BMP6, E2F1, PPARA, E2F3, RSF1, MITF, PPARG, SPI1, ZEB1, GLI3, CCNE1, ZNF148, GUCY1A3, MKL2, NR2F2, USP16, MKL1, FOSL1, MYC, SERTAD2, TBL1XR1, EGR2, FOXJ2, SOX11, TP53, LEF1, RB1, ARHGEF11, UBE2N, EYA1, ADRB2, NCOA3, BPTF, EREG, NCOA4, NCOA6, UBC, MGEA5, ZFPM2, MAPRE3, TBX19, CLOCK, MED1, ING5, TCF7L1, IGF1R, NPAS2, SQSTM1, BCL11B, NFAT5, POU2F1, POU3F2, ING1, NFATC3, CEBPB, VHL, PSRC1, CREB5, AFF1, STAT3, ATXN7L3, TP73, ATXN1, PKNOX1, ILF2, IRF1, TBL1X, RBM14, NFIB, F2R

GO:0051254~positive regulation of RNA metabolic process	122	6.68	2.54E-19	1.05E-15	MEF2C, CDX2, THRA, MORF4L2, CASK, HOXD13, FOXO1, FOXO3, CBFB, HOXD10, CITED2, IL11, EPC1, WNT1, APP, GATA6, FOXF2, RARB, SATB2, MTA2, STRN3, ARID1A, HHEX, EP300, HIF1A, MTF1, HNF4A, VEGFA, TGFB1I1, HOXA13, SOX2, NFKBIA, SOX4, SRF, LIF, TAF9, TCF4, RUNX1, ASF1A, TCF3, PLAGL2, TAF2, KLF6, DVL3, BMP2, MLL, IKZF2, EPAS1, MAP2K1, KLF12, SMAD5, FOXA1, NR4A2, NR4A1, SMAD2, NR4A3, HDAC4, RNF6, NOTCH1, RNF4, HDAC1, ETS1, SMARCC1, DYRK1B, BMP7, BMP6, E2F1, PPARA, E2F3, MITF, PPARG, SPI1, ZEB1, CCNE1, ZNF148, MKL2, NR2F2, USP16, MYC, FOSL1, SERTAD2, TBL1XR1, EGR2, FOXJ2, SOX11, TP53, LEF1, RB1, ARHGEF11, ADRB2, BPTF, NCOA3, NCOA4, NCOA6, ZFPM2, MAPRE3, TBX19, CLOCK, MED1, TCF7L1, NPAS2, SQSTM1, BCL11B, POU2F1, NFAT5, POU3F2, NFATC3, CEBPB, PSRC1, CREB5, AFF1, STAT3, TP73, ATXN7L3, ATXN1, PKNOX1, ILF2, IRF1, TBL1X, RBM14, NFIB, F2R

GO:0045893~positive regulation of transcription, DNA-dependent	121	6.63	3.59E-19	1.48E-15	MEF2C, CDX2, THRA, MORF4L2, CASK, HOXD13, FOXO1, FOXO3, CBFB, HOXD10, CITED2, IL11, EPC1, WNT1, APP, GATA6, FOXF2, RARB, SATB2, MTA2, STRN3, ARID1A, HHEX, EP300, HIF1A, MTF1, HNF4A, VEGFA, TGFB1I1, HOXA13, SOX2, NFKBIA, SOX4, SRF, LIF, TAF9, TCF4, RUNX1, ASF1A, TCF3, PLAGL2, TAF2, KLF6, DVL3, BMP2, MLL, IKZF2, EPAS1, KLF12, SMAD5, FOXA1, NR4A2, NR4A1, SMAD2, NR4A3, HDAC4, RNF6, NOTCH1, RNF4, HDAC1, ETS1, SMARCC1, DYRK1B, BMP7, BMP6, E2F1, PPARA, E2F3, MITF, PPARG, SPI1, ZEB1, CCNE1, ZNF148, MKL2, NR2F2, USP16, MYC, FOSL1, SERTAD2, TBL1XR1, EGR2, FOXJ2, SOX11, TP53, LEF1, RB1, ARHGEF11, ADRB2, BPTF, NCOA3, NCOA4, NCOA6, ZFPM2, MAPRE3, TBX19, CLOCK, MED1, TCF7L1, NPAS2, SQSTM1, BCL11B, POU2F1, NFAT5, POU3F2, NFATC3, CEBPB, PSRC1, CREB5, AFF1, STAT3, TP73, ATXN7L3, ATXN1, PKNOX1, ILF2, IRF1, TBL1X, RBM14, NFIB, F2R

GO:0031328~positive regulation of cellular biosynthetic process	154	8.44	7.64E-19	3.15E-15	MEF2C, CDX2, THRA, MORF4L2, CASK, HOXD13, FOXO1, TBP, FOXO3, HOXD10, CBFB, CITED2, IL11, EPC1, WNT1, APP, GATA6, WIBG, FOXF2, RARB, MYST4, SATB2, STRN3, MTA2, ARID1A, IRS1, DDIT3, TNKS2, PTHLH, HHEX, MAPK1, EP300, HIF1A, MTF1, HNF4A, VEGFA, PDGFRA, TGFB1I1, HOXA13, SOX2, NFKBIA, SOX4, ABCA1, SRF, LIF, NPM1, TAF9, DYRK2, TCF4, RUNX1, ASF1A, TCF3, PLAGL2, TAF2, KLF6, DVL3, BMP2, MLL, IKZF2, EPAS1, KLF12, TGFBR1, SMAD5, FOXA1, NR4A2, NR4A1, SMAD2, NR4A3, DDX5, HDAC4, P2RX4, PLA2G4A, RNF6, NOTCH1, RNF4, HDAC1, ETS1, DYRK1B, SMARCC1, BMP7, BMP6, HSP90AB1, E2F1, PPARA, E2F3, RSF1, MITF, PPARG, SPI1, ZEB1, PAWR, GLI3, CCNE1, ZNF148, GUCY1A3, MKL2, NR2F2, FAM129A, USP16, MKL1, FOSL1, MYC, SERTAD2, TBL1XR1, EGR2, FOXJ2, SOX11, TP53, LEF1, RB1, CDK4, ARHGEF11, ADRB2, NCOA3, BPTF, EREG, NCOA4, NCOA6, UBC, ZFPM2, EIF5A2, MAPRE3, CLOCK, TBX19, MED1, ING5, TCF7L1, IGF1R, NPAS2, SQSTM1, BCL11B, NFAT5, POU2F1, POU3F2, THBS1, ING1, NFATC3, CEBPB, VHL, PSRC1, CREB5, AFF1, STAT3, ATXN7L3, TP73, ATXN1, PKNOX1, ILF2, IRF1, JAK2, TBL1X, RBM14, NFIB, F2R

GO:0045941~positive regulation of transcription	134	7.34	1.49E-18	6.12E-15	MEF2C, CDX2, THRA, MORF4L2, CASK, HOXD13, FOXO1, TBP, FOXO3, HOXD10, CBFB, CITED2, IL11, EPC1, WNT1, APP, GATA6, FOXF2, RARB, MYST4, SATB2, STRN3, MTA2, ARID1A, DDIT3, HHEX, MAPK1, EP300, HIF1A, MTF1, HNF4A, VEGFA, TGFB1I1, HOXA13, SOX2, NFKBIA, SOX4, SRF, LIF, TAF9, TCF4, RUNX1, ASF1A, TCF3, PLAGL2, TAF2, KLF6, DVL3, BMP2, MLL, IKZF2, EPAS1, KLF12, TGFBR1, SMAD5, FOXA1, NR4A2, NR4A1, SMAD2, NR4A3, DDX5, HDAC4, RNF6, NOTCH1, RNF4, HDAC1, ETS1, DYRK1B, SMARCC1, BMP7, BMP6, E2F1, PPARA, E2F3, RSF1, MITF, PPARG, SPI1, ZEB1, GLI3, CCNE1, ZNF148, MKL2, NR2F2, USP16, MKL1, MYC, FOSL1, SERTAD2, TBL1XR1, EGR2, FOXJ2, SOX11, TP53, LEF1, RB1, ARHGEF11, ADRB2, NCOA3, BPTF, NCOA4, NCOA6, UBC, ZFPM2, MAPRE3, TBX19, CLOCK, MED1, ING5, TCF7L1, NPAS2, SQSTM1, BCL11B, NFAT5, POU2F1, POU3F2, ING1, NFATC3, CEBPB, VHL, PSRC1, CREB5, AFF1, STAT3, TP73, ATXN7L3, ATXN1, PKNOX1, ILF2, IRF1, TBL1X, RBM14, NFIB, F2R

GO:0006357~regulation of transcription from RNA polymerase II promoter	159	8.71	3.01E-18	1.24E-14	MEF2C, CDX2, THRA, MORF4L2, HIRA, CASK, HOXD13, FOXO1, FOXO3, TCEAL1, HOXD10, CBFB, CITED2, IL11, EPC1, APP, SMARCD2, GATA6, RARB, SATB2, STRN3, MTA2, YY1, RBL1, ZHX2, MECP2, MED11, HNF4G, TAF6L, PKIA, HHEX, EP300, HIF1A, ZNF238, MTF1, HNF4A, VEGFA, SMARCA5, TFAP2A, SUPT6H, HOXA13, LITAF, SOX2, NFKBIA, NEDD8, SRF, NR2C1, LIF, HEXIM1, TAF9, TCF4, RUNX1, TCF3, PLAGL2, TAF2, DNMT3A, KLF6, BMP2, MLL, IKZF2, EPAS1, KLF12, KLF9, MAP2K1, SMAD7, SMAD5, FOXA1, KLF11, NR4A2, RYBP, NR4A1, SKI, SMAD2, NR4A3, UBP1, FOXP1, CDKN1C, HDAC4, HOXB4, ATF5, NOTCH1, RNF4, HDAC1, ETS1, SMARCC1, RNF2, HDAC9, BMP7, TCF12, NCOR2, BMP6, E2F1, PPARA, PPARD, ELF2, MITF, NR6A1, PPARG, FOXK2, SPI1, ZEB1, PAWR, BLZF1, ZNF148, HEY2, RHOA, MKL2, NR2F2, FOSL1, MYC, ZNF281, TBL1XR1, EGR2, SOX11, SOX12, TP53, LEF1, RB1, HLTF, RBBP7, MYCN, TARBP1, TARBP2, ADRB2, BRWD1, BPTF, MED8, NCOA6, C19ORF2, ZFPM2, MDM4, CLOCK, TBX19, MED1, TCF7L1, NPAS2, SQSTM1, BCL11B, NPAT, NFAT5, POU2F1, CHD1, BCL6, POU3F2, NFATC3, ZBTB7A, CEBPB, JARID2, VHL, SIRT1, STAT3, ATXN1, PKNOX1, DR1, IRF1, TBL1X, RBM14, CRK, NFIB

GO:0010628~positive regulation of gene expression	136	7.45	3.06E-18	1.26E-14	MEF2C, CDX2, THRA, MORF4L2, CASK, HOXD13, FOXO1, TBP, FOXO3, HOXD10, CBFB, CITED2, IL11, EPC1, WNT1, APP, GATA6, FOXF2, RARB, MYST4, SATB2, STRN3, MTA2, ARID1A, DDIT3, HHEX, MAPK1, EP300, HIF1A, MTF1, HNF4A, VEGFA, MAPK9, TGFB1I1, HOXA13, SOX2, NFKBIA, SOX4, SRF, LIF, TAF9, TCF4, RUNX1, ASF1A, TCF3, PLAGL2, TAF2, KLF6, DVL3, BMP2, MLL, IKZF2, EPAS1, KLF12, TGFBR1, SMAD5, FOXA1, NR4A2, NR4A1, SMAD2, NR4A3, DDX5, HDAC4, RNF6, NOTCH1, RNF4, HDAC1, ETS1, DYRK1B, SMARCC1, BMP7, BMP6, E2F1, PPARA, E2F3, RSF1, MITF, PPARG, SPI1, ZEB1, GLI3, CCNE1, ZNF148, MKL2, NR2F2, USP16, MKL1, MYC, FOSL1, SERTAD2, TBL1XR1, EGR2, FOXJ2, SOX11, ESR1, TP53, LEF1, RB1, ARHGEF11, ADRB2, NCOA3, BPTF, NCOA4, NCOA6, UBC, ZFPM2, MAPRE3, TBX19, CLOCK, MED1, ING5, TCF7L1, NPAS2, SQSTM1, BCL11B, NFAT5, POU2F1, POU3F2, ING1, NFATC3, CEBPB, VHL, PSRC1, CREB5, AFF1, STAT3, TP73, ATXN7L3, ATXN1, PKNOX1, ILF2, IRF1, TBL1X, RBM14, NFIB, F2R

GO:0009891~positive regulation of biosynthetic process	154	8.44	3.13E-18	1.29E-14	MEF2C, CDX2, THRA, MORF4L2, CASK, HOXD13, FOXO1, TBP, FOXO3, HOXD10, CBFB, CITED2, IL11, EPC1, WNT1, APP, GATA6, WIBG, FOXF2, RARB, MYST4, SATB2, STRN3, MTA2, ARID1A, IRS1, DDIT3, TNKS2, PTHLH, HHEX, MAPK1, EP300, HIF1A, MTF1, HNF4A, VEGFA, PDGFRA, TGFB1I1, HOXA13, SOX2, NFKBIA, SOX4, ABCA1, SRF, LIF, NPM1, TAF9, DYRK2, TCF4, RUNX1, ASF1A, TCF3, PLAGL2, TAF2, KLF6, DVL3, BMP2, MLL, IKZF2, EPAS1, KLF12, TGFBR1, SMAD5, FOXA1, NR4A2, NR4A1, SMAD2, NR4A3, DDX5, HDAC4, P2RX4, PLA2G4A, RNF6, NOTCH1, RNF4, HDAC1, ETS1, DYRK1B, SMARCC1, BMP7, BMP6, HSP90AB1, E2F1, PPARA, E2F3, RSF1, MITF, PPARG, SPI1, ZEB1, PAWR, GLI3, CCNE1, ZNF148, GUCY1A3, MKL2, NR2F2, FAM129A, USP16, MKL1, FOSL1, MYC, SERTAD2, TBL1XR1, EGR2, FOXJ2, SOX11, TP53, LEF1, RB1, CDK4, ARHGEF11, ADRB2, NCOA3, BPTF, EREG, NCOA4, NCOA6, UBC, ZFPM2, EIF5A2, MAPRE3, CLOCK, TBX19, MED1, ING5, TCF7L1, IGF1R, NPAS2, SQSTM1, BCL11B, NFAT5, POU2F1, POU3F2, THBS1, ING1, NFATC3, CEBPB, VHL, PSRC1, CREB5, AFF1, STAT3, ATXN7L3, TP73, ATXN1, PKNOX1, ILF2, IRF1, JAK2, TBL1X, RBM14, NFIB, F2R

GO:0045449~regulation of transcription	413	22.63	7.56E-18	3.12E-14	MEF2C, HOXD13, MXI1, HOXD10, CITED2, EPC1, APP, MED28, FOXF2, PHTF2, TBPL1, RCOR3, ZNF646, RCOR1, ZHX2, MECP2, EOMES, ZNF791, MED11, HNF4G, MAPK1, ZNF236, ZNF238, HNF4A, IFNB1, ZZZ3, CRTC3, CRTC2, NFKBIA, ZNF618, ZNF512, HEXIM1, ZNF124, TCF4, TCF3, ASXL2, DNMT3A, DVL3, MAP2K1, KLF12, TGFBR1, ZNF121, KLF11, L3MBTL, SFMBT1, ZNF629, TRPS1, TGFBR3, ELF2, TSG101, ZNFX1, EZH1, ZNF530, SPI1, DAXX, CCNE1, ZNF148, HSF2, USP16, MYB, MYC, SERTAD2, ZNF281, BRF2, TP53, TLE4, ZNF335, ZNF140, MYCN, ARHGEF11, SS18, BRWD1, EREG, C19ORF2, ZNF551, CLOCK, CREM, ZNF655, ZNF175, MYCBP2, PPP2CA, NPAT, CSDE1, ZNF562, SSRP1, ZNF264, ZNF565, PHF10, ELAVL2, PHF12, TP73, RPS6KA5, RLF, MEF2D, PKNOX1, RPS6KA4, ZBTB5, DNM2, TBP, TCEAL1, CBFB, IL11, CNOT4, CDKN2A, SMARCD2, MIER2, EED, ZNF594, RBL2, MTA2, RBL1, RELB, ARID1A, PKIA, DDIT3, ZNF3, HHEX, ZNF439, EP300, HIF1A, SMARCA5, TFAP2A, NFE2L1, EDA, ZNF586, CAMTA1, LITAF, NR2C1, ZNF695, ZNF597, TAF9, ASF1A, TAF2, EPAS1, TAF5, FOXA1, NR4A2, JRKL, NR4A1, NR4A3, WHSC2, CDKN1C, HDAC4, ATF5, HDAC1, SMARCC1, DYRK1B, PNRC1, ZNF318, HDAC9, DNAJB6, ZNF410, E2F1, E2F3, RSF1, ARID4A, E2F5, GPBP1, DEDD, ARID4B, PRDX3, PAWR, ZNF207, TMF1, MAX, PRMT5, RTF1, HEY2, RHOA, FOSL1, PPP2R1A, TBL1XR1, ZFX, ESR1, CDK8, RB1, HLTF, HMGA2, CCNL2, PURA, TARBP1, HOXC10, TARBP2, ADRB2, HOXC11, NCOA3, NCOA4, NCOA6, UBC, ZFPM2, JMJD1C, CARM1, MAPRE3, BTAF1, NR3C1, CHD9, HIC2, TFAM, TSC22D3, TSPYL2, TSC22D2, TSC22D4,

CHD1, CHD5, NFATC3, CEBPB, VHL, CREB5, JAK2, DENND4A, TBL1X, CDX2, HIRA, RAB1A, ZFP91, CRY2, RARB, BCL7A, STRN3, YY1, PIM1, TAF6L, RFC1, PRDM4, MTF1, PIAS3, PARP14, VEGFA, PRDM2, TGFB1I1, SUPT6H, SUV420H1, HOXA13, GPBP1L1, LIF, TCF20, KRAS, HOXA10, PLAGL2, PLAG1, IKZF4, KLF6, BMP2, MLL, IKZF2, KLF9, SMAD7, SMAD6, SMAD5, ZBTB41, RYBP, SKI, SMAD2, CELSR2, EN2, DDX5, UBP1, NOTCH2, NOTCH1, RNF6, RNF4, ETS1, DMTF1, RNF2, BMP7, TCF12, NCOR2, BMP6, KLF3, CCNT2, COPS2, PPARA, PPARD, ZBTB34, PPARG, MITF, ZEB2, ZEB1, GLI3, BLZF1, MBTD1, NR2F2, CTBP2, SNAPC2, SNAPC1, RBBP7, MBD1, MED8, ASH1L, MAP3K10, MAP3K13, TBX19, MED1, NEO1, SQSTM1, XBP1, BCL11B, RB1CC1, ETV1, HBP1, BCL6, ACTL6A, CC2D1A, ETV6, BAZ2A, MLLT3, POLR3F, JARID2, NLK, AFF4, YWHAB, AFF1, STAT3, PREB, ILF2, YWHAQ, RBM14, APBB2, MORF4L1, THRA, MORF4L2, CASK, FOXO1, FOXO3, LASS6, CBX7, PNN, WNT1, GATA6, TARDBP, MLL3, MYST4, GABPB2, MLL2, SATB2, ELL2, PTHLH, ZNF193, PAF1, MYNN, SOX3, SOX2, HDGF, SOX4, NEDD8, ELK3, SRF, NPM1, BTF3, RUNX1, MAFG, ZMYM2, FOXP1, ATF7IP2, HOXB4, PHF1, HABP4, PDCD6, NR6A1, FOXK2, ACVR1B, FUBP3, SOX17, MKL2, MKL1, IRAK2, KHDRBS1, NANOG, EGR2, FOXJ2, SOX11, SOX12, RUNX1T1, LEF1, FOXJ3, UBE2N, EYA1, BPTF, BTG2, BTG1, ZNF711, MDM4, ING5, ING3, ZNF800, HAT1, TRIB3, TCF7L1, TRIB1, NPAS2, NFAT5, POU2F1, POU3F2, ZNF701, ING1, ZNF700, TXNIP, ZBTB7A, KAT2B, HMBOX1, PSRC1, EDA2R, SIRT1, ATXN7L3, ATXN1, DR1, SP3,

GRLF1, IRF1, POFUT1, CRK, F2R, NFIB

GO:0010557~positive regulation of macromolecule biosynthetic process	146	8.00	1.32E-17	5.43E-14	MEF2C, CDX2, THRA, MORF4L2, CASK, HOXD13, FOXO1, TBP, FOXO3, HOXD10, CBFB, CITED2, IL11, EPC1, WNT1, APP, GATA6, WIBG, FOXF2, RARB, MYST4, SATB2, STRN3, MTA2, ARID1A, IRS1, DDIT3, TNKS2, HHEX, MAPK1, EP300, HIF1A, MTF1, HNF4A, VEGFA, PDGFRA, TGFB1I1, HOXA13, SOX2, NFKBIA, SOX4, SRF, LIF, TAF9, DYRK2, TCF4, RUNX1, ASF1A, TCF3, PLAGL2, TAF2, KLF6, DVL3, BMP2, MLL, IKZF2, EPAS1, KLF12, TGFBR1, SMAD5, FOXA1, NR4A2, NR4A1, SMAD2, NR4A3, DDX5, HDAC4, RNF6, NOTCH1, RNF4, HDAC1, ETS1, DYRK1B, SMARCC1, BMP7, BMP6, E2F1, PPARA, E2F3, RSF1, MITF, PPARG, SPI1, ZEB1, PAWR, GLI3, CCNE1, ZNF148, MKL2, NR2F2, FAM129A, USP16, MKL1, FOSL1, MYC, SERTAD2, TBL1XR1, EGR2, FOXJ2, SOX11, TP53, LEF1, RB1, CDK4, ARHGEF11, ADRB2, NCOA3, BPTF, EREG, NCOA4, NCOA6, UBC, ZFPM2, EIF5A2, MAPRE3, TBX19, CLOCK, MED1, ING5, TCF7L1, IGF1R, NPAS2, SQSTM1, BCL11B, NFAT5, POU2F1, POU3F2, THBS1, ING1, NFATC3, CEBPB, VHL, PSRC1, CREB5, AFF1, STAT3, ATXN7L3, TP73, ATXN1, PKNOX1, ILF2, IRF1, TBL1X, RBM14, NFIB, F2R

GO:0010605~negative regulation of macromolecule metabolic process	150	8.22	2.34E-14	9.66E-11	MEF2C, CDX2, THRA, TCEAL1, CITED2, EPC1, CDKN2A, ATG5, FOXF2, EED, RARB, BCL7A, MYST4, SATB2, STRN3, MTA2, RBL1, ZHX2, MECP2, PKIA, HHEX, PSMA1, ZNF238, HNF4A, IFNB1, ASB1, EIF2AK3, EIF2C3, RAD17, EIF2C4, CAV1, SOX2, ELK3, IGF2BP3, TIMP3, NR2C1, PSMB5, HEXIM1, PRKRA, NPM1, TAF9, UBE2D1, TCF4, DNMT3A, BMP2, KLF12, PTPN3, SMAD7, SMAD6, KLF11, L3MBTL, FOXA1, RYBP, CDC23, SKI, SMAD2, FURIN, UBP1, FOXP1, CDKN1C, HDAC4, HOXB4, PSMC6, HDAC1, RNF2, TRPS1, ADRA1B, HDAC9, DNAJB6, NCOR2, UBE2E1, HSP90AB1, E2F1, PPARA, COPS2, PPARD, RSF1, ARID4A, TSG101, DEDD, PPARG, NR6A1, SPI1, ZEB2, ZEB1, PAWR, DAXX, GLI3, ZNF148, HEY2, PSMD5, NR2F2, FAM129A, MYC, ZNF281, KHDRBS1, PPP2R1A, TBL1XR1, CTBP2, TP53, LEF1, RB1, RBBP7, MBD1, PURA, TARBP2, BPTF, EREG, PSME1, UBC, MAP3K10, MGEA5, PEBP1, BUB1B, ZFPM2, MDM4, SRP9, BTAF1, HAT1, FKBP1A, TCF7L1, HIC2, TSPYL2, SET, PPP2CA, RNF128, POU2F1, BCL6, THBS1, TNRC6B, BAZ2A, BUB3, ZBTB7A, MSH3, JARID2, VHL, YWHAB, PHF12, SIRT1, STAT3, ATXN1, PSMD11, DR1, BNIP3L, GRLF1, YWHAQ, HBEGF, VPS28, TBL1X, IGFBP5

GO:0010941~regulation of cell death	161	8.82	4.90E-14	2.02E-10	MEF2C, CADM1, ZAK, WFS1, FOXO1, PMAIP1, FOXO3, CITED2, CUL3, MAP3K7, ZFP91, CUL2, MAP3K5, APP, CDKN2A, ATG5, CD44, CDKN2C, TPT1, RARB, FAS, CUL1, ADAM9, PIM1, HBXIP, MARK4, STK4, DDIT3, MAPK1, IFNB1, F3, LYST, VEGFA, TNFAIP8, MAPK9, TNFAIP3, LRRK2, EIF2AK3, SYVN1, MCL1, HOXA13, PAFAH2, ERBB3, ERBB2, CLU, STK17B, RRAGA, NFKBIA, SOX4, AKAP13, BCL2L2, HSPA1B, TIMP3, SRC, SERINC3, PEA15, KRAS, PRKRA, MTCH1, NPM1, FAIM, TAF9, DYRK2, TRAF4, PLAGL2, B4GALT1, CARD8, ABR, BECN1, SMAD6, TGFBR1, NR4A2, NR4A1, FURIN, ATP7A, NOTCH2, P2RX4, ATF5, PLA2G4A, EPHA7, CDKN1A, P2RX7, NOTCH1, VCP, HDAC1, ETS1, BMP7, PDCD6, DNAJB6, DEDD, FASTK, ZMAT3, MITF, PRDX3, PAWR, GLI3, DAXX, PTEN, MCF2L, ACVR1B, CASP8, RHOA, DLG5, MKL1, CASP2, FOSL1, MYC, KCNMA1, ARHGEF3, PPP2R1A, NOL3, SLC25A4, ESR1, TP53, MBD4, PRKCE, ECT2, ARHGEF11, PROK2, EYA1, ADRB2, TNFRSF10B, BTG2, BTG1, BNIP2, IGF2R, UBC, MAP3K10, MAP3K11, YWHAZ, ING3, APH1A, XIAP, PPP3R1, NR3C1, IGF1R, SQSTM1, SOS1, RB1CC1, PPP2CA, BCL11B, BCL2, BCL6, THBS1, APC, TXNIP, CEBPB, VHL, YWHAB, BIRC3, SIRT1, TP73, SON, HSP90B1, EEF1E1, BNIP3L, JAK2, APBB2, DNM2, F2R, TP53INP1

GO:0043067~regulation of programmed cell death	159	8.71	1.57E-13	6.49E-10	MEF2C, CADM1, ZAK, WFS1, FOXO1, PMAIP1, FOXO3, CITED2, CUL3, MAP3K7, ZFP91, CUL2, MAP3K5, APP, CDKN2A, ATG5, CD44, CDKN2C, TPT1, RARB, FAS, CUL1, ADAM9, PIM1, HBXIP, MARK4, STK4, DDIT3, MAPK1, IFNB1, F3, LYST, VEGFA, TNFAIP8, MAPK9, TNFAIP3, LRRK2, EIF2AK3, SYVN1, MCL1, HOXA13, PAFAH2, ERBB3, ERBB2, CLU, STK17B, NFKBIA, SOX4, AKAP13, BCL2L2, HSPA1B, TIMP3, SRC, SERINC3, PEA15, KRAS, PRKRA, MTCH1, NPM1, FAIM, TAF9, DYRK2, TRAF4, PLAGL2, B4GALT1, CARD8, ABR, BECN1, SMAD6, TGFBR1, NR4A2, NR4A1, FURIN, ATP7A, NOTCH2, P2RX4, ATF5, PLA2G4A, EPHA7, CDKN1A, P2RX7, NOTCH1, VCP, HDAC1, ETS1, BMP7, PDCD6, DNAJB6, DEDD, FASTK, ZMAT3, MITF, PRDX3, PAWR, GLI3, DAXX, PTEN, MCF2L, ACVR1B, CASP8, RHOA, DLG5, MKL1, CASP2, FOSL1, MYC, KCNMA1, ARHGEF3, PPP2R1A, NOL3, ESR1, TP53, MBD4, PRKCE, ECT2, ARHGEF11, PROK2, EYA1, ADRB2, TNFRSF10B, BTG2, BTG1, BNIP2, IGF2R, UBC, MAP3K10, MAP3K11, YWHAZ, ING3, APH1A, XIAP, PPP3R1, NR3C1, IGF1R, SQSTM1, SOS1, RB1CC1, PPP2CA, BCL11B, BCL2, BCL6, THBS1, APC, TXNIP, CEBPB, VHL, YWHAB, BIRC3, SIRT1, TP73, SON, HSP90B1, EEF1E1, BNIP3L, JAK2, APBB2, DNM2, F2R, TP53INP1

GO:0042981~regulation of apoptosis	157	8.60	2.89E-13	1.19E-09	MEF2C, CADM1, ZAK, WFS1, FOXO1, PMAIP1, FOXO3, CITED2, CUL3, MAP3K7, ZFP91, CUL2, MAP3K5, APP, CDKN2A, ATG5, CD44, CDKN2C, TPT1, RARB, FAS, CUL1, ADAM9, PIM1, HBXIP, STK4, DDIT3, MAPK1, IFNB1, F3, LYST, VEGFA, TNFAIP8, MAPK9, TNFAIP3, EIF2AK3, SYVN1, MCL1, HOXA13, PAFAH2, ERBB3, ERBB2, CLU, STK17B, NFKBIA, SOX4, AKAP13, BCL2L2, HSPA1B, TIMP3, SRC, SERINC3, PEA15, KRAS, PRKRA, MTCH1, NPM1, FAIM, TAF9, DYRK2, TRAF4, PLAGL2, B4GALT1, CARD8, ABR, BECN1, SMAD6, TGFBR1, NR4A2, NR4A1, FURIN, ATP7A, NOTCH2, P2RX4, ATF5, PLA2G4A, EPHA7, CDKN1A, P2RX7, NOTCH1, VCP, HDAC1, ETS1, BMP7, PDCD6, DNAJB6, DEDD, FASTK, ZMAT3, MITF, PRDX3, PAWR, GLI3, DAXX, PTEN, MCF2L, ACVR1B, CASP8, RHOA, DLG5, MKL1, CASP2, FOSL1, MYC, KCNMA1, ARHGEF3, PPP2R1A, NOL3, ESR1, TP53, MBD4, PRKCE, ECT2, ARHGEF11, PROK2, EYA1, ADRB2, TNFRSF10B, BTG2, BTG1, BNIP2, IGF2R, UBC, MAP3K10, MAP3K11, YWHAZ, ING3, APH1A, XIAP, PPP3R1, NR3C1, IGF1R, SQSTM1, SOS1, RB1CC1, PPP2CA, BCL11B, BCL2, BCL6, THBS1, APC, TXNIP, CEBPB, VHL, YWHAB, BIRC3, SIRT1, TP73, SON, HSP90B1, EEF1E1, BNIP3L, JAK2, APBB2, DNM2, TP53INP1, F2R

GO:0045944~positive regulation of transcription from RNA polymerase II promoter	90	4.93	4.47E-13	1.84E-09	E2F1, MEF2C, PPARA, THRA, MORF4L2, MITF, PPARG, FOXO1, CASK, HOXD13, ZEB1, FOXO3, CBFB, HOXD10, IL11, CITED2, EPC1, APP, ZNF148, GATA6, RARB, MKL2, MYC, FOSL1, TBL1XR1, SATB2, EGR2, MTA2, STRN3, SOX11, TP53, LEF1, RB1, HHEX, ADRB2, EP300, HIF1A, MTF1, HNF4A, NCOA6, VEGFA, ZFPM2, TBX19, CLOCK, MED1, HOXA13, SOX2, NFKBIA, SRF, TCF7L1, LIF, NPAS2, SQSTM1, BCL11B, POU2F1, NFAT5, TAF9, POU3F2, RUNX1, NFATC3, TCF3, PLAGL2, TAF2, KLF6, BMP2, CEBPB, IKZF2, MLL, EPAS1, KLF12, FOXA1, SMAD5, NR4A2, NR4A1, SMAD2, NR4A3, STAT3, ATXN1, HDAC4, NOTCH1, PKNOX1, RNF4, HDAC1, ETS1, IRF1, RBM14, TBL1X, BMP7, NFIB, BMP6
GO:0016568~chromatin modification	73	4.00	7.70E-13	3.18E-09	MORF4L1, RSF1, EZH1, MORF4L2, HIRA, CBX7, EPC1, SMARCD2, PRMT5, RTF1, EED, TLK1, MLL3, USP16, MYST4, MLL2, TBL1XR1, SATB2, RBL2, RCOR1, RBL1, ARID1A, RB1, TAF6L, RBBP7, HLTF, UBE2B, UBE2N, EYA1, EP300, BPTF, ASH1L, SMARCA5, PAF1, JMJD1C, CARM1, MAP3K12, SUV420H1, SUPT6H, ING5, ING3, HAT1, CDC73, NR3C1, CHD9, TSPYL2, CHD1, ACTL6A, TAF9, ASF1A, BAZ2A, CHD5, DNMT3A, KAT2B, MLL, TAF5, NASP, L3MBTL, SMYD3, SIRT1, ATXN7L3, CTR9, RPS6KA5, HDAC4, PHF1, HDAC1, SMARCC1, RNF2, PHF15, H2AFY2, RBM14, HDAC9, UBE2E1

GO:0006350~transcription	328	17.97	1.58E-12	6.53E-09	MEF2C, CDX2, HIRA, HOXD13, PTTG1, MXI1, HOXD10, CITED2, EPC1, ZFP91, CRY2, MED28, FOXF2, PHTF2, RARB, TBPL1, RCOR3, RCOR1, YY1, ZHX2, MECP2, EOMES, MED11, ZNF791, HNF4G, TAF6L, ZNF236, ZNF238, RFC1, PRDM4, MTF1, HNF4A, PIAS3, PARP14, ZZZ3, PRDM2, TGFB1I1, SUPT6H, SUV420H1, CRTC3, CRTC2, HOXA13, GPBP1L1, AHCTF1, ZNF618, ZNF512, TCF20, HEXIM1, HOXA10, ZNF124, TCF4, TCF3, PLAGL2, ASXL2, PLAG1, IKZF4, KLF6, MLL, IKZF2, KLF9, KLF12, SMAD7, ZNF121, SMAD6, SMAD5, KLF11, L3MBTL, RYBP, ZBTB41, SMAD2, UBP1, ZNF629, NOTCH2, NOTCH1, RNF4, ETS1, DMTF1, TRPS1, RNF2, TCF12, NCOR2, KLF3, CCNT2, COPS2, PPARA, PPARD, ELF2, ZBTB34, EZH1, ZNF530, PPARG, MITF, SPI1, ZEB2, ZEB1, DAXX, GLI3, HSF2, ZNF148, NR2F2, USP16, MYB, TWISTNB, MYC, SERTAD2, ZNF281, SNAPC2, BRF2, SNAPC1, TP53, TLE4, RBBP7, MBD1, ZNF335, ZNF140, BRWD1, EREG, MED8, ASH1L, ZNF551, CLOCK, TBX19, MED1, POLR2H, CREM, ZNF655, ZNF175, MYCBP2, XBP1, RB1CC1, BCL11B, NPAT, ETV1, BCL6, HBP1, ACTL6A, CC2D1A, ETV6, BAZ2A, TRIP11, ZNF562, MLLT3, POLR3F, SSRP1, ZNF264, ZNF565, JARID2, NLK, AFF4, PPP1R10, PHF10, PHF12, TP73, STAT3, PREB, RLF, MEF2D, PAPOLA, ILF2, ZBTB5, RBM14, MORF4L1, THRA, MORF4L2, FOXO1, TBP, FOXO3, CBX7, TCEAL1, CBFB, PNN, CNOT4, CDKN2A, SMARCD2, MIER2, GATA6, TARDBP, EED, MLL3, GABPB2, MYST4, MLL2, ZNF594, RBL2, RBL1, RELB, ARID1A, ZNF3, DDIT3, ELL2, ZNF193, HHEX, ZNF439, EP300, HIF1A, SMARCA5, TFAP2A, NFE2L1, PAF1, MYNN, ZNF586, CAMTA1, LITAF, SOX3,

SOX2, BMPR2, HDGF, SOX4, ELK3, SRF, NR2C1, ZNF695, ZNF597, BTF3, TAF9, RUNX1, ASF1A, TAF2, MAFG, ZMYM2, EPAS1, TAF5, FOXA1, NR4A2, NR4A1, WHSC2, NR4A3, ATF7IP2, FOXP1, HDAC4, ATF5, HOXB4, HDAC1, PHF1, SMARCC1, PNRC1, HABP4, ZNF318, HDAC9, PDCD6, ZNF410, E2F1, E2F3, RSF1, ARID4A, E2F5, GPBP1, DEDD, ARID4B, FOXK2, NR6A1, PAWR, TMF1, MAX, FUBP3, PRMT5, HEY2, RTF1, MKL2, SOX17, MKL1, FOSL1, KHDRBS1, TBL1XR1, NANOG, EGR2, PTGER3, FOXJ2, SOX11, ZFX, SOX12, RUNX1T1, CDK8, ESR1, LEF1, RB1, FOXJ3, HLTF, HMGA2, CCNL2, PURA, HOXC10, EYA1, HOXC11, NCOA3, BPTF, BTG2, NCOA4, NCOA6, ZNF711, ZFPM2, JMJD1C, CARM1, ING5, ING3, ZNF800, TRIB3, CDC73, NR3C1, TCF7L1, CHD9, TFAM, HIC2, NPAS2, TSPYL2, TSC22D4, NFAT5, POU2F1, POU3F2, NFATC3, ZNF701, CHD5, ZNF700, TXNIP, ZBTB7A, CEBPB, KAT2B, HMBOX1, CREB5, SIRT1, ATXN7L3, SP3, DR1, IRF1, GRLF1, DENND4A, TBL1X, NFIB

GO:0045184~establishment of protein localization	149	8.16	2.43E-12	1.00E-08	LTBP2, SELENBP1, KLHL2, RAB1A, SSR1, KIF13A, CRY2, ANK3, TLK1, VPS13B, NUP35, SAR1B, SAR1A, SCAMP5, NUP133, SCAMP2, ATG9A, MYH9, NUPL1, CLPX, MAPK1, LYST, AKAP5, RAB14, SDCBP, PDCD6IP, RAB10, DERL2, SNX5, MTX2, SNX7, AKAP12, NFKBIA, CCDC91, CTSA, ABCA1, LMAN1, RAB40B, STX12, NPM1, NUP54, DOPEY1, TRAM1, AP2M1, GABARAPL2, ABCB9, RAB8B, TAOK2, LIN7B, LIN7C, ICMT, ARFIP1, EPS15, RABEP1, VCP, TRPS1, ARF3, SPTBN1, GOSR1, LGTN, SMURF1, FBXO34, CLTA, RAB5B, TSG101, ZMAT3, TIMM17A, TIMM17B, PPARG, CLTC, GLI3, RAB3IP, AP1S1, BLZF1, AP2B1, TRIM3, AP1S2, RNF103, ZFYVE16, ZFYVE9, RANBP6, RANBP2, TPR, SEC24C, SEC24D, KDELR1, TOMM34, STX6, SEC23A, STX1A, PGAP1, TP53, STXBP3, RAB11FIP5, MYRIP, ZDHHC17, IPO7, ARCN1, MGEA5, GNAS, KPNA4, KPNA3, KPNA2, SNX13, EIF5A2, RAB11FIP1, KPNA1, SRP9, SERP1, YWHAZ, SNX16, NXT2, UEVLD, TMED2, CEP57, PEX19, PPP3CB, AP3D1, BCL6, PEX13, APPBP2, PPP3CA, TNPO1, FAM125B, VTA1, NASP, YWHAB, PPP1R10, AP4S1, MON2, PREB, HSP90B1, RAB30, TOM1L1, ERBB2IP, GRIA2, AP2A1, MCFD2, RAB22A, RAB34, YWHAQ, JAK2, LRP2, SLC15A4, VPS28, SSR2, F2R, TOB1, SSR3

GO:0015031~protein transport	147	8.05	4.96E-12	2.04E-08	LTBP2, SELENBP1, KLHL2, RAB1A, SSR1, KIF13A, CRY2, TLK1, VPS13B, NUP35, SAR1B, SAR1A, SCAMP5, NUP133, SCAMP2, ATG9A, MYH9, NUPL1, CLPX, MAPK1, LYST, AKAP5, RAB14, SDCBP, PDCD6IP, RAB10, DERL2, SNX5, MTX2, SNX7, AKAP12, NFKBIA, CCDC91, CTSA, ABCA1, LMAN1, RAB40B, STX12, NPM1, NUP54, DOPEY1, TRAM1, AP2M1, GABARAPL2, ABCB9, RAB8B, TAOK2, LIN7B, LIN7C, ICMT, ARFIP1, EPS15, RABEP1, VCP, TRPS1, ARF3, SPTBN1, GOSR1, LGTN, SMURF1, FBXO34, CLTA, RAB5B, TSG101, ZMAT3, TIMM17A, TIMM17B, PPARG, CLTC, GLI3, RAB3IP, AP1S1, BLZF1, AP2B1, TRIM3, AP1S2, RNF103, ZFYVE16, ZFYVE9, RANBP6, RANBP2, TPR, SEC24C, SEC24D, KDELR1, TOMM34, STX6, SEC23A, STX1A, PGAP1, TP53, STXBP3, RAB11FIP5, MYRIP, ZDHHC17, IPO7, ARCN1, MGEA5, GNAS, KPNA4, KPNA3, KPNA2, SNX13, EIF5A2, RAB11FIP1, KPNA1, SRP9, SERP1, YWHAZ, SNX16, NXT2, UEVLD, TMED2, CEP57, PEX19, PPP3CB, AP3D1, BCL6, PEX13, APPBP2, PPP3CA, TNPO1, FAM125B, VTA1, NASP, YWHAB, PPP1R10, AP4S1, MON2, PREB, HSP90B1, RAB30, TOM1L1, ERBB2IP, AP2A1, MCFD2, RAB22A, RAB34, YWHAQ, JAK2, LRP2, SLC15A4, VPS28, SSR2, F2R, TOB1, SSR3

GO:0046907~intracellular transport	131	7.18	8.11E-12	3.34E-08	NRBP1, LTBP2, KLHL2, SSR1, APP, CRY2, TLK1, SAR1B, SAR1A, NUP133, SCAMP2, KIF5B, MYH9, HHEX, MAPK1, NDEL1, VAMP8, LYST, AKAP5, RAB14, SLC25A37, SDCBP, VAMP2, TRAPPC1, DERL2, MTX2, AKAP12, NFKBIA, CTSA, ABCA1, LMAN1, STX12, NPM1, NUP54, DOPEY1, TRAM1, AP2M1, RHOBTB3, XPOT, GABARAPL2, KIF3B, TAOK2, MAP2K1, SMG7, LIN7B, ICMT, ARFIP1, NXF1, SLC25A13, ATP2A2, VCP, TRPS1, RHOT1, SPTBN1, GOSR1, LGTN, SMURF1, CLTA, TIMM17A, TIMM17B, CLTC, GLI3, RAB3IP, AP2B1, AP1S1, AP1S2, ZFYVE16, ZFYVE9, RANBP2, TPR, SEC24C, KDELR1, SEC24D, GOLGA3, TOMM34, STX6, SEC23A, ARL1, STX1A, PGAP1, TP53, M6PR, KIF1C, MYRIP, ADRB2, KIF1B, IPO7, ARCN1, MGEA5, GNAS, KPNA4, KPNA3, KPNA2, SRP9, KPNA1, YWHAZ, EEA1, TPM1, DTNBP1, SET, PEX19, CEP57, SQSTM1, KLC1, BCL2, PPP3CB, AP3D1, PAFAH1B1, BCL6, PEX13, PPP3CA, APPBP2, TNPO1, HSPA8, YWHAB, PPP1R10, AP4S1, MON2, PREB, ATXN1, ERBB2IP, TOM1L1, AP2A1, YWHAQ, JAK2, LRP2, SSR2, DNM2, TOB1, SSR3, F2R

GO:0006796~phosphate metabolic process	176	9.64	8.88E-12	3.66E-08	NRBP1, ZAK, STK38, EFNA1, CASK, RPS6KB1, MAP3K7, TOP1, MAP3K5, APP, CSNK2A1, CLK3, MAP3K9, AAK1, GAB1, MAP3K8, TLK1, PRKACB, FRS2, ADAM9, PTPRJ, TWF1, CSNK1G1, WNK1, PIM1, PPP1CC, STK4, MARK4, WEE1, MARK1, SPAG9, MAPK1, MAP4K5, MAPK4, EIF2S1, MAPK3, DLD, PDGFRA, MAPK9, NEK9, LRRK2, EIF2AK3, LRRK1, PPP2R3A, ENPP2, NEK2, ERBB3, ERBB2, MAPKAPK5, STK17B, BMPR2, CHEK1, DUSP11, SRC, VRK1, DUSP14, PRKRA, ZAP70, LMTK2, DULLARD, DYRK4, DYRK2, PTPN9, BMP2, MAP2K1, TAOK2, PTPN3, SMAD7, TAOK1, RYK, TGFBR1, PTPN4, TAOK3, MAP2K4, MET, TGFBR2, SMAD2, PTPN12, EPHA5, EPHA4, EPHA7, P2RX7, DYRK1B, DYRK1A, CDC42BPA, SPTBN1, TGFBR3, PTPN1, BMP7, ATP6V0E1, IMPA1, ATP5B, FASTK, PIP5K1B, PTEN, LATS1, DAXX, RNGTT, ACVR1B, SLK, PKN3, CXCR4, BRD4, AKT3, CHUK, IRAK2, CDC7, PPP2R1A, SRPK2, LIMK1, INPPL1, PKN2, CDK8, MINK1, PRKCH, CDK6, DGUOK, PRKCE, NDUFA10, CDK4, ATP6V1D, SRPK1, SACM1L, PPM1G, PROK2, PDIK1L, CCND1, EYA1, MAP3K10, MAP3K14, MAP3K13, MAP3K12, UGP2, MAP3K11, PPP6C, ABI2, TRIB3, AKAP9, PPM1B, TRIB1, MTMR2, MTMR3, IGF1R, MAP3K3, MAP3K2, PPP2CA, BCL2, PPP3CB, CAMK2B, PPP3CA, PPAP2A, YES1, THBS1, MTMR4, PIK3R1, PTPRD, FLT1, NLK, AXL, MON2, CDC25A, CDC25B, RPS6KA5, GMFB, DUSP4, RPS6KA3, DUSP2, RPS6KA4, FYN, RPS6KA2, PTP4A2, JAK1, JAK2, DUSP8, F2R, BMPR1A

GO:0006793~phosphorus metabolic process	176	9.64	8.88E-12	3.66E-08	NRBP1, ZAK, STK38, EFNA1, CASK, RPS6KB1, MAP3K7, TOP1, MAP3K5, APP, CSNK2A1, CLK3, MAP3K9, AAK1, GAB1, MAP3K8, TLK1, PRKACB, FRS2, ADAM9, PTPRJ, TWF1, CSNK1G1, WNK1, PIM1, PPP1CC, STK4, MARK4, WEE1, MARK1, SPAG9, MAPK1, MAP4K5, MAPK4, EIF2S1, MAPK3, DLD, PDGFRA, MAPK9, NEK9, LRRK2, EIF2AK3, LRRK1, PPP2R3A, ENPP2, NEK2, ERBB3, ERBB2, MAPKAPK5, STK17B, BMPR2, CHEK1, DUSP11, SRC, VRK1, DUSP14, PRKRA, ZAP70, LMTK2, DULLARD, DYRK4, DYRK2, PTPN9, BMP2, MAP2K1, TAOK2, PTPN3, SMAD7, TAOK1, RYK, TGFBR1, PTPN4, TAOK3, MAP2K4, MET, TGFBR2, SMAD2, PTPN12, EPHA5, EPHA4, EPHA7, P2RX7, DYRK1B, DYRK1A, CDC42BPA, SPTBN1, TGFBR3, PTPN1, BMP7, ATP6V0E1, IMPA1, ATP5B, FASTK, PIP5K1B, PTEN, LATS1, DAXX, RNGTT, ACVR1B, SLK, PKN3, CXCR4, BRD4, AKT3, CHUK, IRAK2, CDC7, PPP2R1A, SRPK2, LIMK1, INPPL1, PKN2, CDK8, MINK1, PRKCH, CDK6, DGUOK, PRKCE, NDUFA10, CDK4, ATP6V1D, SRPK1, SACM1L, PPM1G, PROK2, PDIK1L, CCND1, EYA1, MAP3K10, MAP3K14, MAP3K13, MAP3K12, UGP2, MAP3K11, PPP6C, ABI2, TRIB3, AKAP9, PPM1B, TRIB1, MTMR2, MTMR3, IGF1R, MAP3K3, MAP3K2, PPP2CA, BCL2, PPP3CB, CAMK2B, PPP3CA, PPAP2A, YES1, THBS1, MTMR4, PIK3R1, PTPRD, FLT1, NLK, AXL, MON2, CDC25A, CDC25B, RPS6KA5, GMFB, DUSP4, RPS6KA3, DUSP2, RPS6KA4, FYN, RPS6KA2, PTP4A2, JAK1, JAK2, DUSP8, F2R, BMPR1A

GO:0031327~negative regulation of cellular biosynthetic process	116	6.36	1.38E-11	5.69E-08	MEF2C, CDX2, THRA, TCEAL1, CITED2, EPC1, HTR1B, FOXF2, EED, RARB, BCL7A, MYST4, SATB2, MTA2, STRN3, RBL1, MECP2, ZHX2, PKIA, HHEX, ZNF238, IFNB1, ASB1, EIF2AK3, EIF2C3, RAD17, EIF2C4, CAV1, SOX2, IGF2BP3, ELK3, NR2C1, HEXIM1, TCF4, DNMT3A, BMP2, KLF12, SMAD7, L3MBTL, KLF11, FOXA1, RYBP, SMAD2, SKI, UBP1, FOXP1, CDKN1C, HOXB4, HDAC4, HDAC1, RNF2, TRPS1, HDAC9, NCOR2, DNAJB6, E2F1, PPARA, COPS2, PPARD, RSF1, ARID4A, TSG101, DEDD, PPARG, NR6A1, SPI1, ZEB2, PAWR, ZEB1, DAXX, GLI3, ZNF148, HEY2, NR2F2, KHDRBS1, ZNF281, TBL1XR1, CTBP2, TP53, LEF1, RB1, RBBP7, MBD1, PURA, BPTF, EREG, GRM7, MAP3K10, MGEA5, ZFPM2, MDM4, SRP9, BTAF1, HAT1, TCF7L1, HIC2, TSPYL2, RNF128, POU2F1, BCL6, BAZ2A, ZBTB7A, VHL, JARID2, YWHAB, PHF12, SIRT1, STAT3, ATXN1, GLA, DR1, GRLF1, YWHAQ, HBEGF, TBL1X, IGFBP5

GO:0051252~regulation of RNA metabolic process	287	15.73	1.38E-11	5.70E-08	MEF2C, CDX2, HIRA, HOXD13, SYNCRIP, RAB1A, HOXD10, CITED2, EPC1, APP, FOXF2, RARB, TBPL1, STRN3, YY1, ZHX2, MECP2, EOMES, MED11, ZNF791, HNF4G, TAF6L, ZNF236, ZNF238, HNF4A, MTF1, SERBP1, VEGFA, PRDM2, TGFB1I1, SUPT6H, HOXA13, NFKBIA, LIF, TCF20, HEXIM1, HOXA10, ZNF124, PABPC1, TCF4, TCF3, PLAGL2, DNMT3A, DVL3, KLF6, BMP2, IKZF2, MLL, KLF9, MAP2K1, KLF12, SMAD7, PAIP1, SMAD6, SMAD5, L3MBTL, KLF11, RYBP, SKI, CELSR2, SMAD2, EN2, UBP1, NOTCH2, NOTCH1, RNF6, RNF4, ETS1, DMTF1, TRPS1, RNF2, BMP7, TCF12, NCOR2, BMP6, PPARA, PPARD, ELF2, TSG101, ZNFX1, PPARG, MITF, ZNF530, SPI1, ZEB2, ZEB1, DAXX, CCNE1, BLZF1, HSF2, ZNF148, NR2F2, USP16, MYB, MYC, SERTAD2, ZNF281, CTBP2, BRF2, TP53, RBBP7, SRPK1, ZNF140, MYCN, ARHGEF11, SS18, BRWD1, MED8, C19ORF2, ZNF551, CLOCK, TBX19, MED1, CREM, ZNF655, ZNF175, ZFP36L1, SQSTM1, XBP1, BCL11B, NPAT, CSDE1, ETV1, BCL6, ETV6, BAZ2A, ZNF562, MLLT3, POLR3F, ZNF264, ZNF565, JARID2, ELAVL2, PHF12, AFF1, TP73, STAT3, RPS6KA5, PREB, MEF2D, PKNOX1, RPS6KA4, ILF2, YWHAQ, RBM14, THRA, MORF4L2, FOXO1, CASK, TBP, FOXO3, LASS6, TCEAL1, CBFB, IL11, WNT1, CDKN2A, SMARCD2, GATA6, MLL3, MYST4, MLL2, SATB2, MTA2, RBL1, RELB, ARID1A, PKIA, DDIT3, ZNF3, ZNF193, HHEX, ZNF439, EP300, HIF1A, SMARCA5, TFAP2A, NFE2L1, ZNF586, LITAF, SOX2, SOX4, NEDD8, ELK3, SRF, NR2C1, ZNF695, NPM1, ZNF597, TAF9, RUNX1, ASF1A, TAF2, MAFG, EPAS1, FOXA1, NR4A2, NR4A1, NR4A3, FOXP1, CDKN1C,

HDAC4, ATF5, HOXB4, HDAC1, DYRK1B, SMARCC1, HDAC9, PDCD6, DNAJB6, E2F1, E2F3, RSF1, ARID4A, E2F5, FOXK2, NR6A1, PAWR, ZNF207, TMF1, HEY2, RHOA, MKL2, FOSL1, TBL1XR1, NANOG, EGR2, FOXJ2, SOX11, SOX12, RUNX1T1, ESR1, LEF1, RB1, FOXJ3, MBNL1, HLTF, HMGA2, PURA, TARBP1, HOXC10, TARBP2, ADRB2, HOXC11, NCOA3, BPTF, NCOA4, NCOA6, ZFPM2, JMJD1C, MDM4, CARM1, MAPRE3, HAT1, NR3C1, TCF7L1, HIC2, TFAM, NPAS2, TSC22D3, TSC22D2, TSC22D4, NFAT5, POU2F1, CHD1, POU3F2, NFATC3, ZNF701, ZNF700, ZBTB7A, CEBPB, KAT2B, VHL, HMBOX1, PSRC1, CREB5, SIRT1, ATXN7L3, ATXN1, SP3, DR1, IRF1, DENND4A, TBL1X, CRK, NFIB, F2R

GO:0042127~regulation of cell proliferation	149	8.16	1.51E-11	6.24E-08	CDX2, HOXD13, FOXO1, RPS6KB1, JAG1, IL15, MXI1, SSR1, IL11, CUL3, ZFP91, CUL2, CDKN2A, CDKN2C, SERPINE1, RARB, ITCH, CUL1, CGRRF1, CD40, VASH2, IRS1, MARK4, PDCD1LG2, PTHLH, HHEX, MAPK1, HIF1A, HNF4A, IFNB1, F3, VEGFA, PDGFRA, TGFB1I1, LAMC1, DERL2, CAV1, ERBB3, ERBB2, SOX2, CLU, BMPR2, NFKBIA, SOX4, CHEK1, TIMP2, SESN1, LIF, KRAS, AGGF1, PRKRA, NPM1, ZAP70, AXIN2, TCF3, B4GALT1, MAFG, BMP2, MLL, BECN1, TGFBR1, TGFBR2, KLF11, ICMT, SKI, SMAD2, FOXP1, CDKN1C, HDAC4, NOTCH2, PLA2G4A, CDKN1A, NOTCH1, HDAC1, ETS1, TGFBR3, BMP7, PLAU, PPARD, E2F3, TSG101, MARCKSL1, MITF, PPARG, NAP1L1, GJA1, GNRHR, ZEB1, PRDX3, PAWR, GLI3, PTEN, WISP2, ARHGAP5, GPC3, HEY2, DLG5, MYC, FOSL1, CDC7, CTBP2, TP53, CDK6, RB1, CDK4, PURA, MYCN, HOXC10, CCND1, ADRB2, BTG2, EREG, ADM, CCND2, BTG1, BTG3, MDM4, EIF5A2, TBX19, ING5, EGLN3, CDH5, TRIB1, IGF1R, BCL11B, BCL2, BCL6, POU3F2, PPAP2A, THBS1, ING1, APC, TXNIP, FLT1, KAT2B, IL8, JARID2, VHL, SIRT1, CDC25B, EEF1E1, HBEGF, JAK2, NFIB, LRP5, BMPR1A, TOB1, IGFBP5, F2R

GO:0010629~negative regulation of gene expression	107	5.86	1.82E-11	7.50E-08	MEF2C, THRA, CDX2, TCEAL1, CITED2, EPC1, FOXF2, EED, RARB, BCL7A, MYST4, SATB2, MTA2, STRN3, RBL1, MECP2, ZHX2, PKIA, HHEX, ZNF238, IFNB1, EIF2C3, EIF2C4, SOX2, ELK3, NR2C1, HEXIM1, PRKRA, TCF4, DNMT3A, BMP2, KLF12, SMAD7, L3MBTL, KLF11, FOXA1, RYBP, SMAD2, SKI, UBP1, FOXP1, CDKN1C, HOXB4, HDAC4, HDAC1, RNF2, TRPS1, HDAC9, NCOR2, DNAJB6, E2F1, COPS2, PPARA, PPARD, RSF1, ARID4A, TSG101, DEDD, PPARG, NR6A1, SPI1, ZEB2, PAWR, ZEB1, DAXX, GLI3, ZNF148, HEY2, NR2F2, MYC, KHDRBS1, ZNF281, TBL1XR1, CTBP2, TP53, LEF1, RB1, RBBP7, MBD1, PURA, TARBP2, BPTF, EREG, MAP3K10, ZFPM2, MDM4, BTAF1, HAT1, TCF7L1, HIC2, POU2F1, BCL6, TNRC6B, BAZ2A, ZBTB7A, VHL, JARID2, YWHAB, PHF12, SIRT1, STAT3, ATXN1, DR1, BNIP3L, YWHAQ, GRLF1, TBL1X
GO:0016481~negative regulation of transcription	100	5.48	1.93E-11	7.94E-08	MEF2C, THRA, CDX2, TCEAL1, CITED2, EPC1, FOXF2, EED, RARB, BCL7A, MYST4, SATB2, MTA2, STRN3, RBL1, MECP2, ZHX2, PKIA, HHEX, ZNF238, IFNB1, SOX2, ELK3, NR2C1, HEXIM1, TCF4, DNMT3A, BMP2, KLF12, SMAD7, L3MBTL, KLF11, FOXA1, RYBP, SMAD2, SKI, UBP1, FOXP1, CDKN1C, HOXB4, HDAC4, HDAC1, RNF2, TRPS1, HDAC9, NCOR2, DNAJB6, E2F1, COPS2, PPARA, PPARD, RSF1, ARID4A, TSG101, DEDD, PPARG, NR6A1, SPI1, ZEB2, PAWR, ZEB1, DAXX, GLI3, ZNF148, HEY2, NR2F2, KHDRBS1, ZNF281, TBL1XR1, CTBP2, TP53, LEF1, RB1, RBBP7, MBD1, PURA, BPTF, EREG, MAP3K10, ZFPM2, MDM4, BTAF1, HAT1, TCF7L1, HIC2, POU2F1, BCL6, BAZ2A, ZBTB7A, VHL, JARID2, YWHAB, PHF12, SIRT1, STAT3,

ATXN1, DR1, YWHAQ, GRLF1, TBL1X

GO:0008104~protein localization	161	8.82	3.91E-11	1.61E-07	OXA1L, LTBP2, SELENBP1, MXI1, KLHL2, RAB1A, SSR1, KIF13A, CRY2, VPS13C, ANK3, TLK1, VPS13B, NUP35, SAR1B, SAR1A, SCAMP5, NUP133, SCAMP2, ATG9A, MYH9, NUPL1, CLPX, TNKS2, MAPK1, LYST, AKAP5, RAB14, SDCBP, PDCD6IP, RAB10, DERL2, CAV1, SNX5, MTX2, SNX7, AKAP12, NFKBIA, CCDC91, NEDD8, CTSA, ABCA1, LMAN1, RAB40B, STX12, NPM1, NUP54, DOPEY1, TRAM1, SRGN, AP2M1, GABARAPL2, ABCB9, RAB8B, TAOK2, LIN7B, LIN7C, ICMT, ARFIP1, EPS15, RABEP1, VCP, TRPS1, ARF3, SPTBN1, GOSR1, LGTN, SMURF1, FBXO34, CLTA, RAB5B, TSG101, ZMAT3, TIMM17A, TIMM17B, PPARG, NBEA, CLTC, GLI3, RAB3IP, CDC42, AP1S1, BLZF1, AP2B1, TRIM3, AP1S2, RNF103, ZFYVE16, ZFYVE9, RANBP6, RANBP2, TPR, SEC24C, SEC24D, KDELR1, TOMM34, STX6, SEC23A, STX1A, PGAP1, G3BP2, TP53, STXBP3, RAB11FIP5, MYRIP, ZDHHC17, IPO7, ARCN1, MGEA5, GNAS, KPNA4, KPNA3, KPNA2, SNX13, EIF5A2, RAB11FIP1, KPNA1, SRP9, SERP1, YWHAZ, SHROOM3, SNX16, NXT2, UEVLD, TMED2, CEP57, PEX19, SQSTM1, PPP3CB, AP3D1, BCL6, PEX13, APPBP2, PPP3CA, TNPO1, FAM125B, VTA1, NASP, YWHAB, PPP1R10, AP4S1, MON2, PREB, HSP90B1, RAB30, TOM1L1, ERBB2IP, GRIA2, AP2A1, MCFD2, RAB22A, RAB34, YWHAQ, JAK2, LRP2, SLC15A4, VPS28, SSR2, F2R, TOB1, SSR3

GO:0007243~protein kinase cascade	85	4.66	4.51E-11	1.86E-07	STK38, ZAK, EFNA1, PRKAG2, RPS6KB1, ITPKB, MBIP, DAXX, MAP3K7, MAP3K5, CXCR4, MAP3K9, GAB1, PRKACB, RAPGEF2, FRS2, CHUK, ADAM9, IRAK2, SRPK2, PPP2R1A, SOCS6, G3BP2, WNK1, MINK1, STK4, IRS1, SRPK1, MARK1, MAPK1, SPAG9, PROK2, SS18, MAP4K5, CRKL, TNFRSF10B, MAP3K10, MAPK9, LRRK2, MAP3K13, MAP3K12, MAP3K11, WNT5A, CAV1, ERBB3, ERBB2, STK17B, NFKBIA, AKAP11, SRC, TRIB1, TMED7, IGF1R, MAP3K3, MAP3K2, PPP2CA, RB1CC1, ZAP70, PKD2, PKD1, THBS1, PIK3R1, FLT1, MAP2K1, TAOK2, TGFBR1, NLK, MET, MAP2K4, TAOK3, STAT3, RPS6KA5, DUSP4, RPS6KA3, P2RX7, RPS6KA4, DUSP2, RPS6KA2, FYN, ADRA1B, JAK1, TGFBR3, JAK2, DUSP8, F2R

GO:0006468~protein amino acid phosphorylation	130	7.12	4.92E-11	2.03E-07	NRBP1, ZAK, STK38, EFNA1, CASK, RPS6KB1, MAP3K7, MAP3K5, APP, CSNK2A1, CLK3, AAK1, MAP3K9, GAB1, MAP3K8, TLK1, PRKACB, FRS2, ADAM9, TWF1, CSNK1G1, WNK1, PIM1, MARK4, STK4, WEE1, MARK1, SPAG9, MAPK1, MAP4K5, EIF2S1, MAPK4, MAPK3, PDGFRA, MAPK9, NEK9, LRRK2, LRRK1, EIF2AK3, ERBB3, NEK2, MAPKAPK5, ERBB2, STK17B, BMPR2, CHEK1, SRC, VRK1, PRKRA, ZAP70, LMTK2, DYRK4, DYRK2, BMP2, TAOK2, MAP2K1, SMAD7, RYK, TAOK1, TGFBR1, TAOK3, MAP2K4, MET, TGFBR2, SMAD2, EPHA5, EPHA4, EPHA7, P2RX7, DYRK1B, DYRK1A, CDC42BPA, SPTBN1, TGFBR3, BMP7, FASTK, LATS1, DAXX, ACVR1B, SLK, PKN3, CXCR4, BRD4, AKT3, CHUK, IRAK2, CDC7, SRPK2, LIMK1, CDK8, PKN2, PRKCH, MINK1, CDK6, DGUOK, PRKCE, CDK4, SRPK1, PROK2, PDIK1L, CCND1, MAP3K10, MAP3K14, MAP3K13, MAP3K12, MAP3K11, TRIB3, ABI2, AKAP9, TRIB1, IGF1R, MAP3K3, MAP3K2, BCL2, CAMK2B, THBS1, YES1, FLT1, NLK, AXL, RPS6KA5, GMFB, RPS6KA3, RPS6KA4, FYN, RPS6KA2, JAK1, JAK2, BMPR1A, F2R

GO:0051172~negative regulation of nitrogen compound metabolic process	108	5.92	5.10E-11	2.10E-07	MEF2C, THRA, CDX2, TCEAL1, CITED2, EPC1, HTR1B, FOXF2, EED, RARB, BCL7A, MYST4, SATB2, MTA2, STRN3, RBL1, MECP2, ZHX2, PKIA, HHEX, ZNF238, IFNB1, RAD17, CAV1, SOX2, ELK3, NR2C1, HEXIM1, NPM1, TCF4, DNMT3A, BMP2, KLF12, SMAD7, L3MBTL, KLF11, FOXA1, RYBP, SMAD2, SKI, UBP1, FOXP1, CDKN1C, HOXB4, HDAC4, HDAC1, RNF2, TRPS1, HDAC9, NCOR2, DNAJB6, E2F1, COPS2, PPARA, PPARD, RSF1, ARID4A, TSG101, DEDD, PPARG, NR6A1, SPI1, ZEB2, PAWR, ZEB1, DAXX, GLI3, ZNF148, HEY2, NR2F2, KHDRBS1, ZNF281, TBL1XR1, CTBP2, TP53, LEF1, RB1, RBBP7, MBD1, PURA, BPTF, EREG, GRM7, MAP3K10, ZFPM2, MDM4, BTAF1, HAT1, TCF7L1, HIC2, TSPYL2, POU2F1, BCL6, BAZ2A, ZBTB7A, MSH3, VHL, JARID2, YWHAB, PHF12, SIRT1, STAT3, ATXN1, GLA, DR1, YWHAQ, GRLF1, TBL1X

GO:0009890~negative regulation of biosynthetic process	116	6.36	5.57E-11	2.30E-07	MEF2C, CDX2, THRA, TCEAL1, CITED2, EPC1, HTR1B, FOXF2, EED, RARB, BCL7A, MYST4, SATB2, MTA2, STRN3, RBL1, MECP2, ZHX2, PKIA, HHEX, ZNF238, IFNB1, ASB1, EIF2AK3, EIF2C3, RAD17, EIF2C4, CAV1, SOX2, IGF2BP3, ELK3, NR2C1, HEXIM1, TCF4, DNMT3A, BMP2, KLF12, SMAD7, L3MBTL, KLF11, FOXA1, RYBP, SMAD2, SKI, UBP1, FOXP1, CDKN1C, HOXB4, HDAC4, HDAC1, RNF2, TRPS1, HDAC9, NCOR2, DNAJB6, E2F1, PPARA, COPS2, PPARD, RSF1, ARID4A, TSG101, DEDD, PPARG, NR6A1, SPI1, ZEB2, PAWR, ZEB1, DAXX, GLI3, ZNF148, HEY2, NR2F2, KHDRBS1, ZNF281, TBL1XR1, CTBP2, TP53, LEF1, RB1, RBBP7, MBD1, PURA, BPTF, EREG, GRM7, MAP3K10, MGEA5, ZFPM2, MDM4, SRP9, BTAF1, HAT1, TCF7L1, HIC2, TSPYL2, RNF128, POU2F1, BCL6, BAZ2A, ZBTB7A, VHL, JARID2, YWHAB, PHF12, SIRT1, STAT3, ATXN1, GLA, DR1, GRLF1, YWHAQ, HBEGF, TBL1X, IGFBP5

GO:0006355~regulation of transcription, DNA-dependent	279	15.29	5.68E-11	2.34E-07	MEF2C, CDX2, HIRA, HOXD13, RAB1A, HOXD10, CITED2, EPC1, APP, FOXF2, RARB, TBPL1, STRN3, YY1, ZHX2, MECP2, EOMES, MED11, ZNF791, HNF4G, TAF6L, ZNF236, ZNF238, HNF4A, MTF1, VEGFA, PRDM2, TGFB1I1, SUPT6H, HOXA13, NFKBIA, LIF, TCF20, HEXIM1, HOXA10, ZNF124, TCF4, TCF3, PLAGL2, DNMT3A, DVL3, KLF6, BMP2, IKZF2, MLL, KLF9, MAP2K1, KLF12, SMAD7, SMAD6, SMAD5, L3MBTL, KLF11, RYBP, SKI, CELSR2, SMAD2, EN2, UBP1, NOTCH2, NOTCH1, RNF6, RNF4, ETS1, DMTF1, TRPS1, RNF2, BMP7, TCF12, NCOR2, BMP6, PPARA, PPARD, ELF2, TSG101, ZNFX1, PPARG, MITF, ZNF530, SPI1, ZEB2, ZEB1, DAXX, CCNE1, BLZF1, HSF2, ZNF148, NR2F2, USP16, MYB, MYC, SERTAD2, ZNF281, CTBP2, BRF2, TP53, RBBP7, ZNF140, MYCN, ARHGEF11, SS18, BRWD1, MED8, C19ORF2, ZNF551, CLOCK, TBX19, MED1, CREM, ZNF655, ZNF175, SQSTM1, XBP1, BCL11B, NPAT, CSDE1, ETV1, BCL6, ETV6, BAZ2A, ZNF562, MLLT3, POLR3F, ZNF264, ZNF565, JARID2, ELAVL2, PHF12, AFF1, TP73, STAT3, RPS6KA5, PREB, MEF2D, PKNOX1, RPS6KA4, ILF2, YWHAQ, RBM14, THRA, MORF4L2, FOXO1, CASK, TBP, FOXO3, LASS6, TCEAL1, CBFB, IL11, WNT1, CDKN2A, SMARCD2, GATA6, MLL3, MYST4, MLL2, SATB2, MTA2, RBL1, RELB, ARID1A, PKIA, DDIT3, ZNF3, ZNF193, HHEX, ZNF439, EP300, HIF1A, SMARCA5, TFAP2A, NFE2L1, ZNF586, LITAF, SOX2, SOX4, NEDD8, ELK3, SRF, NR2C1, ZNF695, ZNF597, TAF9, RUNX1, ASF1A, TAF2, MAFG, EPAS1, FOXA1, NR4A2, NR4A1, NR4A3, FOXP1, CDKN1C, HDAC4, ATF5, HOXB4, HDAC1, DYRK1B, SMARCC1, HDAC9,

PDCD6, DNAJB6, E2F1, E2F3, RSF1, ARID4A, E2F5, NR6A1, FOXK2, PAWR, ZNF207, TMF1, HEY2, RHOA, MKL2, FOSL1, TBL1XR1, NANOG, EGR2, FOXJ2, SOX11, SOX12, RUNX1T1, ESR1, LEF1, RB1, FOXJ3, HLTF, HMGA2, PURA, TARBP1, HOXC10, TARBP2, ADRB2, HOXC11, NCOA3, BPTF, NCOA4, NCOA6, ZFPM2, JMJD1C, MDM4, CARM1, MAPRE3, HAT1, NR3C1, TCF7L1, HIC2, TFAM, NPAS2, TSC22D3, TSC22D2, TSC22D4, NFAT5, POU2F1, CHD1, POU3F2, ZNF701, NFATC3, ZNF700, ZBTB7A, CEBPB, KAT2B, VHL, HMBOX1, PSRC1, CREB5, SIRT1, ATXN7L3, ATXN1, DR1, SP3, IRF1, DENND4A, TBL1X, CRK, NFIB, F2R

GO:0010558~negative regulation of macromolecule biosynthetic process	112	6.14	6.09E-11	2.51E-07	MEF2C, CDX2, THRA, TCEAL1, CITED2, EPC1, FOXF2, EED, RARB, BCL7A, MYST4, SATB2, MTA2, STRN3, RBL1, MECP2, ZHX2, PKIA, HHEX, ZNF238, IFNB1, ASB1, EIF2AK3, RAD17, EIF2C3, EIF2C4, SOX2, IGF2BP3, ELK3, NR2C1, HEXIM1, TCF4, DNMT3A, BMP2, KLF12, SMAD7, L3MBTL, KLF11, FOXA1, RYBP, SMAD2, SKI, UBP1, FOXP1, CDKN1C, HOXB4, HDAC4, HDAC1, RNF2, TRPS1, HDAC9, NCOR2, DNAJB6, E2F1, PPARA, COPS2, PPARD, RSF1, ARID4A, TSG101, DEDD, PPARG, NR6A1, SPI1, ZEB2, PAWR, ZEB1, DAXX, GLI3, ZNF148, HEY2, NR2F2, KHDRBS1, ZNF281, TBL1XR1, CTBP2, TP53, LEF1, RB1, RBBP7, MBD1, PURA, BPTF, EREG, MAP3K10, MGEA5, ZFPM2, MDM4, SRP9, BTAF1, HAT1, TCF7L1, HIC2, TSPYL2, RNF128, POU2F1, BCL6, BAZ2A, ZBTB7A, VHL, JARID2, YWHAB, PHF12, SIRT1, STAT3, ATXN1, DR1, GRLF1, YWHAQ, HBEGF, TBL1X, IGFBP5

GO:0007242~intracellular signaling cascade	211	11.56	7.34E-11	3.02E-07	PLEKHM1, EFNA1, ADCY6, RAB1A, MAP3K7, MAP3K5, HTR1B, CTGF, MAP3K9, GAB1, RAPGEF4, TLK1, DEPDC1B, RAPGEF2, ADAM9, GTPBP2, MAGI3, WNK1, DEPDC1, MARK1, DEPDC5, SPAG9, RND3, MAPK1, MAPK3, RAB14, MAPK9, TGFB1I1, ARL8B, RAB10, EIF2AK3, DCBLD2, ERBB3, ERBB2, STK17B, NFKBIA, AKAP13, CHEK1, AKAP11, ARFGEF2, RAB40B, KRAS, ZAP70, DVL3, ABR, MAP2K1, TAOK2, KLF9, SMAD7, TGFBR1, MAP2K4, TAOK3, SMAD2, HOMER1, RNF4, ARF3, CDC42BPA, TGFBR3, RIT1, RAB5B, SHOC2, DAXX, LATS1, MCF2L, CCNE1, ARL5A, NISCH, GUCY1A3, RAP2A, SRPK2, ARHGEF3, RAP2C, TP53, MBD4, SRPK1, ARHGEF11, SS18, PROK2, CCND1, TNFRSF10B, ADM, GNB2, GNB1, MAP3K10, GNB4, GNAS, ARL4C, MAP3K13, MAP3K12, MAP3K11, MED1, CREM, PLCL2, SQSTM1, SOS1, PPP2CA, RB1CC1, PKD2, PKD1, THBS1, IL8, SPSB1, NLK, YWHAB, STAT3, TP73, RPS6KA5, DUSP4, RPS6KA3, DUSP2, RAB30, RPS6KA4, RPS6KA2, FYN, RAB34, RAB22A, YWHAQ, RBM14, APBB2, DUSP8, THRA, STK38, ZAK, RPS6KB1, ITPKB, MBIP, RGL1, RRAS, PRKACB, MLL3, FRS2, SAR1A, STMN3, SOCS6, ARID1A, STK4, IRS1, PTHLH, MAP4K5, ASB1, SDCBP, STMN1, LRRK2, LRRK1, RAD17, WNT5A, CAV1, ABCA1, SRC, RHOBTB1, DYRK2, ARHGDIB, RAB8B, MET, SHANK2, P2RX7, PLCG1, RHOT1, ADRA1B, RAP1A, PRKAG2, CDC42, ARHGAP5, GSN, CXCR4, ARHGAP1, RHOA, RHOC, DLG5, PLCB1, CHUK, C9ORF86, IRAK2, ARL1, PPP2R1A, PTGER3, LIMK1, ESR1, G3BP2, MINK1, PRKCH, ADIPOR1, RB1, PRKCE, ECT2, ADRB2, CRKL, NCOA3, NCOA4, NCOA6, BUB1B, CARM1, NR3C1, TRIB1,

TYMS, TMED7, IGF1R, MAP3K3, MAP3K2, PPAP2A, PIK3R1, FLT1, WSB1, SH3BP5, RASL11B, WSB2, JAK1, JAK2, CRK, F2R, IGFBP5

GO:0045934~negative regulation of nucleobase, nucleoside, nucleotide and nucleic acid metabolic process	106	5.81	1.08E-10	4.47E-07	MEF2C, THRA, CDX2, TCEAL1, CITED2, EPC1, HTR1B, FOXF2, EED, RARB, BCL7A, MYST4, SATB2, MTA2, STRN3, RBL1, MECP2, ZHX2, PKIA, HHEX, ZNF238, IFNB1, RAD17, SOX2, ELK3, NR2C1, HEXIM1, NPM1, TCF4, DNMT3A, BMP2, KLF12, SMAD7, L3MBTL, KLF11, FOXA1, RYBP, SMAD2, SKI, UBP1, FOXP1, CDKN1C, HOXB4, HDAC4, HDAC1, RNF2, TRPS1, HDAC9, NCOR2, DNAJB6, E2F1, COPS2, PPARA, PPARD, RSF1, ARID4A, TSG101, DEDD, PPARG, NR6A1, SPI1, ZEB2, PAWR, ZEB1, DAXX, GLI3, ZNF148, HEY2, NR2F2, KHDRBS1, ZNF281, TBL1XR1, CTBP2, TP53, LEF1, RB1, RBBP7, MBD1, PURA, BPTF, EREG, GRM7, MAP3K10, ZFPM2, MDM4, BTAF1, HAT1, TCF7L1, HIC2, TSPYL2, POU2F1, BCL6, BAZ2A, ZBTB7A, MSH3, VHL, JARID2, YWHAB, PHF12, SIRT1, STAT3, ATXN1, DR1, YWHAQ, GRLF1, TBL1X

GO:0032268~regulation of cellular protein metabolic process	100	5.48	1.32E-10	5.45E-07	ZAK, IL11, ZFP91, APP, MAP3K5, CDKN2A, ATG5, MAP3K9, WIBG, GAB1, RAPGEF4, PDGFD, PUM2, CUL1, ADAM9, PSMA1, MAPK1, MAP4K5, EP300, HNF4A, PIAS3, EIF2S1, LRRK2, EIF2AK3, EIF2C3, EIF2C4, CAV1, BMPR2, NFKBIA, IGF2BP3, TIMP3, LIF, PSMB5, TAF9, UBE2D1, BMP2, MLL, PTPN3, SMAD7, PAIP1, TGFBR1, SMAD6, CDC23, CASC3, FURIN, HDAC4, P2RX7, PSMC6, SMURF1, BMP7, UBE2E1, HSP90AB1, METAP1, PRKAG2, ZEB2, DAXX, QKI, PSMD5, FAM129A, PPP2R1A, ACO1, CDK4, PRKCE, UBE2N, EIF4G2, TARBP2, EIF4G3, CCND1, PSME1, CCND3, CCND2, EIF4A2, UBC, MAP3K10, MGEA5, BUB1B, PEBP1, EIF5A2, SRP9, MAP3K11, FKBP1A, ZFP36L1, SET, MAP3K2, BCL2, PPP2CA, RB1CC1, KRT7, THBS1, TNRC6B, BUB3, YWHAB, ETF1, TP73, ATXN2, PSMD11, JAK2, VPS28, IGFBP5, BMPR1A
GO:0001568~blood vessel development	63	3.45	1.67E-10	6.88E-07	NRP2, CDX2, LMO2, ATP5B, FOXO1, JAG1, PTEN, MMP2, CITED2, MAP3K7, SHB, CD44, ATG5, CTGF, CXCR4, HEY2, CASP8, QKI, SOX17, MKL2, NR2F2, CYR61, RECK, PTPRJ, SGPL1, MYH9, SLIT2, PROK2, SH2D2A, CRKL, HIF1A, EREG, VEGFA, AAMP, ZFPM2, CAV1, TIPARP, ELK3, SRF, CDH5, ZFP36L1, AGGF1, PKD1, THBS1, B4GALT1, FLT1, EPAS1, IL8, VHL, SMAD7, TGFBR1, TGFBR2, ITGA4, UBP1, COL5A1, ATP7A, NOTCH1, PKNOX1, ADRA1B, AMOT, TGFBR3, POFUT1, PLAU

GO:0001944~vasculature development	64	3.51	1.71E-10	7.06E-07	NRP2, CDX2, LMO2, ATP5B, FOXO1, JAG1, MMP2, PTEN, CITED2, MAP3K7, SHB, CD44, ATG5, CTGF, CXCR4, HEY2, CASP8, QKI, SOX17, MKL2, NR2F2, CYR61, PTPRJ, RECK, SGPL1, EFNB2, MYH9, SLIT2, PROK2, SH2D2A, CRKL, HIF1A, EREG, VEGFA, AAMP, ZFPM2, CAV1, TIPARP, ELK3, SRF, CDH5, ZFP36L1, AGGF1, PKD1, THBS1, B4GALT1, FLT1, EPAS1, IL8, VHL, SMAD7, TGFBR1, TGFBR2, ITGA4, UBP1, COL5A1, ATP7A, NOTCH1, PKNOX1, ADRA1B, AMOT, TGFBR3, POFUT1, PLAU
GO:0051325~interphase	37	2.03	2.29E-10	9.42E-07	E2F1, PPP6C, CHEK1, ZNF655, LATS1, CUL3, CCNE1, ACVR1B, CUL2, APP, CDKN2A, CDKN2C, BCL2, KRT7, NPAT, PPP3CB, CAMK2B, PPP3CA, TCF3, CUL1, KHDRBS1, CDC7, TAF2, PIM1, CDC23, CDK6, RB1, CDK4, MARK4, CDC25A, CDKN1C, CDKN1A, CCND1, CCND2, KPNA2, MAP3K11, DNM2

GO:0042325~regulation of phosphorylation	98	5.37	2.47E-10	1.02E-06	CCNT2, ZAK, EFNA1, ADCY6, PRKAG2, ZEB2, MBIP, PRDX3, PTEN, LATS1, DAXX, IL11, CCNE2, MAP3K7, CDC42, ZFP91, MAP3K5, APP, CDKN2A, CDKN2C, CXCR4, MAP3K9, GAB1, RAPGEF4, PRKACB, PDGFD, FAM129A, FRS2, ADAM9, PPP2R1A, RBL2, RBL1, PIM1, RB1, PRKCE, PKIA, IRS1, SPAG9, TARBP2, PROK2, ADRB2, CCND1, MAP4K5, TNFRSF10B, CCND3, HNF4A, EREG, CCND2, MAP3K10, PEBP1, MAP3K13, RAD17, MAP3K11, CAV1, ERBB2, BMPR2, TRIB3, FKBP1A, CHEK1, TRIB1, LIF, TSPYL2, KRAS, HEXIM1, MAP3K2, PPP2CA, BCL2, RB1CC1, NPM1, PPAP2A, THBS1, APC, BMP2, FLT1, MAP2K1, TAOK2, SMAD7, SMAD6, TGFBR1, MET, TGFBR2, CDC25A, TP73, CDC25B, CDKN1C, ATXN1, SH3BP5, ATP7A, CDKN1A, P2RX7, LRP1, DUSP2, JAK2, BMP7, DUSP8, F2R, VLDLR, BMPR1A
GO:0006325~chromatin organization	84	4.60	3.43E-10	1.41E-06	MORF4L1, RSF1, ARID4A, EZH1, MORF4L2, ARID4B, HIRA, NAP1L1, CBX7, EPC1, SMARCD2, PRMT5, RTF1, EED, TLK1, USP16, MLL3, MYST4, MLL2, H1F0, TBL1XR1, SATB2, RBL2, RCOR1, MTA2, RBL1, ARID1A, RB1, TAF6L, HMGA2, HLTF, RBBP7, UBE2B, UBE2N, EYA1, EP300, BPTF, ASH1L, SMARCA5, PAF1, JMJD1C, CARM1, MAP3K12, SUV420H1, SUPT6H, ING5, ING3, SOX2, HAT1, CDC73, NR3C1, TCF7L1, CHD9, SET, TSPYL2, NPM1, CHD1, ACTL6A, TAF9, ASF1A, BAZ2A, CHD5, DNMT3A, KAT2B, MLL, TAF5, NASP, L3MBTL, SMYD3, SIRT1, ATXN7L3, CTR9, RPS6KA5, HDAC4, PHF1, HDAC1, SMARCC1, RNF2, PHF15, H2AFY2, H3F3B, RBM14, HDAC9, UBE2E1

GO:0019220~regulation of phosphate metabolic process	100	5.48	4.90E-10	2.02E-06	ZAK, EFNA1, ADCY6, MBIP, IL11, MAP3K7, ZFP91, APP, MAP3K5, CDKN2A, CDKN2C, MAP3K9, GAB1, RAPGEF4, PDGFD, PRKACB, FRS2, ADAM9, RBL2, RBL1, PIM1, IRS1, PKIA, SPAG9, MAP4K5, HNF4A, RAD17, CAV1, ERBB2, BMPR2, CHEK1, LIF, KRAS, HEXIM1, NPM1, BMP2, TAOK2, MAP2K1, SMAD7, TGFBR1, SMAD6, TGFBR2, SMG7, MET, ATP7A, CDKN1C, P2RX7, CDKN1A, BMP7, CCNT2, PRKAG2, ZEB2, PRDX3, DAXX, LATS1, PTEN, CCNE2, CDC42, CXCR4, FAM129A, PPP2R1A, RB1, PRKCE, PROK2, TARBP2, CCND1, ADRB2, TNFRSF10B, EREG, CCND3, CCND2, MAP3K10, PEBP1, MAP3K13, MAP3K11, TRIB3, FKBP1A, TRIB1, TSPYL2, MAP3K2, RB1CC1, BCL2, PPP2CA, THBS1, PPAP2A, APC, FLT1, YWHAB, TP73, CDC25A, CDC25B, SH3BP5, ATXN1, DUSP2, LRP1, JAK2, DUSP8, BMPR1A, VLDLR, F2R

GO:0051174~regulation of phosphorus metabolic process	100	5.48	4.90E-10	2.02E-06	ZAK, EFNA1, ADCY6, MBIP, IL11, MAP3K7, ZFP91, APP, MAP3K5, CDKN2A, CDKN2C, MAP3K9, GAB1, RAPGEF4, PDGFD, PRKACB, FRS2, ADAM9, RBL2, RBL1, PIM1, IRS1, PKIA, SPAG9, MAP4K5, HNF4A, RAD17, CAV1, ERBB2, BMPR2, CHEK1, LIF, KRAS, HEXIM1, NPM1, BMP2, TAOK2, MAP2K1, SMAD7, TGFBR1, SMAD6, TGFBR2, SMG7, MET, ATP7A, CDKN1C, P2RX7, CDKN1A, BMP7, CCNT2, PRKAG2, ZEB2, PRDX3, DAXX, LATS1, PTEN, CCNE2, CDC42, CXCR4, FAM129A, PPP2R1A, RB1, PRKCE, PROK2, TARBP2, CCND1, ADRB2, TNFRSF10B, EREG, CCND3, CCND2, MAP3K10, PEBP1, MAP3K13, MAP3K11, TRIB3, FKBP1A, TRIB1, TSPYL2, MAP3K2, RB1CC1, BCL2, PPP2CA, THBS1, PPAP2A, APC, FLT1, YWHAB, TP73, CDC25A, CDC25B, SH3BP5, ATXN1, DUSP2, LRP1, JAK2, DUSP8, BMPR1A, VLDLR, F2R
GO:0051253~negative regulation of RNA metabolic process	81	4.44	5.15E-10	2.12E-06	E2F1, MEF2C, PPARA, PPARD, CDX2, RSF1, ARID4A, TSG101, NR6A1, PPARG, SPI1, PAWR, ZEB1, DAXX, TCEAL1, CITED2, EPC1, ZNF148, FOXF2, HEY2, RARB, NR2F2, ZNF281, TBL1XR1, SATB2, CTBP2, MTA2, STRN3, RBL1, MECP2, TP53, ZHX2, LEF1, RB1, RBBP7, PKIA, PURA, HHEX, ZNF238, BPTF, ZFPM2, MDM4, SOX2, HAT1, TCF7L1, NR2C1, HIC2, HEXIM1, NPM1, POU2F1, BCL6, TCF4, BAZ2A, DNMT3A, ZBTB7A, BMP2, KLF12, VHL, JARID2, SMAD7, FOXA1, KLF11, RYBP, SKI, SMAD2, PHF12, SIRT1, FOXP1, STAT3, CDKN1C, HOXB4, HDAC4, HDAC1, DR1, TRPS1, RNF2, YWHAQ, TBL1X, HDAC9, NCOR2, DNAJB6

GO:0045892~negative regulation of transcription, DNA-dependent	80	4.38	5.45E-10	2.25E-06	E2F1, MEF2C, PPARA, PPARD, CDX2, RSF1, ARID4A, TSG101, NR6A1, PPARG, SPI1, PAWR, ZEB1, DAXX, TCEAL1, CITED2, EPC1, ZNF148, FOXF2, HEY2, RARB, NR2F2, ZNF281, TBL1XR1, SATB2, CTBP2, MTA2, STRN3, RBL1, MECP2, TP53, ZHX2, LEF1, RB1, RBBP7, PKIA, PURA, HHEX, ZNF238, BPTF, ZFPM2, MDM4, SOX2, HAT1, TCF7L1, NR2C1, HIC2, HEXIM1, POU2F1, BCL6, TCF4, BAZ2A, DNMT3A, ZBTB7A, BMP2, KLF12, VHL, JARID2, SMAD7, FOXA1, KLF11, RYBP, SKI, SMAD2, PHF12, SIRT1, FOXP1, STAT3, CDKN1C, HOXB4, HDAC4, HDAC1, DR1, TRPS1, RNF2, YWHAQ, TBL1X, HDAC9, NCOR2, DNAJB6

GO:0007049~cell cycle	142	7.78	5.67E-10	2.34E-06	ZAK, PTTG1, WTAP, CUL3, CUL2, APP, CDKN2A, CDKN2C, INCENP, TARDBP, MAP3K8, TLK1, CUL1, STAG2, STAG1, CGRRF1, RBL2, RBL1, PIM1, MND1, MYH9, PPP1CC, MARK4, WEE1, DDIT3, HHEX, PSMA1, MAPK1, EP300, MAPK4, MAPK3, NEK9, STMN1, PDCD6IP, RAD17, NEK2, ANAPC13, CHEK1, CCNG2, SESN1, SESN3, PSMB5, NIPBL, NPM1, UBE2D1, TCF3, TAF2, PARD6B, MLL, MAP2K1, CDC23, PCNP, CDKN1C, NOTCH2, CDKN1A, PSMC6, RGS2, DMTF1, RNF2, ZNF318, UBE2E1, CCNT2, E2F1, E2F3, LZTS2, TSG101, SENP5, LATS1, DAXX, CCNE2, KIF2C, CCNE1, ACVR1B, RNF103, PSMD5, USP16, MYC, CDC7, KHDRBS1, TP53, NUSAP1, CDK6, RB1, HMGA2, CDK4, PPM1G, EIF4G2, CCND1, CCND3, EREG, PSME1, CCND2, UBC, BUB1B, SIAH1, MAPRE2, MDM4, MAPRE1, KPNA2, MAPRE3, MAP3K11, PPP6C, RABGAP1, CETN3, CDC73, ZNF655, CYLD, NUMA1, TSPYL2, KRT7, RB1CC1, BCL2, NPAT, PKD2, PPP3CB, PKD1, PAFAH1B1, CAMK2B, HBP1, PPP3CA, THBS1, ING1, BUB3, APC, TXNIP, EXO1, KAT2B, IL8, PDS5A, NASP, PSRC1, CDC25A, RGS14, TP73, CDC25B, ERBB2IP, PSMD11, RASSF2, APBB2, CALM2, DNM2, TP53INP1

GO:0010942~positive regulation of cell death	92	5.04	6.91E-10	2.85E-06	ZAK, CADM1, DEDD, ZMAT3, FASTK, PAWR, FOXO3, PMAIP1, DAXX, PTEN, MCF2L, CUL3, CUL2, ACVR1B, MAP3K5, APP, CDKN2A, CD44, CDKN2C, CASP8, RARB, FAS, CASP2, MYC, FOSL1, CUL1, KCNMA1, PPP2R1A, ARHGEF3, TP53, MBD4, PRKCE, STK4, ECT2, MARK4, DDIT3, ARHGEF11, MAPK1, ADRB2, TNFRSF10B, IFNB1, LYST, UBC, MAP3K10, MAPK9, LRRK2, MAP3K11, ING3, APH1A, HOXA13, STK17B, PPP3R1, SOX4, AKAP13, RRAGA, NR3C1, TIMP3, SRC, SERINC3, SQSTM1, SOS1, BCL2, PPP2CA, PRKRA, MTCH1, BCL6, DYRK2, PLAGL2, APC, TXNIP, B4GALT1, CEBPB, ABR, TGFBR1, YWHAB, NR4A1, TP73, NOTCH2, PLA2G4A, EPHA7, CDKN1A, NOTCH1, P2RX7, ETS1, EEF1E1, BNIP3L, JAK2, APBB2, BMP7, PDCD6, TP53INP1, DNM2
GO:0008285~negative regulation of cell proliferation	80	4.38	1.09E-09	4.49E-06	PPARD, TSG101, PPARG, GJA1, GNRHR, PAWR, ZEB1, IL15, MXI1, PTEN, GLI3, CUL2, WISP2, CDKN2A, GPC3, CDKN2C, DLG5, RARB, ITCH, FOSL1, CUL1, CTBP2, CGRRF1, TP53, CDK6, RB1, PDCD1LG2, PTHLH, BTG2, ADM, HNF4A, EREG, IFNB1, BTG1, BTG3, TGFB1I1, MDM4, ING5, CAV1, ERBB2, CHEK1, TIMP2, SESN1, CDH5, TRIB1, BCL2, BCL11B, NPM1, PRKRA, BCL6, THBS1, AXIN2, PPAP2A, ING1, APC, B4GALT1, BMP2, KAT2B, MLL, IL8, BECN1, VHL, JARID2, TGFBR2, KLF11, SMAD2, SKI, CDKN1C, NOTCH2, HDAC4, CDKN1A, ETS1, EEF1E1, TGFBR3, JAK2, BMP7, NFIB, IGFBP5, F2R, TOB1

GO:0006886~intracellular protein transport	82	4.49	1.12E-09	4.62E-06	CLTA, LTBP2, TIMM17A, TIMM17B, CLTC, GLI3, KLHL2, SSR1, AP2B1, AP1S1, AP1S2, CRY2, ZFYVE16, ZFYVE9, TLK1, RANBP2, TPR, SEC24C, SAR1B, SAR1A, KDELR1, SEC24D, TOMM34, STX6, SEC23A, STX1A, PGAP1, TP53, MAPK1, MYRIP, IPO7, ARCN1, AKAP5, MGEA5, SDCBP, KPNA4, KPNA3, KPNA2, SRP9, KPNA1, DERL2, YWHAZ, MTX2, AKAP12, NFKBIA, CTSA, STX12, PEX19, CEP57, NPM1, PPP3CB, AP3D1, BCL6, PEX13, PPP3CA, NUP54, APPBP2, TRAM1, TNPO1, AP2M1, TAOK2, LIN7B, YWHAB, PPP1R10, ICMT, ARFIP1, AP4S1, VCP, ERBB2IP, TOM1L1, AP2A1, TRPS1, YWHAQ, SPTBN1, JAK2, GOSR1, SMURF1, LGTN, SSR2, SSR3, F2R, TOB1
GO:0043068~positive regulation of programmed cell death	91	4.99	1.21E-09	5.00E-06	ZAK, CADM1, DEDD, ZMAT3, FASTK, PAWR, FOXO3, PMAIP1, DAXX, PTEN, MCF2L, CUL3, CUL2, ACVR1B, MAP3K5, APP, CDKN2A, CD44, CDKN2C, CASP8, RARB, FAS, CASP2, MYC, FOSL1, CUL1, KCNMA1, PPP2R1A, ARHGEF3, TP53, MBD4, PRKCE, STK4, ECT2, MARK4, DDIT3, ARHGEF11, MAPK1, ADRB2, TNFRSF10B, IFNB1, LYST, UBC, MAP3K10, MAPK9, LRRK2, MAP3K11, ING3, APH1A, HOXA13, STK17B, PPP3R1, SOX4, AKAP13, NR3C1, TIMP3, SRC, SERINC3, SQSTM1, SOS1, BCL2, PPP2CA, PRKRA, MTCH1, BCL6, DYRK2, PLAGL2, APC, TXNIP, B4GALT1, CEBPB, ABR, TGFBR1, YWHAB, NR4A1, TP73, NOTCH2, PLA2G4A, EPHA7, CDKN1A, NOTCH1, P2RX7, ETS1, EEF1E1, BNIP3L, JAK2, APBB2, BMP7, PDCD6, TP53INP1, DNM2

GO:0070727~cellular macromolecule localization	88	4.82	1.31E-09	5.40E-06	CLTA, OXA1L, LTBP2, TIMM17A, TIMM17B, CLTC, GLI3, RAB3IP, KLHL2, SSR1, CDC42, AP2B1, AP1S1, AP1S2, CRY2, ZFYVE16, ZFYVE9, TLK1, RANBP2, TPR, SEC24C, SAR1B, SAR1A, KDELR1, SEC24D, TOMM34, STX6, SEC23A, STX1A, PGAP1, TP53, TNKS2, MAPK1, MYRIP, IPO7, ARCN1, AKAP5, MGEA5, SDCBP, KPNA4, KPNA3, KPNA2, SRP9, KPNA1, DERL2, YWHAZ, MTX2, AKAP12, NFKBIA, CTSA, STX12, PEX19, CEP57, NPM1, PPP3CB, AP3D1, BCL6, PEX13, PPP3CA, NUP54, APPBP2, TRAM1, TNPO1, SRGN, AP2M1, TAOK2, YWHAB, LIN7B, PPP1R10, ICMT, CASC3, ARFIP1, AP4S1, VCP, ERBB2IP, TOM1L1, AP2A1, TRPS1, YWHAQ, SPTBN1, JAK2, GOSR1, SMURF1, LGTN, SSR2, SSR3, F2R, TOB1
GO:0051329~interphase of mitotic cell cycle	35	1.92	1.69E-09	6.97E-06	E2F1, PPP6C, CHEK1, LATS1, CUL3, CCNE1, ACVR1B, CUL2, APP, CDKN2A, CDKN2C, BCL2, NPAT, PPP3CB, CAMK2B, PPP3CA, TCF3, CUL1, KHDRBS1, CDC7, TAF2, PIM1, CDC23, CDK6, RB1, CDK4, MARK4, CDC25A, CDKN1C, CDKN1A, CCND1, CCND2, KPNA2, MAP3K11, DNM2

GO:0034613~cellular protein localization	87	4.77	2.03E-09	8.38E-06	CLTA, OXA1L, LTBP2, TIMM17A, TIMM17B, CLTC, GLI3, RAB3IP, KLHL2, SSR1, CDC42, AP2B1, AP1S1, AP1S2, CRY2, ZFYVE16, ZFYVE9, TLK1, RANBP2, TPR, SEC24C, SAR1B, SAR1A, KDELR1, SEC24D, TOMM34, STX6, SEC23A, STX1A, PGAP1, TP53, TNKS2, MAPK1, MYRIP, IPO7, ARCN1, AKAP5, MGEA5, SDCBP, KPNA4, KPNA3, KPNA2, SRP9, KPNA1, DERL2, YWHAZ, MTX2, AKAP12, NFKBIA, CTSA, STX12, PEX19, CEP57, NPM1, PPP3CB, AP3D1, BCL6, PEX13, PPP3CA, NUP54, APPBP2, TRAM1, TNPO1, SRGN, AP2M1, TAOK2, YWHAB, LIN7B, PPP1R10, ICMT, ARFIP1, AP4S1, VCP, ERBB2IP, TOM1L1, AP2A1, TRPS1, YWHAQ, SPTBN1, JAK2, GOSR1, SMURF1, LGTN, SSR2, SSR3, F2R, TOB1
GO:0043065~positive regulation of apoptosis	89	4.88	4.12E-09	1.70E-05	ZAK, CADM1, DEDD, ZMAT3, FASTK, PAWR, FOXO3, PMAIP1, DAXX, PTEN, MCF2L, CUL3, CUL2, ACVR1B, MAP3K5, APP, CDKN2A, CD44, CDKN2C, CASP8, RARB, FAS, CASP2, MYC, FOSL1, CUL1, KCNMA1, PPP2R1A, ARHGEF3, TP53, MBD4, PRKCE, STK4, ECT2, DDIT3, ARHGEF11, MAPK1, ADRB2, TNFRSF10B, IFNB1, LYST, UBC, MAP3K10, MAPK9, MAP3K11, ING3, APH1A, HOXA13, STK17B, PPP3R1, AKAP13, SOX4, NR3C1, TIMP3, SRC, SERINC3, SQSTM1, SOS1, BCL2, PPP2CA, PRKRA, MTCH1, BCL6, DYRK2, PLAGL2, APC, TXNIP, B4GALT1, CEBPB, ABR, TGFBR1, YWHAB, NR4A1, TP73, NOTCH2, PLA2G4A, EPHA7, CDKN1A, NOTCH1, P2RX7, ETS1, EEF1E1, BNIP3L, JAK2, APBB2, BMP7, PDCD6, TP53INP1, DNM2

GO:0009967~positive regulation of signal transduction	68	3.73	4.19E-09	1.73E-05	PPARD, GJA1, SHOC2, ITPKB, ZEB2, JAG1, IL11, CITED2, MAP3K7, ACVR1B, ZFP91, GPC3, CASP8, RHOA, RHOC, LTBR, ESR1, CD40, ECT2, IRS1, UBE2N, HHEX, ZDHHC17, ADRB2, TNFRSF10B, HIF1A, EREG, NCOA3, F3, VEGFA, SEMA4C, PEBP1, TGFB1I1, CAV1, LITAF, ERBB3, ERBB2, SOX2, SOX4, FKBP1A, SRC, LIF, TMED7, KRAS, ECE1, MAP3K3, SOS1, ZAP70, CC2D1A, THBS1, BMP2, FLT1, TAOK2, TGFBR1, TAOK3, EDA2R, FURIN, CDKN1C, P2RX4, NOTCH2, P2RX7, CDKN2AIP, EEF1E1, GOLPH3, JAK2, BMP7, F2R, BMPR1A
GO:0043549~regulation of kinase activity	77	4.22	8.15E-09	3.36E-05	CCNT2, ZAK, EFNA1, PRKAG2, ADCY6, ZEB2, MBIP, PRDX3, DAXX, LATS1, PTEN, CCNE2, MAP3K7, CDC42, MAP3K5, APP, CDKN2A, CDKN2C, CXCR4, MAP3K9, GAB1, PRKACB, FRS2, ADAM9, PPP2R1A, RBL2, RBL1, PIM1, RB1, IRS1, PKIA, SPAG9, PROK2, TARBP2, MAP4K5, CCND1, ADRB2, TNFRSF10B, EREG, CCND3, CCND2, MAP3K10, MAP3K13, MAP3K11, CAV1, ERBB2, TRIB3, CHEK1, TRIB1, KRAS, TSPYL2, HEXIM1, MAP3K2, PPP2CA, NPM1, THBS1, PPAP2A, APC, FLT1, MAP2K1, TAOK2, TGFBR1, TGFBR2, MET, TP73, CDC25A, CDC25B, CDKN1C, SH3BP5, CDKN1A, P2RX7, LRP1, DUSP2, JAK2, DUSP8, F2R, VLDLR

GO:0051276~chromosome organization	96	5.26	1.01E-08	4.15E-05	MORF4L1, RSF1, ARID4A, EZH1, MORF4L2, ARID4B, HIRA, NAP1L1, PTTG1, LATS1, CBX7, EPC1, SMARCD2, PRMT5, RTF1, EED, TLK1, USP16, MLL3, MYST4, MLL2, H1F0, TBL1XR1, SATB2, RBL2, RCOR1, MTA2, RBL1, TP53, NUSAP1, ARID1A, RB1, TAF6L, HMGA2, HLTF, RBBP7, UBE2B, UBE2N, EYA1, EP300, BPTF, RFC1, ASH1L, SMARCA5, PAF1, JMJD1C, CARM1, MAP3K12, SUV420H1, SUPT6H, ING5, ING3, NEK2, SOX2, HAT1, CDC73, NR3C1, TCF7L1, CHD9, NIPBL, SET, TSPYL2, NPM1, CHD1, TAF9, ACTL6A, ASF1A, CHD5, BAZ2A, BUB3, APC, DNMT3A, KAT2B, MLL, MSH3, TAF5, NASP, L3MBTL, SMYD3, CDC23, SIRT1, ATXN7L3, CTR9, TNKS1BP1, RPS6KA5, HDAC4, PHF1, HDAC1, SMARCC1, RNF2, PHF15, H2AFY2, H3F3B, RBM14, HDAC9, UBE2E1

GO:0016265~death	130	7.12	1.13E-08	4.66E-05	MEF2C, CADM1, ZAK, TBP, PMAIP1, FOXO3, SHB, TOP1, MAP3K5, APP, CDKN2A, ATG5, GATA6, TARDBP, FAS, RNF34, CUL1, SGPL1, MAGI3, SLC33A1, IL24, STK4, EP300, IFNB1, F3, TNFAIP8, PDCD6IP, TNFAIP3, EIF2AK3, MCL1, LITAF, ERBB3, CLU, STK17B, NFKBIA, AKAP13, RRAGA, BCL2L2, PEA15, ECE1, KRAS, MTCH1, TMEM123, FAIM, DYRK2, AXIN2, SRGN, TRAF4, CARD8, MLL, ABR, TAOK2, KLF11, NR4A2, RYBP, FXR1, VDAC1, P2RX7, SYNE1, VCP, RABEP1, RHOT1, SPTBN2, PDCD6, E2F1, PPARD, SGPP1, DEDD, FASTK, ZMAT3, GJA1, PAWR, DAXX, PTEN, MCF2L, NISCH, SLK, GSN, CXCR4, CASP7, CASP8, SPG20, CASP2, MYC, ARHGEF3, LTBR, PTGER3, NOL3, TP53, ECT2, ARHGEF11, EIF4G2, ZDHHC16, TNFRSF10B, ZFYVE26, BNIP2, UBC, SH3KBP1, MGEA5, BUB1B, SIAH1, MDM4, MAP3K11, TNFRSF21, APH1A, XIAP, KIAA0196, EGLN3, PPP3R1, TRIB3, HPRT1, TSC22D3, SQSTM1, SOS1, BCL2, CABC1, THBS1, PHLDA2, YWHAB, BIRC3, SIRT1, TP73, ITPR1, ATXN1, MEF2D, ATXN2, BNIP3L, JAK2, TP53INP1, F2R
GO:0046777~protein amino acid autophosphorylation	30	1.64	1.13E-08	4.67E-05	NEK2, ERBB2, ACVR1B, IGF1R, VRK1, CSNK2A1, CLK3, MAP3K3, MAP3K9, ZAP70, LMTK2, CAMK2B, YES1, TGFBR1, NLK, MET, TAOK3, PIM1, STK4, FYN, EIF2S1, DYRK1A, PDGFRA, MAP3K10, JAK2, LRRK2, EIF2AK3, MAP3K13, MAP3K12, MAP3K11

GO:0051094~positive regulation of developmental process	64	3.51	1.33E-08	5.49E-05	PPARD, CDX2, MORF4L2, PPARG, RPS6KB1, ITPKB, FOXO3, JAG1, GLI3, ACVR1B, ZFP91, CCNE1, GATA6, HEY2, RHOA, MKL2, FNDC3B, ADAM9, LIMK1, EOMES, RB1, VASH2, SLIT2, NUMBL, ADRB2, NDEL1, HIF1A, BTG1, F3, NPTN, MAPK9, GNAS, TGFB1I1, SOX2, CLU, BMPR2, SRF, LIF, AGGF1, BCL2, ZAP70, AP3D1, RUNX1, THBS1, APC, B4GALT1, BMP2, MAP2K1, VHL, FOXA1, SMAD5, TGFBR2, SMAD2, PLA2G4A, NOTCH1, P2RX7, TNFSF11, ETS1, AMOT, JAK2, BMP7, HDAC9, BMP6, BMPR1A
GO:0008219~cell death	129	7.07	1.37E-08	5.64E-05	MEF2C, CADM1, ZAK, TBP, PMAIP1, FOXO3, SHB, TOP1, MAP3K5, APP, CDKN2A, ATG5, GATA6, TARDBP, FAS, RNF34, CUL1, SGPL1, MAGI3, SLC33A1, IL24, STK4, EP300, IFNB1, F3, TNFAIP8, PDCD6IP, TNFAIP3, EIF2AK3, MCL1, LITAF, ERBB3, CLU, STK17B, NFKBIA, AKAP13, RRAGA, BCL2L2, PEA15, ECE1, KRAS, MTCH1, TMEM123, FAIM, DYRK2, AXIN2, SRGN, TRAF4, CARD8, MLL, ABR, TAOK2, KLF11, RYBP, FXR1, VDAC1, P2RX7, SYNE1, VCP, RABEP1, RHOT1, SPTBN2, PDCD6, E2F1, PPARD, SGPP1, DEDD, FASTK, ZMAT3, GJA1, PAWR, DAXX, PTEN, MCF2L, NISCH, SLK, GSN, CXCR4, CASP7, CASP8, SPG20, CASP2, MYC, ARHGEF3, LTBR, PTGER3, NOL3, TP53, ECT2, ARHGEF11, EIF4G2, ZDHHC16, TNFRSF10B, ZFYVE26, BNIP2, UBC, SH3KBP1, MGEA5, BUB1B, SIAH1, MDM4, MAP3K11, TNFRSF21, APH1A, XIAP, KIAA0196, EGLN3, PPP3R1, TRIB3, HPRT1, TSC22D3, SQSTM1, SOS1, BCL2, CABC1, THBS1, PHLDA2, YWHAB, BIRC3, SIRT1, TP73, ITPR1, ATXN1, MEF2D, ATXN2, BNIP3L, JAK2, TP53INP1, F2R

GO:0016310~phosphorylation	140	7.67	1.54E-08	6.34E-05	NRBP1, ZAK, STK38, EFNA1, CASK, RPS6KB1, MAP3K7, TOP1, MAP3K5, APP, CSNK2A1, CLK3, AAK1, MAP3K9, GAB1, MAP3K8, TLK1, PRKACB, FRS2, ADAM9, TWF1, CSNK1G1, WNK1, PIM1, MARK4, STK4, WEE1, MARK1, SPAG9, MAPK1, MAP4K5, EIF2S1, MAPK4, MAPK3, DLD, PDGFRA, MAPK9, NEK9, LRRK2, EIF2AK3, LRRK1, NEK2, ERBB3, MAPKAPK5, ERBB2, STK17B, BMPR2, CHEK1, SRC, VRK1, PRKRA, ZAP70, LMTK2, DYRK4, DYRK2, BMP2, TAOK2, MAP2K1, SMAD7, TAOK1, RYK, TGFBR1, TAOK3, MAP2K4, MET, TGFBR2, SMAD2, EPHA5, EPHA4, EPHA7, P2RX7, DYRK1B, DYRK1A, CDC42BPA, SPTBN1, TGFBR3, BMP7, ATP6V0E1, ATP5B, FASTK, PIP5K1B, LATS1, DAXX, ACVR1B, SLK, PKN3, CXCR4, BRD4, AKT3, CHUK, IRAK2, CDC7, SRPK2, LIMK1, PKN2, CDK8, PRKCH, MINK1, CDK6, DGUOK, PRKCE, NDUFA10, CDK4, ATP6V1D, SRPK1, PROK2, PDIK1L, CCND1, MAP3K10, MAP3K14, MAP3K13, MAP3K12, UGP2, MAP3K11, TRIB3, ABI2, AKAP9, TRIB1, IGF1R, MAP3K3, MAP3K2, BCL2, CAMK2B, YES1, THBS1, PIK3R1, FLT1, NLK, AXL, MON2, RPS6KA5, GMFB, RPS6KA3, RPS6KA4, FYN, RPS6KA2, JAK1, JAK2, BMPR1A, F2R
GO:0035295~tube development	54	2.96	2.19E-08	9.02E-05	WNT3A, HOXD13, ZEB2, GLI3, LGR4, ZIC2, MAP3K7, GPC3, CD44, CTGF, GATA6, CXCR4, CASP8, RARB, CYR61, SLIT2, VASP, MYCN, PTHLH, HHEX, EYA1, EP300, VEGFA, PDGFRA, TFAP2A, ZFPM2, MED1, WNT5A, SHROOM3, BMPR2, SRF, HECA, BCL2, TRAF4, B4GALT1, BMP2, FLT1, EPAS1, TGFBR1, FOXA1, TGFBR2, SMAD2, FZD3, NR4A3, FOXP1, FZD6, ATP7A, NOTCH1, SP3, ZIC5, GRLF1, BMP7, NFIB, BMPR1A

GO:0051338~regulation of transferase activity	78	4.27	2.35E-08	9.71E-05	CCNT2, ZAK, EFNA1, PRKAG2, PPARG, ADCY6, ZEB2, PRDX3, MBIP, DAXX, LATS1, PTEN, CCNE2, MAP3K7, CDC42, MAP3K5, APP, CDKN2A, CDKN2C, CXCR4, MAP3K9, GAB1, PRKACB, FRS2, ADAM9, PPP2R1A, RBL2, RBL1, PIM1, RB1, IRS1, PKIA, SPAG9, PROK2, TARBP2, MAP4K5, CCND1, ADRB2, TNFRSF10B, EREG, CCND3, CCND2, MAP3K10, MAP3K13, MAP3K11, CAV1, ERBB2, TRIB3, CHEK1, TRIB1, KRAS, TSPYL2, HEXIM1, MAP3K2, PPP2CA, NPM1, THBS1, PPAP2A, APC, FLT1, MAP2K1, TAOK2, TGFBR1, TGFBR2, MET, TP73, CDC25A, CDC25B, CDKN1C, SH3BP5, CDKN1A, P2RX7, LRP1, DUSP2, JAK2, DUSP8, F2R, VLDLR
GO:0010647~positive regulation of cell communication	71	3.89	3.27E-08	1.35E-04	PPARD, GJA1, SHOC2, ITPKB, ZEB2, JAG1, IL11, CITED2, MAP3K7, ACVR1B, ZFP91, GPC3, CASP8, RHOA, RHOC, LTBR, ESR1, CD40, ECT2, IRS1, UBE2N, HHEX, ZDHHC17, ADRB2, TNFRSF10B, HIF1A, EREG, NCOA3, F3, VEGFA, UBC, SEMA4C, PEBP1, TGFB1I1, CAV1, LITAF, ERBB3, ERBB2, SOX2, SOX4, FKBP1A, SRC, LIF, TMED7, KRAS, ECE1, MAP3K3, SOS1, ZAP70, CC2D1A, THBS1, BMP2, FLT1, TAOK2, BECN1, TGFBR1, TAOK3, EDA2R, FURIN, CDKN1C, P2RX4, NOTCH2, P2RX7, GRIA2, CDKN2AIP, EEF1E1, GOLPH3, JAK2, BMP7, F2R, BMPR1A

GO:0000122~negative regulation of transcription from RNA polymerase II promoter	61	3.34	3.53E-08	1.46E-04	MEF2C, E2F1, PPARA, PPARD, CDX2, NR6A1, PPARG, SPI1, PAWR, ZEB1, TCEAL1, CITED2, EPC1, ZNF148, HEY2, RARB, NR2F2, ZNF281, TBL1XR1, SATB2, STRN3, MTA2, RBL1, MECP2, ZHX2, TP53, LEF1, RB1, RBBP7, PKIA, HHEX, BPTF, ZNF238, ZFPM2, MDM4, SOX2, NR2C1, HEXIM1, BCL6, TCF4, DNMT3A, ZBTB7A, KLF12, VHL, JARID2, SMAD7, FOXA1, KLF11, RYBP, SKI, FOXP1, STAT3, CDKN1C, HDAC4, HOXB4, HDAC1, DR1, RNF2, HDAC9, TBL1X, NCOR2
GO:0048514~blood vessel morphogenesis	52	2.85	3.59E-08	1.48E-04	NRP2, ATP5B, JAG1, PTEN, CITED2, MAP3K7, SHB, ATG5, CXCR4, CTGF, HEY2, CASP8, QKI, SOX17, MKL2, NR2F2, CYR61, PTPRJ, SGPL1, MYH9, SLIT2, PROK2, SH2D2A, HIF1A, EREG, VEGFA, AAMP, ZFPM2, CAV1, TIPARP, ELK3, SRF, ZFP36L1, AGGF1, THBS1, B4GALT1, FLT1, IL8, EPAS1, VHL, SMAD7, TGFBR1, TGFBR2, ITGA4, UBP1, ATP7A, NOTCH1, PKNOX1, ADRA1B, AMOT, POFUT1, PLAU
GO:0030097~hemopoiesis	56	3.07	4.05E-08	1.67E-04	LMO2, WNT3A, PPARG, ITPKB, JAG1, IL15, PRDX3, CBFB, IL11, CDC42, CASP8, FAS, CHUK, SGPL1, RELB, EOMES, TP53, MINK1, DLL1, CDK6, RB1, MYH9, VEGFA, NCOA6, MED1, TIPARP, SOX4, BCL2, BCL11B, PPP3CB, ZAP70, BCL6, RUNX1, TCF3, PIK3R1, APC, EBP, KLF6, MLL, EPAS1, L3MBTL, SMAD5, TGFBR2, ITGA4, FOXP1, ATP7A, HOXB4, NOTCH2, HDAC4, PKNOX1, TNFSF11, SP3, IRF1, TGFBR3, JAK2, HDAC9

GO:0010033~response to organic substance	127	6.96	5.43E-08	2.24E-04	THRA, ADCY6, PDE3B, FOXO1, RPS6KB1, PMAIP1, GOT2, SLC2A8, CD48, HTR1B, CD44, GAB1, PRKACB, FAS, ADAM9, STRN3, IRS1, DDIT3, MAPK1, EP300, HNF4A, PIAS3, SLC25A36, PDGFRA, EIF2AK3, DERL2, CAV1, MCL1, ERBB3, ERBB2, NFKBIA, NEDD8, HSPA1B, SRF, TIMP3, SRC, KRAS, PRKRA, SCARB1, TAF9, ERO1L, TAF2, MAP2K1, TGFBR1, TGFBR2, NR4A2, SMAD2, NR4A3, HDAC4, P2RX4, PLA2G4A, CDKN1A, P2RX7, VCP, EPS8, ADRA1B, TGFBR3, DNAJB1, PTPN1, HDAC9, BMP7, DNAJB4, DNAJB6, HSP90AB1, PPARA, ATP6V0E1, LDLR, PPARG, PRDX3, UQCRFS1, LATS1, PTEN, CCNE1, GSN, CASP8, MYC, FOSL1, CYR61, IRAK2, KCNMA1, PPP2R1A, EGR2, ESR1, LEF1, ADIPOR1, UBE2B, TARBP2, CCND1, BTG2, GNB2, ADM, CCND2, SQLE, GNB1, NCOA6, MGEA5, PEBP1, GNAS, GNB4, CALCOCO2, FKBP1A, C1S, NR3C1, HPRT1, TRIB1, IGF1R, ACSL1, BCL2, PPP2CA, PPP3CB, PPP3CA, THBS1, HSPA8, PIK3R1, TXNIP, KAT2B, MAT2A, STAT3, DUSP4, ZFP106, FYN, HSPA4L, JAK2, RBM14, ABCC5, VLDLR, F2R
GO:0000165~MAPKK K cascade	47	2.58	5.65E-08	2.33E-04	WNT5A, CAV1, ZAK, EFNA1, ITPKB, MBIP, DAXX, TRIB1, MAP3K7, MAP3K5, MAP3K3, CXCR4, MAP3K2, MAP3K9, PPP2CA, RB1CC1, GAB1, THBS1, RAPGEF2, FRS2, ADAM9, PPP2R1A, FLT1, MAP2K1, TAOK2, TGFBR1, TAOK3, MAP2K4, MET, MINK1, MAPK1, SPAG9, PROK2, DUSP4, MAP4K5, P2RX7, CRKL, DUSP2, MAP3K10, MAPK9, JAK2, LRRK2, DUSP8, MAP3K13, MAP3K12, MAP3K11, F2R

GO:0060548~negative regulation of cell death	75	4.11	5.97E-08	2.46E-04	MEF2C, WFS1, MITF, FOXO1, PRDX3, PTEN, CITED2, MAP3K7, ZFP91, ATG5, RHOA, TPT1, FAS, MKL1, CASP2, MYC, NOL3, SLC25A4, PIM1, ESR1, TP53, HBXIP, PROK2, EYA1, BTG2, BNIP2, F3, TNFAIP8, VEGFA, UBC, TNFAIP3, YWHAZ, SYVN1, MCL1, XIAP, PAFAH2, ERBB3, ERBB2, CLU, SOX4, NFKBIA, BCL2L2, HSPA1B, PEA15, IGF1R, KRAS, SQSTM1, BCL2, BCL11B, RB1CC1, NPM1, BCL6, TAF9, FAIM, THBS1, APC, CEBPB, BECN1, VHL, SMAD6, TGFBR1, NR4A2, BIRC3, FURIN, TP73, ATF5, NOTCH2, HSP90B1, NOTCH1, SON, CDKN1A, HDAC1, BNIP3L, APBB2, F2R
GO:0043066~negative regulation of apoptosis	74	4.05	6.41E-08	2.64E-04	MEF2C, WFS1, MITF, FOXO1, PRDX3, PTEN, CITED2, MAP3K7, ZFP91, ATG5, RHOA, TPT1, FAS, MKL1, CASP2, MYC, NOL3, PIM1, ESR1, TP53, HBXIP, PROK2, EYA1, BTG2, BNIP2, F3, TNFAIP8, VEGFA, UBC, TNFAIP3, YWHAZ, SYVN1, MCL1, XIAP, PAFAH2, ERBB3, ERBB2, CLU, SOX4, NFKBIA, BCL2L2, HSPA1B, PEA15, IGF1R, KRAS, SQSTM1, BCL2, BCL11B, RB1CC1, NPM1, BCL6, TAF9, FAIM, THBS1, APC, CEBPB, BECN1, VHL, SMAD6, TGFBR1, NR4A2, BIRC3, FURIN, TP73, ATF5, NOTCH2, HSP90B1, NOTCH1, SON, CDKN1A, HDAC1, BNIP3L, APBB2, F2R

GO:0007167~enzyme linked receptor protein signaling pathway	72	3.95	7.36E-08	3.03E-04	LTBP2, EFNA1, CD8B, SHOC2, FOXO1, PTEN, SLC2A8, MAP3K7, ACVR1B, CTGF, ZFYVE9, ZFYVE16, GAB1, FRS2, ADAM9, PTPRJ, SGPL1, IRS1, SS18, ADRB2, EREG, VEGFA, PDGFRA, TGFB1I1, EIF2AK3, ERBB3, ERBB2, TIPARP, BMPR2, FKBP1A, SRC, LIF, IGF1R, CEP57, SOS1, UBE2D1, PIK3R1, TXNIP, BMP2, PTPRD, FLT1, SMAD7, SMAD6, TGFBR1, NLK, TGFBR2, MET, SMAD5, AXL, SMAD2, SKI, STAT3, EPHA5, EPS15, RPS6KA5, EPHA4, EPHA7, ZFP106, ERBB2IP, EPS8, JAK1, SPTBN1, HBEGF, TGFBR3, JAK2, PTPN1, SMURF1, BAMBI, BMP7, BMPR1A, TOB1, BMP6
GO:0048534~hemopoietic or lymphoid organ development	59	3.23	8.98E-08	3.70E-04	LMO2, WNT3A, PPARG, ITPKB, JAG1, IL15, PRDX3, CBFB, IL11, CDC42, CASP8, FAS, CHUK, SGPL1, RELB, EOMES, TP53, MINK1, DLL1, CDK6, RB1, MYH9, CRKL, VEGFA, NCOA6, MED1, TIPARP, SOX4, BCL2, BCL11B, ZAP70, PPP3CB, BCL6, RUNX1, TCF3, PIK3R1, APC, EBP, KLF6, MLL, EPAS1, JARID2, TGFBR1, L3MBTL, SMAD5, TGFBR2, ITGA4, FOXP1, ATP7A, HOXB4, NOTCH2, HDAC4, PKNOX1, TNFSF11, SP3, IRF1, TGFBR3, JAK2, HDAC9

GO:0045859~regulation of protein kinase activity	72	3.95	1.06E-07	4.35E-04	CCNT2, ZAK, EFNA1, PRKAG2, ADCY6, ZEB2, MBIP, DAXX, LATS1, PTEN, CCNE2, MAP3K7, MAP3K5, APP, CDKN2A, CDKN2C, CXCR4, MAP3K9, GAB1, PRKACB, FRS2, ADAM9, PPP2R1A, PIM1, RB1, PKIA, SPAG9, PROK2, TARBP2, MAP4K5, CCND1, ADRB2, TNFRSF10B, EREG, CCND3, CCND2, MAP3K10, MAP3K13, MAP3K11, CAV1, ERBB2, TRIB3, CHEK1, TRIB1, KRAS, TSPYL2, HEXIM1, MAP3K2, PPP2CA, NPM1, THBS1, PPAP2A, APC, FLT1, MAP2K1, TAOK2, TGFBR1, MET, TGFBR2, TP73, CDC25A, CDC25B, CDKN1C, SH3BP5, P2RX7, CDKN1A, LRP1, DUSP2, JAK2, DUSP8, F2R, VLDLR
GO:0008361~regulation of cell size	50	2.74	1.13E-07	4.66E-04	DCBLD2, DERL2, ATP6V0E1, PPARG, ZFP91, ACVR1B, APP, DAB2, TSPYL2, ARHGAP5, CDKN2A, NDRG3, CDKN2C, PPP2CA, BCL2, RB1CC1, NPM1, TMEM123, BCL6, TAF9, ING1, SERTAD2, PPP2R1A, CGRRF1, LIMK1, TGFBR1, PSRC1, TP53, ADIPOR1, RB1, DDX5, CDK4, VAT1, TP73, CAPRIN2, NOTCH2, CDKN1A, RNF6, EP300, PRDM4, HNF4A, ERBB2IP, BTG1, CDKN2AIP, ADRA1B, AMOT, TGFBR3, HBEGF, APBB2, EMP1

GO:0022402~cell cycle process	104	5.70	1.15E-07	4.75E-04	ZAK, PTTG1, CUL3, CUL2, APP, CDKN2A, CDKN2C, INCENP, TARDBP, STAG2, CUL1, STAG1, CGRRF1, PIM1, MND1, MYH9, MARK4, WEE1, DDIT3, PSMA1, NEK9, STMN1, ANAPC13, NEK2, CHEK1, CCNG2, SESN1, SESN3, PSMB5, NIPBL, NPM1, UBE2D1, TCF3, TAF2, MAP2K1, CDC23, CDKN1C, NOTCH2, PSMC6, CDKN1A, ZNF318, UBE2E1, E2F1, LZTS2, TSG101, LATS1, DAXX, ACVR1B, CCNE1, KIF2C, PSMD5, USP16, MYC, KHDRBS1, CDC7, TP53, NUSAP1, CDK6, RB1, HMGA2, CDK4, EIF4G2, PPM1G, CCND1, EREG, PSME1, CCND2, UBC, BUB1B, MAPRE2, MAPRE1, MDM4, KPNA2, MAPRE3, MAP3K11, PPP6C, CETN3, ZNF655, NUMA1, BCL2, KRT7, NPAT, PPP3CB, PKD2, PKD1, PAFAH1B1, HBP1, CAMK2B, PPP3CA, THBS1, BUB3, APC, EXO1, KAT2B, PDS5A, IL8, TP73, RGS14, CDC25A, CDC25B, PSMD11, APBB2, TP53INP1, DNM2
GO:0043069~negative regulation of programmed cell death	74	4.05	1.15E-07	4.76E-04	MEF2C, WFS1, MITF, FOXO1, PRDX3, PTEN, CITED2, MAP3K7, ZFP91, ATG5, RHOA, TPT1, FAS, MKL1, CASP2, MYC, NOL3, PIM1, ESR1, TP53, HBXIP, PROK2, EYA1, BTG2, BNIP2, F3, TNFAIP8, VEGFA, UBC, TNFAIP3, YWHAZ, SYVN1, MCL1, XIAP, PAFAH2, ERBB3, ERBB2, CLU, SOX4, NFKBIA, BCL2L2, HSPA1B, PEA15, IGF1R, KRAS, SQSTM1, BCL2, BCL11B, RB1CC1, NPM1, BCL6, TAF9, FAIM, THBS1, APC, CEBPB, BECN1, VHL, SMAD6, TGFBR1, NR4A2, BIRC3, FURIN, TP73, ATF5, NOTCH2, HSP90B1, NOTCH1, SON, CDKN1A, HDAC1, BNIP3L, APBB2, F2R

GO:0031399~regulation of protein modification process	64	3.51	1.38E-07	5.68E-04	ZAK, PRKAG2, ZEB2, DAXX, IL11, ZFP91, MAP3K5, CDKN2A, ATG5, MAP3K9, GAB1, RAPGEF4, PDGFD, PSMD5, FAM129A, CUL1, PPP2R1A, PRKCE, UBE2N, PSMA1, MAP4K5, CCND1, PSME1, CCND3, HNF4A, CCND2, PIAS3, UBC, MAP3K10, MGEA5, BUB1B, PEBP1, LRRK2, MAP3K11, CAV1, BMPR2, FKBP1A, PSMB5, LIF, SET, MAP3K2, PPP2CA, BCL2, RB1CC1, UBE2D1, BUB3, BMP2, MLL, SMAD7, TGFBR1, SMAD6, YWHAB, CDC23, TP73, HDAC4, P2RX7, PSMC6, PSMD11, JAK2, SMURF1, VPS28, BMP7, UBE2E1, BMPR1A
GO:0002520~immune system development	61	3.34	1.42E-07	5.86E-04	LMO2, WNT3A, PPARG, ITPKB, JAG1, IL15, PRDX3, CBFB, IL11, CDC42, CASP8, FAS, CHUK, SGPL1, RELB, EOMES, TP53, MINK1, DLL1, CDK6, RB1, MYH9, CRKL, VEGFA, NCOA6, MED1, TIPARP, SOX4, BCL2, BCL11B, ZAP70, PPP3CB, BCL6, RUNX1, TCF3, PIK3R1, APC, EXO1, EBP, KLF6, MLL, MSH3, EPAS1, JARID2, TGFBR1, L3MBTL, SMAD5, TGFBR2, ITGA4, FOXP1, ATP7A, HOXB4, NOTCH2, HDAC4, PKNOX1, TNFSF11, SP3, IRF1, TGFBR3, JAK2, HDAC9
GO:0000082~G1/S transition of mitotic cell cycle	22	1.21	1.89E-07	7.77E-04	CDC7, PPP6C, PIM1, RB1, CDK4, MARK4, CDC25A, CUL3, CCNE1, CUL2, ACVR1B, CCND1, CDKN1A, CDKN2A, CCND2, CDKN2C, BCL2, NPAT, PPP3CB, CAMK2B, PPP3CA, CUL1

GO:0000278~mitotic cell cycle	75	4.11	1.90E-07	7.83E-04	E2F1, LZTS2, PTTG1, LATS1, CUL3, CUL2, KIF2C, ACVR1B, CCNE1, APP, CDKN2A, CDKN2C, TARDBP, INCENP, PSMD5, USP16, STAG2, CUL1, STAG1, CDC7, KHDRBS1, PIM1, NUSAP1, CDK6, RB1, HMGA2, CDK4, MARK4, WEE1, PSMA1, CCND1, PSME1, CCND2, UBC, BUB1B, NEK9, MAPRE2, MAPRE1, STMN1, KPNA2, MAPRE3, MAP3K11, PPP6C, NEK2, ANAPC13, CETN3, CHEK1, CCNG2, PSMB5, NUMA1, NIPBL, BCL2, NPAT, PPP3CB, PAFAH1B1, CAMK2B, PPP3CA, UBE2D1, TCF3, BUB3, APC, TAF2, MAP2K1, PDS5A, CDC23, CDC25A, RGS14, CDC25B, CDKN1C, PSMC6, CDKN1A, PSMD11, RNF2, DNM2, UBE2E1
GO:0001558~regulation of cell growth	47	2.58	3.03E-07	1.25E-03	DCBLD2, DERL2, TSG101, MORF4L2, PPARG, ZFP91, ACVR1B, BLZF1, WISP2, DAB2, TSPYL2, CDKN2A, CD44, NDRG3, CTGF, CDKN2C, PPP2CA, BCL2, BCL6, TAF9, ITCH, ING1, CRIM1, SERTAD2, CYR61, PPP2R1A, CGRRF1, TAOK2, LIMK1, TGFBR1, PSRC1, TP53, ADIPOR1, RB1, TP73, CAPRIN2, ARHGEF11, CDKN1A, RNF6, EP300, PRDM4, HNF4A, BTG1, CDKN2AIP, HBEGF, APBB2, IGFBP5

GO:0051603~proteolysis involved in cellular protein catabolic process	107	5.86	3.74E-07	1.54E-03	PPP2R5C, MYLIP, CNOT4, CUL3, CUL2, USP53, ITCH, FBXO21, RNF34, CUL1, AUP1, ADAM9, UBE2J1, SOCS6, MYH9, BRAP, CLPX, PSMA1, PIAS3, FBXO18, FBXL5, ASB1, TGFB1I1, TNFAIP3, RAD23B, DERL2, SYVN1, ANAPC13, NEDD8, RAB40B, PSMB5, FBXW7, UBE2D2, FBXO44, UBE2D1, SPOP, CDC23, PCNP, ATE1, P2RX7, RNF6, PSMC6, VCP, OTUB1, RNF2, FBXO30, SMURF1, FBXO34, UBE2E1, USPL1, TSG101, SENP5, CASP8, RANBP2, YOD1, PSMD5, USP16, USP14, KLHL20, TBL1XR1, UFD1L, HERC6, UBE2F, HLTF, UBE2B, UBE2N, RFWD2, PJA1, PSME1, MED8, UBC, BUB1B, UBE2W, SIAH1, PCYOX1, USP24, UBE2T, APH1A, USP3, XIAP, UBE3A, UBE3C, MYCBP2, CYLD, UEVLD, SQSTM1, RNF128, NEDD4L, USP34, USP33, FBXW11, USP32, BUB3, TRIP12, SPSB1, VHL, MID1, FEM1C, WSB1, HSP90B1, TOM1L1, KCMF1, PSMD11, WSB2, USP47, USP46, TBL1X

GO:0006915~apoptosis	107	5.86	4.44E-07	1.83E-03	MEF2C, CADM1, PMAIP1, FOXO3, SHB, MAP3K5, APP, CDKN2A, ATG5, GATA6, FAS, RNF34, CUL1, SGPL1, MAGI3, IL24, STK4, EP300, IFNB1, F3, TNFAIP8, PDCD6IP, TNFAIP3, EIF2AK3, LITAF, MCL1, ERBB3, CLU, STK17B, NFKBIA, AKAP13, RRAGA, BCL2L2, PEA15, KRAS, ECE1, MTCH1, FAIM, DYRK2, TRAF4, SRGN, CARD8, MLL, ABR, TAOK2, KLF11, RYBP, FXR1, VDAC1, VCP, RABEP1, RHOT1, PDCD6, E2F1, PPARD, SGPP1, DEDD, ZMAT3, FASTK, GJA1, PAWR, DAXX, PTEN, MCF2L, NISCH, SLK, CXCR4, GSN, CASP7, CASP8, CASP2, MYC, ARHGEF3, LTBR, NOL3, TP53, ECT2, ARHGEF11, ZDHHC16, TNFRSF10B, BNIP2, UBC, SH3KBP1, BUB1B, SIAH1, MDM4, TNFRSF21, APH1A, XIAP, PPP3R1, EGLN3, TRIB3, TSC22D3, SQSTM1, BCL2, SOS1, THBS1, PHLDA2, YWHAB, BIRC3, SIRT1, TP73, MEF2D, BNIP3L, JAK2, TP53INP1, F2R
GO:0016569~covalent chromatin modification	35	1.92	4.45E-07	1.83E-03	MORF4L1, ING5, ING3, HAT1, CDC73, EPC1, PRMT5, RTF1, TAF9, ACTL6A, USP16, BAZ2A, MYST4, DNMT3A, KAT2B, TAF6L, SIRT1, UBE2B, ATXN7L3, CTR9, UBE2N, RPS6KA5, HDAC4, EYA1, EP300, HDAC1, RNF2, PHF15, PAF1, CARM1, HDAC9, RBM14, MAP3K12, UBE2E1, SUV420H1

GO:0044257~cellular protein catabolic process	107	5.86	4.84E-07	1.99E-03	PPP2R5C, MYLIP, CNOT4, CUL3, CUL2, USP53, ITCH, FBXO21, RNF34, CUL1, AUP1, ADAM9, UBE2J1, SOCS6, MYH9, BRAP, CLPX, PSMA1, PIAS3, FBXO18, FBXL5, ASB1, TGFB1I1, TNFAIP3, RAD23B, DERL2, SYVN1, ANAPC13, NEDD8, RAB40B, PSMB5, FBXW7, UBE2D2, FBXO44, UBE2D1, SPOP, CDC23, PCNP, ATE1, P2RX7, RNF6, PSMC6, VCP, OTUB1, RNF2, FBXO30, SMURF1, FBXO34, UBE2E1, USPL1, TSG101, SENP5, CASP8, RANBP2, YOD1, PSMD5, USP16, USP14, KLHL20, TBL1XR1, UFD1L, HERC6, UBE2F, HLTF, UBE2B, UBE2N, RFWD2, PJA1, PSME1, MED8, UBC, BUB1B, UBE2W, SIAH1, PCYOX1, USP24, UBE2T, APH1A, USP3, XIAP, UBE3A, UBE3C, MYCBP2, CYLD, UEVLD, SQSTM1, RNF128, NEDD4L, USP34, USP33, FBXW11, USP32, BUB3, TRIP12, SPSB1, VHL, MID1, FEM1C, WSB1, HSP90B1, TOM1L1, KCMF1, PSMD11, WSB2, USP47, USP46, TBL1X
GO:0006351~transcription, DNA-dependent	62	3.40	4.85E-07	2.00E-03	CCNT2, COPS2, PPARA, E2F3, CDX2, THRA, RSF1, ARID4A, HOXD13, TBP, PTTG1, CBFB, TMF1, MAX, CDKN2A, HSF2, TARDBP, RTF1, FOSL1, MYC, TBPL1, PTGER3, SNAPC2, BRF2, ESR1, TP53, HNF4G, TAF6L, MBD1, DDIT3, ELL2, HIF1A, PRDM4, EREG, NCOA6, ASH1L, SMARCA5, NFE2L1, TGFB1I1, MYNN, MED1, POLR2H, BMPR2, NR3C1, TFAM, NFAT5, BTF3, ETV1, TAF9, NFATC3, TRIP11, TAF2, CEBPB, MLL, EPAS1, TAF5, KLF11, AFF4, CREB5, ETS1, TRPS1, IRF1
GO:0045926~negative regulation of growth	32	1.75	5.05E-07	2.08E-03	DCBLD2, ING5, PPARG, ACVR1B, DAB2, TSPYL2, GPC3, CDKN2A, NDRG3, CDKN2C, BCL2, PPP2CA, BCL6, ING1, SERTAD2, PPP2R1A, CGRRF1, PSRC1, TP53, ADIPOR1, RB1, TP73, CAPRIN2, ATXN2, CDKN1A, ADRB2, RNF6, PRDM4, HNF4A,

					BTG1, CDKN2AIP, APBB2
GO:0012501~programmed cell death	108	5.92	5.20E-07	2.14E-03	MEF2C, CADM1, PMAIP1, FOXO3, TOP1, SHB, MAP3K5, APP, CDKN2A, ATG5, GATA6, FAS, RNF34, CUL1, SGPL1, MAGI3, IL24, STK4, EP300, IFNB1, F3, TNFAIP8, PDCD6IP, TNFAIP3, EIF2AK3, LITAF, MCL1, ERBB3, CLU, STK17B, NFKBIA, AKAP13, RRAGA, BCL2L2, PEA15, KRAS, ECE1, MTCH1, FAIM, DYRK2, TRAF4, SRGN, CARD8, MLL, ABR, TAOK2, KLF11, RYBP, FXR1, VDAC1, VCP, RABEP1, RHOT1, PDCD6, E2F1, PPARD, SGPP1, DEDD, ZMAT3, FASTK, GJA1, PAWR, DAXX, PTEN, MCF2L, NISCH, SLK, GSN, CXCR4, CASP7, CASP8, CASP2, MYC, ARHGEF3, LTBR, NOL3, TP53, ECT2, ARHGEF11, ZDHHC16, TNFRSF10B, BNIP2, UBC, SH3KBP1, BUB1B, SIAH1, MDM4, TNFRSF21, APH1A, XIAP, PPP3R1, EGLN3, TRIB3, TSC22D3, SQSTM1, BCL2, SOS1, THBS1, PHLDA2, YWHAB, BIRC3, SIRT1, TP73, MEF2D, BNIP3L, JAK2, TP53INP1, F2R
GO:0016570~histone modification	34	1.86	6.16E-07	2.54E-03	MORF4L1, ING5, ING3, HAT1, CDC73, EPC1, PRMT5, RTF1, TAF9, ACTL6A, USP16, BAZ2A, MYST4, KAT2B, TAF6L, SIRT1, UBE2B, ATXN7L3, CTR9, UBE2N, RPS6KA5, HDAC4, EYA1, EP300, HDAC1, RNF2, PHF15, PAF1, CARM1, HDAC9, RBM14, MAP3K12, UBE2E1, SUV420H1

GO:0051270~regulation of cell motion	46	2.52	6.73E-07	2.77E-03	ENPP2, RPS6KB1, ABHD2, JAG1, TPM1, PTEN, CITED2, VCL, TRIB1, IGF1R, ARHGAP5, NISCH, CXCR4, BCL2, GAB1, RRAS, BCL6, SCARB1, THBS1, PIK3R1, ADAM9, APC, B4GALT1, PARD6B, FLT1, MAP2K1, SMAD7, TGFBR1, FURIN, IRS1, MAPK1, SPAG9, LAMA3, HIF1A, ETS1, F3, VEGFA, PDGFRA, AAMP, AMOT, TGFBR3, HBEGF, JAK2, HDAC9, IGFBP5, F2R
GO:0030334~regulation of cell migration	42	2.30	6.94E-07	2.86E-03	ENPP2, RPS6KB1, ABHD2, JAG1, TPM1, PTEN, CITED2, VCL, TRIB1, IGF1R, ARHGAP5, NISCH, CXCR4, BCL2, GAB1, RRAS, SCARB1, THBS1, PIK3R1, ADAM9, APC, PARD6B, FLT1, MAP2K1, SMAD7, FURIN, IRS1, MAPK1, SPAG9, LAMA3, HIF1A, F3, VEGFA, PDGFRA, AAMP, AMOT, TGFBR3, HBEGF, JAK2, HDAC9, IGFBP5, F2R
GO:0032583~regulation of gene-specific transcription	36	1.97	7.06E-07	2.91E-03	MEF2C, PPARA, THRA, PPARG, SPI1, NFKBIA, SRF, CITED2, GATA6, HEY2, TAF9, NR2F2, TCF3, TAF2, TBL1XR1, BMP2, IKZF2, SMAD7, PSRC1, TP53, CELSR2, SMAD2, SIRT1, CDKN1C, HDAC4, HHEX, NOTCH1, HIF1A, HNF4A, HDAC1, BPTF, ETS1, HDAC9, TBL1X, MAPRE3, NCOR2

GO:0044265~cellular macromolecule catabolic process	123	6.74	7.24E-07	2.98E-03	PPP2R5C, MYLIP, CNOT4, CUL3, CUL2, USP53, CDKN2A, WIBG, ITCH, RNF34, FBXO21, CUL1, AUP1, ADAM9, ZHX2, UBE2J1, SOCS6, MYH9, BRAP, CLPX, PSMA1, PIAS3, FBXO18, FBXL5, ASB1, TGFB1I1, TNFAIP3, EIF2C3, EIF2C4, RAD23B, DERL2, SYVN1, ANAPC13, NEDD8, HSPA1B, RAB40B, PSMB5, FBXW7, UBE2D2, FBXO44, UBE2D1, SPOP, SMG7, CDC23, PCNP, CASC3, ATE1, P2RX7, RNF6, PSMC6, UPF3B, VCP, OTUB1, RNF2, DCP1A, FBXO30, SMURF1, FBXO34, UBE2E1, USPL1, LDLR, TSG101, SENP5, CASP8, PSMD5, YOD1, RANBP2, USP16, MYC, USP14, KLHL20, TBL1XR1, UFD1L, HERC6, UBE2F, MBD4, HLTF, UBE2B, UBE2N, RFWD2, PJA1, PSME1, MED8, UBC, BUB1B, UBE2W, SIAH1, PCYOX1, USP24, UBE2T, APH1A, USP3, XIAP, UBE3A, RNH1, UBE3C, MYCBP2, ZFP36L1, EXOSC10, CYLD, UEVLD, SQSTM1, RNF128, NEDD4L, USP34, FBXW11, USP33, USP32, BUB3, TRIP12, SPSB1, VHL, MID1, FEM1C, WSB1, HSP90B1, KCMF1, TOM1L1, PSMD11, WSB2, USP47, USP46, TBL1X
GO:0032774~RNA biosynthetic process	62	3.40	7.90E-07	3.25E-03	CCNT2, COPS2, PPARA, E2F3, CDX2, THRA, RSF1, ARID4A, HOXD13, TBP, PTTG1, CBFB, TMF1, MAX, CDKN2A, HSF2, TARDBP, RTF1, FOSL1, MYC, TBPL1, PTGER3, SNAPC2, BRF2, ESR1, TP53, HNF4G, TAF6L, MBD1, DDIT3, ELL2, HIF1A, PRDM4, EREG, NCOA6, ASH1L, SMARCA5, NFE2L1, TGFB1I1, MYNN, MED1, POLR2H, BMPR2, NR3C1, TFAM, NFAT5, BTF3, ETV1, TAF9, NFATC3, TRIP11, TAF2, CEBPB, MLL, EPAS1, TAF5, KLF11, AFF4, CREB5, ETS1, TRPS1, IRF1

GO:0009719~response to endogenous stimulus	78	4.27	8.94E-07	3.68E-03	PPARA, ATP6V0E1, THRA, LDLR, PPARG, ADCY6, PDE3B, FOXO1, RPS6KB1, UQCRFS1, LATS1, PTEN, SLC2A8, CCNE1, HTR1B, GAB1, PRKACB, FAS, FOSL1, ADAM9, KCNMA1, EGR2, STRN3, ESR1, ADIPOR1, UBE2B, IRS1, DDIT3, MAPK1, CCND1, EP300, BTG2, ADM, GNB2, CCND2, PIAS3, GNB1, SLC25A36, NCOA6, MGEA5, PDGFRA, PEBP1, GNAS, GNB4, CAV1, ERBB3, ERBB2, HPRT1, SRF, TIMP3, SRC, IGF1R, KRAS, BCL2, PPP3CA, THBS1, PIK3R1, TXNIP, KAT2B, MAP2K1, MAT2A, TGFBR1, TGFBR2, NR4A2, NR4A3, STAT3, PLA2G4A, CDKN1A, ZFP106, ADRA1B, TGFBR3, JAK2, PTPN1, RBM14, BMP7, HDAC9, ABCC5, VLDLR
GO:0032535~regulation of cellular component size	58	3.18	9.09E-07	3.74E-03	ATP6V0E1, LIMA1, PPARG, LATS1, ACVR1B, ZFP91, APP, DAB2, ARHGAP5, CDKN2A, CDKN2C, GSN, SERTAD2, PPP2R1A, CGRRF1, LIMK1, TP53, ADIPOR1, RB1, CDK4, VAT1, ARPC1A, EP300, PRDM4, HNF4A, BTG1, ARPC5L, EMP1, DCBLD2, DERL2, PFN2, TSPYL2, NDRG3, PPP2CA, BCL2, RB1CC1, TMEM123, NPM1, BCL6, TAF9, ING1, TGFBR1, PSRC1, DDX5, TP73, CAPRIN2, NOTCH2, RNF6, CDKN1A, ERBB2IP, CDKN2AIP, SPTBN2, ADRA1B, AMOT, TGFBR3, HBEGF, SPTBN1, APBB2
GO:0006605~protein targeting	49	2.68	1.11E-06	4.58E-03	YWHAZ, LTBP2, TIMM17A, MTX2, TIMM17B, AKAP12, NFKBIA, GLI3, SSR1, CRY2, PEX19, CEP57, ZFYVE9, ZFYVE16, PPP3CB, BCL6, PEX13, PPP3CA, RANBP2, NUP54, TPR, TNPO1, TRAM1, TOMM34, TAOK2, YWHAB, TP53, PPP1R10, ICMT, MAPK1, ERBB2IP, IPO7, TRPS1, YWHAQ, AKAP5, MGEA5, SPTBN1, SDCBP, JAK2, SMURF1, KPNA4, KPNA3, KPNA2, SSR2, SRP9, KPNA1, TOB1, F2R, SSR3

GO:0007507~heart development	49	2.68	1.11E-06	4.58E-03	NRP2, WNT3A, GJA1, PTEN, GLI3, CITED2, ATG5, GATA6, CASP7, CASP8, RARB, MKL2, PTPRJ, HHEX, CRKL, EP300, ADM, NCOA6, ZFPM2, MED1, ERBB3, ERBB2, SOX4, FKBP1A, TPM1, SRF, ECE1, HEXIM1, RB1CC1, PKD2, PPP3CB, PKD1, NFATC3, DVL3, BMP2, SMAD7, TGFBR1, TGFBR2, FBN1, SMAD2, ITGA4, FOXP1, COL5A1, NOTCH1, ADRA1B, TGFBR3, HDAC9, POFUT1, BMPR1A
GO:0030308~negative regulation of cell growth	28	1.53	1.14E-06	4.67E-03	DCBLD2, PPARG, ACVR1B, DAB2, TSPYL2, CDKN2A, NDRG3, CDKN2C, BCL2, PPP2CA, BCL6, ING1, SERTAD2, PPP2R1A, CGRRF1, PSRC1, TP53, ADIPOR1, RB1, TP73, CAPRIN2, CDKN1A, RNF6, HNF4A, PRDM4, BTG1, CDKN2AIP, APBB2
GO:0007050~cell cycle arrest	30	1.64	1.19E-06	4.91E-03	ZAK, TSG101, SESN1, SESN3, CUL3, CUL2, CDKN2A, CDKN2C, PKD2, PKD1, HBP1, THBS1, MYC, CUL1, APC, KHDRBS1, CGRRF1, KAT2B, IL8, TP53, RB1, DDIT3, TP73, CDKN1C, NOTCH2, EIF4G2, PPM1G, CDKN1A, APBB2, TP53INP1
GO:0009725~response to hormone stimulus	72	3.95	1.23E-06	5.07E-03	PPARA, THRA, LDLR, PPARG, ADCY6, PDE3B, FOXO1, RPS6KB1, UQCRFS1, PTEN, LATS1, SLC2A8, CCNE1, HTR1B, GAB1, PRKACB, FAS, FOSL1, ADAM9, KCNMA1, EGR2, STRN3, ESR1, ADIPOR1, UBE2B, IRS1, MAPK1, CCND1, EP300, BTG2, ADM, GNB2, CCND2, PIAS3, GNB1, SLC25A36, NCOA6, MGEA5, PDGFRA, PEBP1, GNB4, GNAS, CAV1, ERBB3, ERBB2, SRF, TIMP3, SRC, IGF1R, KRAS, BCL2, THBS1, PIK3R1, TXNIP, KAT2B, MAP2K1, MAT2A, TGFBR1, TGFBR2, NR4A3, STAT3, PLA2G4A, ZFP106, CDKN1A, TGFBR3, JAK2, PTPN1, RBM14, HDAC9, BMP7, ABCC5, VLDLR

GO:0030163~protein catabolic process	108	5.92	1.24E-06	5.11E-03	PPP2R5C, MYLIP, CNOT4, CUL3, CUL2, USP53, ITCH, FBXO21, RNF34, CUL1, AUP1, ADAM9, UBE2J1, SOCS6, MYH9, BRAP, CLPX, PSMA1, PIAS3, FBXO18, FBXL5, ASB1, YME1L1, TGFB1I1, TNFAIP3, RAD23B, DERL2, SYVN1, ANAPC13, NEDD8, RAB40B, PSMB5, FBXW7, UBE2D2, FBXO44, UBE2D1, SPOP, CDC23, PCNP, ATE1, P2RX7, RNF6, PSMC6, VCP, OTUB1, RNF2, FBXO30, SMURF1, FBXO34, UBE2E1, USPL1, TSG101, SENP5, CASP8, RANBP2, YOD1, PSMD5, USP16, USP14, KLHL20, TBL1XR1, UFD1L, HERC6, UBE2F, HLTF, UBE2B, UBE2N, RFWD2, PJA1, PSME1, MED8, UBC, BUB1B, UBE2W, SIAH1, PCYOX1, USP24, UBE2T, APH1A, USP3, XIAP, UBE3A, UBE3C, MYCBP2, CYLD, UEVLD, SQSTM1, RNF128, NEDD4L, USP34, USP33, FBXW11, USP32, BUB3, TRIP12, SPSB1, VHL, MID1, FEM1C, WSB1, HSP90B1, TOM1L1, KCMF1, PSMD11, WSB2, USP47, USP46, TBL1X
GO:0040012~regulation of locomotion	45	2.47	1.47E-06	6.05E-03	ENPP2, RPS6KB1, ABHD2, JAG1, TPM1, PTEN, VCL, CITED2, TRIB1, IGF1R, ARHGAP5, NISCH, CXCR4, BCL2, GAB1, RRAS, SCARB1, THBS1, PIK3R1, ADAM9, APC, PARD6B, FLT1, MAP2K1, IL8, SMAD7, FURIN, IRS1, SLIT2, MAPK1, SPAG9, LAMA3, HIF1A, IFNB1, F3, VEGFA, PDGFRA, AAMP, AMOT, TGFBR3, HBEGF, JAK2, HDAC9, IGFBP5, F2R

GO:0010627~regulation of protein kinase cascade	54	2.96	1.49E-06	6.13E-03	PPARD, ZAK, GJA1, ZEB2, PTEN, DAXX, IL11, MAP3K7, ZFP91, MAP3K5, MAP3K9, GAB1, CASP8, RHOA, RHOC, PPP2R1A, LTBR, CD40, ECT2, UBE2N, ADRB2, MAP4K5, ZDHHC17, TNFRSF10B, HNF4A, F3, MAP3K10, SEMA4C, PEBP1, TNFAIP3, MAP3K11, CAV1, LITAF, ERBB3, ERBB2, FKBP1A, TIMP2, LIF, TMED7, MAP3K3, SQSTM1, MAP3K2, PPP2CA, CC2D1A, APC, CARD8, TAOK2, TGFBR1, TAOK3, EDA2R, TP73, P2RX7, JAK2, F2R
GO:0019941~modification-dependent protein catabolic process	101	5.53	1.56E-06	6.41E-03	PPP2R5C, MYLIP, CNOT4, CUL3, CUL2, USP53, ITCH, FBXO21, RNF34, CUL1, AUP1, UBE2J1, SOCS6, BRAP, PSMA1, PIAS3, FBXO18, FBXL5, ASB1, TGFB1I1, TNFAIP3, RAD23B, DERL2, SYVN1, ANAPC13, NEDD8, RAB40B, PSMB5, FBXW7, UBE2D2, FBXO44, UBE2D1, SPOP, CDC23, PCNP, ATE1, RNF6, PSMC6, VCP, OTUB1, RNF2, FBXO30, SMURF1, FBXO34, UBE2E1, USPL1, TSG101, SENP5, RANBP2, YOD1, PSMD5, USP16, USP14, KLHL20, TBL1XR1, UFD1L, HERC6, UBE2F, HLTF, UBE2B, UBE2N, RFWD2, PJA1, PSME1, MED8, UBC, BUB1B, UBE2W, SIAH1, PCYOX1, USP24, UBE2T, XIAP, USP3, UBE3A, UBE3C, MYCBP2, CYLD, UEVLD, SQSTM1, RNF128, NEDD4L, USP34, USP33, FBXW11, USP32, BUB3, TRIP12, SPSB1, VHL, MID1, FEM1C, WSB1, HSP90B1, TOM1L1, KCMF1, PSMD11, WSB2, USP47, USP46, TBL1X

GO:0043632~modification-dependent macromolecule catabolic process	101	5.53	1.56E-06	6.41E-03	PPP2R5C, MYLIP, CNOT4, CUL3, CUL2, USP53, ITCH, FBXO21, RNF34, CUL1, AUP1, UBE2J1, SOCS6, BRAP, PSMA1, PIAS3, FBXO18, FBXL5, ASB1, TGFB1I1, TNFAIP3, RAD23B, DERL2, SYVN1, ANAPC13, NEDD8, RAB40B, PSMB5, FBXW7, UBE2D2, FBXO44, UBE2D1, SPOP, CDC23, PCNP, ATE1, RNF6, PSMC6, VCP, OTUB1, RNF2, FBXO30, SMURF1, FBXO34, UBE2E1, USPL1, TSG101, SENP5, RANBP2, YOD1, PSMD5, USP16, USP14, KLHL20, TBL1XR1, UFD1L, HERC6, UBE2F, HLTF, UBE2B, UBE2N, RFWD2, PJA1, PSME1, MED8, UBC, BUB1B, UBE2W, SIAH1, PCYOX1, USP24, UBE2T, XIAP, USP3, UBE3A, UBE3C, MYCBP2, CYLD, UEVLD, SQSTM1, RNF128, NEDD4L, USP34, USP33, FBXW11, USP32, BUB3, TRIP12, SPSB1, VHL, MID1, FEM1C, WSB1, HSP90B1, TOM1L1, KCMF1, PSMD11, WSB2, USP47, USP46, TBL1X
GO:0051098~regulation of binding	38	2.08	2.59E-06	1.06E-02	RSF1, THRA, CALD1, NFKBIA, FKBP1A, PRDX3, TRIB1, KRAS, CDKN2A, BCL2, NPM1, BRD4, NR2F2, IRAK2, EPB41, SMAD7, PIM1, RUNX1T1, EDA2R, SKI, SMAD2, TAF6L, SIRT1, LDLRAP1, DDIT3, UBE2N, PTHLH, HDAC4, ADRB2, EP300, NCOA3, HNF4A, TGFBR3, JAK2, EDA, MAP3K13, EIF2AK3, CALM2

GO:0009057~macromolecule catabolic process	128	7.01	2.69E-06	1.10E-02	PPP2R5C, MYLIP, CNOT4, CUL3, CUL2, USP53, CDKN2A, WIBG, ITCH, RNF34, FBXO21, CUL1, AUP1, ADAM9, ZHX2, UBE2J1, SOCS6, MYH9, BRAP, CLPX, GNS, PSMA1, PIAS3, FBXO18, FBXL5, ASB1, YME1L1, TGFB1I1, TNFAIP3, EIF2C3, EIF2C4, RAD23B, DERL2, SYVN1, ANAPC13, NEDD8, HSPA1B, RAB40B, PSMB5, FBXW7, UBE2D2, FBXO44, UBE2D1, SPOP, SMG7, CDC23, PCNP, CASC3, ATE1, PSMC6, P2RX7, RNF6, UPF3B, VCP, OTUB1, RNF2, DCP1A, FBXO30, SMURF1, FBXO34, UBE2E1, USPL1, LDLR, TSG101, SENP5, CASP8, PSMD5, YOD1, RANBP2, USP16, MYC, USP14, KLHL20, TBL1XR1, UFD1L, HERC6, UBE2F, MBD4, HLTF, UBE2B, UBE2N, RFWD2, PJA1, PSME1, MED8, UBC, MGEA5, BUB1B, UBE2W, SIAH1, PCYOX1, USP24, UBE2T, APH1A, USP3, XIAP, UBE3A, RNH1, FKBP1A, UBE3C, MYCBP2, ZFP36L1, EXOSC10, CYLD, UEVLD, SQSTM1, RNF128, NEDD4L, USP34, FBXW11, USP33, USP32, BUB3, TRIP12, SPSB1, VHL, MID1, FUCA1, FEM1C, WSB1, HSP90B1, KCMF1, TOM1L1, PSMD11, WSB2, USP47, USP46, TBL1X

GO:0040008~regulation of growth	67	3.67	2.86E-06	1.17E-02	MORF4L1, TSG101, ZMAT3, MORF4L2, PPARG, RPS6KB1, ACVR1B, ZFP91, EPC1, WISP2, BLZF1, APP, DAB2, CDKN2A, GPC3, CD44, CTGF, CDKN2C, HEY2, ITCH, SERTAD2, CYR61, SGPL1, PPP2R1A, CGRRF1, LIMK1, SOCS6, TP53, ADIPOR1, RB1, HMGA2, TNKS2, ARHGEF11, ADRB2, EP300, PRDM4, HNF4A, BTG1, UBC, DCBLD2, ING5, DERL2, ING3, TSPYL2, NDRG3, PPP2CA, BCL2, BCL6, ACTL6A, TAF9, POU3F2, ING1, CRIM1, TAOK2, TGFBR1, PSRC1, TGFBR2, STAT3, TP73, CAPRIN2, ATXN2, RNF6, CDKN1A, CDKN2AIP, HBEGF, APBB2, IGFBP5
GO:0045597~positive regulation of cell differentiation	50	2.74	3.14E-06	1.29E-02	PPARD, CDX2, PPARG, MORF4L2, ITPKB, FOXO3, JAG1, GLI3, ACVR1B, ZFP91, CCNE1, GATA6, RHOA, FNDC3B, LIMK1, EOMES, RB1, SLIT2, NUMBL, NDEL1, HIF1A, BTG1, NPTN, MAPK9, GNAS, TGFB1I1, CLU, SOX2, BMPR2, SRF, LIF, BCL2, ZAP70, AP3D1, RUNX1, APC, BMP2, MAP2K1, VHL, FOXA1, SMAD5, TGFBR2, SMAD2, NOTCH1, TNFSF11, ETS1, JAK2, BMP7, BMP6, BMPR1A
GO:0002521~leukocyte differentiation	34	1.86	3.45E-06	1.41E-02	PPARG, SOX4, ITPKB, IL15, CBFB, IL11, CDC42, BCL11B, BCL2, CASP8, PPP3CB, ZAP70, BCL6, FAS, TCF3, CHUK, PIK3R1, APC, KLF6, RELB, TGFBR2, EOMES, TP53, MINK1, ITGA4, MYH9, FOXP1, ATP7A, HDAC4, PKNOX1, TNFSF11, SP3, IRF1, HDAC9

GO:0044093~positive regulation of molecular function	101	5.53	4.05E-06	1.66E-02	ZAK, EFNA1, ADCY6, PMAIP1, MAP3K7, MAP3K5, CDKN2A, MAP3K9, GAB1, PRKACB, FRS2, CUL1, ADAM9, STIM2, PIM1, IRS1, PTHLH, PSMA1, SPAG9, MAP4K5, HIF1A, NDEL1, EP300, IFNB1, F3, EDA, EIF2AK3, CAV1, ERBB2, PSMB5, KRAS, NPM1, MTCH1, SCARB1, UBE2D1, GABARAPL2, CARD8, MAP2K1, TAOK2, EPB41, TGFBR1, TGFBR2, MET, NR4A2, CDC23, SKI, HOMER1, ATP7A, HDAC4, P2RX7, PSMC6, VCP, TGFBR3, UBE2E1, PRKAG2, ZEB2, PRDX3, DAXX, CDC42, CXCR4, BRD4, PSMD5, MYC, IRAK2, PTGER3, TP53, UBE2N, PROK2, CCND1, ADRB2, TNFRSF10B, PSME1, EREG, CCND3, NCOA3, CCND2, GNB1, UBC, MAP3K10, GNAS, MAP3K13, MAP3K11, APH1A, CALD1, FKBP1A, TPM1, MAP3K2, BCL2, THBS1, PPAP2A, FLT1, MSH3, YWHAB, EDA2R, CDC25B, LRP1, PSMD11, JAK2, CALM2, VLDLR, F2R
GO:0051272~positive regulation of cell motion	28	1.53	4.34E-06	1.77E-02	RPS6KB1, IGF1R, ARHGAP5, BCL2, BCL6, SCARB1, THBS1, PIK3R1, ADAM9, APC, FLT1, MAP2K1, TGFBR1, FURIN, IRS1, SPAG9, MAPK1, HIF1A, ETS1, F3, VEGFA, PDGFRA, AAMP, HBEGF, AMOT, JAK2, HDAC9, F2R

GO:0008283~cell proliferation	80	4.38	4.46E-06	1.82E-02	E2F1, MORF4L1, COPS2, PPARD, ZAK, WNT3A, NR6A1, ZEB2, ZEB1, IL15, PTEN, LGR4, SHB, KIF2C, BLZF1, DAB2, CXCR4, PRMT5, GAB1, TGFBI, MYC, FRS2, CUL1, IMPDH2, CYR61, DLG1, KHDRBS1, NANOG, SOX11, PIM1, TP53, CD40, RBBP7, NUMBL, HHEX, SH2D2A, PROK2, NDEL1, PRDM4, CCND3, EREG, IFNB1, GNB1, VEGFA, BUB1B, TXNRD1, MAPRE2, MAPRE1, MDM4, BIN1, EMP1, MAP3K11, CDV3, ERBB2, HDGF, BCL2L2, FKBP1A, HPRT1, SRC, BCL2, PAFAH1B1, SCARB1, POU3F2, BUB3, MAP2K1, NASP, MET, FZD3, FURIN, CDC25A, EPS15, HOXB4, P2RX7, LRP1, EPS8, FYN, GOLPH3, ADRA1B, TGFBR3, LRP2
GO:0016055~Wnt receptor signaling pathway	34	1.86	4.92E-06	2.01E-02	WNT5A, LZTS2, WNT3A, MITF, SOX4, TCF7L1, WNT1, CSNK2A1, HBP1, AXIN2, FBXW11, APC, DVL3, TBL1XR1, WNT10B, CSNK1G1, RYK, NLK, LEF1, TLE4, FZD3, CELSR2, MARK4, UBE2B, FZD4, FZD7, FZD6, HHEX, CCND1, DACT1, LRP6, TGFB1I1, TBL1X, LRP5
GO:0045792~negative regulation of cell size	28	1.53	5.35E-06	2.18E-02	DCBLD2, PPARG, ACVR1B, DAB2, TSPYL2, CDKN2A, NDRG3, CDKN2C, BCL2, PPP2CA, BCL6, ING1, SERTAD2, PPP2R1A, CGRRF1, PSRC1, TP53, ADIPOR1, RB1, TP73, CAPRIN2, CDKN1A, RNF6, HNF4A, PRDM4, BTG1, CDKN2AIP, APBB2

GO:0016192~vesicle-mediated transport	99	5.42	5.74E-06	2.34E-02	NRBP1, RAB1A, APP, DAB2, RAPGEF4, SAR1B, SAR1A, RBM12, SCAMP5, SCAMP2, TRIM36, GAPVD1, FNBP1L, VAMP8, LYST, RAB14, VAMP2, BIN1, TRAPPC1, CAV1, GNAI3, CDC42SE1, ABCA1, LMAN1, ARFGEF2, PEX5L, SRC, STX12, KRAS, SCARB1, DOPEY1, AP2M1, RHOBTB3, GABARAPL2, TGFBR2, LIN7B, LIN7C, CORO1C, P2RX7, RABEP1, VCP, ARF3, SPTBN2, GOSR1, CLTA, LDLR, MARCKSL1, ATP5B, SORL1, EPS15L1, CLTC, RAB3IP, BLZF1, AP1S1, AP2B1, AP1S2, GSN, ZFYVE9, ZFYVE16, SEC24C, SEC24D, KDELR1, GOLGA3, SEC23A, STX6, ARL1, STX1A, STXBP3, LDLRAP1, M6PR, KIF1C, ADRB2, IGF2R, ARCN1, SH3KBP1, YWHAZ, EEA1, DTNBP1, TMED2, SQSTM1, AP3D1, THBS1, HSPA8, DNM3, AP4S1, MON2, PREB, LMBR1L, LRP1, GRIA2, AP2A1, RAB34, RAB22A, MCFD2, LRP6, LRP2, DNM2, VLDLR, LRP5
GO:0006366~transcription from RNA polymerase II promoter	50	2.74	5.93E-06	2.42E-02	CCNT2, COPS2, PPARA, E2F3, THRA, CDX2, ARID4A, HOXD13, TBP, PTTG1, CBFB, TMF1, MAX, HSF2, TARDBP, FOSL1, MYC, TBPL1, SNAPC2, HNF4G, MBD1, DDIT3, ELL2, HIF1A, PRDM4, ASH1L, NCOA6, NFE2L1, TGFB1I1, MED1, POLR2H, BMPR2, NR3C1, NFAT5, BTF3, ETV1, TAF9, TRIP11, NFATC3, TAF2, MLL, CEBPB, EPAS1, TAF5, KLF11, AFF4, CREB5, ETS1, TRPS1, IRF1

GO:0006511~ubiquitin-dependent protein catabolic process	51	2.79	7.10E-06	2.88E-02	USPL1, TSG101, PPP2R5C, CUL3, CUL2, USP53, ITCH, PSMD5, USP16, FBXO21, USP14, CUL1, TBL1XR1, UFD1L, UBE2B, PSMA1, PSME1, UBC, BUB1B, SIAH1, TGFB1I1, USP24, RAD23B, DERL2, SYVN1, USP3, UBE3A, NEDD8, PSMB5, CYLD, UBE2D2, SQSTM1, UBE2D1, USP34, USP33, BUB3, USP32, CDC23, PCNP, ATE1, PSMC6, RNF6, HSP90B1, TOM1L1, VCP, PSMD11, USP47, USP46, SMURF1, TBL1X, UBE2E1
GO:0010608~posttranscriptional regulation of gene expression	46	2.52	8.57E-06	3.47E-02	METAP1, SOX4, SYNCRIP, IGF2BP3, PTEN, ZFP36L1, APP, STX12, CDKN2A, WIBG, KRT7, BCL2, PRKRA, QKI, PUM2, SOX17, PABPC1, TNRC6B, FAM129A, THBS1, VHL, ACO1, SMAD7, PAIP1, SMAD2, CASC3, ETF1, CDK4, UBE2B, MAPK1, TARBP2, EIF4G2, ATXN2, EIF4G3, EIF4A2, CDKN2AIP, SERBP1, EIF2S1, VEGFA, MDM4, EIF5A2, EIF2AK3, SRP9, EIF2C3, EIF2C4, IGFBP5
GO:0006928~cell motion	84	4.60	1.08E-05	4.37E-02	NRP2, PPARD, ATP5B, RPS6KB1, ZEB2, MYLIP, PTEN, VCL, WNT1, APP, CD44, CTGF, ANK3, SPINT2, CXCR4, NR2F2, SGPL1, SATB2, EGR2, KIF5C, CCL4L1, MYH9, VASP, SLIT2, CCNL2, PLAUR, ARHGEF11, TNS1, HIF1A, NDEL1, BTG1, LYST, UBC, AAMP, SDCBP, SIAH1, LAMC1, ENPP2, ERBB2, CALD1, ABI2, SRF, TPM1, CCL4, SRC, MYCBP2, TPM3, CCL22, PAFAH1B1, PEX13, SCARB1, POU3F2, THBS1, PPAP2A, APC, ARHGDIB, ACTB, B4GALT1, FLT1, MAP2K1, TAOK2, IL8, VHL, TGFBR1, MET, NR4A2, DPYSL5, ITGA4, COL5A1, STAT3, EPHA4, EPHA7, ETS1, FYN, LRP6, AMOT, HBEGF, TGFBR3, JAK2, APBB2, BMP7, PLAU, FEZ2, LRP5

GO:0048598~embryonic morphogenesis	60	3.29	1.19E-05	4.79E-02	WNT3A, HIRA, HOXD13, ZEB2, ZEB1, GLI3, HOXD10, ZIC2, MAP3K7, CUL3, WNT1, CASP8, RARB, FRS2, MYC, SATB2, TP53, EOMES, LEF1, MBNL1, VASP, SLIT2, HOXC10, EYA1, HOXC11, DLD, TFAP2A, GNAS, TXNRD1, TGFB1I1, MED1, WNT5A, SHROOM3, HOXA13, SOX2, BMPR2, ECE1, PRKRA, HOXA10, POU2F1, FBN2, TGFBR1, TGFBR2, FZD3, SKI, SMAD2, NR4A3, FZD6, HOXB4, NOTCH2, NOTCH1, SP3, RNF2, ZIC5, GRLF1, LRP6, AMOT, BMP7, LRP5, BMPR1A

Table S3-2

Significant Cellular Component terms (adjusted p-value<0.05) enriched by targets of identified miRNA biomarkers.

Term	Count	%	p-value	Adjusted p-value	Genes

GO:0031981~nuclear lumen	275	15.07	1.92E-28	1.23E-25	MEF2C, TGOLN2, CDX2, SYNCRIP, WTAP, EPC1, INTS5, FOXF2, INTS6, GIT2, OGT, TBPL1, CUL1, OXR1, RCOR3, STRN3, YY1, MED11, HNF4G, TAF6L, SFRS2, MAPK1, RFC3, RFC1, HNF4A, PIAS3, RFC2, MAPK3, TGFB1I1, RAD23B, LMNB1, NEK2, NHP2L1, AHCTF1, CHEK1, VRK1, HOXA10, NUP54, TCF4, POLQ, TCF3, SFRS11, DNMT3A, TSEN54, MLL, SMAD7, SMAD6, SMAD5, L3MBTL, RYBP, CDC23, SKI, SMAD2, NXF1, DDX5, FXR1, PAXIP1, RNF6, RNF2, PARP2, TCF12, NCOR2, CCNT2, COPS2, TSG101, ZMAT3, EZH1, GNL3L, ZEB1, SENP5, GLI3, DAXX, RNGTT, CCNE2, CCNE1, TMEM109, ZNF148, QKI, BRD4, TWISTNB, MYB, MYC, CDC7, SRPK2, CTBP2, BRF2, EXOSC2, TP53, MBD4, EXOSC1, RBBP7, MBD1, RBBP6, ARHGEF11, CCND1, MED8, C19ORF2, SH3KBP1, NOL10, CLOCK, TBX19, MED1, POLR2H, CREM, ZNF655, SF3B1, NUMA1, SET, SQSTM1, NPAT, ACTL6A, WAC, CC2D1A, ETV6, BAZ2A, BUB3, RBM25, SSRP1, POLR3F, NLK, LMNA, YWHAB, PHF12, ITPR1, STAT3, RPS6KA5, MEF2D, DUSP4, PAPOLA, RPS6KA3, ZFP106, PKNOX1, ILF2, RPS6KA2, PHF15, RBM14, CALM2, MORF4L1, RNMT, STK38, MORF4L2, SNRPD1, FOXO1, TBP, MBIP, PNN, KLHL7, TOP1, CDKN2A, CSNK2A1, GATA6, PGRMC1, TARDBP, WIBG, RNF34, MYST4, MLL2, SATB2, RBL2, MTA2, RBL1, FMR1, PRKAB2, ARID1A, TOPBP1, PPP1CC, WEE1, ZNF3, ELL2, EP300, HIF1A, SMARCA5, SLU7, PAF1, SNRPC, RAD17, SYVN1, MCL1, SOX2, ELK3, SRF, ORC6L, NPM1, ZNF597, TAF9, RPL5, UBE2D1, TAF2, XPOT, ZMYM2, EPAS1,

TAF5, NR4A1, HEATR1, SSB, CASC3, WHSC2, FOXP1, CTR9, CDKN1C, HDAC4, ATF5, CDKN1A, DNAJB9, HDAC1, VCP, SMARCC1, CDKN2AIP, DYRK1A, SPTBN1, DNAJB1, HDAC9, UBE2E1, E2F1, E2F3, ARID4A, E2F5, DEDD, NR6A1, PRKAG2, ZNF207, GPKOW, TRIM8, CASP7, CASP8, RTF1, TPR, TBL1XR1, ATPAF2, NOL4, NOL3, SOX11, ZFX, CDK8, LEF1, NUSAP1, PRPF3, RB1, CDK4, PRPF4, CCNL2, RFWD2, ADRB2, NCOA6, UBC, ORC5L, KPNA2, KPNA1, ING5, ING3, TRIB3, CDC73, WBP11, NR3C1, EXOSC10, KIAA0020, NPAS2, TSPYL2, POU2F1, POU3F2, ZNF701, ACTB, PDCD11, ADARB1, KAT2B, CEBPB, SIRT1, CDC25A, ATXN7L3, CDC25B, ATXN1, SON, JAK2, TBL1X, NFIB

GO:0005654~nucleoplasm	186	10.19	3.06E-24	1.97E-21	MEF2C, MORF4L1, CDX2, RNMT, STK38, SNRPD1, SYNCRIP, FOXO1, TBP, WTAP, PNN, EPC1, TOP1, CSNK2A1, CDKN2A, INTS5, GATA6, WIBG, FOXF2, INTS6, GIT2, OGT, RNF34, CUL1, MYST4, TBPL1, MLL2, SATB2, RBL2, STRN3, YY1, MTA2, RBL1, PRKAB2, FMR1, MED11, TOPBP1, PPP1CC, TAF6L, WEE1, ELL2, SFRS2, MAPK1, RFC3, EP300, HIF1A, HNF4A, RFC1, PIAS3, RFC2, MAPK3, SMARCA5, SLU7, SNRPC, PAF1, RAD23B, MCL1, SOX2, AHCTF1, CHEK1, ORC6L, NPM1, HOXA10, TAF9, NUP54, UBE2D1, TCF4, POLQ, TCF3, TAF2, XPOT, ZMYM2, MLL, EPAS1, SMAD7, TAF5, SMAD6, SMAD5, L3MBTL, RYBP, CDC23, NR4A1, SKI, SMAD2, NXF1, CASC3, WHSC2, CTR9, HDAC4, ATF5, PAXIP1, CDKN1A, RNF6, HDAC1, RNF2, SMARCC1, CDKN2AIP, DYRK1A, HDAC9, TCF12, PARP2, NCOR2, UBE2E1, E2F1, CCNT2, E2F3, E2F5, ARID4A, NR6A1, PRKAG2, ZEB1, GLI3, DAXX, RNGTT, CCNE2, CCNE1, TRIM8, CASP7, RTF1, TPR, MYC, CDC7, TBL1XR1, ATPAF2, CTBP2, BRF2, CDK8, TP53, LEF1, PRPF3, RB1, RBBP7, CDK4, MBD1, PRPF4, CCNL2, RFWD2, CCND1, MED8, NCOA6, UBC, C19ORF2, ORC5L, KPNA2, CLOCK, KPNA1, MED1, POLR2H, ING5, ING3, CREM, CDC73, WBP11, SF3B1, NUMA1, NPAS2, SET, SQSTM1, NPAT, POU2F1, POU3F2, ACTL6A, WAC, RBM25, ACTB, SSRP1, POLR3F, ADARB1, KAT2B, YWHAB, PHF12, SIRT1, CDC25A, ATXN7L3, CDC25B, RPS6KA5, ATXN1, DUSP4, RPS6KA3, SON, PKNOX1, RPS6KA2, PHF15, TBL1X, RBM14, CALM2

GO:0031974~membrane-enclosed lumen	314	17.21	8.05E-24	5.18E-21	MEF2C, TGOLN2, CDX2, SYNCRIP, WTAP, GOT2, EPC1, APP, INTS5, FOXF2, INTS6, GIT2, OGT, TBPL1, CUL1, OXR1, RCOR3, STRN3, YY1, MED11, HNF4G, TAF6L, SFRS2, MAPK1, RFC3, RFC1, HNF4A, PIAS3, RFC2, MAPK3, VEGFA, DLD, MRPL47, TGFB1I1, MRPL43, MDH2, RAD23B, LMNB1, NEK2, NHP2L1, AHCTF1, CHEK1, CALU, VRK1, HOXA10, NUP54, TCF4, POLQ, TCF3, SFRS11, DNMT3A, TSEN54, MLL, SMAD7, SMAD6, SMAD5, L3MBTL, RYBP, CDC23, SKI, SMAD2, DLAT, NXF1, DDX5, FXR1, VDAC1, HYOU1, PAXIP1, RNF6, RNF2, TCF12, PARP2, NCOR2, CCNT2, COPS2, TSG101, ZMAT3, EZH1, ATP5B, PDIA6, GNL3L, ZEB1, SENP5, GLI3, DAXX, RNGTT, CCNE2, CCNE1, BLZF1, TMEM109, ZNF148, QKI, BRD4, TWISTNB, MYB, MYC, COX17, CDC7, SRPK2, CTBP2, BRF2, EXOSC2, TP53, MBD4, EXOSC1, RBBP7, NDUFA10, MBD1, RBBP6, ARHGEF11, CCND1, MED8, C19ORF2, SH3KBP1, NOL10, CLOCK, TBX19, MED1, POLR2H, CREM, ZNF655, SF3B1, NUMA1, SET, SQSTM1, NPAT, ACTL6A, WAC, CC2D1A, THBS1, ETV6, BAZ2A, PDHX, RBM25, BUB3, POLR3F, SSRP1, SHMT2, NLK, LMNA, YWHAB, PHF12, ITPR1, STAT3, RPS6KA5, DUSP4, MEF2D, PAPOLA, RPS6KA3, ZFP106, PKNOX1, ILF2, RPS6KA2, GOLPH3, PHF15, RBM14, CALM2, MORF4L1, RNMT, STK38, MORF4L2, SNRPD1, FOXO1, TBP, MBIP, PDHB, PNN, KLHL7, TOP1, CDKN2A, CSNK2A1, GATA6, PGRMC1, CPOX, TARDBP, WIBG, LRRC59, RNF34, MYST4, MLL2, SATB2, RBL2, MTA2, RBL1, FMR1, PRKAB2, ARID1A, TOPBP1,

PPP1CC, CLPX, WEE1, ZNF3, ELL2, EP300, HIF1A, MRPS18B, SMARCA5, SLU7, NEU1, PAF1, SNRPC, RAD17, CROT, PCCA, SYVN1, MCL1, SOX2, CLU, ELK3, SRF, KARS, NPM1, ORC6L, ZNF597, TAF9, RPL5, UBE2D1, SRGN, TAF2, XPOT, P4HB, ZMYM2, EPAS1, TAF5, NR4A1, HEATR1, SSB, WHSC2, CASC3, FOXP1, CTR9, CDKN1C, HDAC4, ATF5, CDKN1A, DNAJB9, HDAC1, VCP, CDKN2AIP, SMARCC1, DYRK1A, SPTBN1, DNAJB1, HDAC9, UBE2E1, E2F1, E2F3, ARID4A, E2F5, DEDD, NR6A1, PRKAG2, ZNF207, GPKOW, TRIM8, CASP7, CASP8, RTF1, TPR, TBL1XR1, NOL4, ATPAF2, NOL3, SOX11, ZFX, CDK8, LEF1, NUSAP1, PRPF3, RB1, CDK4, PRPF4, CCNL2, RFWD2, ADRB2, C1QBP, IGF2R, NCOA6, UBC, ORC5L, KPNA2, KPNA1, ING5, ING3, TRIB3, CDC73, WBP11, NR3C1, DCI, EXOSC10, KIAA0020, TFAM, NPAS2, TSPYL2, POU2F1, POU3F2, ZNF701, ACTB, TXNIP, PDCD11, CEBPB, KAT2B, ADARB1, SIRT1, ATXN7L3, CDC25A, CDC25B, ATXN1, SON, HSP90B1, SUMF2, JAK2, TBL1X, NFIB

GO:0070013~intracellular organelle lumen	304	16.66	1.06E-23	6.81E-21	MEF2C, TGOLN2, CDX2, SYNCRIP, WTAP, GOT2, EPC1, INTS5, FOXF2, INTS6, GIT2, OGT, TBPL1, CUL1, OXR1, RCOR3, STRN3, YY1, MED11, HNF4G, TAF6L, SFRS2, MAPK1, RFC3, RFC1, HNF4A, PIAS3, RFC2, MAPK3, DLD, MRPL47, TGFB1I1, MRPL43, MDH2, RAD23B, LMNB1, NEK2, NHP2L1, AHCTF1, CHEK1, CALU, VRK1, HOXA10, NUP54, TCF4, POLQ, TCF3, SFRS11, DNMT3A, TSEN54, MLL, SMAD7, SMAD6, SMAD5, L3MBTL, RYBP, CDC23, SKI, SMAD2, DLAT, NXF1, DDX5, FXR1, VDAC1, HYOU1, PAXIP1, RNF6, RNF2, TCF12, PARP2, NCOR2, CCNT2, COPS2, TSG101, ZMAT3, EZH1, ATP5B, PDIA6, GNL3L, ZEB1, SENP5, GLI3, DAXX, RNGTT, CCNE2, CCNE1, BLZF1, TMEM109, ZNF148, QKI, BRD4, TWISTNB, MYB, MYC, CDC7, SRPK2, CTBP2, BRF2, EXOSC2, TP53, MBD4, EXOSC1, RBBP7, NDUFA10, MBD1, RBBP6, ARHGEF11, CCND1, MED8, C19ORF2, SH3KBP1, NOL10, CLOCK, TBX19, MED1, POLR2H, CREM, ZNF655, SF3B1, NUMA1, SET, SQSTM1, NPAT, ACTL6A, WAC, CC2D1A, ETV6, BAZ2A, PDHX, RBM25, BUB3, POLR3F, SSRP1, SHMT2, NLK, LMNA, YWHAB, PHF12, ITPR1, STAT3, RPS6KA5, DUSP4, MEF2D, PAPOLA, RPS6KA3, ZFP106, PKNOX1, ILF2, RPS6KA2, PHF15, RBM14, CALM2, MORF4L1, RNMT, STK38, MORF4L2, SNRPD1, FOXO1, TBP, MBIP, PDHB, PNN, KLHL7, TOP1, CDKN2A, CSNK2A1, GATA6, PGRMC1, TARDBP, WIBG, LRRC59, RNF34, MYST4, MLL2, SATB2, RBL2, MTA2, RBL1, FMR1, PRKAB2, ARID1A, TOPBP1, PPP1CC, CLPX, WEE1, ZNF3, ELL2, EP300, HIF1A,

					MRPS18B, SMARCA5, SLU7, NEU1, PAF1, SNRPC, RAD17, CROT, PCCA, SYVN1, MCL1, SOX2, ELK3, SRF, KARS, NPM1, ORC6L, ZNF597, TAF9, RPL5, UBE2D1, TAF2, XPOT, P4HB, ZMYM2, EPAS1, TAF5, NR4A1, HEATR1, SSB, WHSC2, CASC3, FOXP1, CTR9, CDKN1C, HDAC4, ATF5, CDKN1A, DNAJB9, HDAC1, VCP, CDKN2AIP, SMARCC1, DYRK1A, SPTBN1, DNAJB1, HDAC9, UBE2E1, E2F1, E2F3, ARID4A, E2F5, DEDD, NR6A1, PRKAG2, ZNF207, GPKOW, TRIM8, CASP7, CASP8, RTF1, TPR, TBL1XR1, NOL4, ATPAF2, NOL3, SOX11, ZFX, CDK8, LEF1, NUSAP1, PRPF3, RB1, CDK4, PRPF4, CCNL2, RFWD2, ADRB2, C1QBP, NCOA6, UBC, ORC5L, KPNA2, KPNA1, ING5, ING3, TRIB3, CDC73, WBP11, NR3C1, DCI, EXOSC10, KIAA0020, TFAM, NPAS2, TSPYL2, POU2F1, POU3F2, ZNF701, ACTB, PDCD11, CEBPB, KAT2B, ADARB1, SIRT1, CDC25A, ATXN7L3, CDC25B, ATXN1, SON, HSP90B1, SUMF2, JAK2, TBL1X, NFIB
GO:0043233~organelle lumen	309	16.93	1.17E-23	7.53E-21	MEF2C, TGOLN2, CDX2, SYNCRIP, WTAP, GOT2, EPC1, APP, INTS5, FOXF2, INTS6, GIT2, OGT, TBPL1, CUL1, OXR1, RCOR3, STRN3, YY1, MED11, HNF4G, TAF6L, SFRS2, MAPK1, RFC3, RFC1, HNF4A, PIAS3, RFC2, MAPK3, VEGFA, DLD, MRPL47, TGFB1I1, MRPL43, MDH2, RAD23B, LMNB1, NEK2, NHP2L1, AHCTF1, CHEK1, CALU, VRK1, HOXA10, NUP54, TCF4, POLQ, TCF3, SFRS11, DNMT3A, TSEN54, MLL, SMAD7, SMAD6, SMAD5, L3MBTL, RYBP, CDC23, SKI, SMAD2, DLAT, NXF1, DDX5, FXR1, VDAC1, HYOU1, PAXIP1, RNF6, RNF2, TCF12, PARP2, NCOR2, CCNT2, COPS2, TSG101,

ZMAT3, EZH1, ATP5B, PDIA6, GNL3L, ZEB1, SENP5, GLI3, DAXX, RNGTT, CCNE2, CCNE1, BLZF1, TMEM109, ZNF148, QKI, BRD4, TWISTNB, MYB, MYC, CDC7, SRPK2, CTBP2, BRF2, EXOSC2, TP53, MBD4, EXOSC1, RBBP7, NDUFA10, MBD1, RBBP6, ARHGEF11, CCND1, MED8, C19ORF2, SH3KBP1, NOL10, CLOCK, TBX19, MED1, POLR2H, CREM, ZNF655, SF3B1, NUMA1, SET, SQSTM1, NPAT, ACTL6A, WAC, CC2D1A, THBS1, ETV6, BAZ2A, PDHX, RBM25, BUB3, POLR3F, SSRP1, SHMT2, NLK, LMNA, YWHAB, PHF12, ITPR1, STAT3, RPS6KA5, DUSP4, MEF2D, PAPOLA, RPS6KA3, ZFP106, PKNOX1, ILF2, RPS6KA2, PHF15, RBM14, CALM2, MORF4L1, RNMT, STK38, MORF4L2, SNRPD1, FOXO1, TBP, MBIP, PDHB, PNN, KLHL7, TOP1, CDKN2A, CSNK2A1, GATA6, PGRMC1, TARDBP, WIBG, LRRC59, RNF34, MYST4, MLL2, SATB2, RBL2, MTA2, RBL1, FMR1, PRKAB2, ARID1A, TOPBP1, PPP1CC, CLPX, WEE1, ZNF3, ELL2, EP300, HIF1A, MRPS18B, SMARCA5, SLU7, NEU1, PAF1, SNRPC, RAD17, CROT, PCCA, SYVN1, MCL1, SOX2, CLU, ELK3, SRF, KARS, NPM1, ORC6L, ZNF597, TAF9, RPL5, UBE2D1, SRGN, TAF2, XPOT, P4HB, ZMYM2, EPAS1, TAF5, NR4A1, HEATR1, SSB, WHSC2, CASC3, FOXP1, CTR9, CDKN1C, HDAC4, ATF5, CDKN1A, DNAJB9, HDAC1, VCP, CDKN2AIP, SMARCC1, DYRK1A, SPTBN1, DNAJB1, HDAC9, UBE2E1, E2F1, E2F3, ARID4A, E2F5, DEDD, NR6A1, PRKAG2, ZNF207, GPKOW, TRIM8, CASP7, CASP8, RTF1, TPR, TBL1XR1, NOL4, ATPAF2, NOL3, SOX11, ZFX, CDK8, LEF1, NUSAP1, PRPF3, RB1, CDK4,

					PRPF4, CCNL2, RFWD2, ADRB2, C1QBP, NCOA6, UBC, ORC5L, KPNA2, KPNA1, ING5, ING3, TRIB3, CDC73, WBP11, NR3C1, DCI, EXOSC10, KIAA0020, TFAM, NPAS2, TSPYL2, POU2F1, POU3F2, ZNF701, ACTB, PDCD11, CEBPB, KAT2B, ADARB1, SIRT1, ATXN7L3, CDC25A, CDC25B, ATXN1, SON, HSP90B1, SUMF2, JAK2, TBL1X, NFIB
GO:0044451~nucleoplasm part	121	6.63	5.68E-17	7.14E-14	MORF4L1, MEF2C, CDX2, STK38, TBP, PNN, EPC1, CSNK2A1, INTS5, GATA6, FOXF2, INTS6, OGT, RNF34, TBPL1, MYST4, MLL2, SATB2, RBL2, MTA2, YY1, RBL1, MED11, TOPBP1, TAF6L, PPP1CC, ELL2, SFRS2, EP300, HIF1A, HNF4A, PIAS3, SLU7, SNRPC, PAF1, SOX2, NPM1, HOXA10, TAF9, TCF4, TCF3, TAF2, ZMYM2, MLL, EPAS1, SMAD7, TAF5, SMAD6, SMAD5, SKI, SMAD2, CASC3, NXF1, CTR9, ATF5, HDAC4, PAXIP1, RNF6, HDAC1, RNF2, DYRK1A, HDAC9, TCF12, NCOR2, E2F1, E2F3, E2F5, ARID4A, NR6A1, ZEB1, DAXX, GLI3, TRIM8, MYC, TBL1XR1, ATPAF2, CTBP2, BRF2, TP53, CDK8, LEF1, PRPF3, RB1, CDK4, RBBP7, PRPF4, MBD1, CCNL2, RFWD2, MED8, NCOA6, C19ORF2, CLOCK, MED1, ING5, POLR2H, ING3, CREM, WBP11, CDC73, SF3B1, NPAS2, NPAT, POU2F1, WAC, ACTL6A, POU3F2, RBM25, ACTB, POLR3F, KAT2B, YWHAB, PHF12, SIRT1, ATXN7L3, ATXN1, SON, PKNOX1, PHF15, TBL1X, RBM14
GO:0005829~cytosol	211	11.56	1.98E-12	1.27E-09	LDHA, RPL13, PDLIM5, SELENBP1, PTTG1, MAP3K7, ZFP91, MAP3K8, GAB1, RAPGEF4, FAS, OGT, CUL1, MYH9, MAPK1, GAPVD1, EIF2S1, MAPK3, RAB14, RPS10, MDH1, PFKFB3, NEK2, NFKBIA, AKAP13, BCL2L2, CACNB3, ARFGEF2,

RPS29, ZAP70, PABPC1, MOCS2, ABR, MAP2K1, SMAD7, SMAD6, TGFBR2, SMAD5, CDC23, SMAD2, ARFIP1, PTPN12, PLA2G4A, PPARG, NBEA, PTEN, MCF2L, CCNE2, CCNE1, AP1S1, AP2B1, AP1S2, NISCH, AASDHPPT, ZFYVE9, SLMAP, GUCY1A3, ARHGEF3, UFD1L, ACO1, INPPL1, HERC6, TP53, DGUOK, LDLRAP1, MTRR, ARHGEF11, CCND1, EPB41L1, ADK, EIF4A2, EIF4A1, C19ORF2, SH3KBP1, MAP3K14, EIF5A2, MAP3K13, MAP3K12, PPP6C, RABGAP1, ABI2, EEA1, FKBP1A, ATP6V1G1, HPRT1, ZFP36L1, NUMA1, SET, ACSL1, KLC1, SQSTM1, SOS1, PPP2CA, BCL2, PPP3CB, PPP3CA, BUB3, MARS, YWHAB, APRT, ZFP106, PKNOX1, TOM1L1, PSMD11, AP2A1, FYN, EEF1E1, GOLPH3, CALM2, OCLN, THRA, CTPS, SNRPD1, CASK, FOXO1, RPS6KB1, FOXO3, VCL, PDE4A, PRKACB, FNDC3A, DARS, PRKAB2, HBXIP, PSMA1, AKAP5, SDCBP, PDCD6IP, CAV1, GNE, SRC, KARS, PSMB5, NPM1, RPL5, UBE2D1, AP2M1, GABARAPL2, ACY1, EPS15, ATP7A, CDKN1A, PLCG1, VCP, HDAC1, SPTBN2, SPTBN1, RAP1A, PTPN1, UBE2E1, PRKAG2, PRDX3, RAB3IP, CDC42, RNF103, GSN, CASP7, CASP8, RANBP2, PLCB1, TUBA1A, CASP2, FOSL1, IMPDH2, CHUK, DLG1, SEC23A, PPP2R1A, NOL3, PFKP, STXBP3, MRPS7, CDK4, ECT2, VASP, PSME1, ARCN1, UBC, BUB1B, TXNRD1, MAPRE1, ALDH9A1, UGP2, KPNA1, XIAP, UBE3A, PPP3R1, AKAP9, NR3C1, TYMS, CEP57, MAP3K2, ODF2, PAFAH1B1, UCK2, YES1, PPAP2A, PIK3R1, HSPA8, APC, ACTB, PDCD11, VHL, XPNPEP1, CDC25B, HSP90B1, RPL13A, GRLF1, JAK2, VPS28, CRK

GO:0043228~non-membrane-bounded organelle	358	19.62	3.81E-12	2.45E-09	TGOLN2, SNCG, DYNC1LI2, CDX2, RPL13, PDLIM5, HIRA, MYLIP, WTAP, CITED2, KIF13A, APP, INCENP, INTS6, MRPL33, STAG2, OXR1, STAG1, H1F0, KIF5B, RCOR3, MPDZ, KIF5C, MECP2, SPIRE1, KRT10, HNF4G, MYH9, MARK4, FARP1, MARK1, MAPK1, RFC3, ZNF238, RFC1, CEP350, RFC2, MAPK3, RPS10, MRPL47, ARL8B, TGFB1I1, MRPL48, BIN1, MRPL43, SUV420H1, ALDOA, LMNB1, NEK2, NHP2L1, AKAP12, DAG1, STK17B, AHCTF1, CHEK1, AKAP11, PEA15, VRK1, NPHP4, PFN2, RPS29, CEP170, CDC42EP3, SFRS11, DNMT3A, TSEN54, KIF3B, MAP2K1, L3MBTL, HOMER1, DDX5, FXR1, VDAC1, CSPP1, TNKS1BP1, SYNE1, EPS8, LASP1, RNF2, TMOD3, MAP4, H3F3B, MAP7, MARCKS, PARP2, NCOR2, CCNT2, COPS2, LZTS2, LIMA1, WASF3, TSG101, ZMAT3, EZH1, ATP5B, GNL3L, SENP5, LATS1, DAXX, GLI3, CCNE1, TMEM109, ZNF148, SLMAP, QKI, BRD4, TWISTNB, MYC, SRPK2, KIAA1949, STX1A, SH3PXD2A, INPPL1, TP53, EXOSC2, MBD4, EXOSC1, RBBP6, ARHGEF11, MYCN, SS18, MYRIP, EPB41L1, SH3KBP1, NOL10, SEMA4C, SGCE, CLOCK, TBX19, SERP1, MAP3K11, POLR2H, YWHAZ, SHROOM3, RABGAP1, CETN3, ABI2, ZNF655, ZNF175, MYCBP2, CYLD, GPHN, NUMA1, KLC1, PPP2CA, SOS1, PKD2, WIPF2, SLC4A7, BCL6, CC2D1A, KIF21A, ETV6, BAZ2A, TRIP11, BUB3, DNM3, SSRP1, SHMT2, PDS5A, NLK, LMNA, PPP1R10, PHF12, MID1, ITPR1, STAT3, MRPL24, MEF2D, PAPOLA, ZFP106, ILF2, GRIA2, RPS6KA2, SVIL, AMOT, RBM14, CALM2, DNM2, MORF4L1, MORF4L2, CASK,

					RPS6KB1, MBIP, CBX7, KLHL2, PNN, VCL, KLHL7, TOP1, KLHL5, CDKN2A, CSNK2A1, ANK3, PGRMC1, TARDBP, WIBG, LRRC59, TPT1, SS18L1, MYST4, NUP133, TWF1, RBL2, MTA2, FMR1, ARID1A, TOPBP1, BICD2, ZNF3, CLPX, TNKS2, ARPC1A, CCDC6, EP300, TNS1, HIF1A, TRIM36, NDEL1, MRPS18B, FNBP1L, LYST, SMARCA5, SDCBP, PDCD6IP, STMN1, EDA, TNFAIP3, RAD17, SYVN1, HMGB3, LITAF, GNE, CDC42SE1, ELK3, SRF, NPM1, ORC6L, ZNF597, RPL5, ARHGDIB, GABARAPL1, MRPS23, PTPN3, EPB41, MYO1B, TAF5, PTPN4, JRKL, WAPAL, HEATR1, SSB, SHANK2, FOXP1, DOCK4, CDKN1C, CORO1C, P2RX4, MYO10, DNAJB9, HDAC1, VCP, CDKN2AIP, SMARCC1, SPTBN2, SPTBN1, DNAJB1, ARID4A, DEDD, ARID4B, RAB3IP, ZNF207, KIF2C, GPKOW, GSN, CAMSAP1, CASP8, RTF1, RHOA, SNTB2, ZYX, TPR, TUBA1A, KLHL20, DLG1, TBL1XR1, PPP2R1A, NOL4, NOL3, SOX11, HMBS, ZFX, LEF1, NUSAP1, DYNLT1, RB1, MRPS7, PALLD, HMGA2, UBE2B, VASP, ARHGAP26, PURA, KIF1C, ADRB2, KIF1B, TBCA, ARPC5L, NCOA6, UBC, PEBP1, BUB1B, ORC5L, MAPRE2, MAPRE1, MAPRE3, ALDH9A1, CNN3, CALD1, CALCOCO2, TRIB3, AKAP9, NR3C1, TPM1, TPM3, EXOSC10, KIAA0020, CHD9, TFAM, TSPYL2, CEP57, KRT7, CHD1, ODF2, PAFAH1B1, APPBP2, ZNF701, CHD5, APC, CENPO, ACTB, PDCD11, ADARB1, CENPQ, PSRC1, DPYSL2, SIRT1, RGS14, CDC25B, ATXN1, RPL13A, H2AFY2, GRLF1, JAK1, JAK2, TBL1X, NFIB, ACTR10
GO:0043232~intracellular	358	19.62	3.81E-12	2.45E-09	TGOLN2, SNCG, DYNC1LI2, CDX2, RPL13, PDLIM5, HIRA,

non-membrane-bounded organelle	MYLIP, WTAP, CITED2, KIF13A, APP, INCENP, INTS6, MRPL33, STAG2, OXR1, STAG1, H1F0, KIF5B, RCOR3, MPDZ, KIF5C, MECP2, SPIRE1, KRT10, HNF4G, MYH9, MARK4, FARP1, MARK1, MAPK1, RFC3, ZNF238, RFC1, CEP350, RFC2, MAPK3, RPS10, MRPL47, ARL8B, TGFB1I1, MRPL48, BIN1, MRPL43, SUV420H1, ALDOA, LMNB1, NEK2, NHP2L1, AKAP12, DAG1, STK17B, AHCTF1, CHEK1, AKAP11, PEA15, VRK1, NPHP4, PFN2, RPS29, CEP170, CDC42EP3, SFRS11, DNMT3A, TSEN54, KIF3B, MAP2K1, L3MBTL, HOMER1, DDX5, FXR1, VDAC1, CSPP1, TNKS1BP1, SYNE1, EPS8, LASP1, RNF2, TMOD3, MAP4, H3F3B, MAP7, MARCKS, PARP2, NCOR2, CCNT2, COPS2, LZTS2, LIMA1, WASF3, TSG101, ZMAT3, EZH1, ATP5B, GNL3L, SENP5, LATS1, DAXX, GLI3, CCNE1, TMEM109, ZNF148, SLMAP, QKI, BRD4, TWISTNB, MYC, SRPK2, KIAA1949, STX1A, SH3PXD2A, INPPL1, TP53, EXOSC2, MBD4, EXOSC1, RBBP6, ARHGEF11, MYCN, SS18, MYRIP, EPB41L1, SH3KBP1, NOL10, SEMA4C, SGCE, CLOCK, TBX19, SERP1, MAP3K11, POLR2H, YWHAZ, SHROOM3, RABGAP1, CETN3, ABI2, ZNF655, ZNF175, MYCBP2, CYLD, GPHN, NUMA1, KLC1, PPP2CA, SOS1, PKD2, WIPF2, SLC4A7, BCL6, CC2D1A, KIF21A, ETV6, BAZ2A, TRIP11, BUB3, DNM3, SSRP1, SHMT2, PDS5A, NLK, LMNA, PPP1R10, PHF12, MID1, ITPR1, STAT3, MRPL24, MEF2D, PAPOLA, ZFP106, ILF2, GRIA2, RPS6KA2, SVIL, AMOT, RBM14, CALM2, DNM2, MORF4L1, MORF4L2, CASK, RPS6KB1, MBIP, CBX7, KLHL2, PNN, VCL, KLHL7, TOP1,

					KLHL5, CDKN2A, CSNK2A1, ANK3, PGRMC1, TARDBP, WIBG, LRRC59, TPT1, SS18L1, MYST4, NUP133, TWF1, RBL2, MTA2, FMR1, ARID1A, TOPBP1, BICD2, ZNF3, CLPX, TNKS2, ARPC1A, CCDC6, EP300, TNS1, HIF1A, TRIM36, NDEL1, MRPS18B, FNBP1L, LYST, SMARCA5, SDCBP, PDCD6IP, STMN1, EDA, TNFAIP3, RAD17, SYVN1, HMGB3, LITAF, GNE, CDC42SE1, ELK3, SRF, NPM1, ORC6L, ZNF597, RPL5, ARHGDIB, GABARAPL1, MRPS23, PTPN3, EPB41, MYO1B, TAF5, PTPN4, JRKL, WAPAL, HEATR1, SSB, SHANK2, FOXP1, DOCK4, CDKN1C, CORO1C, P2RX4, MYO10, DNAJB9, HDAC1, VCP, CDKN2AIP, SMARCC1, SPTBN2, SPTBN1, DNAJB1, ARID4A, DEDD, ARID4B, RAB3IP, ZNF207, KIF2C, GPKOW, GSN, CAMSAP1, CASP8, RTF1, RHOA, SNTB2, ZYX, TPR, TUBA1A, KLHL20, DLG1, TBL1XR1, PPP2R1A, NOL4, NOL3, SOX11, HMBS, ZFX, LEF1, NUSAP1, DYNLT1, RB1, MRPS7, PALLD, HMGA2, UBE2B, VASP, ARHGAP26, PURA, KIF1C, ADRB2, KIF1B, TBCA, ARPC5L, NCOA6, UBC, PEBP1, BUB1B, ORC5L, MAPRE2, MAPRE1, MAPRE3, ALDH9A1, CNN3, CALD1, CALCOCO2, TRIB3, AKAP9, NR3C1, TPM1, TPM3, EXOSC10, KIAA0020, CHD9, TFAM, TSPYL2, CEP57, KRT7, CHD1, ODF2, PAFAH1B1, APPBP2, ZNF701, CHD5, APC, CENPO, ACTB, PDCD11, ADARB1, CENPQ, PSRC1, DPYSL2, SIRT1, RGS14, CDC25B, ATXN1, RPL13A, H2AFY2, GRLF1, JAK1, JAK2, TBL1X, NFIB, ACTR10
GO:0012505~endomembrane system	131	7.18	2.25E-09	1.45E-06	SYT1, TUSC3, SLC9A6, NRBP1, WFS1, LEMD3, WTAP, SSR1, EPC1, DAB2, APP, AAK1, RC3H2, RPN2, ELOVL6, PDGFD,

					NUP35, FNDC3A, FRS2, SCAMP5, NUP133, SGPL1, SCAMP2, SPTLC1, SLC33A1, NUPL1, TNKS2, NDEL1, VAMP8, RAB14, VAMP2, EDA, EIF2AK3, DERL2, CAV1, LMNB1, AHCTF1, LMAN1, ARFGEF2, DULLARD, ENTPD4, NUP54, DOPEY1, ERO1L, TRAM1, AP2M1, XPOT, GABARAPL2, SEC11A, DOCK9, ARFIP1, NXF1, FURIN, EPS15, SYNE1, P2RX7, ATP2A2, GOSR1, SPCS2, CLTA, LDLR, PIP5K1B, GJA1, NBEA, EPS15L1, CLTC, SC4MOL, TMF1, TMEM109, AP2B1, AP1S1, AP1S2, PIGF, CHST12, CASP7, PIGB, RANBP2, TPR, SEC24C, SEC24D, GOLGA3, DLG1, SEC23A, STX1A, PTGER3, PGAP1, CHST2, KTN1, KRTCAP2, SIGMAR1, LDLRAP1, PIGO, SACM1L, KIF1B, SQLE, IPO7, BNIP2, IGF2R, ARCN1, SEMA4C, GNAS, KPNA4, KPNA3, KPNA2, RAB11FIP1, EIF5A2, SRP9, KPNA1, FKBP1A, TMED2, BCL2, PAFAH1B1, CAMK2B, TNPO1, NSDHL, LMNA, AP4S1, MAN1C1, SIRT1, ITPR1, HSP90B1, LRP1, GRIA2, AP2A1, BNIP3L, CYP4F3, LRP2, SSR2, VLDLR, GOLGB1, SSR3
GO:0005730~nucleolus	114	6.25	1.17E-07	7.52E-05	TGOLN2, MORF4L2, MBIP, WTAP, PNN, KLHL7, TOP1, CDKN2A, WIBG, TARDBP, PGRMC1, INTS6, OXR1, RCOR3, MTA2, FMR1, ARID1A, HNF4G, ZNF3, MAPK1, EP300, HIF1A, MAPK3, SMARCA5, RAD17, SYVN1, NHP2L1, NEK2, ELK3, SRF, VRK1, NPM1, ZNF597, RPL5, SFRS11, TSEN54, TAF5, L3MBTL, SSB, HEATR1, DDX5, FOXP1, FXR1, CDKN1C, DNAJB9, VCP, CDKN2AIP, SPTBN1, DNAJB1, PARP2, NCOR2, CCNT2, COPS2, TSG101, DEDD, EZH1, ZMAT3, GNL3L, SENP5, GLI3, ZNF207, TMEM109, GPKOW, ZNF148, RTF1,

					CASP8, QKI, BRD4, TWISTNB, SRPK2, NOL4, NOL3, SOX11, ZFX, TP53, EXOSC2, LEF1, NUSAP1, MBD4, EXOSC1, RBBP6, ARHGEF11, ADRB2, NCOA6, NOL10, SH3KBP1, TBX19, CLOCK, POLR2H, TRIB3, NR3C1, ZNF655, EXOSC10, KIAA0020, TSPYL2, CC2D1A, ETV6, ZNF701, BAZ2A, BUB3, PDCD11, ADARB1, NLK, PHF12, SIRT1, STAT3, ITPR1, ATXN1, MEF2D, PAPOLA, ZFP106, ILF2, RBM14, NFIB
GO:0000267~cell fraction	161	8.82	1.44E-07	9.25E-05	SLC9A6, CADM1, PDLIM5, ADCY6, LEMD3, CASK, SYNCRIP, PDE3B, RPS6KB1, FOXO3, IL15, RAB1A, APP, ANK3, PGRMC1, PDE4A, LRRC59, SERPINE1, SLC2A1, RC3H2, RAPGEF4, FAS, ITCH, NSMAF, FNDC3A, FRS2, RECK, DARS, SLC33A1, MPDZ, FMR1, WNK1, HLA-C, HLA-B, PKIA, IRS1, POR, TNKS2, MAPK1, VAMP8, RAB14, VAMP2, CPD, EDA, LRRK2, EMP1, MDH1, WNT5A, CAV1, GNAI3, DAG1, TCL1A, AKAP13, ABCA1, LMAN1, NAGK, PEX5L, KARS, ECE1, KRAS, SCARB1, ERO1L, GABARAPL2, P4HB, RAB8B, ACY1, MAP2K1, RYK, SEC11A, MET, LIN7B, CELSR3, LIN7C, ICMT, GPR137B, HOMER1, PTPN12, LAMP1, PLA2G4A, P2RX7, EPS8, ATP2A2, VCP, ADRA1B, PTPN1, SPCS2, NRP2, ATP6V0E1, RAB5B, SGPP1, FCRL2, CD151, WISP2, NISCH, SPINT2, CASP7, SNTB2, PLCB1, FOSL1, KDELR1, USP14, STX6, PPP2R1A, STX1A, MPP2, PFKP, TP53, KTN1, SH2D2A, SLC26A6, ADRB2, ADM, SQLE, IPO7, IGF2R, SH3KBP1, PEBP1, GNAS, MAP3K13, MAP3K12, MAP3K11, DEGS1, GALNT3, YWHAZ, PPP4R1, CALD1, CALCOCO2, EEA1, FKBP1A, HPRT1, CDH5, MTMR3, IGF1R, ACSL1, TMED2,

					PPP2CA, BCL2, CLEC2D, PPP3CB, PKD1, PAFAH1B1, PEX13, PPP3CA, ACSL4, PPAP2A, YES1, ACTB, EBP, LMNA, FUCA1, ITPR1, ZFP106, HSP90B1, LRP1, SLC16A6, YWHAQ, LRP6, JAK2, CYP4F3, LRP2, ABCC5, VLDLR, SSR3
GO:0005626~insoluble fraction	130	7.12	2.98E-07	1.91E-04	SLC9A6, CADM1, PDLIM5, ADCY6, PDE3B, CASK, LEMD3, SYNCRIP, RPS6KB1, FOXO3, IL15, RAB1A, APP, ANK3, PDE4A, PGRMC1, LRRC59, SLC2A1, RC3H2, RAPGEF4, ITCH, FNDC3A, FRS2, RECK, SLC33A1, MPDZ, WNK1, HLA-C, HLA-B, IRS1, POR, TNKS2, MAPK1, VAMP8, RAB14, VAMP2, CPD, LRRK2, EDA, EMP1, CAV1, GNAI3, DAG1, AKAP13, TCL1A, ABCA1, LMAN1, PEX5L, ECE1, KRAS, SCARB1, ERO1L, GABARAPL2, P4HB, RAB8B, RYK, SEC11A, MET, LIN7B, CELSR3, LIN7C, ICMT, GPR137B, HOMER1, LAMP1, PLA2G4A, P2RX7, ATP2A2, VCP, EPS8, ADRA1B, PTPN1, SPCS2, NRP2, ATP6V0E1, RAB5B, SGPP1, CD151, NISCH, SNTB2, PLCB1, KDELR1, FOSL1, USP14, STX6, STX1A, MPP2, TP53, KTN1, SLC26A6, ADRB2, SQLE, IGF2R, SH3KBP1, PEBP1, GNAS, MAP3K13, MAP3K12, DEGS1, GALNT3, CALD1, EEA1, FKBP1A, CDH5, MTMR3, IGF1R, ACSL1, TMED2, BCL2, PPP3CB, CLEC2D, PKD1, PAFAH1B1, PEX13, PPP3CA, ACSL4, YES1, PPAP2A, EBP, LMNA, ITPR1, ZFP106, HSP90B1, LRP1, SLC16A6, LRP6, JAK2, CYP4F3, LRP2, ABCC5, SSR3, VLDLR
GO:0005905~coated pit	18	0.99	4.51E-07	2.90E-04	CLTA, LDLR, EPS15L1, CLTC, AP4S1, LDLRAP1, EPS15, DAB2, AP2B1, APP, AP1S1, AP1S2, LRP1, AP2A1, AAK1, LRP2, AP2M1, VLDLR

GO:0005624~membrane fraction	124	6.79	1.02E-06	6.59E-04	SLC9A6, CADM1, PDLIM5, ADCY6, PDE3B, CASK, LEMD3, SYNCRIP, RPS6KB1, FOXO3, IL15, APP, ANK3, PDE4A, PGRMC1, LRRC59, SLC2A1, RC3H2, RAPGEF4, ITCH, FNDC3A, FRS2, RECK, SLC33A1, MPDZ, WNK1, HLA-C, HLA-B, IRS1, POR, TNKS2, VAMP8, RAB14, VAMP2, CPD, LRRK2, EDA, EMP1, CAV1, GNAI3, DAG1, AKAP13, TCL1A, ABCA1, LMAN1, PEX5L, ECE1, KRAS, SCARB1, ERO1L, GABARAPL2, P4HB, RYK, SEC11A, MET, LIN7B, CELSR3, LIN7C, ICMT, GPR137B, HOMER1, LAMP1, PLA2G4A, P2RX7, ATP2A2, VCP, EPS8, ADRA1B, SPCS2, NRP2, ATP6V0E1, RAB5B, SGPP1, CD151, NISCH, SNTB2, PLCB1, KDELR1, FOSL1, USP14, STX6, STX1A, MPP2, KTN1, SLC26A6, ADRB2, SQLE, IGF2R, SH3KBP1, PEBP1, GNAS, MAP3K13, MAP3K12, DEGS1, GALNT3, CALD1, EEA1, FKBP1A, CDH5, MTMR3, IGF1R, ACSL1, TMED2, BCL2, PPP3CB, CLEC2D, PKD1, PAFAH1B1, PEX13, PPP3CA, ACSL4, YES1, PPAP2A, EBP, ITPR1, ZFP106, HSP90B1, LRP1, SLC16A6, LRP6, JAK2, CYP4F3, LRP2, ABCC5, SSR3, VLDLR
GO:0005794~Golgi apparatus	130	7.12	2.38E-06	1.53E-03	TGOLN2, PDE3B, IL15, UXS1, RAB1A, HS2ST1, CUL3, APP, CD44, ANK3, PDGFD, SAR1B, FNDC3A, GOLGA8A, SAR1A, SCAMP5, H1F0, SCAMP2, STRN3, BICD2, GCC2, TNKS2, RND3, GYLTL1B, VAMP8, RAB14, PRDM2, CPD, RAB10, LRRK2, TRAPPC1, CAV1, GNAI3, LITAF, RRAGA, CCDC91, ABCA1, LMAN1, OCRL, ARFGEF2, CALU, SERINC3, ECE1, STX12, ENTPD6, DOPEY1, ENTPD4, B4GALT6, SRGN, B4GALT4, RHOBTB3, B4GALT1, GABARAPL2, GABARAPL1,

					LAPTM4A, B4GALT2, MAP2K1, BECN1, ARFIP1, FURIN, NMT2, ATP7A, NOTCH1, SYNE1, ARF3, YIPF3, GOSR1, CLTA, GJA1, NBEA, CLTC, TMF1, BLZF1, AP1S1, AP1S2, PKN3, ZNF148, CHST12, SLC35D2, GOLGA1, GKAP1, SEC24C, SEC24D, GOLGA3, STX6, SEC23A, ARL1, ZDHHC3, ACO1, CHST2, LDLRAP1, ARHGEF11, SACM1L, KIF1C, ZDHHC17, NCOA3, IGF2R, ARCN1, PEBP1, GNAS, MAPRE1, GALNT3, GALNT1, APH1A, GALNT7, CALCOCO2, AKAP9, SEC14L1, TMED7, PRRG4, TMED2, CEP57, RNF128, AP3D1, PKD1, TRIP11, HS3ST3A1, AP4S1, MAN1C1, ATXN2, RAB30, TOM1L1, GLA, AP2A1, GOLPH3, MCFD2, RAB34, LRP2, GOLGB1, F2R
GO:0016585~chromatin remodeling complex	22	1.21	3.49E-06	2.24E-03	MORF4L1, TBL1XR1, SATB2, RSF1, KAT2B, MTA2, ESR1, ARID1A, RB1, TAF6L, RBBP7, HDAC4, CSNK2A1, SMARCD2, HDAC1, SMARCC1, SMARCA5, ACTL6A, TBL1X, HDAC9, ASF1A, BAZ2A
GO:0005667~transcription factor complex	44	2.41	3.73E-06	2.39E-03	E2F1, E2F3, E2F5, CREM, SOX2, NR6A1, TBP, ZEB1, NPAS2, GATA6, FOXF2, HOXA10, POU2F1, POU3F2, TAF9, TCF4, TCF3, TBPL1, TAF2, SATB2, EPAS1, BRF2, RBL2, TAF5, SMAD7, SMAD6, YY1, RBL1, SMAD5, TP53, LEF1, SMAD2, RB1, CDK4, ATF5, EP300, PKNOX1, HIF1A, HNF4A, NCOA6, RBM14, HDAC9, TCF12, CLOCK
GO:0005802~trans-Golgi network	17	0.93	4.97E-06	3.19E-03	TGOLN2, STX6, ARL1, SCAMP2, BECN1, CHST2, NBEA, ARFGEF2, FURIN, ATP7A, ATXN2, GOLPH3, GOLGA1, GNAS, CPD, LRRK2, SCAMP5
GO:0005856~cytoskeleton	185	10.14	1.61E-05	1.03E-02	SNCG, DYNC1LI2, PDLIM5, CASK, MYLIP, KLHL2, VCL,

					PNN, KIF13A, KLHL5, APP, ANK3, INCENP, INTS6, TPT1, STAG2, H1F0, TWF1, KIF5B, MPDZ, KIF5C, SPIRE1, KRT10, TOPBP1, MYH9, BICD2, MARK4, FARP1, MARK1, TNKS2, ARPC1A, CCDC6, MAPK1, TRIM36, NDEL1, TNS1, CEP350, FNBP1L, LYST, MAPK3, SDCBP, TGFB1I1, ARL8B, STMN1, PDCD6IP, BIN1, EDA, TNFAIP3, ALDOA, LMNB1, LITAF, NEK2, GNE, STK17B, DAG1, AKAP12, CDC42SE1, CHEK1, AKAP11, PEA15, PFN2, NPHP4, CEP170, NPM1, CDC42EP3, ARHGDIB, KIF3B, GABARAPL1, EPB41, MAP2K1, PTPN3, MYO1B, TAF5, PTPN4, HOMER1, SHANK2, CSPP1, CORO1C, P2RX4, SYNE1, MYO10, EPS8, LASP1, SPTBN2, SPTBN1, MAP4, TMOD3, MARCKS, MAP7, LIMA1, LZTS2, WASF3, LATS1, RAB3IP, KIF2C, CCNE1, GSN, SLMAP, CAMSAP1, CASP8, RHOA, SNTB2, ZYX, TUBA1A, MYC, KLHL20, DLG1, PPP2R1A, KIAA1949, TBL1XR1, STX1A, SH3PXD2A, INPPL1, NUSAP1, DYNLT1, RB1, PALLD, ARHGAP26, VASP, KIF1C, SS18, MYRIP, KIF1B, EPB41L1, TBCA, ARPC5L, SH3KBP1, SEMA4C, PEBP1, BUB1B, MAPRE2, SGCE, MAPRE1, MAPRE3, ALDH9A1, MAP3K11, YWHAZ, RABGAP1, SHROOM3, CNN3, CALD1, CETN3, ABI2, CALCOCO2, AKAP9, TPM1, ZNF175, MYCBP2, TPM3, CYLD, GPHN, NUMA1, CEP57, KLC1, SOS1, KRT7, PPP2CA, PKD2, WIPF2, ODF2, PAFAH1B1, APPBP2, KIF21A, TRIP11, ZNF701, APC, ACTB, DNM3, PSRC1, LMNA, DPYSL2, MID1, RGS14, ITPR1, CDC25B, GRIA2, SVIL, GRLF1, AMOT, JAK1, JAK2, TBL1X, CALM2, DNM2, ACTR10
GO:0048471~perinuclear region of	53	2.90	1.75E-05	1.12E-02	GALNT3, SNCG, CYB5R4, GALNT1, CAV1, NRBP1, ERBB2,

cytoplasm					CLU, CALCOCO2, RPS6KB1, GLB1, CYLD, APP, ECE1, SET, AGGF1, PKN3, GSN, PDE4A, RNF128, PRKRA, GOLGA1, RC3H2, PAFAH1B1, SEC24C, SEC24D, STX6, SEC23A, KCNMA1, DNM3, KIF5B, MAP2K1, YWHAB, LMNA, SPIRE1, CASC3, TNKS2, TRIM37, ATP7A, SPAG9, ATXN2, TARBP2, P2RX4, PLA2G4A, HSP90B1, GNB2, BNIP2, SERBP1, ARF3, RAB14, MAP7, ABCC5, DNAJB6
GO:0016604~nuclear body	36	1.97	1.98E-05	1.26E-02	MEF2C, WBP11, DAXX, PNN, SF3B1, TRIM8, NPM1, NPAT, WAC, RNF34, MYC, RBM25, ATPAF2, ZMYM2, TP53, PRPF3, SKI, TOPBP1, RB1, NXF1, CASC3, SIRT1, PRPF4, MBD1, CCNL2, CTR9, SFRS2, RFWD2, RNF6, SON, PIAS3, RNF2, DYRK1A, SLU7, SNRPC, NCOR2
GO:0031982~vesicle	101	5.53	2.41E-05	1.54E-02	TGOLN2, SYT1, CADM1, C14ORF1, SLC2A8, APP, DAB2, DMXL2, CLK3, SLC2A1, ITCH, FNDC3A, SCAMP5, ATG9A, DLL1, SPAG9, TRIM36, FNBP1L, DLD, VEGFA, RAB14, SDCBP, NEU1, PDCD6IP, VAMP2, LRRK2, BIN1, ALDOA, PAM, CAV1, GNAI3, CLU, ABCA1, OCRL, CALU, STX12, SYPL1, ECE1, ENTPD4, SRGN, ARHGDIB, AP2M1, P4HB, BMP2, RAB8B, TAOK2, BECN1, FURIN, ATP7A, LAMP1, NOTCH1, PLA2G4A, YIPF3, PTPN1, BMP7, HSP90AB1, CLTA, LDLR, RAB5B, NAP1L1, GJA1, PDIA6, CLTC, AP2B1, AP1S1, AP1S2, SNTB2, SEC24C, SEC24D, SEC23A, STX6, STX1A, STXBP3, LDLRAP1, ZDHHC17, MYRIP, KIF1B, IGF2R, ARCN1, SEMA4C, SH3KBP1, PEBP1, SIAH1, GNAS, RAB11FIP1, IFT74, YWHAZ, TMED2, KLC1, CAMK2B, THBS1, HSPA8, YWHAB, TMEM168, ITPR1, HSP90B1, LRP1, GRIA2, AP2A1, AMOT,

					LRP2
GO:0017053~transcriptional repressor complex	15	0.82	2.47E-05	1.57E-02	TBL1XR1, CTBP2, CDX2, ARID4A, MTA2, YWHAB, SKI, PHF12, RBBP7, GLI3, HDAC4, CSNK2A1, HDAC1, TBL1X, NCOR2
GO:0031410~cytoplasmic vesicle	97	5.32	3.20E-05	2.04E-02	HSP90AB1, TGOLN2, SYT1, CLTA, LDLR, RAB5B, CADM1, NAP1L1, GJA1, PDIA6, CLTC, C14ORF1, SLC2A8, DAB2, APP, AP2B1, AP1S1, AP1S2, DMXL2, CLK3, SLC2A1, SNTB2, ITCH, SEC24C, FNDC3A, SEC24D, SCAMP5, STX6, SEC23A, STX1A, ATG9A, STXBP3, DLL1, LDLRAP1, SPAG9, ZDHHC17, MYRIP, KIF1B, TRIM36, FNBP1L, IGF2R, ARCN1, DLD, VEGFA, SH3KBP1, SEMA4C, RAB14, SDCBP, PEBP1, NEU1, SIAH1, PDCD6IP, VAMP2, BIN1, LRRK2, RAB11FIP1, IFT74, PAM, YWHAZ, CAV1, GNAI3, CLU, ABCA1, OCRL, CALU, ECE1, STX12, SYPL1, TMED2, KLC1, CAMK2B, ENTPD4, THBS1, HSPA8, SRGN, ARHGDIB, AP2M1, P4HB, RAB8B, TAOK2, BECN1, YWHAB, TMEM168, FURIN, ITPR1, ATP7A, LAMP1, PLA2G4A, HSP90B1, NOTCH1, LRP1, GRIA2, AP2A1, YIPF3, AMOT, PTPN1, LRP2
GO:0030140~trans-Golgi network transport vesicle	11	0.60	5.12E-05	3.24E-02	ATP7A, TGOLN2, AP1S1, CLTA, AP1S2, AP2A1, IGF2R, RAB14, CLTC, FURIN, LDLRAP1
GO:0030133~transport vesicle	19	1.04	5.73E-05	3.62E-02	TGOLN2, SEC23A, SYT1, CLTA, TMEM168, C14ORF1, CLTC, FURIN, LDLRAP1, ATP7A, AP1S1, AP1S2, AP2A1, IGF2R, YIPF3, RAB14, VAMP2, SEC24C, SEC24D

Table S3-3

Significant Molecular Function terms (adjusted p-value<0.05) enriched by targets of identified miRNA biomarkers.

Term	Count	%	p-value	Adjusted p-value	Genes
GO:0030528~transcription regulator activity	266	14.58	3.72E-16	4.12E-13	MEF2C, CDX2, HIRA, HOXD13, PTTG1, MXI1, HOXD10, CITED2, EPC1, FOXF2, RARB, TBPL1, STRN3, YY1, ZHX2, EOMES, MECP2, MED11, HNF4G, TAF6L, SFRS2, ZNF236, ZNF238, RFC1, HNF4A, MTF1, PRDM4, IFNB1, MLLT10, PRDM2, TGFB1I1, SUPT6H, HOXA13, TCF20, HEXIM1, HOXA10, TCF4, TCF3, PLAGL2, PLAG1, IKZF4, KLF6, BMP2, IKZF2, MLL, KLF9, KLF12, SMAD7, SMAD6, SMAD5, L3MBTL, KLF11, RYBP, SKI, SMAD2, EN2, DDX5, UBP1, NOTCH2, NOTCH1, RNF6, RNF4, ETS1, DMTF1, TRPS1, RNF2, TCF12, NCOR2, KLF3, COPS2, PPARA, PPARD, ELF2, TSG101, ZNFX1, PPARG, MITF, SPI1, ZEB2, ZEB1, GLI3, DAXX, CCNE1, BLZF1, HSF2, ZNF148, NR2F2, USP16, MYB, MYC, SERTAD2, ZNF281, CTBP2, SNAPC2, BRF2, TP53, RBBP7, MBD1, ZNF140, MYCN, BRWD1, MED8, ASH1L, C19ORF2, MAP3K10, CLOCK, TBX19, MED1, CREM, NEO1, ZNF175, ZFP36L1, XBP1, BCL2, NPAT, ETV1, BCL6, HBP1, ETV6, BAZ2A, TRIP11, JARID2, AFF4, YWHAB, PPP1R10, AFF1, PHF12, TP73, STAT3, PREB, RLF, MEF2D, PKNOX1, ILF2, RBM14, DNM2, TOB1, THRA, FOXO1, TBP, FOXO3, LASS6, TCEAL1, CBFB, CDKN2A, SMARCD2, GATA6, TARDBP, EED, MYST4, SATB2, MTA2, RELB, ARID1A, DDIT3, ZNF3, ELL2, ZNF193, HHEX, EP300, HIF1A, SMARCA5, TFAP2A, NFE2L1, MYNN, CAMTA1, LITAF, SOX2, SOX4, ELK3, SRF, NR2C1, NPM1,

					BTF3, TAF9, RUNX1, MAFG, EPAS1, TAF5, FOXA1, NR4A2, NR4A1, ICMT, NR4A3, FOXP1, CDKN1C, HDAC4, ATF5, HOXB4, HDAC1, PHF1, DYRK1B, SMARCC1, HDAC9, PDCD6, DNAJB6, E2F1, E2F3, RSF1, ARID4A, E2F5, NR6A1, FOXK2, PAWR, ZNF207, TMF1, MAX, FUBP3, HEY2, MKL2, MKL1, FOSL1, KHDRBS1, TBL1XR1, NANOG, EGR2, FOXJ2, SOX11, SOX12, ZFX, RUNX1T1, ESR1, LEF1, RB1, FOXJ3, HLTF, PURA, HOXC10, HOXC11, NCOA3, BPTF, BTG1, NCOA4, NCOA6, ZNF711, UBC, ZFPM2, CARM1, BTAF1, TRIB3, NR3C1, TCF7L1, TFAM, TSC22D3, NPAS2, TSC22D2, TSC22D4, NFAT5, POU2F1, POU3F2, ING1, NFATC3, ZBTB7A, KAT2B, CEBPB, HMBOX1, CREB5, SIRT1, ATXN7L3, ATXN1, DR1, SP3, IRF1, GRLF1, TBL1X, NFIB
GO:0008134~transcription factor binding	118	6.47	4.08E-15	5.08E-12	CDX2, HIRA, TBP, MXI1, RAB1A, CBFB, CITED2, CDKN2A, SMARCD2, GATA6, TBPL1, MYST4, YY1, RBL1, RELB, MECP2, PIM1, TAF6L, STK4, DDIT3, SFRS2, HHEX, EP300, HIF1A, MTF1, HNF4A, IFNB1, PIAS3, TFAP2A, NFE2L1, TGFB1I1, ENPP2, NFKBIA, ELK3, SRF, TCF20, NPM1, TAF9, TCF4, RUNX1, TCF3, EPAS1, KLF12, RYBP, ICMT, SKI, SMAD2, DDX5, UBP1, ATF5, HDAC4, RNF4, HDAC1, ETS1, SMARCC1, DYRK1B, HDAC9, NCOR2, E2F1, COPS2, PPARD, E2F5, TSG101, PPARG, ZEB1, PAWR, DAXX, TMF1, MAX, CCNE1, DIP2A, HSF2, MKL2, SOX17, NR2F2, MKL1, USP16, SERTAD2, TBL1XR1, CTBP2, ZFX, TP53, RB1, MBD1, PURA, BPTF, NCOA3, NCOA4, BTG1, NCOA6, C19ORF2, MAP3K10, ZFPM2, JMJD1C, CARM1, MED1, YWHAZ, CREM, TRIB3, TRIB1, BCL2, NPAT, TRIP11, TRIP12, PDCD11, KAT2B, VHL, NLK, YWHAB, SIRT1, STAT3, ATXN7L3, DR1, GRLF1, APBB2, TBL1X, RBM14,

					TOB1
GO:0019899~enzyme binding	117	6.41	4.44E-14	5.49E-11	WFS1, PDLIM5, PDE3B, FOXO3, CUL3, CUL2, CDKN2A, DMXL2, CDKN2C, SERPINE1, SLC2A1, LUC7L2, FAS, CUL1, ADAM9, STRN3, PRKAB2, CD40, PPP1CC, IRS1, PLAUR, SPAG9, MAPK1, HIF1A, PIAS3, F3, AKAP5, BIN1, AKAP1, AKAP2, CAV1, NEK2, ERBB2, NFKBIA, CHEK1, AKAP11, ABCA1, PEX5L, NR2C1, AXIN2, RHOBTB3, GABARAPL2, ZMYM2, EPAS1, MAP2K1, TAOK1, SMAD7, TGFBR1, TGFBR2, SMG7, DOCK9, SMAD2, SKI, FURIN, DOCK4, TNKS1BP1, HDAC4, HDAC1, RAP1A, MARCKS, HDAC9, PRKAG2, SHOC2, NBEA, PAWR, PRDX3, LATS1, RAB3IP, ACVR1B, RANBP2, DOCK10, PLCB1, CASP2, DLG1, TP53, PRKCH, RB1, PRKCE, ECT2, UBE2B, MYRIP, CCND1, ADRB2, CCND3, CCND2, IPO7, BTG1, IGF2R, PEBP1, KPNA2, MAP3K13, MAP3K12, MAP3K11, TRIB3, ABI2, ATP6V1G1, CDH5, TRIB1, IGF1R, SQSTM1, BCL2, PKD2, PAFAH1B1, PIK3R1, APC, ACTB, ZBTB7A, KAT2B, NLK, YWHAB, PPP1R10, STAT3, RPS6KA4, DUSP2, LRP1, JAK2, DNM2
GO:0016563~transcription activator activity	98	5.37	9.88E-14	1.22E-10	THRA, FOXO1, FOXO3, CBFB, CITED2, EPC1, SMARCD2, GATA6, FOXF2, RARB, TBPL1, MYST4, MTA2, YY1, EOMES, ARID1A, HNF4G, TAF6L, EP300, MTF1, HNF4A, SMARCA5, TFAP2A, TGFB1I1, TCF20, NPM1, TAF9, RUNX1, TCF3, KLF6, IKZF2, EPAS1, SMAD5, NR4A2, NR4A1, SMAD2, ICMT, NR4A3, CDKN1C, HDAC4, RNF6, NOTCH1, RNF4, HDAC1, SMARCC1, DYRK1B, TCF12, E2F1, PPARA, E2F3, PPARD, ELF2, RSF1, TSG101, PPARG, MITF, ZEB1, MAX, CCNE1, FUBP3, ZNF148, HSF2, MKL2, MYB, MKL1, USP16, FOSL1, SERTAD2, ZNF281, TBL1XR1, FOXJ2, ZFX, LEF1, RB1,

					HLTF, NCOA3, NCOA4, NCOA6, CARM1, MED1, CREM, BCL2, POU2F1, NPAT, ING1, TRIP11, CEBPB, KAT2B, AFF1, ATXN7L3, STAT3, PREB, MEF2D, SP3, RBM14, TBL1X, DNM2, NFIB
GO:0016564~transcription repressor activity	79	4.33	2.90E-12	3.58E-09	E2F1, COPS2, PPARD, CDX2, ELF2, THRA, RSF1, ARID4A, TSG101, PPARG, HIRA, PAWR, ZEB1, MXI1, DAXX, CITED2, EPC1, ZNF148, EED, NR2F2, MYST4, KHDRBS1, ZNF281, TBL1XR1, CTBP2, MTA2, YY1, STRN3, RELB, MECP2, RUNX1T1, RB1, RBBP7, MBD1, DDIT3, SFRS2, HHEX, RFC1, IFNB1, MAP3K10, C19ORF2, ZFPM2, TRIB3, ELK3, NR2C1, HEXIM1, TSC22D4, NPAT, POU2F1, BCL6, TCF4, IKZF4, ZBTB7A, BMP2, KLF12, JARID2, L3MBTL, RYBP, YWHAB, SKI, PHF12, UBP1, SIRT1, FOXP1, CDKN1C, ATXN1, ATF5, HOXB4, HDAC4, DR1, SP3, TRPS1, RNF2, GRLF1, TBL1X, HDAC9, NCOR2, DNAJB6, TOB1
GO:0004672~protein kinase activity	122	6.68	2.51E-11	3.10E-08	NRBP1, LTBP1, ZAK, STK38, CASK, RPS6KB1, ITPKA, MAP3K7, MAP3K5, CSNK2A1, CLK3, AAK1, MAP3K9, MAP3K8, TLK1, PRKACB, TWF1, CSNK1G1, WNK1, PIM1, CCL4L1, MARK4, IRS1, STK4, WEE1, MARK1, MAPK1, MAP4K5, MAPK4, MAPK3, PDGFRA, MAPK9, NEK9, LRRK2, LRRK1, EIF2AK3, ERBB3, NEK2, MAPKAPK5, ERBB2, STK17B, BMPR2, AKAP13, CHEK1, CCL4, SRC, VRK1, ZAP70, LMTK2, DYRK4, DYRK2, TAOK2, MAP2K1, RYK, TAOK1, TGFBR1, TAOK3, TGFBR2, MET, MAP2K4, EPHA5, EPHA4, EPHA7, DYRK1B, DYRK1A, CDC42BPA, TGFBR3, NRP2, FASTK, PRKAG2, LATS1, ACVR1B, SLK, PKN3, AKT3, CHUK, IRAK2, CDC7, SRPK2, LIMK1, CDK8, PKN2, PRKCH, MINK1, CDK6, PRKCE, CDK4, SRPK1, PDIK1L, CCND1, CRKL, CCND3, IGF2R, MAP3K10, BUB1B, MAP3K14, MAP3K13, MAP3K12, MAP3K11,

					POLR2H, TRIB3, FKBP1A, AKAP9, TRIB1, EXOSC10, IGF1R, MAP3K3, MAP3K2, CAMK2B, YES1, CRIM1, FLT1, NLK, AXL, RPS6KA5, RPS6KA3, RPS6KA4, RPS6KA2, FYN, JAK1, JAK2, BMPR1A
GO:0004674~protein serine/threonine kinase activity	92	5.04	3.83E-10	4.74E-07	LTBP1, STK38, ZAK, FASTK, PRKAG2, CASK, RPS6KB1, ITPKA, LATS1, MAP3K7, ACVR1B, MAP3K5, CSNK2A1, CLK3, PKN3, SLK, MAP3K9, AAK1, MAP3K8, TLK1, PRKACB, AKT3, CHUK, IRAK2, CDC7, SRPK2, CSNK1G1, LIMK1, PIM1, CDK8, WNK1, PKN2, MINK1, PRKCH, CDK6, CDK4, PRKCE, STK4, MARK4, WEE1, SRPK1, MARK1, MAPK1, PDIK1L, MAP4K5, CCND3, MAPK4, MAPK3, MAP3K10, BUB1B, MAPK9, NEK9, MAP3K14, LRRK2, MAP3K13, EIF2AK3, LRRK1, MAP3K12, MAP3K11, NEK2, MAPKAPK5, STK17B, BMPR2, AKAP13, FKBP1A, AKAP9, CHEK1, EXOSC10, VRK1, MAP3K3, MAP3K2, LMTK2, DYRK4, CAMK2B, DYRK2, MAP2K1, TAOK2, TAOK1, TGFBR1, NLK, TGFBR2, MAP2K4, TAOK3, RPS6KA5, RPS6KA3, RPS6KA4, RPS6KA2, DYRK1B, DYRK1A, CDC42BPA, TGFBR3, BMPR1A
GO:0019900~kinase binding	48	2.63	7.94E-09	9.82E-06	CAV1, PDLIM5, PRKAG2, PDE3B, TRIB3, ABI2, CHEK1, NBEA, PRDX3, FOXO3, LATS1, TRIB1, IGF1R, CDKN2A, SQSTM1, CDKN2C, SLC2A1, FAS, AXIN2, APC, DLG1, ADAM9, ACTB, MAP2K1, TAOK1, TGFBR2, PRKAB2, SKI, RB1, PPP1CC, IRS1, STAT3, MAPK1, SPAG9, CCND1, DUSP2, RPS6KA4, CCND3, CCND2, BTG1, AKAP5, PEBP1, MARCKS, JAK2, AKAP1, MAP3K13, MAP3K12, MAP3K11
GO:0003712~transcription cofactor activity	78	4.27	7.98E-09	9.88E-06	E2F1, COPS2, CDX2, TSG101, HIRA, PAWR, ZEB1, MXI1, CBFB, CITED2, TMF1, MAX, CCNE1, SMARCD2, HSF2, MKL2, NR2F2,

					USP16, MKL1, TBPL1, SERTAD2, TBL1XR1, CTBP2, YY1, RELB, ZFX, MECP2, RB1, TAF6L, MBD1, DDIT3, SFRS2, EP300, MTF1, NCOA3, IFNB1, NCOA4, BTG1, NCOA6, MAP3K10, C19ORF2, TFAP2A, NFE2L1, ZFPM2, TGFB1I1, CARM1, MED1, CREM, TRIB3, ELK3, TCF20, NPM1, NPAT, TAF9, TCF4, TRIP11, KAT2B, EPAS1, KLF12, RYBP, YWHAB, SKI, ICMT, DDX5, UBP1, SIRT1, ATXN7L3, ATF5, RNF4, DR1, DYRK1B, SMARCC1, GRLF1, RBM14, TBL1X, HDAC9, NCOR2, TOB1
GO:0003700~transcription factor activity	164	8.99	1.34E-08	1.66E-05	MEF2C, CDX2, THRA, HIRA, HOXD13, FOXO1, TBP, PTTG1, FOXO3, TCEAL1, LASS6, HOXD10, CBFB, CITED2, CDKN2A, GATA6, TARDBP, FOXF2, RARB, SATB2, STRN3, MTA2, YY1, RELB, ZHX2, EOMES, HNF4G, ZNF3, DDIT3, HHEX, ZNF193, ZNF236, EP300, HIF1A, ZNF238, MTF1, HNF4A, MLLT10, TFAP2A, NFE2L1, PRDM2, MYNN, SUPT6H, HOXA13, SOX2, SOX4, ELK3, SRF, NR2C1, HOXA10, TCF4, RUNX1, TCF3, PLAGL2, MAFG, PLAG1, KLF6, IKZF4, MLL, EPAS1, KLF12, KLF9, SMAD7, TAF5, SMAD6, SMAD5, FOXA1, KLF11, L3MBTL, NR4A2, NR4A1, SMAD2, NR4A3, EN2, UBP1, FOXP1, HOXB4, ATF5, NOTCH1, RNF4, PHF1, HDAC1, DMTF1, ETS1, TRPS1, TCF12, KLF3, E2F1, PPARA, PPARD, E2F3, ELF2, E2F5, ARID4A, ZNFX1, MITF, NR6A1, PPARG, FOXK2, SPI1, ZEB2, ZEB1, GLI3, ZNF207, MAX, BLZF1, HSF2, ZNF148, HEY2, NR2F2, FOSL1, MYC, ZNF281, NANOG, EGR2, SNAPC2, FOXJ2, SOX11, ESR1, RUNX1T1, TP53, LEF1, RB1, FOXJ3, MBD1, ZNF140, MYCN, PURA, HOXC10, HOXC11, CLOCK, TBX19, BTAF1, CREM, NR3C1, ZNF175, TCF7L1, ZFP36L1, TFAM, TSC22D3, NPAS2, TSC22D2, TSC22D4, XBP1, NPAT, NFAT5, POU2F1, ETV1, BCL6,

					POU3F2, ETV6, NFATC3, CEBPB, HMBOX1, AFF4, CREB5, AFF1, STAT3, TP73, RLF, MEF2D, PKNOX1, IRF1, NFIB
GO:0019901~protein kinase binding	40	2.19	1.09E-07	1.35E-04	PDLIM5, PRKAG2, PDE3B, TRIB3, CHEK1, NBEA, PRDX3, FOXO3, LATS1, TRIB1, CDKN2A, SQSTM1, CDKN2C, AXIN2, DLG1, ADAM9, APC, ACTB, MAP2K1, TAOK1, TGFBR2, PRKAB2, SKI, PPP1CC, IRS1, STAT3, MAPK1, SPAG9, CCND1, DUSP2, RPS6KA4, CCND3, CCND2, AKAP5, PEBP1, MARCKS, AKAP1, MAP3K13, MAP3K12, MAP3K11
GO:0003714~transcription corepressor activity	39	2.14	2.23E-07	2.76E-04	E2F1, COPS2, CDX2, TSG101, HIRA, TRIB3, PAWR, ELK3, ZEB1, MXI1, CITED2, NPAT, TCF4, NR2F2, TBL1XR1, CTBP2, KLF12, YY1, RELB, YWHAB, RYBP, MECP2, SKI, SIRT1, UBP1, MBD1, DDIT3, SFRS2, ATF5, IFNB1, DR1, MAP3K10, C19ORF2, GRLF1, ZFPM2, HDAC9, TBL1X, NCOR2, TOB1
GO:0000166~nucleotide binding	315	17.26	7.88E-07	9.74E-04	RBPMS2, DYNC1LI2, PGD, ADCY6, SYNCRIP, LEMD3, RNF213, RAB1A, MAP3K7, KIF13A, ATP2B1, SFRS7, MAP3K5, DDX17, SFRS5, ATP2B4, CRY2, CLK3, MAP3K9, MAP3K8, ABCB10, RAPGEF4, TLK1, DDX10, GTPBP2, MAGI3, MAGI1, KIF5B, KIF5C, SPAG1, PIM1, WNK1, UBE2J1, MYH9, MARK4, MARK1, SFRS3, SFRS2, RND3, MAPK1, RFC3, RFC1, DHX29, RFC2, MAPK4, MAPK3, DLD, RAB14, PDGFRA, NEK9, MAPK9, YME1L1, ARL8B, RAB10, EIF2AK3, MDH1, ADSS, GNAI3, NEK2, ERBB3, PFKFB3, ERBB2, STK17B, CHEK1, IGF2BP3, HSPA1B, NAGK, ATP12A, RAB40B, VRK1, KRAS, ZAP70, PABPC3, PABPC1, POLQ, SFRS11, DHX57, KIF3B, MLL, MAP2K1, TAOK2, TAOK1, TGFBR1, TAOK3, MAP2K4, TGFBR2, SKI, ATP11C, NXF1, DDX5, EPHA5, HYOU1, EPHA4, EPHA7, UPF3B, ATP2A2, ARF3, CDC42BPA, RIT1, DHX40, RAB5B,

ZNFX1, FASTK, ATP5B, GNL3L, LATS1, RNGTT, ARL5A, CHST12, GUCY1A3, DOCK10, CDC7, RAP2A, SRPK2, CTBP2, RAP2C, TP53, DECR1, DGUOK, NDUFA10, MBD1, SRPK1, MTRR, PDIK1L, TAF15, SQLE, ADK, EIF4A2, EIF4A1, MAP3K10, GNAS, MAP3K14, MAP3K13, ARL4C, MAP3K12, REV3L, MAP3K11, GPHN, ACSL1, ACTL6A, KIF21A, ACSL4, RBM25, MARS, RBM26, DNM3, MAT2A, NLK, AXL, ELAVL2, APRT, RPS6KA5, MEF2D, PAPOLA, RPS6KA3, RAB30, RPS6KA4, ILF2, FYN, RPS6KA2, RAB22A, RAB34, HSPA4L, RBM18, RBM14, ABCC5, DNM2, RALY, NRBP1, STK38, ZAK, CTPS, RBM6, CASK, RPS6KB1, ITPKB, ITPKA, CNOT4, TOP1, CSNK2A1, TARDBP, AAK1, RRAS, PRKACB, SAR1B, MX2, SAR1A, RBM12, DARS, CSNK1G1, STK4, POR, CLPX, WEE1, MAP4K5, FBXO18, SMARCA5, LRRK2, LRRK1, RAD17, PCCA, ASS1, GNE, MAPKAPK5, HDGF, BMPR2, RRAGA, ABCA1, RRAGD, KARS, SRC, UBE2D2, DDX3Y, PPIL4, LMTK2, DYRK4, RHOBTB1, TAF9, ERO1L, DYRK2, UBE2D1, RUNX1, HCN3, RHOBTB3, ABCB9, RAB8B, MYO1B, RYK, MET, DOCK9, SACS, SSB, ABCB7, ABCB6, ATP7A, P2RX4, MYO10, PSMC6, P2RX7, VCP, DYRK1B, DYRK1A, RHOT1, RAP1A, SCN8A, UBE2E1, HSP90AB1, PRKAG2, PIP5K1B, CDC42, KIF2C, ACVR1B, ARHGAP5, SLK, PKN3, RHOA, RHOC, TPR, TUBA1A, AKT3, CHUK, KIAA0232, IRAK2, C9ORF86, ARL1, LIMK1, G3BP2, CDK8, PKN2, PFKP, MINK1, UBE2F, PRKCH, CDK6, PRKCE, CDK4, HLTF, UBE2B, UBE2N, KIF1C, PANK3, KIF1B, PEBP1, BUB1B, UBE2W, ORC5L, TXNRD1, UBE2T, BTAF1, SR140, TRIB3, AKAP9, TRIB1, TYMS, IGF1R, CHD9, MAP3K3, MAP3K2, CHD1, CAMK2B, UCK2, CABC1, YES1, TNRC6B, CHD5, HSPA8, ACTB,

GO:0032553~ribonucleotide binding	265	14.52	8.14E-07	1.01E-03	FLT1, MSH3, SIRT1, RASL11B, HSP90B1, JAK1, JAK2, BMPR1A DYNC1LI2, ADCY6, RAB1A, MAP3K7, ATP2B1, KIF13A, MAP3K5, DDX17, ATP2B4, CLK3, MAP3K9, MAP3K8, ABCB10, RAPGEF4, TLK1, DDX10, GTPBP2, MAGI3, KIF5B, MAGI1, KIF5C, SPAG1, WNK1, UBE2J1, PIM1, MYH9, MARK4, MARK1, RND3, MAPK1, RFC3, RFC1, DHX29, RFC2, MAPK4, MAPK3, RAB14, PDGFRA, MAPK9, NEK9, YME1L1, ARL8B, RAB10, EIF2AK3, ADSS, GNAI3, NEK2, ERBB3, PFKFB3, ERBB2, STK17B, CHEK1, HSPA1B, NAGK, ATP12A, RAB40B, VRK1, KRAS, ZAP70, POLQ, DHX57, KIF3B, MLL, MAP2K1, TAOK2, TAOK1, TGFBR1, TAOK3, MAP2K4, TGFBR2, ATP11C, DDX5, EPHA5, HYOU1, EPHA4, EPHA7, ATP2A2, ARF3, CDC42BPA, RIT1, DHX40, RAB5B, ZNFX1, ATP5B, FASTK, GNL3L, LATS1, RNGTT, ARL5A, GUCY1A3, DOCK10, CDC7, RAP2A, SRPK2, RAP2C, TP53, DGUOK, NDUFA10, SRPK1, PDIK1L, ADK, EIF4A2, EIF4A1, MAP3K10, GNAS, MAP3K14, ARL4C, MAP3K13, MAP3K12, MAP3K11, ACSL1, ACTL6A, KIF21A, ACSL4, MARS, DNM3, MAT2A, NLK, AXL, APRT, RPS6KA5, PAPOLA, RPS6KA3, RAB30, RPS6KA4, ILF2, FYN, RPS6KA2, RAB22A, RAB34, HSPA4L, ABCC5, DNM2, NRBP1, STK38, ZAK, CTPS, CASK, RPS6KB1, ITPKB, ITPKA, TOP1, CSNK2A1, AAK1, RRAS, PRKACB, SAR1B, MX2, SAR1A, DARS, CSNK1G1, STK4, CLPX, WEE1, MAP4K5, FBXO18, SMARCA5, LRRK2, LRRK1, PCCA, RAD17, ASS1, GNE, MAPKAPK5, BMPR2, RRAGA, ABCA1, RRAGD, KARS, SRC, UBE2D2, DDX3Y, LMTK2, DYRK4, RHOBTB1, TAF9, DYRK2, UBE2D1, RUNX1, HCN3, RHOBTB3, ABCB9, RAB8B, MYO1B, RYK, MET, DOCK9, SACS, ABCB7, ABCB6, ATP7A, P2RX4, PSMC6,

GO:0032555~purine ribonucleotide binding	265	14.52	8.14E-07	1.01E-03	MYO10, P2RX7, VCP, DYRK1B, DYRK1A, RHOT1, RAP1A, SCN8A, UBE2E1, HSP90AB1, PRKAG2, PIP5K1B, CDC42, KIF2C, ACVR1B, ARHGAP5, SLK, PKN3, RHOA, RHOC, TPR, TUBA1A, AKT3, CHUK, KIAA0232, IRAK2, C9ORF86, ARL1, LIMK1, PFKP, PKN2, CDK8, MINK1, UBE2F, PRKCH, CDK6, PRKCE, HLTF, CDK4, UBE2B, UBE2N, KIF1C, PANK3, KIF1B, PEBP1, UBE2W, ORC5L, BUB1B, UBE2T, BTAF1, TRIB3, AKAP9, TRIB1, IGF1R, CHD9, MAP3K3, MAP3K2, CHD1, CAMK2B, UCK2, CABC1, YES1, CHD5, HSPA8, ACTB, FLT1, MSH3, RASL11B, HSP90B1, JAK1, JAK2, BMPR1A DYNC1LI2, ADCY6, RAB1A, MAP3K7, ATP2B1, KIF13A, MAP3K5, DDX17, ATP2B4, CLK3, MAP3K9, MAP3K8, ABCB10, RAPGEF4, TLK1, DDX10, GTPBP2, MAGI3, KIF5B, MAGI1, KIF5C, SPAG1, WNK1, UBE2J1, PIM1, MYH9, MARK4, MARK1, RND3, MAPK1, RFC3, RFC1, DHX29, RFC2, MAPK4, MAPK3, RAB14, PDGFRA, MAPK9, NEK9, YME1L1, ARL8B, RAB10, EIF2AK3, ADSS, GNAI3, NEK2, ERBB3, PFKFB3, ERBB2, STK17B, CHEK1, HSPA1B, NAGK, ATP12A, RAB40B, VRK1, KRAS, ZAP70, POLQ, DHX57, KIF3B, MLL, MAP2K1, TAOK2, TAOK1, TGFBR1, TAOK3, MAP2K4, TGFBR2, ATP11C, DDX5, EPHA5, HYOU1, EPHA4, EPHA7, ATP2A2, ARF3, CDC42BPA, RIT1, DHX40, RAB5B, ZNFX1, ATP5B, FASTK, GNL3L, LATS1, RNGTT, ARL5A, GUCY1A3, DOCK10, CDC7, RAP2A, SRPK2, RAP2C, TP53, DGUOK, NDUFA10, SRPK1, PDIK1L, ADK, EIF4A2, EIF4A1, MAP3K10, GNAS, MAP3K14, ARL4C, MAP3K13, MAP3K12, MAP3K11, ACSL1, ACTL6A, KIF21A, ACSL4, MARS, DNM3, MAT2A, NLK, AXL, APRT, RPS6KA5, PAPOLA, RPS6KA3, RAB30, RPS6KA4, ILF2, FYN, RPS6KA2, RAB22A,

					RAB34, HSPA4L, ABCC5, DNM2, NRBP1, STK38, ZAK, CTPS, CASK, RPS6KB1, ITPKB, ITPKA, TOP1, CSNK2A1, AAK1, RRAS, PRKACB, SAR1B, MX2, SAR1A, DARS, CSNK1G1, STK4, CLPX, WEE1, MAP4K5, FBXO18, SMARCA5, LRRK2, LRRK1, PCCA, RAD17, ASS1, GNE, MAPKAPK5, BMPR2, RRAGA, ABCA1, RRAGD, KARS, SRC, UBE2D2, DDX3Y, LMTK2, DYRK4, RHOBTB1, TAF9, DYRK2, UBE2D1, RUNX1, HCN3, RHOBTB3, ABCB9, RAB8B, MYO1B, RYK, MET, DOCK9, SACS, ABCB7, ABCB6, ATP7A, P2RX4, PSMC6, MYO10, P2RX7, VCP, DYRK1B, DYRK1A, RHOT1, RAP1A, SCN8A, UBE2E1, HSP90AB1, PRKAG2, PIP5K1B, CDC42, KIF2C, ACVR1B, ARHGAP5, SLK, PKN3, RHOA, RHOC, TPR, TUBA1A, AKT3, CHUK, KIAA0232, IRAK2, C9ORF86, ARL1, LIMK1, PFKP, PKN2, CDK8, MINK1, UBE2F, PRKCH, CDK6, PRKCE, HLTF, CDK4, UBE2B, UBE2N, KIF1C, PANK3, KIF1B, PEBP1, UBE2W, ORC5L, BUB1B, UBE2T, BTAF1, TRIB3, AKAP9, TRIB1, IGF1R, CHD9, MAP3K3, MAP3K2, CHD1, CAMK2B, UCK2, CABC1, YES1, CHD5, HSPA8, ACTB, FLT1, MSH3, RASL11B, HSP90B1, JAK1, JAK2, BMPR1A
GO:0004709~MAP kinase kinase kinase activity	12	0.66	8.97E-07	1.11E-03	MAP3K7, MAP3K5, ZAK, MAP3K3, MAP3K2, MAP3K9, MAP3K8, MAP3K10, MAP3K14, MAP3K13, MAP3K12, MAP3K11
GO:0019904~protein domain specific binding	67	3.67	1.00E-06	1.24E-03	HSP90AB1, PPARA, CADM1, UVRAG, GJA1, PAWR, PTTG1, PTEN, ARHGAP5, SFRS5, ARHGAP1, QKI, CNTNAP1, TUBA1A, CASP2, ADAM9, KHDRBS1, STMN3, INPPL1, STRN3, MECP2, IRS1, SIRPA, VASP, SH2D2A, CCDC6, GRM7, SH3KBP1, YWHAZ, LITAF, CRIPT, ABI2, DAZAP2, SRC, KRAS, SQSTM1, SOS1, BCL2, TAF9, ETV6, CARD8, BMP2, NLK, L3MBTL, YWHAB, LIN7C, SKI, SHANK2, SIRT1, PTPN12, DOCK4, CTR9, EPS15, SH3BP5, SON, ZFP106, VCP,

					TOM1L1, PLSCR4, GRIA2, YWHAQ, TGFBR3, JAK2, LRP2, TBL1X, CRK, CALM2
GO:0042802~identical protein binding	110	6.03	1.68E-06	2.08E-03	SYT1, NRBP1, THRA, ZAK, CADM1, MBIP, MAP3K5, APP, MAP3K9, CPOX, EED, FAS, GABPB2, MYH9, PPP1CC, STK4, BRAP, ZNF3, GCC2, HNF4A, VEGFA, PDGFRA, SNRPC, LRRK2, EIF2AK3, ALDOA, CAV1, PFKFB3, ERBB3, STRAP, ERBB2, NFKBIA, RRAGA, CCDC91, LMAN1, SRF, NR2C1, ECE1, NPM1, PRKRA, RUNX1, TCF3, B4GALT1, CARD8, BMP2, IKZF2, MLL, MOCS2, SMAD6, L3MBTL, NR4A1, ITGA4, CASC3, FOXP1, P2RX4, P2RX7, HDAC1, CDC42BPA, PON2, SCHIP1, IMPA1, NR6A1, PRDX3, DAXX, TRIM8, HSF2, CASP8, MKL2, CASP2, CHUK, PHC2, IRAK2, NOL3, RUNX1T1, ADIPOR1, TARBP2, ADRB2, MAP3K10, ORC5L, GNAS, MAP3K13, MAP3K12, MAP3K11, SAV1, CALCOCO2, EEA1, HPRT1, DTNBP1, DCI, MYCBP2, EXOSC10, MTMR2, IGF1R, CEP57, BCL2, PCMT1, PAFAH1B1, POU3F2, THBS1, CEBPB, FLT1, MSH3, PDS5A, SIRT1, ATXN1, GLA, FYN, BNIP3L, SUMF2, BMPR1A
GO:0017076~purine nucleotide binding	271	14.85	3.36E-06	4.15E-03	DYNC1LI2, ADCY6, RAB1A, MAP3K7, ATP2B1, KIF13A, MAP3K5, DDX17, ATP2B4, CLK3, MAP3K9, MAP3K8, ABCB10, RAPGEF4, TLK1, DDX10, GTPBP2, MAGI3, KIF5B, MAGI1, KIF5C, SPAG1, WNK1, UBE2J1, PIM1, MYH9, MARK4, MARK1, RND3, MAPK1, RFC3, RFC1, DHX29, RFC2, MAPK4, MAPK3, DLD, RAB14, PDGFRA, NEK9, MAPK9, YME1L1, ARL8B, RAB10, EIF2AK3, ADSS, GNAI3, NEK2, ERBB3, PFKFB3, ERBB2, STK17B, CHEK1, HSPA1B, NAGK, ATP12A, RAB40B, VRK1, KRAS, ZAP70, POLQ, DHX57, KIF3B, MLL, MAP2K1, TAOK2, TAOK1, TGFBR1, TAOK3, MAP2K4, TGFBR2, ATP11C, DDX5, EPHA5, HYOU1, EPHA4, EPHA7, ATP2A2,

					ARF3, CDC42BPA, RIT1, DHX40, RAB5B, ZNFX1, ATP5B, FASTK, GNL3L, LATS1, RNGTT, ARL5A, CHST12, GUCY1A3, DOCK10, CDC7, RAP2A, SRPK2, RAP2C, TP53, DGUOK, NDUFA10, SRPK1, MTRR, PDIK1L, SQLE, ADK, EIF4A2, EIF4A1, MAP3K10, GNAS, MAP3K14, ARL4C, MAP3K13, MAP3K12, MAP3K11, ACSL1, ACTL6A, KIF21A, ACSL4, MARS, DNM3, MAT2A, NLK, AXL, APRT, RPS6KA5, PAPOLA, RPS6KA3, RAB30, RPS6KA4, ILF2, FYN, RPS6KA2, RAB22A, RAB34, HSPA4L, ABCC5, DNM2, NRBP1, STK38, ZAK, CTPS, CASK, RPS6KB1, ITPKB, ITPKA, TOP1, CSNK2A1, AAK1, RRAS, PRKACB, SAR1B, MX2, SAR1A, DARS, CSNK1G1, STK4, CLPX, WEE1, MAP4K5, FBXO18, SMARCA5, LRRK2, LRRK1, PCCA, RAD17, ASS1, GNE, MAPKAPK5, BMPR2, RRAGA, ABCA1, RRAGD, KARS, SRC, UBE2D2, DDX3Y, LMTK2, DYRK4, RHOBTB1, TAF9, ERO1L, DYRK2, UBE2D1, RUNX1, HCN3, RHOBTB3, ABCB9, RAB8B, MYO1B, RYK, MET, DOCK9, SACS, ABCB7, ABCB6, ATP7A, P2RX4, PSMC6, MYO10, P2RX7, VCP, DYRK1B, DYRK1A, RHOT1, RAP1A, SCN8A, UBE2E1, HSP90AB1, PRKAG2, PIP5K1B, CDC42, KIF2C, ACVR1B, ARHGAP5, SLK, PKN3, RHOA, RHOC, TPR, TUBA1A, AKT3, CHUK, KIAA0232, IRAK2, C9ORF86, ARL1, LIMK1, PFKP, PKN2, CDK8, MINK1, UBE2F, PRKCH, CDK6, PRKCE, HLTF, CDK4, UBE2B, UBE2N, KIF1C, PANK3, KIF1B, PEBP1, UBE2W, ORC5L, BUB1B, TXNRD1, UBE2T, BTAF1, TRIB3, AKAP9, TRIB1, IGF1R, CHD9, MAP3K3, MAP3K2, CHD1, CAMK2B, UCK2, CABC1, YES1, CHD5, HSPA8, ACTB, FLT1, MSH3, RASL11B, HSP90B1, JAK1, JAK2, BMPR1A
GO:0032559~adenyl	218	11.95	5.15E-06	6.35E-03	DYNC1LI2, ADCY6, RAB1A, ATP2B1, KIF13A, MAP3K7, MAP3K5,

ribonucleotide binding	DDX17, ATP2B4, CLK3, MAP3K9, MAP3K8, ABCB10, RAPGEF4, TLK1, DDX10, MAGI3, KIF5B, MAGI1, KIF5C, WNK1, UBE2J1, PIM1, MYH9, MARK4, MARK1, MAPK1, RFC3, DHX29, RFC1, RFC2, MAPK4, MAPK3, PDGFRA, MAPK9, NEK9, YME1L1, EIF2AK3, PFKFB3, ERBB3, NEK2, ERBB2, STK17B, CHEK1, HSPA1B, NAGK, ATP12A, VRK1, ZAP70, POLQ, DHX57, KIF3B, MAP2K1, TAOK2, TAOK1, TGFBR1, MAP2K4, TGFBR2, TAOK3, ATP11C, DDX5, EPHA5, HYOU1, EPHA4, EPHA7, ATP2A2, CDC42BPA, DHX40, ZNFX1, ATP5B, FASTK, LATS1, CDC7, SRPK2, TP53, DGUOK, NDUFA10, SRPK1, PDIK1L, ADK, EIF4A2, EIF4A1, MAP3K10, MAP3K14, MAP3K13, MAP3K12, MAP3K11, ACSL1, ACTL6A, ACSL4, KIF21A, MARS, MAT2A, NLK, AXL, APRT, RPS6KA5, PAPOLA, RPS6KA3, RPS6KA4, ILF2, RPS6KA2, FYN, HSPA4L, ABCC5, NRBP1, STK38, ZAK, CTPS, CASK, RPS6KB1, ITPKB, ITPKA, TOP1, CSNK2A1, AAK1, PRKACB, CSNK1G1, DARS, STK4, WEE1, CLPX, MAP4K5, FBXO18, SMARCA5, LRRK2, LRRK1, PCCA, RAD17, ASS1, GNE, MAPKAPK5, BMPR2, ABCA1, SRC, KARS, UBE2D2, DDX3Y, LMTK2, DYRK4, TAF9, DYRK2, UBE2D1, RUNX1, HCN3, RHOBTB3, ABCB9, RYK, MYO1B, MET, SACS, ABCB7, ABCB6, ATP7A, P2RX4, P2RX7, PSMC6, MYO10, VCP, DYRK1B, DYRK1A, SCN8A, UBE2E1, HSP90AB1, PRKAG2, PIP5K1B, ACVR1B, KIF2C, SLK, PKN3, TPR, AKT3, CHUK, KIAA0232, IRAK2, LIMK1, PFKP, PKN2, CDK8, MINK1, UBE2F, PRKCH, CDK6, PRKCE, HLTF, CDK4, UBE2B, UBE2N, KIF1C, PANK3, KIF1B, UBE2W, ORC5L, BUB1B, PEBP1, UBE2T, BTAF1, TRIB3, AKAP9, TRIB1, CHD9, IGF1R, MAP3K3, MAP3K2, CHD1,

					CAMK2B, UCK2, CABC1, YES1, CHD5, HSPA8, ACTB, FLT1, MSH3, HSP90B1, JAK1, JAK2, BMPR1A
GO:0008092~cytoskeletal protein binding	89	4.88	6.27E-06	7.73E-03	LIMA1, WASF3, PDLIM5, LMO7, MYLIP, SDC2, KLHL2, VCL, KLHL5, MED28, GSN, CXCR4, TARDBP, PLS1, RHOA, SNTB2, MKL2, MKL1, KLHL20, DLG1, KCNMA1, KIAA1949, STX1A, TWF1, KIF5B, INPPL1, NUSAP1, SPIRE1, MYH9, PALLD, MARK4, VASP, FARP1, ARPC1A, SPAG9, RAB11FIP5, MYRIP, EPB41L1, TNS1, KIF1B, NDEL1, ARPC5L, SDCBP, MAPRE2, MAPRE1, STMN1, ARL8B, MAPRE3, ALDOA, SHROOM3, RABGAP1, CNN3, SSFA2, CALD1, ABI2, ARFGEF2, TPM1, TPM3, PFN2, NUMA1, PKD2, WIPF2, PAFAH1B1, SSX2IP, CDC42EP3, APC, B4GALT1, GABARAPL2, GABARAPL1, PHACTR4, EPB41, PTPN3, MYO1B, PTPN4, PSRC1, TNKS1BP1, CORO1C, GMFB, MYO10, SYNE1, LASP1, FYN, SVIL, SPTBN2, SPTBN1, TMOD3, MARCKS, CALM2, DNM2
GO:0005524~ATP binding	215	11.78	6.62E-06	8.16E-03	DYNC1LI2, ADCY6, RAB1A, ATP2B1, KIF13A, MAP3K7, MAP3K5, DDX17, ATP2B4, CLK3, MAP3K9, MAP3K8, ABCB10, TLK1, DDX10, MAGI3, KIF5B, MAGI1, KIF5C, WNK1, UBE2J1, PIM1, MYH9, MARK4, MARK1, MAPK1, RFC3, DHX29, RFC1, RFC2, MAPK4, MAPK3, PDGFRA, MAPK9, NEK9, YME1L1, EIF2AK3, PFKFB3, ERBB3, NEK2, ERBB2, STK17B, CHEK1, HSPA1B, NAGK, ATP12A, VRK1, ZAP70, POLQ, DHX57, KIF3B, MAP2K1, TAOK2, TAOK1, TGFBR1, MAP2K4, TGFBR2, TAOK3, ATP11C, DDX5, EPHA5, HYOU1, EPHA4, EPHA7, ATP2A2, CDC42BPA, DHX40, ZNFX1, ATP5B, FASTK, LATS1, CDC7, SRPK2, TP53, DGUOK, NDUFA10, SRPK1, PDIK1L, ADK, EIF4A2, EIF4A1, MAP3K10, MAP3K14,

					MAP3K13, MAP3K12, MAP3K11, ACSL1, ACTL6A, ACSL4, KIF21A, MARS, MAT2A, NLK, AXL, RPS6KA5, PAPOLA, RPS6KA3, RPS6KA4, ILF2, RPS6KA2, FYN, HSPA4L, ABCC5, NRBP1, STK38, ZAK, CTPS, CASK, RPS6KB1, ITPKB, ITPKA, TOP1, CSNK2A1, AAK1, PRKACB, CSNK1G1, DARS, STK4, WEE1, CLPX, MAP4K5, FBXO18, SMARCA5, LRRK2, LRRK1, PCCA, RAD17, ASS1, GNE, MAPKAPK5, BMPR2, ABCA1, SRC, KARS, UBE2D2, DDX3Y, LMTK2, DYRK4, TAF9, DYRK2, UBE2D1, RUNX1, RHOBTB3, ABCB9, RYK, MYO1B, MET, SACS, ABCB7, ABCB6, ATP7A, P2RX4, P2RX7, PSMC6, MYO10, VCP, DYRK1B, DYRK1A, SCN8A, UBE2E1, HSP90AB1, PRKAG2, PIP5K1B, ACVR1B, KIF2C, SLK, PKN3, TPR, AKT3, CHUK, KIAA0232, IRAK2, LIMK1, PFKP, PKN2, CDK8, MINK1, UBE2F, PRKCH, CDK6, PRKCE, HLTF, CDK4, UBE2B, UBE2N, KIF1C, PANK3, KIF1B, UBE2W, ORC5L, BUB1B, PEBP1, UBE2T, BTAF1, TRIB3, AKAP9, TRIB1, CHD9, IGF1R, MAP3K3, MAP3K2, CHD1, CAMK2B, UCK2, CABC1, YES1, CHD5, HSPA8, ACTB, FLT1, MSH3, HSP90B1, JAK1, JAK2, BMPR1A
GO:0046983~protein dimerization activity	94	5.15	7.19E-06	8.86E-03	NRBP1, THRA, CADM1, NR6A1, PPARG, DAXX, MAX, MAP3K5, TRIM8, HSF2, MAP3K9, GUCY1A3, FOSL1, GABPB2, IRAK2, PPP2R1A, STX1A, LIMK1, TP53, RUNX1T1, ADIPOR1, MYH9, STK4, DDIT3, TARBP2, ADRB2, HIF1A, HNF4A, VEGFA, MAP3K10, PDGFRA, TFAP2A, SDCBP, NFE2L1, SNRPC, BIN1, LRRK2, MAP3K13, MAP3K12, MAP3K11, MCL1, ERBB3, CREM, ERBB2, RRAGA, CALCOCO2, EEA1, RRAGD, HPRT1, SRF, ZNF618, MYCBP2, NR2C1, MTMR2, ECE1, CEP57, XBP1, PPP2CA, BCL2, NPM1, PRKRA, PAFAH1B1, PPP3CA, RUNX1, TCF4, TCF3,

					B4GALT1, CARD8, BMP2, CEBPB, IKZF2, ACY1, MLL, MSH3, EPAS1, TAF5, SMAD6, TGFBR1, TGFBR2, NR4A2, NR4A1, CREB5, FOXP1, STAT3, P2RX4, ATF5, NOTCH2, P2RX7, PKNOX1, GLA, BNIP3L, SUMF2, PDCD6, BMPR1A
GO:0003677~DNA binding	318	17.42	9.12E-06	1.12E-02	MEF2C, CDX2, HIRA, HOXD13, LEMD3, C16ORF75, PTTG1, MXI1, HOXD10, CITED2, ZFP91, APP, FOXF2, PHTF2, RARB, TBPL1, H1F0, RCOR3, ZNF646, STRN3, YY1, RCOR1, ZHX2, MECP2, EOMES, ZNF791, HNF4G, TAF6L, ZNF236, RFC3, ZNF238, RFC1, PRDM4, HNF4A, MTF1, RFC2, MLLT10, ZZZ3, PRDM2, SUPT6H, RAD23B, HOXA13, GPBP1L1, AHCTF1, OBFC2A, ZNF618, ZNF512, TCF20, HOXA10, ZNF124, TCF4, POLQ, TCF3, PLAGL2, PLAG1, DNMT3A, IKZF4, KLF6, MLL, IKZF2, KLF9, KLF12, SMAD7, ZNF121, SMAD6, SMAD5, L3MBTL, KLF11, RYBP, ZBTB41, SMAD2, EN2, UBP1, ZNF629, NOTCH1, RNF6, RNF4, ETS1, DMTF1, TRPS1, H3F3B, TCF12, PARP2, NCOR2, KLF3, PPARA, PPARD, ELF2, ZBTB34, ZNFX1, TSG101, EZH1, PPARG, MITF, ZNF530, SPI1, ZEB2, ZEB1, GLI3, BLZF1, GLI4, HSF2, ZNF148, BRD4, NR2F2, MYB, MYC, ZNF281, ZDHHC1, SNAPC2, SNAPC1, TP53, MBD5, MBD4, MBD1, ZNF140, ZNF335, MYCN, ZNF691, TAF15, ASH1L, ZNF551, CLOCK, TBX19, MED1, REV3L, CREM, ABI2, ZNF655, ZNF175, ZFP36L1, XBP1, SOS1, NPAT, CSDE1, ETV1, BCL6, HBP1, CC2D1A, ETV6, BAZ2A, ZNF562, POLR3F, SSRP1, ZNF264, ZNF565, JARID2, AFF4, PPP1R10, AFF1, TP73, STAT3, PREB, RLF, MEF2D, PKNOX1, ILF2, ZBTB5, THRA, LMO2, FOXO1, RBM6, TBP, FOXO3, LASS6, TCEAL1, CBFB, ZIC2, PNN, TOP1, CDKN2A, MIER2, GATA6, TARDBP, RC3H2, MLL3, MYST4, MLL2, ZNF594, SATB2, RBL2,

					MTA2, RELB, MND1, ARID1A, TOPBP1, DDIT3, ZNF3, ZNF193, HHEX, ZNF439, EP300, HIF1A, FBXO18, SMARCA5, TFAP2A, NFE2L1, TNFAIP3, MYNN, ZNF586, ZFAND6, HMGB3, SOX3, SOX2, HDGF, SOX4, ELK3, SRF, NR2C1, DDX3Y, ORC6L, ZNF597, TAF9, RUNX1, TRAF4, TAF2, MAFG, EPAS1, TAF5, FOXA1, JRKL, NR4A2, NR4A1, NR4A3, FOXP1, HDAC4, ATF5, HOXB4, HDAC1, PHF1, SMARCC1, ZIC5, PDCD6, DNAJB6, ZNF410, E2F1, E2F3, ARID4A, E2F5, GPBP1, DEDD, ARID4B, FOXK2, NR6A1, ZNF207, TMF1, PHC3, KIF2C, MAX, FUBP3, SLK, HEY2, RTF1, SOX17, FOSL1, PHC2, KHDRBS1, TBL1XR1, NANOG, REV1, EGR2, FOXJ2, SOX11, ZFX, SOX12, RUNX1T1, ESR1, LEF1, NUSAP1, RB1, FOXJ3, HLTF, HMGA2, VAT1, PURA, HOXC10, HOXC11, BPTF, ZNF711, ORC5L, ZFPM2, BTAF1, ZNF800, WBP11, NR3C1, TCF7L1, CHD9, TFAM, HIC2, NPAS2, TSC22D3, TSC22D2, TSPYL2, TSC22D4, NFAT5, POU2F1, CHD1, POU3F2, NFATC3, ZNF701, CHD5, ZNF700, BAHD1, EXO1, ZBTB7A, CEBPB, MSH3, HMBOX1, CREB5, SON, SP3, DR1, H2AFY2, IRF1, GRLF1, DENND4A, TBL1X, NFIB
GO:0003682~chromatin binding	36	1.97	1.14E-05	1.40E-02	MORF4L1, MKRN1, ARID4A, EZH1, SOX2, MITF, ARID4B, HIRA, HOXD13, ZEB1, GLI3, CBX7, HOXD10, CHD9, TOP1, SF3B1, GATA6, CHD1, BCL6, ACTL6A, NFATC3, CHD5, ASF1A, DNMT3A, SATB2, MLL, L3MBTL, TP53, LEF1, NOTCH1, SP3, RNF2, SMARCC1, NCOA6, SMARCA5, MED1
GO:0030554~adenyl nucleotide binding	224	12.27	2.16E-05	2.64E-02	DYNC1LI2, ADCY6, RAB1A, ATP2B1, KIF13A, MAP3K7, MAP3K5, DDX17, ATP2B4, CLK3, MAP3K9, MAP3K8, ABCB10, RAPGEF4, TLK1, DDX10, MAGI3, KIF5B, MAGI1, KIF5C, WNK1, UBE2J1, PIM1, MYH9, MARK4, MARK1, MAPK1, RFC3, DHX29, RFC1, RFC2,

					MAPK4, DLD, MAPK3, PDGFRA, MAPK9, NEK9, YME1L1, EIF2AK3, PFKFB3, ERBB3, NEK2, ERBB2, STK17B, CHEK1, HSPA1B, NAGK, ATP12A, VRK1, ZAP70, POLQ, DHX57, KIF3B, MAP2K1, TAOK2, TAOK1, TGFBR1, TAOK3, MAP2K4, TGFBR2, ATP11C, DDX5, EPHA5, HYOU1, EPHA4, EPHA7, ATP2A2, CDC42BPA, DHX40, ZNFX1, ATP5B, FASTK, LATS1, CHST12, CDC7, SRPK2, TP53, DGUOK, NDUFA10, SRPK1, MTRR, PDIK1L, SQLE, ADK, EIF4A2, EIF4A1, MAP3K10, MAP3K14, MAP3K13, MAP3K12, MAP3K11, ACSL1, ACTL6A, ACSL4, KIF21A, MARS, MAT2A, NLK, AXL, APRT, RPS6KA5, PAPOLA, RPS6KA3, RPS6KA4, ILF2, RPS6KA2, FYN, HSPA4L, ABCC5, NRBP1, STK38, ZAK, CTPS, CASK, RPS6KB1, ITPKB, ITPKA, TOP1, CSNK2A1, AAK1, PRKACB, DARS, CSNK1G1, STK4, WEE1, CLPX, MAP4K5, FBXO18, SMARCA5, LRRK2, LRRK1, PCCA, RAD17, ASS1, GNE, MAPKAPK5, BMPR2, ABCA1, SRC, KARS, UBE2D2, DDX3Y, LMTK2, DYRK4, TAF9, DYRK2, ERO1L, UBE2D1, RUNX1, HCN3, RHOBTB3, ABCB9, RYK, MYO1B, MET, SACS, ABCB7, ABCB6, ATP7A, P2RX4, P2RX7, PSMC6, MYO10, VCP, DYRK1B, DYRK1A, SCN8A, UBE2E1, HSP90AB1, PRKAG2, PIP5K1B, ACVR1B, KIF2C, SLK, PKN3, TPR, AKT3, CHUK, KIAA0232, IRAK2, LIMK1, PFKP, PKN2, CDK8, MINK1, UBE2F, PRKCH, CDK6, PRKCE, HLTF, CDK4, UBE2B, UBE2N, KIF1C, PANK3, KIF1B, PEBP1, UBE2W, ORC5L, BUB1B, TXNRD1, UBE2T, BTAF1, TRIB3, AKAP9, TRIB1, CHD9, IGF1R, MAP3K3, MAP3K2, CHD1, CAMK2B, UCK2, CABC1, YES1, CHD5, HSPA8, ACTB, FLT1, MSH3, HSP90B1, JAK1, JAK2, BMPR1A
GO:0001883~purine	226	12.38	2.98E-05	3.62E-02	DYNC1LI2, ADCY6, RAB1A, ATP2B1, KIF13A, MAP3K7, MAP3K5,

nucleoside binding	DDX17, ATP2B4, CLK3, MAP3K9, MAP3K8, ABCB10, RAPGEF4, TLK1, DDX10, MAGI3, KIF5B, MAGI1, KIF5C, WNK1, UBE2J1, PIM1, MYH9, MARK4, MARK1, MAPK1, RFC3, DHX29, RFC1, RFC2, MAPK4, DLD, MAPK3, PDGFRA, MAPK9, NEK9, YME1L1, ARL8B, EIF2AK3, PFKFB3, ERBB3, NEK2, ERBB2, STK17B, CHEK1, HSPA1B, NAGK, ATP12A, VRK1, KRAS, ZAP70, POLQ, DHX57, KIF3B, MAP2K1, TAOK2, TAOK1, TGFBR1, TAOK3, MAP2K4, TGFBR2, ATP11C, DDX5, EPHA5, HYOU1, EPHA4, EPHA7, ATP2A2, CDC42BPA, DHX40, ZNFX1, ATP5B, FASTK, LATS1, CHST12, CDC7, SRPK2, TP53, DGUOK, NDUFA10, SRPK1, MTRR, PDIK1L, SQLE, ADK, EIF4A2, EIF4A1, MAP3K10, MAP3K14, MAP3K13, MAP3K12, MAP3K11, ACSL1, ACTL6A, ACSL4, KIF21A, MARS, MAT2A, NLK, AXL, APRT, RPS6KA5, PAPOLA, RPS6KA3, RPS6KA4, ILF2, FYN, RPS6KA2, HSPA4L, ABCC5, NRBP1, STK38, ZAK, CTPS, CASK, RPS6KB1, ITPKB, ITPKA, TOP1, CSNK2A1, AAK1, PRKACB, DARS, CSNK1G1, STK4, WEE1, CLPX, MAP4K5, FBXO18, SMARCA5, LRRK2, LRRK1, PCCA, RAD17, ASS1, GNE, MAPKAPK5, BMPR2, ABCA1, SRC, KARS, UBE2D2, DDX3Y, LMTK2, DYRK4, TAF9, DYRK2, ERO1L, UBE2D1, RUNX1, HCN3, RHOBTB3, ABCB9, RYK, MYO1B, MET, SACS, ABCB7, ABCB6, ATP7A, P2RX4, P2RX7, PSMC6, MYO10, VCP, DYRK1B, DYRK1A, SCN8A, UBE2E1, HSP90AB1, PRKAG2, PIP5K1B, ACVR1B, KIF2C, SLK, PKN3, TPR, AKT3, CHUK, KIAA0232, IRAK2, LIMK1, PFKP, PKN2, CDK8, MINK1, UBE2F, PRKCH, CDK6, PRKCE, HLTF, CDK4, UBE2B, UBE2N, KIF1C, PANK3, KIF1B, PEBP1, UBE2W, ORC5L, BUB1B, TXNRD1, UBE2T, BTAF1, TRIB3, AKAP9, TRIB1, CHD9,

GO:0043565~sequence-specific DNA binding	100	5.48	3.22E-05	3.91E-02	IGF1R, MAP3K3, MAP3K2, CHD1, CAMK2B, UCK2, CABC1, YES1, CHD5, HSPA8, ACTB, FLT1, MSH3, HSP90B1, JAK1, JAK2, BMPR1A MEF2C, THRA, CDX2, FOXO1, HOXD13, FOXO3, LASS6, HOXD10, GATA6, FOXF2, RARB, SATB2, MTA2, ZHX2, HNF4G, DDIT3, HHEX, HIF1A, HNF4A, NFE2L1, HOXA13, SOX2, ELK3, SRF, NR2C1, HOXA10, TAF9, TCF3, MAFG, TAF2, EPAS1, TAF5, SMAD7, FOXA1, NR4A2, NR4A1, SMAD2, NR4A3, EN2, FOXP1, ATF5, HOXB4, HDAC4, NOTCH1, ETS1, TRPS1, TCF12, PPARA, PPARD, ELF2, FOXK2, PPARG, NR6A1, MITF, SPI1, ZEB2, ZEB1, GLI3, MAX, KIF2C, ZNF148, HSF2, NR2F2, MYC, FOSL1, ZNF281, TBL1XR1, NANOG, FOXJ2, TP53, ESR1, LEF1, MBD4, FOXJ3, HMGA2, MBD1, PURA, HOXC10, HOXC11, BPTF, ORC5L, CREM, NR3C1, TSPYL2, XBP1, POU2F1, ETV1, POU3F2, BCL6, ETV6, CEBPB, MSH3, HMBOX1, CREB5, STAT3, MEF2D, PKNOX1, DR1, IRF1, TBL1X

Table S4

Significant KEGG pathway terms (adjusted p-value<0.05) enriched for the targets of the identified miRNA biomarkers.

Term	Count	%	p-value	Adjusted p-value	Genes
hsa05200:Pathways in cancer	94	5.150	4.43E-15	8.08E-13	HSP90AB1, E2F1, PPARD, E2F3, WNT3A, MITF, PPARG, SPI1, FOXO1, MMP2, PTEN, GLI3, CCNE2, CDC42, CUL2, ACVR1B, MAX, WNT1, CCNE1, CDKN2A, SLC2A1, CASP8, RHOA, RARB, FAS, TPR, MYC, CSF2RA, AKT3, CHUK, WNT10B, CTBP2, TP53, RUNX1T1, LEF1, CDK6, RB1, CDK4, STK4, MAPK1, CCDC6, CCND1, CRKL, EP300, HIF1A, PIAS3, NCOA4, MAPK3, VEGFA, PDGFRA, MAPK9, LAMC1, WNT5A, XIAP, ERBB2, EGLN3, NFKBIA, TCF7L1, TPM3, IGF1R, KRAS, SOS1, BCL2, RUNX1, AXIN2, TRAF4, PIK3R1, APC, DVL3, BMP2, COL4A1, MSH3, EPAS1, IL8, MAP2K1, VHL, TGFBR1, TGFBR2, MET, FZD3, SMAD2, BIRC3, FZD4, STAT3, FZD7, FZD6, CDKN1A, HSP90B1, LAMA3, PLCG1, HDAC1, ETS1, JAK1, CRK
hsa04310:Wnt signaling pathway	49	2.684	4.53E-10	8.25E-08	PPARD, WNT3A, PPP2R5C, MAP3K7, WNT1, CSNK2A1, RHOA, PRKACB, SOX17, PLCB1, MYC, FOSL1, CUL1, PPP2R1B, PPP2R1A, TBL1XR1, WNT10B, CTBP2, VANGL1, TP53, LEF1, CCND1, EP300, CCND3, CCND2, MAPK9, SIAH1, WNT5A, PPP3R1, TCF7L1, PPP2CA, NFAT5, PPP3CB, CAMK2B, PPP3CA, AXIN2, FBXW11, NFATC3, APC, DVL3, NLK, SMAD2, FZD3, FZD4, FZD7, FZD6, LRP6, TBL1X, LRP5

hsa04110:Cell cycle	40	2.191	3.69E-08	6.72E-06	E2F1, YWHAZ, E2F3, E2F5, ANAPC13, CHEK1, PTTG1, CCNE2, CCNE1, CDKN2A, CDKN2C, ORC6L, MYC, STAG2, CUL1, BUB3, STAG1, CDC7, RBL2, RBL1, YWHAB, TP53, CDC23, CDK6, SMAD2, RB1, CDK4, CDC25A, WEE1, CDC25B, CDKN1C, CDKN1A, CCND1, EP300, CCND3, HDAC1, CCND2, YWHAQ, BUB1B, ORC5L
hsa04115:p53 signaling pathway	26	1.424	3.46E-07	6.30E-05	ZMAT3, CHEK1, PMAIP1, CCNG2, SESN1, PTEN, SESN3, CCNE2, CCNE1, CDKN2A, CASP8, SERPINE1, FAS, THBS1, TP53, CDK6, CDK4, TP73, RFWD2, CDKN1A, CCND1, TNFRSF10B, CCND3, CCND2, SIAH1, MDM4
hsa04010:MAPK signaling pathway	64	3.506	4.14E-07	7.54E-05	MEF2C, ZAK, DAXX, MAP3K7, CDC42, ACVR1B, MAX, MAP3K5, MAP3K8, RRAS, FAS, PRKACB, RAPGEF2, MYC, CHUK, AKT3, RELB, TP53, STK4, DDIT3, MAPK1, CRKL, MAPK3, PDGFRA, MAPK9, STMN1, MAP3K14, MAP3K13, MAP3K12, MAP3K11, MAPKAPK5, PPP3R1, CACNB3, HSPA1B, PPM1B, SRF, KRAS, MAP3K3, MAP3K2, DUSP14, SOS1, PPP3CB, PPP3CA, HSPA8, MAP2K1, TAOK2, TAOK1, NLK, TGFBR1, TAOK3, MAP2K4, TGFBR2, NR4A1, CDC25B, RPS6KA5, DUSP4, RPS6KA3, PLA2G4A, DUSP2, RPS6KA4, RPS6KA2, RAP1A, DUSP8, CRK
hsa04350:TGF-beta signaling pathway	30	1.643	4.55E-07	8.29E-05	LTBP1, E2F5, BMPR2, RPS6KB1, ACVR1B, ZFYVE9, ZFYVE16, PPP2CA, RHOA, THBS1, MYC, CUL1, PPP2R1B, PPP2R1A, BMP2, RBL2, SMAD7, TGFBR1, SMAD6, RBL1, TGFBR2, SMAD5, SMAD2, MAPK1, EP300, MAPK3, SMURF1, BMP7, BMP6, BMPR1A

hsa05210:Colorectal cancer	29	1.589	7.20E-07	1.31E-04	TCF7L1, ACVR1B, IGF1R, KRAS, BCL2, SOS1, AXIN2, MYC, AKT3, PIK3R1, APC, DVL3, MSH3, MAP2K1, TGFBR1, TGFBR2, MET, TP53, LEF1, SMAD2, FZD3, FZD4, FZD7, FZD6, MAPK1, CCND1, MAPK3, PDGFRA, MAPK9
hsa05220:Chronic myeloid leukemia	27	1.479	7.64E-07	1.39E-04	E2F1, E2F3, NFKBIA, ACVR1B, CDKN2A, KRAS, SOS1, RUNX1, MYC, CHUK, AKT3, PIK3R1, CTBP2, MAP2K1, TGFBR1, TGFBR2, TP53, CDK6, RB1, CDK4, MAPK1, CDKN1A, CCND1, CRKL, HDAC1, MAPK3, CRK
hsa05215:Prostate cancer	30	1.643	7.83E-07	1.42E-04	E2F1, HSP90AB1, E2F3, ERBB2, NFKBIA, FOXO1, PTEN, TCF7L1, CCNE2, CCNE1, IGF1R, KRAS, BCL2, SOS1, PDGFD, CHUK, AKT3, PIK3R1, MAP2K1, TP53, LEF1, CREB5, RB1, MAPK1, HSP90B1, CDKN1A, CCND1, EP300, MAPK3, PDGFRA
hsa05219:Bladder cancer	19	1.0410	1.15E-06	2.09E-04	E2F1, E2F3, IL8, MAP2K1, ERBB2, TP53, RB1, CDK4, MMP2, RPS6KA5, MAPK1, CCND1, CDKN1A, CDKN2A, KRAS, VEGFA, MAPK3, THBS1, MYC
hsa04520:Adherens junction	27	1.479	1.36E-06	2.48E-04	WASF3, ERBB2, LMO7, TCF7L1, SRC, VCL, MAP3K7, CDC42, ACVR1B, IGF1R, CSNK2A1, RHOA, SSX2IP, YES1, ACTB, PTPRJ, TGFBR1, NLK, TGFBR2, MET, LEF1, SMAD2, MAPK1, EP300, FYN, MAPK3, PTPN1
hsa04722:Neurotrophin signaling pathway	36	1.972	2.65E-06	4.83E-04	YWHAZ, NFKBIA, FOXO3, CDC42, MAP3K5, KRAS, MAP3K3, BCL2, SOS1, GAB1, RHOA, CAMK2B, FRS2, AKT3, PIK3R1, ARHGDIB, IRAK2, MAP2K1, YWHAB, TP53, IRS1, TP73, RPS6KA5, MAPK1, RPS6KA3, CRKL, RPS6KA4, PLCG1, PRDM4, RPS6KA2, MAPK3, YWHAQ, MAPK9, RAP1A, CRK, CALM2

hsa04144:Endocytosis	47	2.575	3.12E-06	5.67E-04	CLTA, LDLR, RAB5B, TSG101, ERBB3, PIP5K1B, EEA1, HSPA1B, CLTC, SRC, CDC42, IGF1R, ACVR1B, AP2B1, DAB2, RNF103, CXCR4, GIT2, NEDD4L, ITCH, HSPA8, IQSEC1, AP2M1, FAM125B, DNM3, PARD6B, FLT1, TGFBR1, VTA1, MET, TGFBR2, HLA-C, HLA-B, LDLRAP1, EPS15, RAB11FIP5, ADRB2, RABEP1, AP2A1, RAB22A, PDGFRA, SH3KBP1, SMURF1, PDCD6IP, VPS28, RAB11FIP1, F2R, DNM2
hsa05212:Pancreatic cancer	25	1.369	4.51E-06	8.20E-04	E2F1, E2F3, MAP2K1, TGFBR1, ERBB2, TGFBR2, TP53, CDK6, SMAD2, RB1, CDK4, STAT3, CDC42, MAPK1, ACVR1B, CCND1, KRAS, CDKN2A, VEGFA, MAPK3, JAK1, MAPK9, CHUK, AKT3, PIK3R1
hsa05214:Glioma	22	1.205	1.78E-05	0.003239616	E2F1, E2F3, MAP2K1, TP53, CDK6, RB1, CDK4, PTEN, MAPK1, IGF1R, CCND1, CDKN1A, CDKN2A, KRAS, PLCG1, SOS1, MAPK3, PDGFRA, CAMK2B, CALM2, PIK3R1, AKT3
hsa05223:Non-small cell lung cancer	20	1.095	1.87E-05	0.00340011	E2F1, E2F3, MAP2K1, ERBB2, TP53, CDK6, RB1, FOXO3, CDK4, STK4, MAPK1, CCND1, CDKN2A, KRAS, PLCG1, SOS1, MAPK3, RARB, PIK3R1, AKT3
hsa05216:Thyroid cancer	14	0.767	2.03E-05	0.0036813	MAP2K1, PPARG, TP53, LEF1, TCF7L1, TPM3, MAPK1, CCDC6, CCND1, KRAS, NCOA4, MAPK3, TPR, MYC
hsa04114:Oocyte meiosis	30	1.643	7.68E-05	0.013873988	YWHAZ, ANAPC13, ADCY6, PPP2R5C, PPP3R1, PTTG1, CCNE2, CCNE1, IGF1R, SLK, PPP2CA, PPP3CB, CAMK2B, PRKACB, PPP3CA, FBXW11, CUL1, PPP2R1B, PPP2R1A, MAP2K1, YWHAB, CDC23, PPP1CC, ITPR1, MAPK1, RPS6KA3, RPS6KA2, MAPK3, YWHAQ, CALM2
hsa05222:Small cell lung cancer	25	1.369	8.07E-05	0.014588043	E2F1, E2F3, COL4A1, XIAP, TP53, NFKBIA, CDK6, RB1, BIRC3, CDK4, PTEN, CCNE2, CCNE1, MAX, CCND1, LAMA3, PIAS3,

					BCL2, LAMC1, RARB, MYC, CHUK, PIK3R1, TRAF4, AKT3
hsa05211:Renal cell carcinoma	22	1.205	1.04E-04	0.018713192	MAP2K1, EPAS1, VHL, MET, EGLN3, CDC42, MAPK1, CUL2, CRKL, EP300, HIF1A, KRAS, ETS1, SOS1, VEGFA, GAB1, SLC2A1, MAPK3, RAP1A, CRK, PIK3R1, AKT3
hsa04510:Focal adhesion	45	2.465	1.67E-04	0.029851255	CAV1, XIAP, TNC, ERBB2, PTEN, SRC, VCL, CDC42, IGF1R, ARHGAP5, ITGB8, BCL2, SOS1, RHOA, PDGFD, ZYX, THBS1, AKT3, PIK3R1, ACTB, TNXB, COL4A1, FLT1, MAP2K1, MET, ITGA4, BIRC3, PPP1CC, COL5A1, VASP, MAPK1, CCND1, CRKL, LAMA3, CCND3, CCND2, FYN, VEGFA, MAPK3, PDGFRA, GRLF1, RAP1A, MAPK9, LAMC1, CRK
hsa05221:Acute myeloid leukemia	19	1.041	2.00E-04	0.0357429	PPARD, MAP2K1, PIM1, SPI1, RUNX1T1, LEF1, RPS6KB1, STAT3, TCF7L1, MAPK1, CCND1, KRAS, SOS1, MAPK3, RUNX1, MYC, CHUK, PIK3R1, AKT3

Table S5:

Significant IPA pathway terms (adjusted p-value<0.05) enriched for the targets of the identified miRNA biomarkers

Ingenuity Canonical Pathways	p-value	Adjusted p-value	Molecules
Molecular Mechanisms of Cancer	3.9811E-25	1.74E-22	WNT10B,TGFBR1,PIK3R1,CDKN2C,KRAS,RBL1,CCND1,RB1,CCND3,PLCB1,FRS2,ITGA4,TP53,SMAD2,CCNE2,RRAS,TCF3,RAP1A,DAXX,CCND2,MAX,GAB1,IRS1,APH1A,E2F1,ARHGEF10,NOTCH1,WNT1,RAP2A,FYN,TCF4,LRP6,CDK4,BMPR2,CRK,JAK2,MAP3K5,EP300,CHEK1,SOS1,E2F5,PRKCE,ARHGEF3,CASP8,CAMK2B,PMAIP1,RHOC,ADCY6,GNAI3,CCNE1,WNT3A,CDKN1A,BMP7,BMP6,CASP7,LRP1,FZD7,CDKN2A,MAP2K4,PRKACB,JAK1,MAPK1,FZD3,MYC,TGFBR2,MAPK3,MAP3K7,ARHGEF11,BIRC3,CDC25A,CDK6,APC,RND3,CDC42,RHOA,FZD6,PRKCH,LEF1,BMP2,HIF1A,SMAD5,E2F3,KLB,FAS,BCL2,CDC25B,NLK,NFKBIA,BMPR1A,RHOT1,AKT3,MAP2K1,SRC,LRP5,GNAS,HAT1,SMAD7,SMAD6,MAPK9,XIAP,FZD4,FOXO1,PRKAG2,WNT5A
Glioblastoma Multiforme Signaling	3.1623E-16	1.38E-13	CDKN2A,WNT10B,MAPK1,FZD3,PIK3R1,KRAS,CCND1,PTEN,MYC,RB1,MAPK3,PLCB1,FRS2,TP53,RPS6KB1,RRAS,CDK6,ITPR1,PLCL2,TCF3,APC,GAB1,RND3,CDC42,IRS1,RHOA,E2F1,FZD6,LEF1,PDGFD,WNT1,CDK4,E2F3,KLB,MTOR,RHOT1,SOS1,PDGFRA,IGF1R,E2F5,AKT3,MAP2K1,SRC,RHOC,PLCG1,CCNE1,WNT3A,FZD4,FOXO1,CDKN1A,FZD7,WNT5A
Wnt/β-catenin Signaling	7.9433E-16	3.47E-13	CDKN2A,CSNK2A1,TGFBR1,WNT10B,CSNK1G1,PPP2CA,FZD3,TGFBR3,SOX12,CCND1,SOX2,MYC,TGFBR2,MAP3K7,TP53,SOX4,GJA1,AXIN2,TCF3,ACVR1B,APC,PPP2R1A,CDH5,PPP2R3A,FZD6,LEF1,WNT1,TCF4,LRP6,BMPR2,EP300,SOX17,NLK,RARB,AKT3,PPP2R5C,SRC,LRP5,PPARD,HDAC1,TCF7L1,SOX11,WNT3A,FZD4,TLE4,CD44,DVL3,UBC,PPP2R1B,LRP1,SOX3,FZD7,WNT5A

HGF Signaling	7.9433E-14	3.47E-11	CDKN2A,MAP2K4,MAP3K11,MAPK1,PIK3R1,KRAS,MAP3K5,CCND1,KLB,MAP3K10,MAPK3,SOS1,MAP3K7,AKT3,PRKCE,FRS2,MAP2K1,ITGA4,MAP3K2,ETS1,MAP3K9,MAP3K14,RRAS,CRKL,MAP3K13,MAPK9,PLCG1,STAT3,RAP1A,MET,MAP3K12,ELF2,GAB1,CDC42,IRS1,CDKN1A,MAP3K8,PRKCH,MAP3K3,ELK3
NGF Signaling	1.5849E-13	6.93E-11	MAP2K4,MAP3K11,MAPK1,PIK3R1,RPS6KA3,CRK,KRAS,MAP3K5,KLB,EP300,MAP3K10,MAPK3,TRAF4,SOS1,MAP3K7,AKT3,CHUK,RPS6KA2,FRS2,MAP2K1,MAP3K2,TP53,MAP3K14,RPS6KB1,MAP3K9,RRAS,MAP3K13,PLCG1,MAPK9,RPS6KA5,CREB5,RAP1A,MAP3K12,GAB1,CDC42,IRS1,RHOA,MAP3K8,RPS6KA4,MAP3K3
Glucocorticoid Receptor Signaling	6.3096E-13	2.76E-10	MAP2K4,PRKACB,JAK1,TGFBR1,MAPK1,PRKAB2,NFATC3,PIK3R1,HSPA1A/HSPA1B,SMARCD2,KRAS,TSC22D3,TGFBR2,MAPK3,PPP3R1,MAP3K7,SERPINE1,FRS2,MAP3K14,CXCL8,TAF9,SMAD2,CDKN1C,RRAS,MED1,TBP,POLR2H,STAT3,NCOA3,HSPA8,POU2F1,GAB1,TAF5,IRS1,ESR1,PHF10,ARID1A,JAK2,NR3C1,KLB,BCL2,EP300,HSP90B1,NFKBIA,NFAT5,KAT2B,HSP90AB1,PPP3CB,SOS1,FOXO3,AKT3,CHUK,MAP2K1,TAF2,PPP3CA,ADRB2,ACTB,MAPK9,CEBPB,TSG101,TAF6L,CDKN1A,PRKAG2,PLAU,NCOR2,SMARCC1,HLTF,TAF15
STAT3 Pathway	7.9433E-13	3.47E-10	MAP2K4,TGFBR1,MAP3K11,MAPK1,TGFBR3,SOCS6,BMPR2,KRAS,JAK2,BCL2,TGFBR2,MYC,MAP3K10,BMPR1A,PIM1,MAPK3,PDGFRA,IGF1R,MAP2K1,CDC25A,MAP3K9,SRC,FLT1,RRAS,MAPK9,STAT3,IGF2R,PIAS3,MAP3K12,CDKN1A
Mouse Embryonic Stem Cell Pluripotency	3.1623E-12	1.38E-09	TCF4,JAK1,LIF,MAPK1,FZD3,PIK3R1,BMPR2,KRAS,JAK2,SMAD5,KLB,MYC,SOX2,BMPR1A,MAPK3,SOS1,MAP3K7,AKT3,MAP2K1,FRS2,TP53,RRAS,STAT3,TCF7L1,TCF3,APC,XIAP,NANOG,FZD4,WNT3A,GAB1,IRS1,FZD6,LEF1,DVL3,FZD7

PI3K/AKT Signaling	5.0119E-12	2.19E-09	JAK1,MAPK1,PPP2CA,PIK3R1,KRAS,INPPL1,MAP3K5,JAK2,CCND1,OCRL,BCL2,PTEN,YWHAQ,MTOR,HSP90B1,NFKBIA,HSP90AB1,MAPK3,FOXO3,SOS1,AKT3,PPP2R5C,CHUK,MAP2K1,ITGA4,MCL1,TP53,RPS6KB1,RRAS,YWHAB,YWHAZ,PPP2R1A,NANOG,FOXO1,GAB1,PPP2R3A,CDKN1A,MAP3K8,PPP2R1B
Glioma Signaling	1E-11	4.37E-09	CDKN2A,MAPK1,PIK3R1,CDK4,CDKN2C,KRAS,E2F3,RBL1,CCND1,KLB,PTEN,RB1,MTOR,MAPK3,SOS1,E2F5,IGF1R,PDGFRA,PRKCE,AKT3,FRS2,MAP2K1,CAMK2B,TP53,RBL2,RRAS,CDK6,PLCG1,IGF2R,CALM1 (includes others),GAB1,IRS1,CDKN1A,E2F1,PRKCH,PDGFD
RANK Signaling in Osteoclasts	1.2589E-11	5.50E-09	MAP2K4,MAP3K11,MAPK1,PIK3R1,MAP3K5,KLB,MAP3K10,NFKBIA,PPP3CB,MAPK3,PPP3R1,MAP3K7,AKT3,CHUK,MAP2K1,BIRC3,FRS2,PPP3CA,MAP3K2,MAP3K9,MAP3K14,SRC,TNFSF11,MITF,MAP3K13,MAPK9,GSN,XIAP,MAP3K12,CALM1 (includes others),GAB1,IRS1,MAP3K8,MAP3K3
B Cell Receptor Signaling	1.2589E-11	5.50E-09	MAP2K4,RAP2A,MAP3K11,MAPK1,NFATC3,PIK3R1,INPPL1,KRAS,MAP3K5,BCL6,KLB,OCRL,EP300,PTEN,MAP3K10,MTOR,NFKBIA,NFAT5,PPP3CB,MAPK3,PPP3R1,SOS1,MAP3K7,AKT3,CHUK,MAP2K1,FRS2,PPP3CA,CAMK2B,MAP3K2,ETS1,RPS6KB1,MAP3K9,MAP3K14,RRAS,MAP3K13,MAPK9,TCF3,CREB5,RAP1A,CALM1 (includes others),MAP3K12,FOXO1,GAB1,CDC42,IRS1,MEF2C,MAP3K8,MAP3K3
Role of NANOG in Mammalian Embryonic Stem Cell Pluripotency	1.2589E-11	5.50E-09	JAK1,LIF,WNT10B,MAPK1,FZD3,PIK3R1,BMP2,BMPR2,KRAS,JAK2,SMAD5,KLB,SOX2,BMPR1A,MAPK3,SOS1,AKT3,GATA6,MAP2K1,FRS2,TP53,RRAS,CDX2,TCF7L1,STAT3,APC,NANOG,FZD4,WNT3A,GAB1,IRS1,FZD6,BMP7,BMP6,WNT1,WNT5A,FZD7,TCL1A
Cell Cycle: G1/S Checkpoint Regulation	2.5119E-11	1.10E-08	CDKN2A,CUL1,CDK4,CDKN2C,E2F3,RBL1,CCND1,MYC,RB1,CCND3,E2F5,CDC25A,TP53,HDAC9,CCNE2,RBL2,HDAC4,HDAC1,CDK6,CCNE1,CCND2,MAX,FOXO1,E2F1,CDKN1A,RPL5

Colorectal Cancer Metastasis Signaling	2.5119E-11	1.10E-08	MAP2K4,PRKACB,WNT10B,JAK1,TGFBR1,MAPK1,FZD3,MSH3,PIK3R1,GNB5,KRAS,CCND1,MMP24,GNB1,VEGFA,TGFBR2,MYC,GNB4,MAPK3,FRS2,TP53,SMAD2,RRAS,MMP2,STAT3,TCF3,APC,GAB1,RND3,IRS1,RHOA,FZD6,GNB2,LEF1,WNT1,SIAH1,TCF4,PTGER3,LRP6,JAK2,KLB,RHOT1,SOS1,AKT3,MAP2K1,SRC,LRP5,GNAS,RHOC,ADCY6,MAPK9,TCF7L1,FZD4,WNT3A,PRKAG2,LRP1,FZD7,WNT5A
Chronic Myeloid Leukemia Signaling	3.9811E-11	1.74E-08	CDKN2A,TGFBR1,MAPK1,PIK3R1,CDK4,KRAS,CRK,E2F3,RBL1,CCND1,KLB,MYC,TGFBR2,RB1,MAPK3,CTBP2,SOS1,E2F5,AKT3,CHUK,MAP2K1,FRS2,TP53,HDAC9,RBL2,HDAC4,RRAS,CRKL,HDAC1,CDK6,GAB1,IRS1,CDKN1A,E2F1
Cardiac Hypertrophy Signaling	5.0119E-11	2.19E-08	MAP2K4,PRKACB,MAP3K11,TGFBR1,MAPK1,PIK3R1,GNB5,SRF,KRAS,TGFBR2,GNB1,GNB4,MAP3K10,MAPK3,PPP3R1,MAP3K7,PLCB1,FRS2,ADRA1B,MAP3K2,MAP3K14,MAP3K9,RPS6KB1,RRAS,MAP3K13,PLCL2,MAP3K12,GAB1,RND3,IRS1,RHOA,GNB2,MAP3K5,KLB,EP300,MTOR,PPP3CB,RHOT1,SOS1,IGF1R,MAP2K1,PPP3CA,ADRB2,GNAS,RHOC,ADCY6,MAPK9,PLCG1,ADSS,CALM1 (includes others),GNAI3,MEF2D,PRKAG2,MEF2C,MAP3K8,MAP3K3
Protein Kinase A Signaling	5.0119E-11	2.19E-08	PRKACB,DUSP8,TGFBR1,MAPK1,NFATC3,AKAP9,GNB5,PTEN,TGFBR2,YWHAQ,GNB1,GNB4,MAPK3,PPP3R1,PLCB1,DUSP11,CDC25A,SMPDL3A,KDELR1,PTPRD,YWHAB,YWHAZ,ITPR1,PTPN3,PLCL2,CREB5,TCF3,RAP1A,AKAP13,H3F3A/H3F3B,RHOA,CREM,GNB2,PRKCH,LEF1,DUSP4,H1F0,SIRPA,AKAP12,TCF4,PTPN9,PDE4A,CDC23,PTPN12,DUSP2,AKAP11,EP300,CDC25B,AKAP2,PTPN4,PPP1CC,NFAT5,NFKBIA,PTPRJ,PPP1R10,GLI3,PPP3CB,PDE3B,PTPN1,PRKCE,CHUK,MAP2K1,VASP,PPP3CA,MTMR3,CAMK2B,AKAP5,GNAS,ADCY6,PLCG1,TCF7L1,ANAPC13,GNAI3,CALM1 (includes others),PRKAG2,EYA1,AKAP1
Ovarian Cancer Signaling	6.3096E-11	2.76E-08	CDKN2A,PRKACB,TCF4,WNT10B,MAPK1,FZD3,PIK3R1,CDK4,KRAS,CCND1,KLB,PTEN,BCL2,VEGFA,RB1,MTOR,MAPK3,AKT3,FRS2,MAP2K1,TP53,RPS6KB1,SRC,GJA1,RRAS,MMP2,TCF7L1,TCF3,APC,FZD4,WNT3A,GAB1,IRS1,E2F1,C

			D44,PRKAG2,FZD6,LEF1,WNT1,WNT5A,FZD7
Role of Macrophages, Fibroblasts and Endothelial Cells in Rheumatoid Arthritis	6.3096E-11	2.76E-08	MAP2K4,WNT10B,MAPK1,NFATC3,FZD3,PIK3R1,IL32,KRAS,CCND1,MYC,VEGFA,MAPK3,PPP3R1,TRAF4,MAP3K7,PLCB1,LTBR,FRS2,IL1RAP,MAP3K14,CXCL8,RRAS,STAT3,PLCL2,TCF3,CREB5,APC,GAB1,RHOA,IRS1,FZD6,PRKCH,LEF1,PDGFD,WNT1,TCF4,LRP6,JAK2,KLB,EP300,NFKBIA,NLK,NFAT5,PPP3CB,PRKCE,AKT3,CHUK,MAP2K1,PPP3CA,CAMK2B,SRC,TNFSF11,LRP5,IL15,MAPK9,PLCG1,CEBPB,TCF7L1,CALM1 (includes others),FZD4,WNT3A,LRP1,RYK,FZD7,WNT5A,IRAK2
Adipogenesis pathway	6.3096E-11	2.76E-08	CDKN2A,WNT10B,DDIT3,FZD3,BMP2,NR2F2,RBBP7,FBXW7,BMPR2,HIF1A,SMAD5,PAXIP1,EGR2,KAT2B,BMPR1A,CTBP2,CLOCK,TBL1XR1,TP53,PPARG,ATG5,HDAC9,RPS6KB1,HDAC4,HAT1,TXNIP,HDAC1,XBP1,CEBPB,RUNX1T1,FZD4,KAT6B,FOXO1,SIRT1,FZD6,KLF3,BMP7,FZD7,WNT5A
p53 Signaling	7.9433E-11	3.47E-08	CDKN2A,TP73,PIK3R1,CDK4,HIF1A,CCND1,KLB,FAS,EP300,BCL2,PTEN,CHEK1,RB1,KAT2B,THBS1,STAG1,ADCK3,AKT3,FRS2,TP53,HDAC9,PMAIP1,TP53INP1,MED1,TOPBP1,HDAC1,TNFRSF10B,TIGAR,MDM4,CCND2,GAB1,IRS1,CDKN1A,E2F1,SIRT1
ERK/MAPK Signaling	7.9433E-11	3.47E-08	PRKACB,MAPK1,PPP2CA,PIK3R1,SRF,KRAS,MYC,YWHAQ,MAPK3,FRS2,ITGA4,ETS1,YWHAB,RRAS,CRKL,YWHAZ,MAPKAPK5,STAT3,CREB5,RAP1A,PPP2R1A,H3F3A/H3F3B,GAB1,PPP2R3A,IRS1,DUSP4,ESR1,ELK3,FYN,CRK,RAPGEF4,KLB,DUSP2,EP300,PPP1CC,PPP1R10,SOS1,PRKCE,PPP2R5C,MAP2K1,PPARG,SRC,MYCN,PLCG1,RPS6KA5,PLA2G4A,ELF2,PRKAG2,RPS6KA4,PPP2R1B
Regulation of the Epithelial-Mesenchymal Transition	1.1482E-10	5.02E-08	MAP2K4,TCF4,JAK1,TGFBR1,WNT10B,MAPK1,FZD3,PIK3R1,PARD6B,KRAS,HIF1A,JAK2,KLB,SMURF1,TGFBR2,NOTCH2,MAPK3,SOS1,AKT3,FRS2,MAP2K1,HMGA2,ETS1,SMAD2,RRAS,MMP2,ZEB1,TCF7L1,STAT3,TCF3,APC,MET,FZD4

Pathway			,WNT3A,GAB1,APH1A,IRS1,RHOA,ZEB2,FZD6,LEF1,DVL3,PDGFD,JAG1,NOTCH1,WNT1,WNT5A,FZD7
Role of NFAT in Cardiac Hypertrophy	1.6982E-10	7.42E-08	MAP2K4,PRKACB,TGFBR1,LIF,MAPK1,PIK3R1,GNB5,KRAS,KLB,EP300,GNB1,TGFBR2,GNB4,PPP3CB,PPP3R1,MAPK3,SOS1,MAP3K7,IGF1R,PRKCE,AKT3,PLCB1,FRS2,MAP2K1,PPP3CA,CAMK2B,HDAC9,AKAP5,SRC,GNAS,HDAC4,RRAS,HDAC1,ADCY6,MAPK9,PLCG1,PLCL2,ITPR1,GNAI3,CALM1 (includes others),GAB1,IRS1,MEF2D,GNB2,PRKAG2,MEF2C,PRKCH,IL11
ERK5 Signaling	1.6982E-10	7.42E-08	SH2D2A,RPS6KB1,SRC,LIF,YWHAB,RRAS,RPS6KA3,YWHAZ,RPS6KA5,KRAS,CREB5,EP300,YWHAQ,MYC,GAB1,MEF2D,FOXO3,RPS6KA4,MAP3K8,MEF2C,FOSL1,RPS6KA2,WNK1,MAP3K3,MAP3K2
Role of Osteoblasts, Osteoclasts and Chondrocytes in Rheumatoid Arthritis	1.7783E-10	7.77E-08	MAP2K4,WNT10B,MAPK1,NFATC3,FZD3,PIK3R1,PPP3R1,MAPK3,MAP3K7,BIRC3,IL1RAP,FRS2,MAP3K14,TCF3,GSN,APC,GAB1,IRS1,FZD6,LEF1,WNT1,TCF4,BMP2,LRP6,BMPR2,MAP3K5,SMAD5,KLB,BCL2,SMURF1,NFAT5,NFKBIA,PPP3CB,BMPR1A,AKT3,CHUK,PPP3CA,SRC,LRP5,TNFSF11,SMAD6,MAPK9,TCF7L1,XIAP,CALM1 (includes others),WNT3A,FZD4,FOXO1,BMP7,BMP6,LRP1,IL11,FZD7,WNT5A
Breast Cancer Regulation by Stathmin1	1.9055E-10	8.33E-08	PRKACB,MAPK1,PPP2CA,PIK3R1,GNB5,KRAS,LIMK1,STMN1,GNB1,GNB4,MAPK3,PLCB1,ARHGEF11,FRS2,TP53,CCNE2,RRAS,ITPR1,PPP2R1A,GAB1,CDC42,PPP2R3A,RHOA,IRS1,E2F1,GNB2,PRKCH,ARHGEF10,E2F3,KLB,PPP1CC,PPP1R10,SOS1,RB1CC1,E2F5,PRKCE,PPP2R5C,ARHGEF3,MAP2K1,CAMK2B,GNAS,ADCY6,TSG101,GNAI3,CALM1 (includes others),CCNE1,TUBA1A,CDKN1A,PRKAG2,PPP2R1B

Production of Nitric Oxide and Reactive Oxygen Species in Macrophages	3.02E-10	1.32E-07	PPARA,MAP2K4,JAK1,MAP3K11,MAPK1,PPP2CA,PIK3R1,MAP3K5,JAK2,SPI1,KLB,MAP3K10,PPP1CC,NFKBIA,PPP1R10,RHOT1,MAPK3,HOXA10,MAP3K7,PRKCE,AKT3,PPP2R5C,CHUK,MAP2K1,FRS2,MAP3K2,MAP3K14,MAP3K9,RHOC,MAP3K13,MAPK9,PLCG1,PCYOX1,RAP1A,IRF1,MAP3K12,PPP2R1A,RND3,GAB1,PPP2R3A,IRS1,RHOA,MAP3K8,PRKCH,MAP3K3,PPP2R1B,CLU,SIRPA
Pancreatic Adenocarcinoma Signaling	5.7544E-10	2.51E-07	CDKN2A,MAP2K4,JAK1,TGFBR1,MAPK1,PIK3R1,CDK4,KRAS,JAK2,E2F3,CCND1,KLB,BCL2,TGFBR2,VEGFA,RB1,MAPK3,E2F5,AKT3,ERBB2,MAP2K1,FRS2,TP53,SMAD2,PLD3,HBEGF,MAPK9,STAT3,CCNE1,GAB1,CDC42,IRS1,CDKN1A,E2F1,NOTCH1
Prostate Cancer Signaling	7.7625E-10	3.39E-07	MAPK1,PIK3R1,KRAS,KLB,CCND1,EP300,BCL2,PTEN,RB1,MTOR,HSP90B1,NFKBIA,HSP90AB1,MAPK3,SOS1,AKT3,CHUK,MAP2K1,FRS2,TP53,CCNE2,RRAS,CREB5,CCNE1,FOXO1,GAB1,IRS1,CDKN1A,E2F1,LEF1
TGF-β Signaling	8.1283E-10	3.55E-07	MAP2K4,TGFBR1,MAPK1,BMP2,SKI,BMPR2,KRAS,SMAD5,EP300,BCL2,SMURF1,TGFBR2,BMPR1A,MAPK3,SOS1,MAP3K7,SERPINE1,HNF4A,MAP2K1,ZFYVE9,SMAD2,RRAS,HDAC1,SMAD7,SMAD6,MAPK9,ACVR1B,CDC42,BMP7
SAPK/JNK Signaling	8.5114E-10	3.72E-07	MAP2K4,DUSP8,MAP3K11,NFATC3,PIK3R1,KRAS,CRK,MAP3K5,KLB,GNB1,MAP3K10,SOS1,MAP3K7,FRS2,MAP3K2,TP53,SH2D2A,MAP3K9,RRAS,MAP3K13,CRKL,MAPK9,MINK1,DAXX,MAP3K12,GAB1,CDC42,IRS1,ZAK,DUSP4,MAP4K5,MAP3K3
GNRH Signaling	1.3183E-09	5.76E-07	MAP2K4,PRKACB,MAP3K11,MAPK1,KRAS,MAP3K5,EP300,MAP3K10,MAPK3,SOS1,MAP3K7,PLCB1,PRKCE,MAP2K1,DNM2,CAMK2B,MAP3K2,MAP3K9,MAP3K14,SRC,GNAS,RRAS,MAP3K13,ADCY6,MAPK9,DNM3,ITPR1,CREB5,MAP3K12,GNAI3,CDC42,PRKAG2,MAP3K8,PRKCH,MAP3K3,GNRHR
Factors Promoting Cardiogenesis in Vertebrates	1.5136E-09	6.61E-07	TCF4,TGFBR1,FZD3,BMP2,TGFBR3,LRP6,BMPR2,SMAD5,TGFBR2,BMPR1A,MAP3K7,PRKCE,SMAD2,LRP5,CCNE2,TCF7L1,TCF3,ACVR1B,APC,CCNE1,FZD4,FZD6,BMP7,MEF2C,PRKCH,LEF1,BMP6,LRP1,FZD7

PPARα/RXRα Activation	2.6303E-09	1.15E-06	PPARA,MAP2K4,PRKACB,TGFBR1,PRKAB2,MAPK1,TGFBR3,BMPR2,KRAS,JAK2,ADIPOR1,ABCA1,EP300,TGFBR2,HSP90B1,NFKBIA,CHD5,HSP90AB1,MAPK3,SOS1,MAP3K7,CLOCK,PLCB1,CHUK,GOT2,IL1RAP,MAP2K1,MAP3K14,SMAD2,GNAS,MED1,RRAS,ADCY6,PLCG1,NCOA6,PLCL2,NCOA3,ACVR1B,IRS1,PRKAG2,MEF2C,NCOR2
PKCθ Signaling in T Lymphocytes	3.1623E-09	1.38E-06	MAP2K4,FYN,MAP3K11,MAPK1,NFATC3,PIK3R1,HLA-DQA1,KRAS,MAP3K5,KLB,MAP3K10,NFKBIA,NFAT5,PPP3CB,PPP3R1,MAPK3,SOS1,MAP3K7,CHUK,FRS2,PPP3CA,CAMK2B,MAP3K2,MAP3K14,MAP3K9,RRAS,MAP3K13,PLCG1,MAP3K12,POU2F1,GAB1,IRS1,ZAP70,MAP3K8,MAP3K3
Cyclins and Cell Cycle Regulation	4.4668E-09	1.95E-06	CDKN2A,PPP2CA,CUL1,CDK4,CDKN2C,E2F3,CCND1,RB1,CCND3,E2F5,PPP2R5C,CDC25A,TP53,HDAC9,CCNE2,HDAC4,WEE1,HDAC1,CDK6,PPP2R1A,CCNE1,CCND2,PPP2R3A,E2F1,CDKN1A,PPP2R1B
Human Embryonic Stem Cell Pluripotency	6.4565E-09	2.82E-06	TCF4,TGFBR1,WNT10B,FZD3,PIK3R1,BMP2,BMPR2,SMAD5,KLB,SOX2,TGFBR2,BMPR1A,PDGFRA,AKT3,FRS2,SMAD2,GNAS,SMAD6,SMAD7,TCF7L1,TCF3,APC,NANOG,WNT3A,FZD4,FOXO1,GAB1,IRS1,S1PR1,FZD6,LEF1,BMP7,PDGFD,BMP6,WNT1,WNT5A,FZD7
Role of Tissue Factor in Cancer	1.1482E-08	5.02E-06	FYN,CTGF,MAPK1,PIK3R1,RPS6KA3,KRAS,JAK2,KLB,PTEN,LIMK1,VEGFA,MTOR,YES1,MAPK3,AKT3,PLCB1,RPS6KA2,FRS2,TP53,CXCL8,RPS6KB1,SRC,P4HB,RRAS,HBEGF,PLAUR,RPS6KA5,F3,GAB1,CDC42,IRS1,RPS6KA4,CYR61
AMPK Signaling	1.4454E-08	6.32E-06	PRKACB,CAB39,ARID1A,PRKAB2,MAPK1,PPP2CA,PIK3R1,SMARCD2,CCND1,KLB,EP300,MTOR,KAT2B,CRTC2,FOXO3,MAP3K7,AKT3,PPP2R5C,HNF4A,FRS2,ADRA1B,ADRB2,RPS6KB1,SRC,PFKFB3,GNAS,SLC2A1,ACTB,PFKP,CREB5,PPM1G,PPP2R1A,FOXO1,GAB1,PPM1B,PPP2R3A,IRS1,CDKN1A,SIRT1,PRKAG2,SMARCC1,PPP2R1B,HLTF,PHF10

Germ Cell-Sertoli Cell Junction Signaling	1.4791E-08	6.46E-06	MAP2K4,TGFBR1,MAP3K11,MAPK1,PIK3R1,KRAS,MAP3K5,KLB,LIMK1,TGFBR2,MAP3K10,AGGF1,RHOT1,MAPK3,MAP3K7,MTMR2,VCL,MAP2K1,FRS2,RAB8B,MAP3K2,MAP3K9,MAP3K14,SRC,RRAS,RHOC,MAP3K13,ACTB,MAPK9,GSN,PLS1,MAP3K12,TUBA1A,RND3,GAB1,CDC42,IRS1,RHOA,ZYX,MAP3K8,MAP3K3
Non-Small Cell Lung Cancer Signaling	2.1878E-08	9.56E-06	CDKN2A,MAPK1,PIK3R1,CDK4,KRAS,KLB,CCND1,RB1,STK4,MAPK3,SOS1,RARB,FOXO3,AKT3,ERBB2,MAP2K1,FRS2,TP53,RRAS,CDK6,PLCG1,ITPR1,GAB1,IRS1,E2F1
IGF-1 Signaling	3.1623E-08	1.38E-05	PRKACB,CSNK2A1,CTGF,JAK1,MAPK1,PIK3R1,SOCS6,SRF,KRAS,JAK2,KLB,YWHAQ,MAPK3,FOXO3,SOS1,IGF1R,AKT3,MAP2K1,FRS2,RPS6KB1,RRAS,YWHAB,YWHAZ,IGFBP5,STAT3,FOXO1,GAB1,IRS1,PRKAG2,CYR61
Renal Cell Carcinoma Signaling	3.8905E-08	1.70E-05	ETS1,SLC2A1,MAPK1,RRAS,PIK3R1,KRAS,CRK,HIF1A,RAP1A,KLB,EP300,MET,VEGFA,GAB1,CUL2,CDC42,IRS1,MAPK3,SOS1,AKT3,EGLN3,UBC,FRS2,MAP2K1,VHL
PDGF Signaling	3.9811E-08	1.74E-05	MAP2K4,CSNK2A1,JAK1,MAPK1,PIK3R1,SRF,KRAS,INPPL1,CRK,JAK2,KLB,OCRL,MYC,MAPK3,SOS1,CAV1,PDGFRA,MAP2K1,FRS2,SRC,RRAS,CRKL,PLCG1,STAT3,GAB1,IRS1,PDGFD
PTEN Signaling	4.2658E-08	1.86E-05	CSNK2A1,TGFBR1,MAPK1,PIK3R1,TGFBR3,BMPR2,INPPL1,KRAS,CCND1,OCRL,BCL2,PTEN,TGFBR2,BMPR1A,MAPK3,FOXO3,SOS1,IGF1R,PDGFRA,AKT3,CHUK,MAP2K1,ITGA4,RPS6KB1,FLT1,RRAS,IGF2R,MAGI1,FOXO1,CDC42,CDKN1A,MAGI3
Estrogen-mediated S-phase Entry	4.6774E-08	2.04E-05	MYC,RB1,CCNE2,CCNE1,E2F1,CDKN1A,E2F5,CDK4,RBL1,E2F3,CCND1,ESR1,CDC25A
Telomerase Signaling	5.0119E-08	2.19E-05	MAPK1,PPP2CA,PIK3R1,KRAS,KLB,MYC,RB1,HSP90B1,HSP90AB1,MAPK3,SOS1,AKT3,PPP2R5C,MAP2K1,FRS2,TP53,ETS1,HDAC9,HDAC4,RRAS,HDAC1,ELF2,PPP2R1A,GAB1,PPP2R3A,IRS1,E2F1,CDKN1A,ELK3,PPP2R1B

Superpathway of Inositol Phosphate Compounds	5.2481E-08	2.29E-05	FYN,DUSP8,PPP1R12C,PIK3R1,PIKFYVE,INPPL1,PIP5K1B,HACD2,OCRL,KLB,PTPN12,DUSP2,PXYLP1,PTEN,SACM1L,CDC25B,SET,PPP1CC,ITPKB,PTPRJ,PTPN1,PDGFRA,DUSP11,PLCB1,MTMR2,ERBB2,RNGTT,FRS2,PPP3CA,CDC25A,WBP11,TMEM55B,PLCG1,ERBB3,PAWR,ITPKA,PPP4R1,DUSP14,CALM1 (includes others),MTMR4,TMEM55A,GAB1,PPP2R3A,IRS1,PPIP5K2,PIP4K2C,SIRPA
Axonal Guidance Signaling	5.4954E-08	2.40E-05	DPYSL2,PRKACB,KLC1,WNT10B,MAPK1,NFATC3,FZD3,PIK3R1,GNB5,KRAS,LIMK1,GNB1,VEGFA,GNB4,SEMA6D,MAPK3,PPP3R1,PLCB1,ARHGEF11,FRS2,ITGA4,EPHA7,RRAS,CRKL,MMP2,PLCL2,DPYSL5,RAP1A,MET,SDCBP,ARPC1A,GAB1,CDC42,RHOA,IRS1,FZD6,GNB2,PRKCH,PDGFD,WNT1,FYN,BMP2,EPHA4,CRK,KLB,SEMA4C,EFNB2,NFAT5,GLI3,PPP3CB,SDC2,SOS1,PFN2,AKT3,PRKCE,ERBB2,SHANK2,MAP2K1,VASP,PPP3CA,GNAS,NRP2,CXCR4,ARPC5L,PLCG1,SLIT2,EFNA1,GNAI3,TUBA1A,FZD4,WNT3A,PRKAG2,EPHA5,BMP7,BMP6,ADAM9,FZD7,WNT5A
Neuregulin Signaling	5.8884E-08	2.57E-05	MAPK1,PIK3R1,KRAS,CRK,PTEN,MYC,MTOR,HSP90B1,HSP90AB1,MAPK3,SOS1,PRKCE,AKT3,ERBB2,MAP2K1,ITGA4,RPS6KB1,SRC,RRAS,ERBIN,CRKL,PLCG1,HBEGF,ERBB3,PRKCH,EREG
Phospholipase C Signaling	6.4565E-08	2.82E-05	MAPK1,NFATC3,GNB5,KRAS,GNB1,GNB4,PPP1R12B,MAPK3,PPP3R1,PLCB1,ARHGEF11,ITGA4,HDAC4,PLD3,RRAS,ITPR1,CREB5,RAP1A,MARCKS,RND3,RHOA,ZAP70,GNB2,PRKCH,ARHGEF10,PEBP1,FYN,RPS6KA3,EP300,NFAT5,AHNAK,PPP3CB,RHOT1,SOS1,PRKCE,ARHGEF3,MAP2K1,PPP3CA,SRC,HDAC9,GNAS,RHOC,HDAC1,ADCY6,PLCG1,PLA2G4A,CALM1 (includes others),MEF2D,MEF2C
Melanoma Signaling	6.4565E-08	2.82E-05	TP53,CDKN2A,MAPK1,RRAS,MITF,PIK3R1,CDK4,KRAS,KLB,CCND1,PTEN,RB1,GAB1,IRS1,MAPK3,CDKN1A,E2F1,AKT3,MAP2K1,FRS2

Sertoli Cell-Sertoli Cell Junction Signaling	9.1201E-08	3.99E-05	MAP2K4,PRKACB,SPTBN1,MAP3K11,MAPK1,TGFBR3,KRAS,MAP3K5,PTEN,OCLN,MAP3K10,AGGF1,MAPK3,MAP3K7,AKT3,MTMR2,VCL,MAP2K1,RAB8B,ITGA4,MAP3K2,SPTBN2,SRC,MAP3K14,DLG1,MAP3K9,GUCY1A3,RRAS,MAP3K13,ACTB,MAPK9,PLS1,EPB41,MAP3K12,TUBA1A,CDC42,ZAK,PRKAG2,MAP3K8,MAP3K3
Aryl Hydrocarbon Receptor Signaling	9.3325E-08	4.08E-05	CDKN2A,TRIP11,MAPK1,TP73,CDK4,RBL1,CCND1,FAS,EP300,CHEK1,MYC,RB1,HSP90B1,HSP90AB1,CCND3,MAPK3,NEDD8,RARB,TP53,SRC,CCNE2,RBL2,MED1,CDK6,NCOA3,ALDH9A1,CCNE1,CCND2,CDKN1A,E2F1,ALDH3B1,NFIB,NCOR2,ESR1
HER-2 Signaling in Breast Cancer	0.0000001	4.37E-05	TP53,CCNE2,RRAS,PIK3R1,CDK6,PLCG1,PARD6B,MMP2,KRAS,ERBB3,ITGB8,MAP3K5,CCND1,KLB,CCNE1,FOXO1,GAB1,CDC42,IRS1,CDKN1A,SOS1,AKT3,PRKCE,PRKCH,ERBB2,FRS2
Epithelial Adherens Junction Signaling	1.3183E-07	5.76E-05	TCF4,TGFBR1,MYH9,TGFBR3,BMPR2,KRAS,CRK,PTEN,TGFBR2,NOTCH2,YES1,AGGF1,AKT3,VCL,SRC,DLL1,LMO7,RRAS,ARPC5L,ACTB,TCF7L1,TCF3,RAP1A,ACVR1B,APC,MET,ARPC1A,MAGI1,TUBA1A,CDC42,RHOA,SSX2IP,ZYX,LEF1,NOTCH1
Melanocyte Development and Pigmentation Signaling	1.3804E-07	6.03E-05	PRKACB,MAPK1,PIK3R1,RPS6KA3,CRK,KRAS,KLB,EP300,BCL2,MAPK3,SOS1,RPS6KA2,MAP2K1,FRS2,RPS6KB1,SRC,GNAS,RRAS,MITF,ADCY6,PLCG1,RPS6KA5,CREB5,GAB1,IRS1,PRKAG2,RPS6KA4
GM-CSF Signaling	1.4791E-07	6.46E-05	ETS1,RUNX1,MAPK1,RRAS,PIK3R1,KRAS,JAK2,STAT3,KLB,CCND1,GAB1,PPP3CB,PIM1,CSF2RA,IRS1,MAPK3,PPP3R1,SOS1,AKT3,MAP2K1,FRS2,PPP3CA,CAMK2B
IL-8 Signaling	1.5136E-07	6.61E-05	MAP2K4,MAPK1,PIK3R1,GNB5,KRAS,CCND1,KLB,BCL2,LIMK1,VEGFA,GNB1,GNB4,MTOR,CCND3,RHOT1,MAPK3,PRKCE,AKT3,CHUK,FRS2,MAP2K1,LASP1,VASP,RPS6KB1,CXCL8,SRC,PLD3,GNAS,FLT1,RRAS,RHOC,MAPK9,HBEGF

			,MMP2,GNAI3,CCND2,RND3,GAB1,IRS1,RHOA,GNB2,PRKCH,IRAK2
Acute Myeloid Leukemia Signaling	1.6218E-07	7.09E-05	RUNX1,MAP2K4,TCF4,MAPK1,PIK3R1,KRAS,KLB,CCND1,SPI1,MYC,MTOR,PIM1,CSF2RA,MAPK3,SOS1,AKT3,MAP2K1,FRS2,RPS6KB1,RRAS,STAT3,TCF7L1,TCF3,GAB1,IRS1,LEF1
Huntington's Disease Signaling	1.6982E-07	7.42E-05	MAP2K4,MAPK1,PIK3R1,HSPA1A/HSPA1B,GNB5,KLB,EP300,GNB1,GNB4,MAP3K10,MTOR,MAPK3,SOS1,IGF1R,PRKCE,AKT3,PLCB1,DNAJB1,GOSR1,CASP8,FRS2,DNM2,TP53,HDAC9,HDAC4,RCOR3,HDAC1,CLTC,TBP,RCOR1,MAPK9,POLR2H,DNM3,ITPR1,STX1A,CREB5,ZDHHC17,HSPA8,PSME1,ATP5B,GAB1,CASP2,IRS1,CLTA,GNB2,PRKCH,NCOR2,UBC,CASP7
Hereditary Breast Cancer Signaling	1.9953E-07	8.72E-05	NPM1,ARID1A,PIK3R1,CDK4,SMARCD2,KRAS,CCND1,KLB,EP300,PTEN,CHEK1,RB1,RFC2,AKT3,FRS2,TP53,HDAC9,HDAC4,RRAS,WEE1,ACTB,HDAC1,CDK6,POLR2H,RFC1,GAB1,IRS1,CDKN1A,E2F1,SMARCC1,UBC,HLTF,RFC3,PHF10
Ephrin Receptor Signaling	2.2909E-07	0.00010011	FYN,MAPK1,GNB5,KRAS,CRK,EPHA4,JAK2,EP300,LIMK1,VEGFA,GNB1,GNB4,EFNB2,SDC2,MAPK3,SOS1,AKT3,MAP2K1,ITGA4,EPHA7,MAP3K14,SRC,GNAS,CXCR4,ARPC5L,RRAS,CRKL,STAT3,CREB5,RAP1A,EFNA1,GNAI3,ARPC1A,SDCBP,CDC42,RHOA,GNB2,EPHA5,PDGFD
Cell Cycle Regulation by BTG Family Proteins	2.3442E-07	0.00010244	CCNE2,PPP2CA,CDK4,BTG1,E2F3,CCND1,RB1,CCNE1,PPP2R1A,PPP2R3A,BTG2,E2F1,E2F5,PPP2R5C,PPP2R1B
CNTF Signaling	3.3884E-07	0.00014807	RPS6KB1,JAK1,MAPK1,RRAS,PIK3R1,RPS6KA3,RPS6KA5,KRAS,STAT3,JAK2,KLB,MTOR,GAB1,IRS1,MAPK3,SOS1,RPS6KA4,RPS6KA2,MAP2K1,FRS2

UVA-Induced MAPK Signaling	5.3703E-07	0.00023468	MAP2K4,PARP6,MAPK1,PIK3R1,PARP2,RPS6KA3,KRAS,KLB,MTOR,TIPARP,MAPK3,PLCB1,RPS6KA2,FRS2,PARP14,TP53,RPS6KB1,RRAS,ZC3HAV1,MAPK9,PLCG1,TNKS2,RPS6KA5,PLCL2,GAB1,IRS1,RPS6KA4
Regulation of IL-2 Expression in Activated and Anergic T Lymphocytes	5.6234E-07	0.00024574	MAP2K4,SMAD2,FYN,TGFBR1,MAPK1,NFATC3,RRAS,TOB1,PLCG1,MAPK9,KRAS,TGFBR2,CALM1 (includes others),NFAT5,NFKBIA,PPP3CB,MAPK3,PPP3R1,SOS1,ZAP70,CHUK,MAP2K1,PPP3CA
fMLP Signaling in Neutrophils	6.166E-07	0.00026945	MAPK1,NFATC3,PIK3R1,GNB5,KRAS,KLB,GNB1,GNB4,NFAT5,NFKBIA,PPP3CB,PPP3R1,MAPK3,PLCB1,PRKCE,MAP2K1,FRS2,PPP3CA,GNAS,RRAS,ARPC5L,ITPR1,CALM1 (includes others),GNAI3,ARPC1A,GAB1,CDC42,IRS1,GNB2,PRKCH
Death Receptor Signaling	8.1283E-07	0.00035521	TNFRSF21,MAP2K4,MAP3K14,PARP6,ACTB,TNFRSF10B,PARP2,ZC3HAV1,LMNA,TNKS2,MAP3K5,ARHGDIB,XIAP,FAS,LIMK1,BCL2,DAXX,NFKBIA,TIPARP,CASP2,CHUK,CASP8,BIRC3,CASP7,PARP14
p70S6K Signaling	1.2303E-06	0.00053763	JAK1,F2R,MAPK1,PPP2CA,PIK3R1,KRAS,KLB,YWHAQ,MTOR,MAPK3,SOS1,PRKCE,AKT3,PLCB1,PPP2R5C,MAP2K1,FRS2,RPS6KB1,SRC,YWHAB,RRAS,YWHAZ,PLCG1,PLCL2,GNAI3,PPP2R1A,GAB1,PPP2R3A,IRS1,PRKCH,PPP2R1B
Myc Mediated Apoptosis Signaling	1.2589E-06	0.00055015	TP53,MAP2K4,CDKN2A,YWHAB,RRAS,PIK3R1,YWHAZ,MAPK9,KRAS,KLB,FAS,BCL2,YWHAQ,MYC,GAB1,IRS1,SOS1,IGF1R,AKT3,CASP8,FRS2
Protein Ubiquitination Pathway	1.2882E-06	0.00056297	DNAJB4,HLA-B,HSPA1A/HSPA1B,FBXW7,SACS,UBE2B,NEDD4L,DNAJC16,BIRC3,PSMB5,PSMD5,HSPA8,PSMD11,UBE2D2,USP32,DNAJB6,UBE2E1,VHL,USP24,USP14,USP53,UBE2N,CUL1,DNAJC12,CDC23,SMURF1,UBE2F,USP3,HSP90B1,HSP90AB1,PSMC6,USP47,USP16,DNAJB1,HSPA4L,PSMA1,DNAJB9,UBE3A,USP33,UBE2D1,XIAP,PSME1,UBE2J1,HLA-C,CUL2,USP46,UBC,USP34,DNAJC7

Insulin Receptor Signaling	1.349E-06	0.0005895	PRKACB,FYN,JAK1,MAPK1,PIK3R1,INPPL1,KRAS,CRK,JAK2,OCRL,KLB,PTEN,PPP1CC,MTOR,PPP1R10,PDE3B,MAPK3,PTPN1,FOXO3,SOS1,AKT3,MAP2K1,FRS2,RPS6KB1,RRAS,CRKL,VAMP2,FOXO1,GAB1,IRS1,ASIC1,PRKAG2
Integrin Signaling	1.4454E-06	0.00063166	MAP2K4,FYN,RAP2A,ARHGAP26,MAP3K11,MAPK1,PIK3R1,PIKFYVE,KRAS,CRK,ITGB8,KLB,PTEN,PPP1R12B,RHOT1,MAPK3,SOS1,CAV1,AKT3,PFN2,VCL,FRS2,MAP2K1,VASP,ITGA4,SRC,ARPC5L,RRAS,RHOC,CRKL,ACTB,PLCG1,GSN,RAP1A,ARHGAP5,ARPC1A,RND3,GAB1,ARF3,CDC42,IRS1,RHOA,ZYX
Prolactin Signaling	1.8621E-06	0.00081373	FYN,MAPK1,RRAS,PIK3R1,SOCS6,PLCG1,KRAS,JAK2,STAT3,CEBPB,NR3C1,KLB,IRF1,EP300,MYC,GAB1,IRS1,MAPK3,SOS1,PRKCE,PRKCH,MAP2K1,FRS2
Small Cell Lung Cancer Signaling	2.3442E-06	0.00102443	TP53,CCNE2,PIK3R1,CDK4,CDK6,KLB,CCND1,PTEN,BCL2,MYC,RB1,PIAS3,CCNE1,MAX,NFKBIA,GAB1,IRS1,TRAF4,E2F1,RARB,AKT3,CHUK,FRS2
PEDF Signaling	2.3442E-06	0.00102443	TP53,PPARG,TCF4,MAPK1,RRAS,PIK3R1,SRF,KRAS,ZEB1,KLB,FAS,BCL2,TCF12,NFKBIA,GAB1,IRS1,MAPK3,RHOA,AKT3,CHUK,CASP8,FRS2,CASP7
CD27 Signaling in Lymphocytes	2.5704E-06	0.00112326	MAP2K4,MAP3K9,MAP3K14,MAP3K11,MAP3K13,MAPK9,MAP3K5,MAP3K10,MAP3K12,NFKBIA,MAP3K7,MAP3K8,CHUK,CASP8,MAP3K3,MAP2K1,MAP3K2
HIPPO signaling	2.884E-06	0.00126032	DLG1,SMAD2,SAV1,FAT4,YWHAB,PPP2CA,CUL1,YWHAZ,SMAD5,YWHAQ,ITCH,STK4,PPP2R1A,PPP1CC,DLG5,PPP1R10,WWC1,PPP2R3A,CD44,PPP2R5C,AMOT,PPP2R1B,LATS1
EGF Signaling	3.1623E-06	0.00138192	MAP2K4,RPS6KB1,SRC,CSNK2A1,JAK1,MAPK1,PIK3R1,SRF,PLCG1,ITPR1,STAT3,KLB,MTOR,GAB1,IRS1,MAPK3,SOS1,AKT3,MAP2K1,FRS2
BMP signaling pathway	3.3884E-06	0.00148075	MAP2K4,PRKACB,MAPK1,RRAS,BMP2,SMAD6,SMAD7,BMPR2,MAPK9,KRAS,SMAD5,XIAP,SMURF1,BMPR1A,MAPK3,SOS1,PRKAG2,MAP3K7,BMP7,BMP6,MAP2K1

CTLA4 Signaling in Cytotoxic T Lymphocytes	3.5481E-06	0.00155053	FYN,AP2A1,PPP2CA,PIK3R1,AP2B1,HLA-B,JAK2,CD8B,KLB,AKT3,PPP2R5C,FRS2,AP2M1,AP1S2,CLTC,PLCG1,AP1S1,PPP2R1A,GAB1,HLA-C,PPP2R3A,IRS1,CLTA,ZAP70,PPP2R1B
3-phosphoinositide Biosynthesis	3.6308E-06	0.00158665	FYN,PPP1R12C,DUSP8,PIK3R1,PIKFYVE,PIP5K1B,HACD2,KLB,PTPN12,PXYLP1,DUSP2,PTEN,SACM1L,CDC25B,SET,PPP1CC,PTPRJ,PTPN1,PDGFRA,DUSP11,MTMR2,ERBB2,RNGTT,FRS2,PPP3CA,CDC25A,WBP11,PAWR,ERBB3,PPP4R1,DUSP14,MTMR4,GAB1,PPP2R3A,IRS1,PIP4K2C,SIRPA
NF-κB Signaling	3.6308E-06	0.00158665	PRKACB,CSNK2A1,TGFBR1,TGFBR3,PIK3R1,UBE2N,BMP2,BMPR2,TNFAIP3,KRAS,KLB,EP300,TGFBR2,NFKBIA,BMPR1A,MAP3K7,IGF1R,PDGFRA,AKT3,LTBR,CHUK,CASP8,FRS2,MAP3K14,TNFSF11,FLT1,RRAS,RELB,HDAC1,IGF2R,TAB3,CD40,GAB1,IRS1,ZAP70,MAP3K8,MAP3K3
Hypoxia Signaling in the Cardiovascular System	3.6308E-06	0.00158665	TP53,P4HB,UBE2N,HIF1A,CREB5,UBE2D1,UBE2F,PTEN,EP300,VEGFA,UBE2D2,HSP90B1,UBE2J1,NFKBIA,UBE2B,HSP90AB1,LDHA,VHL,UBE2E1
Endometrial Cancer Signaling	4.7863E-06	0.00209161	TP53,MAPK1,RRAS,PIK3R1,KRAS,CCND1,KLB,PTEN,MYC,GAB1,MAPK3,IRS1,SOS1,FOXO3,AKT3,LEF1,ERBB2,FRS2,MAP2K1
Glioma Invasiveness Signaling	5.1286E-06	0.0022412	TIMP3,MAPK1,F2R,RHOC,RRAS,PIK3R1,PLAUR,KRAS,MMP2,KLB,GAB1,RND3,RHOT1,IRS1,MAPK3,RHOA,CD44,PLAU,FRS2,TIMP2
RAR Activation	5.4954E-06	0.00240149	MAP2K4,PRKACB,CSNK2A1,ARID1A,MAPK1,PIK3R1,BMP2,NR2F2,SMARCD2,JAK2,MAP3K5,SMAD5,PTEN,EP300,VEGFA,KAT2B,PNRC1,RARB,PRKCE,AKT3,MAP2K1,CITED2,SMAD2,SRC,MED1,ACTB,RELB,ADCY6,SMAD7,SMAD6,MAPK9,PRKAG2,PRKCH,NCOR2,SMARCC1,HLTF,CARM1,PHF10
mTOR Signaling	6.166E-06	0.00269452	PRKAB2,MAPK1,PPP2CA,PIK3R1,RPS6KA3,FKBP1A,KRAS,EIF4A2,HIF1A,KLB,VEGFA,MTOR,EIF4G2,RHOT1,MAPK3,PRKCE,AKT3,PPP2R5C,RPS6KA2,FRS2,RPS6KB1,PLD3,RHOC,RRAS,RPS10,EIF4G3,RPS6KA5,RPS29,PPP2R1A,RND3,GAB1,PPP2R3A,IRS1,RHOA,EIF4A1,PRKAG2,RPS6KA4,PRKCH,PPP2R1B

IL-3 Signaling	6.9183E-06	0.0030233	JAK1,MAPK1,RRAS,CRKL,PIK3R1,KRAS,JAK2,STAT3,KLB,GAB1,PPP3CB,FOXO1,IRS1,MAPK3,PPP3R1,SOS1,AKT3,PRKCE,PRKCH,MAP2K1,FRS2,PPP3CA
Virus Entry via Endocytic Pathways	7.5858E-06	0.00331499	SRC,FYN,AP2A1,AP2M1,RRAS,AP2B1,ACTB,PIK3R1,HLA-B,CLTC,PLCG1,KRAS,ITGB8,KLB,GAB1,HLA-C,CDC42,IRS1,CLTA,CAV1,PRKCE,PRKCH,FRS2,DNM2,ITGA4
Role of CHK Proteins in Cell Cycle Checkpoint Control	8.1283E-06	0.00355207	TP53,PPP2CA,RFC1,E2F3,CHEK1,PPP2R1A,PPP2R3A,RAD17,E2F1,CDKN1A,RFC2,E2F5,TLK1,PPP2R5C,PPP2R1B,RFC3,CDC25A
ErbB Signaling	9.7724E-06	0.00427053	MAP2K4,RPS6KB1,MAPK1,RRAS,PIK3R1,HBEGF,PLCG1,MAPK9,KRAS,ERBB3,KLB,MTOR,FOXO1,GAB1,CDC42,IRS1,MAPK3,SOS1,PRKCE,PRKCH,ERBB2,MAP2K1,FRS2,EREG
Role of NFAT in Regulation of the Immune Response	1.0715E-05	0.00468254	FYN,CSNK1G1,MAPK1,NFATC3,PIK3R1,GNB5,HLA-DQA1,KRAS,KLB,GNB1,GNB4,NFKBIA,NFAT5,PPP3CB,PPP3R1,MAPK3,SOS1,AKT3,PLCB1,CHUK,MAP2K1,FRS2,PPP3CA,AKAP5,GNAS,RRAS,PLCG1,ITPR1,GNAI3,CALM1 (includes others),GAB1,IRS1,MEF2D,ZAP70,GNB2,MEF2C
Gap Junction Signaling	1.0715E-05	0.00468254	PRKACB,CSNK1G1,MAPK1,PIK3R1,KRAS,KLB,SP3,PPP3CB,MAPK3,PPP3R1,SOS1,CAV1,PLCB1,AKT3,PRKCE,MAP2K1,FRS2,PPP3CA,MAP3K2,SRC,GNAS,GUCY1A3,RRAS,ACTB,ADCY6,PLCG1,PLCL2,ITPR1,GNAI3,TUBA1A,GAB1,IRS1,PRKAG2,PRKCH
p38 MAPK Signaling	1.122E-05	0.00490323	MAP2K4,TGFBR1,DDIT3,RPS6KA3,SRF,MAP3K5,FAS,EP300,CDC25B,TGFBR2,MYC,MAP3K7,RPS6KA2,IL1RAP,TP53,RPS6KB1,MAPKAPK5,RPS6KA5,CREB5,PLA2G4A,DAXX,H3F3A/H3F3B,MAX,MEF2D,MEF2C,RPS6KA4,IRAK2
Thrombin Signaling	1.1482E-05	0.00501742	F2R,MAPK1,PIK3R1,GNB5,KRAS,KLB,GNB1,GNB4,PPP1R12B,RHOT1,MAPK3,SOS1,PRKCE,AKT3,PLCB1,GATA6,ARHGEF11,ARHGEF3,MAP2K1,FRS2,CAMK2B,RPS6KB1,SRC,GNAS,RRAS,RHOC,ADCY6,TBP,PLCG1,PLCL2,ITPR1,GNAI

			3,RND3,GAB1,IRS1,RHOA,GNB2,PRKCH,ARHGEF10
Gαq Signaling	1.2303E-05	0.00537628	MAPK1,NFATC3,PIK3R1,GNB5,KLB,GNB1,GNB4,NFKBIA,PPP3CB,RHOT1,MAPK3,PPP3R1,PRKCE,AKT3,PLCB1,CHUK,MAP2K1,FRS2,ADRA1B,PPP3CA,RGS2,PLD3,GNAS,RHOC,PLCG1,ITPR1,CALM1 (includes others),RND3,GAB1,IRS1,RHOA,GNB2,PRKCH
Reelin Signaling in Neurons	1.2303E-05	0.00537628	PAFAH1B2,MAP2K4,FYN,MAP3K9,SRC,MAP3K11,CRKL,PIK3R1,MAPK9,VLDLR,KLB,APP,MAP3K10,YES1,NDEL1,GAB1,IRS1,ARHGEF11,ARHGEF3,PAFAH1B1,FRS2,ARHGEF10,ITGA4
Aldosterone Signaling in Epithelial Cells	1.2303E-05	0.00537628	ICMT,MAPK1,DNAJB4,PIK3R1,DNAJC12,HSPA1A/HSPA1B,PIKFYVE,KRAS,PIP5K1B,KLB,SACS,HSP90B1,HSP90AB1,MAPK3,SOS1,PRKCE,PLCB1,DNAJB1,MAP2K1,DNAJC16,FRS2,HSPA4L,PLCG1,PLCL2,ITPR1,DNAJB9,HSPA8,GAB1,IRS1,ASIC1,PRKCH,DNAJB6,PIP4K2C,DNAJC7
3-phosphoinositide Degradation	1.5488E-05	0.00676834	PPP1R12C,DUSP8,INPPL1,HACD2,OCRL,PTPN12,DUSP2,PXYLP1,SACM1L,PTEN,CDC25B,SET,PPP1CC,PTPRJ,PTPN1,DUSP11,MTMR2,RNGTT,PPP3CA,CDC25A,MTMR3,WBP11,TMEM55B,PAWR,PPP4R1,DUSP14,MTMR4,TMEM55A,PPP2R3A,SIRPA
Basal Cell Carcinoma Signaling	1.5849E-05	0.00692597	TP53,TCF4,WNT10B,FZD3,BMP2,TCF7L1,TCF3,APC,WNT3A,FZD4,GLI3,FZD6,BMP7,DVL3,LEF1,BMP6,WNT1,WNT5A,FZD7
VEGF Signaling	1.6982E-05	0.00742131	SH2D2A,SRC,FLT1,MAPK1,RRAS,ACTB,PIK3R1,PLCG1,KRAS,HIF1A,EIF2S1,KLB,BCL2,VEGFA,FOXO1,GAB1,IRS1,MAPK3,SOS1,FOXO3,AKT3,VCL,MAP2K1,FRS2
Clathrin-mediated Endocytosis Signaling	1.6982E-05	0.00742131	AP2A1,CSNK2A1,EPS15,F2R,PIK3R1,AP2B1,ITGB8,RAB5B,KLB,VEGFA,PPP3CB,PPP3R1,AAK1,DAB2,LDLRAP1,SH3KBP1,FRS2,DNM2,PPP3CA,SRC,AP2M1,ARPC5L,ACTB,CLTC,DNM3,PCYOX1,TSG101,MET,HSPA8,LDLR,ARPC1A,GAB1

			,CDC42,CLTA,IRS1,UBC,PDGFD,CLU
D-myo-inositol-5-phosphate Metabolism	1.7783E-05	0.00777108	PPP1R12C,DUSP8,HACD2,PTPN12,DUSP2,PXYLP1,SACM1L,PTEN,CDC25B,SET,PPP1CC,PTPRJ,PTPN1,DUSP11,PLCB1,MTMR2,RNGTT,PPP3CA,CDC25A,WBP11,TMEM55B,PLCG1,PAWR,PPP4R1,DUSP14,MTMR4,TMEM55A,PPP2R3A,PIP4K2C,SIRPA
G Beta Gamma Signaling	1.9055E-05	0.00832686	PRKACB,SRC,GNAS,MAPK1,RRAS,GNB5,HBEGF,PLCG1,KRAS,GNB1,GNB4,GNAI3,CDC42,MAPK3,SOS1,CAV1,GNB2,PRKAG2,AKT3,PRKCE,PRKCH,DNM2
Apoptosis Signaling	1.9055E-05	0.00832686	TP53,MAP2K4,MAP3K14,MAPK1,RRAS,LMNA,PLCG1,KRAS,MAP3K5,FAS,XIAP,BCL2,NFKBIA,MAPK3,CASP2,PRKCE,CHUK,CASP8,MAP2K1,BIRC3,CASP7,MCL1
FLT3 Signaling in Hematopoietic Progenitor Cells	1.9498E-05	0.0085208	RPS6KB1,MAPK1,RRAS,PIK3R1,RPS6KA3,RPS6KA5,KRAS,STAT3,CREB5,KLB,EP300,MTOR,GAB1,IRS1,MAPK3,SOS1,AKT3,RPS6KA4,RPS6KA2,MAP2K1,FRS2
Inhibition of Angiogenesis by TSP1	1.9953E-05	0.00871929	MAP2K4,VEGFA,TP53,TGFBR2,FYN,TGFBR1,GUCY1A3,MAPK1,SDC2,THBS1,AKT3,MAPK9
T Cell Receptor Signaling	2.1878E-05	0.00956051	MAP2K4,FYN,MAPK1,NFATC3,PIK3R1,KRAS,CD8B,KLB,NFKBIA,NFAT5,PPP3CB,PPP3R1,MAPK3,SOS1,CHUK,MAP2K1,FRS2,PPP3CA,RRAS,PLCG1,CALM1 (includes others),SHB,GAB1,IRS1,ZAP70
JAK/Stat Signaling	2.3988E-05	0.01048289	JAK1,MAPK1,RRAS,PIK3R1,SOCS6,KRAS,JAK2,STAT3,CEBPB,KLB,PIAS3,MTOR,GAB1,IRS1,MAPK3,CDKN1A,SOS1,PTPN1,AKT3,MAP2K1,FRS2
ILK Signaling	2.5119E-05	0.01097696	MAP2K4,MYH9,MAPK1,PPP2CA,PIK3R1,BMP2,HIF1A,ITGB8,KLB,CCND1,EP300,PTEN,MYC,VEGFA,MTOR,TGFB1I1,RHOT1,MAPK3,AKT3,PPP2R5C,VCL,FRS2,RHOC,ACTB,MAPK9,RPS6KA5,CREB5,PPP2R1A,RND3,GAB1,PPP2R3A,CDC4

			2,IRS1,RHOA,RPS6KA4,LEF1,PPP2R1B
14-3-3-mediated Signaling	2.6303E-05	0.01149428	MAP2K4,MAPK1,TP73,PIK3R1,KRAS,MAP3K5,KLB,YWHAQ,SRPK2,MAPK3,AKT3,PLCB1,PRKCE,PDCD6IP,MAP2K1,FRS2,SRC,RRAS,YWHAB,YWHAZ,MAPK9,PLCG1,PLCL2,TUBA1A,FOXO1,GAB1,IRS1,PRKCH
PPAR Signaling	2.7542E-05	0.01203599	PPARG,PPARA,MAP3K14,MAPK1,PPARD,RRAS,MED1,KRAS,EP300,HSP90B1,NFKBIA,HSP90AB1,MAPK3,SOS1,PDGFRA,MAP3K7,CHUK,NCOR2,PDGFD,MAP2K1,IL1RAP,CITED2
Role of BRCA1 in DNA Damage Response	3.02E-05	0.01319718	TP53,RBL2,ARID1A,TOPBP1,ACTB,SMARCD2,RFC1,RBL1,E2F3,CHEK1,RB1,POU2F1,CDKN1A,E2F1,E2F5,RFC2,SMARCC1,HLTF,RFC3,PHF10
UVB-Induced MAPK Signaling	3.02E-05	0.01319718	TP53,MAP2K4,RPS6KB1,MAPK1,PIK3R1,RPS6KA3,MAPK9,RPS6KA5,KLB,MTOR,H3F3A/H3F3B,GAB1,MAPK3,IRS1,PRKCE,PRKCH,FRS2,MAP2K1
Role of Wnt/GSK-3β Signaling in the Pathogenesis of Influenza	3.0903E-05	0.01350461	SIAH1,TCF4,WNT10B,CSNK1G1,FZD3,IFNB1,TCF7L1,TCF3,NCOA3,APC,NCOA4,FZD4,WNT3A,FZD6,DVL3,LEF1,WNT1,WNT5A,FZD7
RhoGDI Signaling	3.0903E-05	0.01350461	PPP1R12C,PIKFYVE,GNB5,PIP5K1B,EP300,LIMK1,GNB1,GNB4,PPP1R12B,RHOT1,ARHGAP12,ARHGEF11,ARHGEF3,ITGA4,SRC,GNAS,RHOC,ARPC5L,ACTB,ARHGDIB,ARHGAP5,GNAI3,ARPC1A,CDH5,RND3,CDC42,RHOA,GNB2,CD44,ARHGAP35,ARHGEF10,ARHGAP1,ESR1,PIP4K2C
Xenobiotic Metabolism Signaling	3.2359E-05	0.01414106	MAP2K4,MAP3K11,MAPK1,PPP2CA,PIK3R1,HS2ST1,KRAS,MAP3K5,KLB,HS3ST3A1,EP300,CHST2,CUL3,MAP3K10,HSP90B1,HSP90AB1,MAPK3,MAP3K7,PRKCE,PPP2R5C,FRS2,MAP2K1,CITED2,CAMK2B,MAP3K2,MAP3K9,MAP3K14,HDAC4,MED1,RRAS,MAP3K13,MAPK9,CHST12,ALDH9A1,MAP3K12,PPP2R1A,GA

			B1,PPP2R3A,IRS1,ALDH3B1,MAP3K8,PRKCH,NCOR2,MAP3K3,PPP2R1B,EIF2AK3,DNAJC7
TR/RXR Activation	3.7154E-05	0.01623608	AKR1C1/AKR1C2,SLC2A1,MED1,PIK3R1,NCOA6,THRA,PFKP,HIF1A,KLB,NCOA3,NCOA4,EP300,KLF9,MTOR,LDLR,SCARB1,GAB1,PDE3B,IRS1,AKT3,NCOR2,TBL1XR1,FRS2
FAK Signaling	3.7154E-05	0.01623608	SRC,FYN,ARHGAP26,MAPK1,RRAS,ACTB,PIK3R1,PLCG1,KRAS,CRK,KLB,PTEN,GIT2,GAB1,IRS1,MAPK3,SOS1,AKT3,VCL,MAP2K1,TNS1,FRS2,ITGA4
Thyroid Cancer Signaling	3.8905E-05	0.01700127	PPARG,TP53,TCF4,MAPK1,RRAS,KRAS,TCF7L1,TCF3,CCND1,MYC,MAPK3,LEF1,MAP2K1
Type II Diabetes Mellitus Signaling	3.9811E-05	0.01739728	MAP2K4,MAPK1,PRKAB2,PIK3R1,SOCS6,MAP3K5,KLB,ADIPOR1,MTOR,NFKBIA,MAPK3,MAP3K7,AKT3,PRKCE,ACSL4,CHUK,FRS2,NSMAF,PPARG,MAP3K14,MAPK9,CEBPB,GAB1,IRS1,PRKAG2,PRKCH,ACSL1
Oncostatin M Signaling	3.9811E-05	0.01739728	TIMP3,EPAS1,JAK1,MAPK1,RRAS,MAPK3,SOS1,KRAS,STAT3,PLAU,JAK2,MAP2K1
LPS-stimulated MAPK Signaling	4.2658E-05	0.01864155	MAP2K4,MAP3K14,MAPK1,RRAS,PIK3R1,SRF,MAPK9,KRAS,MAP3K5,KLB,NFKBIA,GAB1,CDC42,IRS1,MAPK3,MAP3K7,PRKCE,PRKCH,CHUK,MAP2K1,FRS2
CREB Signaling in Neurons	4.2658E-05	0.01864155	PRKACB,MAPK1,PIK3R1,GNB5,KRAS,KLB,EP300,GNB1,GNB4,MAPK3,SOS1,PLCB1,AKT3,PRKCE,MAP2K1,FRS2,CAMK2B,GNAS,RRAS,GRIA2,ADCY6,TBP,POLR2H,PLCG1,PLCL2,ITPR1,CREB5,GRM7,GNAI3,CALM1 (includes others),GAB1,IRS1,GNB2,PRKAG2,PRKCH
ErbB2-ErbB3 Signaling	4.6774E-05	0.02044002	MAPK1,RRAS,PIK3R1,KRAS,ERBB3,STAT3,KLB,CCND1,PTEN,MYC,GAB1,FOXO1,IRS1,MAPK3,SOS1,ERBB2,FRS2,MAP2K1

Regulation of eIF4 and p70S6K Signaling	4.6774E-05	0.02044002	MAPK1,PPP2CA,PIK3R1,KRAS,EIF4A2,EIF2S1,KLB,MTOR,EIF4G2,MAPK3,SOS1,PAIP1,AKT3,PPP2R5C,MAP2K1,FRS2,ITGA4,PABPC1,RPS6KB1,RRAS,RPS10,EIF4G3,RPS29,PPP2R1A,GAB1,PPP2R3A,IRS1,AGO3,EIF4A1,PPP2R1B,AGO4
IL-6 Signaling	5.2481E-05	0.02293407	MAP2K4,CSNK2A1,MAPK1,PIK3R1,SRF,KRAS,JAK2,KLB,VEGFA,NFKBIA,MAPK3,SOS1,MAP3K7,AKT3,CHUK,MAP2K1,IL1RAP,FRS2,MCL1,CXCL8,MAP3K14,RRAS,MAPK9,STAT3,CEBPB,GAB1,IRS1
Growth Hormone Signaling	5.3703E-05	0.0234683	RPS6KB1,MAPK1,PIK3R1,SOCS6,RPS6KA3,SRF,PLCG1,RPS6KA5,STAT3,JAK2,KLB,GAB1,IRS1,MAPK3,IGF1R,PRKCE,RPS6KA4,PRKCH,RPS6KA2,FRS2
Estrogen Receptor Signaling	6.166E-05	0.0269452	MAPK1,KRAS,NR3C1,EP300,DDX5,CDK8,KAT2B,MAPK3,SOS1,CTBP2,MAP2K1,TAF2,TAF9,SRC,MED1,RRAS,TBP,POLR2H,NCOA3,TAF6L,H3F3A/H3F3B,TAF5,NCOR2,MED13L,ESR1,CARM1,TAF15
CXCR4 Signaling	6.7608E-05	0.02954483	MAP2K4,MAPK1,PIK3R1,GNB5,KRAS,CRK,KLB,GNB1,GNB4,RHOT1,MAPK3,PLCB1,AKT3,PRKCE,ARHGEF11,MAP2K1,FRS2,SRC,GNAS,RHOC,RRAS,CXCR4,ADCY6,MAPK9,ITPR1,GNAI3,RND3,GAB1,IRS1,RHOA,GNB2,PRKCH
Antiproliferative Role of TOB in T Cell Signaling	7.5858E-05	0.03314986	PABPC1,TGFBR2,SMAD2,RB1,CCNE2,CCNE1,TGFBR1,MAPK1,CUL1,TOB1
Rac Signaling	7.7625E-05	0.03392199	MAP2K4,RPS6KB1,ABI2,MAP3K11,MAPK1,ARPC5L,RRAS,PIK3R1,PIKFYVE,KRAS,PIP5K1B,KLB,LIMK1,MCF2L,ARPC1A,GAB1,CDC42,IRS1,MAPK3,RHOA,CD44,MAP2K1,FRS2,PIP4K2C,ITGA4
Thrombopoietin Signaling	8.9125E-05	0.03894767	MAPK1,RRAS,PIK3R1,PLCG1,KRAS,STAT3,JAK2,KLB,MYC,GAB1,MAPK3,IRS1,SOS1,PRKCE,PRKCH,FRS2,MAP2K1
Induction of Apoptosis by HIV1	8.9125E-05	0.03894767	TP53,MAP2K4,MAP3K14,SLC25A13,SLC25A4,CXCR4,MAPK9,MAP3K5,XIAP,FAS,BCL2,DAXX,NFKBIA,CHUK,CASP8,BIRC3

Synaptic Long Term Potentiation	9.1201E-05	0.03985488	PRKACB,MAPK1,RRAS,GRIA2,PLCG1,KRAS,ITPR1,PLCL2,CREB5,RAP1A,EP300,GRM7,CALM1 (includes others),PPP1CC,PPP3CB,PPP1R10,MAPK3,PPP3R1,PRKAG2,PLCB1,PRKCE,PRKCH,MAP2K1,PPP3CA,CAMK2B
PI3K Signaling in B Lymphocytes	9.1201E-05	0.03985488	FYN,MAPK1,ATF5,NFATC3,PIK3R1,KRAS,PTEN,NFAT5,NFKBIA,PPP3CB,MAPK3,PPP3R1,FOXO3,AKT3,PLCB1,CHUK,MAP2K1,PPP3CA,CAMK2B,RRAS,PLCG1,PLCL2,ITPR1,CALM1 (includes others),CD40,IRS1
P2Y Purigenic Receptor Signaling Pathway	9.3325E-05	0.0407832	PRKACB,MAPK1,PIK3R1,GNB5,KRAS,KLB,EP300,GNB1,MYC,GNB4,MAPK3,PLCB1,AKT3,PRKCE,MAP2K1,FRS2,RRAS,ADCY6,PLCG1,PLCL2,CREB5,GNAI3,GAB1,IRS1,PRKAG2,GNB2,PRKCH

Note: The adjusted p-values were calculated by Bonferroni method. © 2000-2016 QIAGEN. All rights reserved.

Figure S1

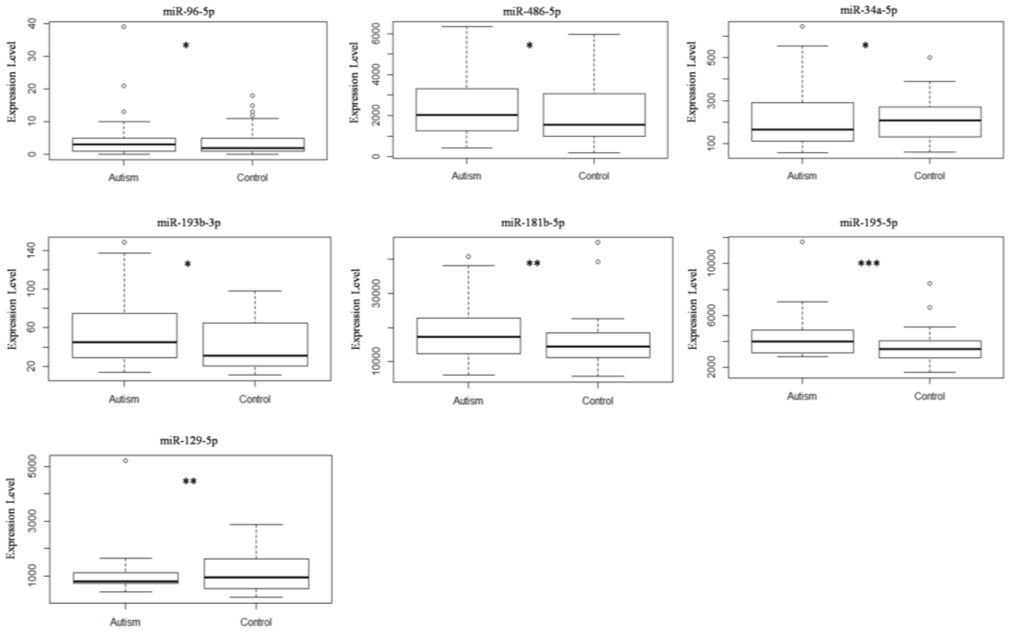

Figure S1: Box-plots for the identified miRNA biomarkers with different expression levels in the study by Wu et al [1]. Here the miRNA expression data and statistical significance (p-value) were retrieved from Wu's study, where the linear or linear mixed-effects model was performed on different brain samples of autism patients as well as normal controls. *: reported p-value ≤ 0.05; **: reported p-value ≤ 0.01, ***: reported p-value ≤ 0.001.

Reference:1 Wu, Y. E., Parikshak, N. N., Belgard, T. G. & Geschwind, D. H. Genome-wide, integrative analysis implicates microRNA dysregulation in autism spectrum disorder. *Nature neuroscience* **19**, 1463-1476, doi:10.1038/nn.4373 (2016).
